# Synthesis of Carbohydrate Based Macrolactones and Their Applications as Receptors for Ion Recognition and Catalysis

**DOI:** 10.3390/molecules26113394

**Published:** 2021-06-03

**Authors:** Surya B. Adhikari, Anji Chen, Guijun Wang

**Affiliations:** Department of Chemistry and Biochemistry, Old Dominion University, Norfolk, VA 23529, USA; sbadhika@odu.edu (S.B.A.); achen@odu.edu (A.C.)

**Keywords:** glycomacrocycles, glucosamine, macrolactonization, click chemistry, triazole, copper catalyst, anion binding

## Abstract

Glycomacrolactones exhibit many interesting biological properties, and they are also important in molecular recognitions and for supramolecular chemistry. Therefore, it is important to be able to access glycomacrocycles with different sizes and functionality. A new series of carbohydrate-based macrocycles containing triazole and lactone moieties have been designed and synthesized. The synthesis features an intramolecular nucleophilic substitution reaction for the macrocyclization step. In this article, the effect of some common sulfonate leaving groups is evaluated for macrolactonization. Using tosylate gave good selectivity for monolactonization products with good yields. Fourteen different macrocycles have been synthesized and characterized, of which eleven macrocycles are from cyclization of the C1 to C6 positions of *N*-acetyl D-glucosamine derivatives and three others from C2 to C6 cyclization of functionalized D-glucosamine derivatives. These novel macrolactones have unique structures and demonstrate interesting anion binding properties, especially for chloride. The macrocycles containing two triazoles form complexes with copper sulfate, and they are effective ligands for copper sulfate mediated azide-alkyne cycloaddition reactions (CuAAC). In addition, several macrocycles show some selectivity for different alkynes.

## 1. Introduction

**Figure 1:**
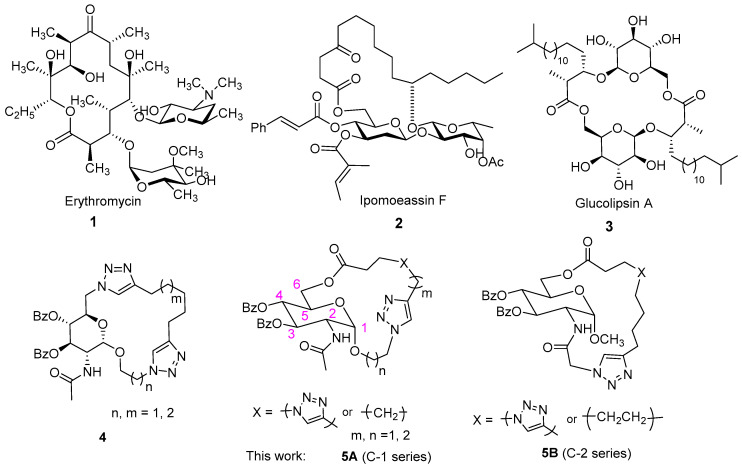
Structures of several carbohydrate-based macrocycles.

Macrocyclic compounds are important classes of molecules with many biological applications and useful for drug discovery [1,2]. Carbohydrate-based macrocyclic compounds have unique molecular architectures and many practical applications [3,4]. Naturally existing and synthetic carbohydrate-based macrocycles have been utilized in drug discovery, molecular recognition, and as advanced functional materials [1,3,5,6,7]. Among the different classes of macrocycles, macrolactones are especially unique compounds that exhibit biological activities and often function as enzyme inhibitors; they also showed applications in molecular recognition for supramolecular chemistry. For example, macrolactones synthesized from cholesterol derivatives have shown utility for molecular recognitions [8], and a synthetic macrodilactone exhibited enantioselective recognition of amino alcohols, as well as metal ions [9]. Naturally existing carbohydrate-based macrolactones or macrolides often exhibit many desirable biological activities for drug development [10,11,12]. The structures of several macrolactones are shown in Figure 1. Erythromycin (**1**) is a sugar-containing macrolide antibiotic. Ipomoeassina F (**2**) and analogs are macrolactones containing a disaccharide unit, which exhibit anticancer activities and recently have been identified as protein-translocation inhibitors [5,13]. Glucolipsin A (**3**) and analogs are natural products containing sugar dilactones—they are inhibitors for dual specific phosphatase Cdc25A [14]. This compound was synthesized from the corresponding hydroxy acids through a macrodilactonization reaction mediated by 2-chloro-1,3-dimethylimidazolinium chloride. 

Using a hexapyranose as the scaffold, a variety of synthetic macrolactones can be prepared depending on the cyclization patterns from different positions of the sugar ring. The lactonization could be introduced at an earlier stage and a different method for the macrocyclization utilized later [12,15]. For instance, the glycomacrolactones with azobenzene functionality have been synthesized through the cyclization from the C-2 to C-3 or C-4 to C-6 positions of D-glucose and D-mannose derivatives. These glycomacrolactones have exhibited applications in supramolecular chemistry for the design of chemosensors and other functional molecular assemblies [16,17]. A macrocyclic sialyl Lewis X (SLeX) mimic synthesized by lactonization via Mitsunobu reaction from the C-1 to C-6 position of the L-galactoside, was 1000 fold more potent for the inhibition of P-selectin than the tetrasaccharide SLeX [18]. 

Triazole-based macrocyclic frameworks are important structural features in many bioactive compounds and pharmaceuticals [19,20,21]. Using Cu (I) catalyzed azide-alkyne cycloaddition reactions (CuAAC), the “click chemistry”, many classes of 1,4-disubstituted 1,2,3-triazole derivatives have been synthesized and studied. There are also many studies towards the effective transformation of [3+2] azide-alkyne cycloaddition reactions, and triazole functions have been used as ligands for catalysis, including the CuAAC reactions [22,23,24,25,26]. Besides being medicinally important, the macrocycles containing the 1,2,3-triazole moieties are also useful in forming host-guest complexes and recognitions for ions [27,28,29,30]. The 1,2,3-triazole functional groups can act as Lewis bases to coordinate with metal cations, and the triazoles can also recognize halides through the triazole C-H---anion hydrogen bonds [30,31]. Triazole-containing macrocycles have shown interesting binding properties for both anions and cations; some of them are also ditopic ion-pair receptors, typically with rigid aromatic structures [31]. 

The synthesis of triazole-containing glycomacrocycles can be accomplished by many different methods. Using click chemistry, several series of glycomacrocycles have been synthesized and analyzed [32,33,34]. Several sugar-based bistriazole-containing macrocycles (**4**) were synthesized, and they are effective ligands in accelerating the CuSO_4_ mediated azide-alkyne cycloaddition (AAC) reactions [35]. The macrocycles with different ring sizes showed different selectivity and catalytic effect for 1-octyne and phenylacetylene. Because of the importance and potential applications of triazole-based glycomacrocycles, in this study, we designed and synthesized two series of macrolactones with the general structures **5A** and **5B**, as shown in Figure 1. 

These compounds contain either one or two triazole moieties and are expected to have properties in binding to guest molecules or ions, resulting in useful structures and applications. 

## 2. Results and Discussion

**Scheme 1:**
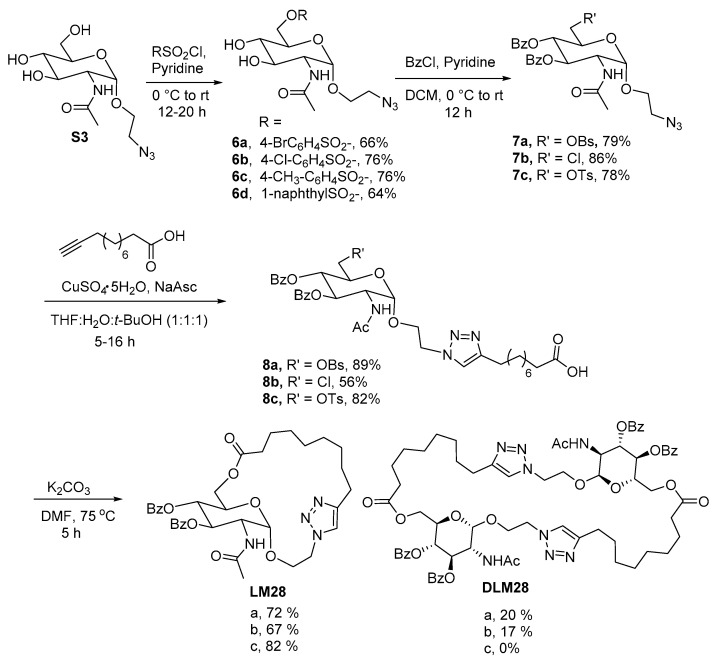
Macrolactonization using different sulfonates as the leaving groups.

As shown in Scheme 1, the macrolactones with general structures **5A** can be synthesized starting from the intermediate **3S**, which was prepared from *N*-acetyl-D-glucosamine (NAG) [35]. By installing a suitable leaving group at the C-6 position and a nucleophile, such as a long-chain and triazole-containing carboxylate linked to the anomeric center, an intramolecular S_N_2 reaction of the carboxylate with the leaving group is expected to form the macrolactones. The carboxylate intermediate can be prepared using click chemistry to include one or two triazole functional groups. 

To test the feasibility of this route to synthesize the glycomacrolactones, several different sulfonate leaving groups towards the macrocyclization were evaluated to obtain suitable conditions for the cyclization reactions. Common aryl sulfonyl chlorides were selected for the derivatiazation of the C-6-primary hydroxyl group of **S3**—these include *p*-bromobenzene-, *p*-chlorobenzene-, *p*-tolyl-, and 1-naphthyl-sulfonyl chloride. Methanesulfonyl chloride was not selected, since it typically reacts with both the C-4 and C-6 hydroxyl groups readily [36]. The C-6 primary hydroxyl group of **S3** was then converted to different sulfonate leaving groups selectively to afford **6a–6d**. The intermediates **6a–6c** were treated with benzoyl chloride to afford the corresponding dibenzoates **7**, with both the C-3 and C-4 positions benzoylated. However, the naphthyl derivative **6d** afforded mainly the C-3 monobenzoylation product, presumably due to steric hindrance. 

**Scheme 2:**
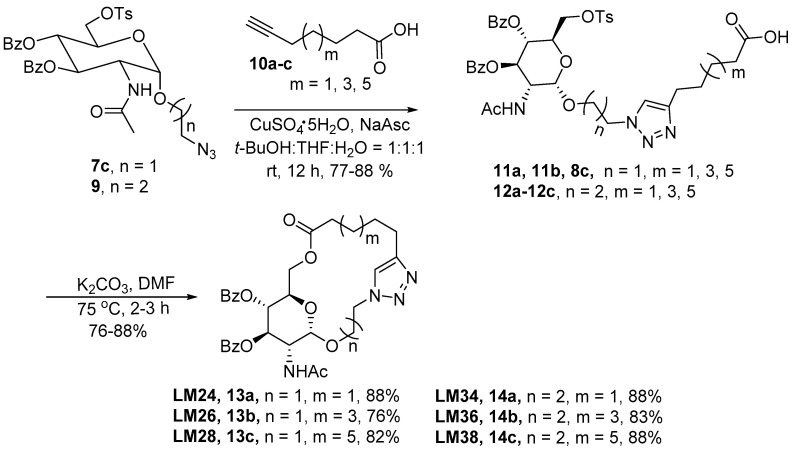
Synthesis of monotriazole-based macrocycles.

**Scheme 3:**
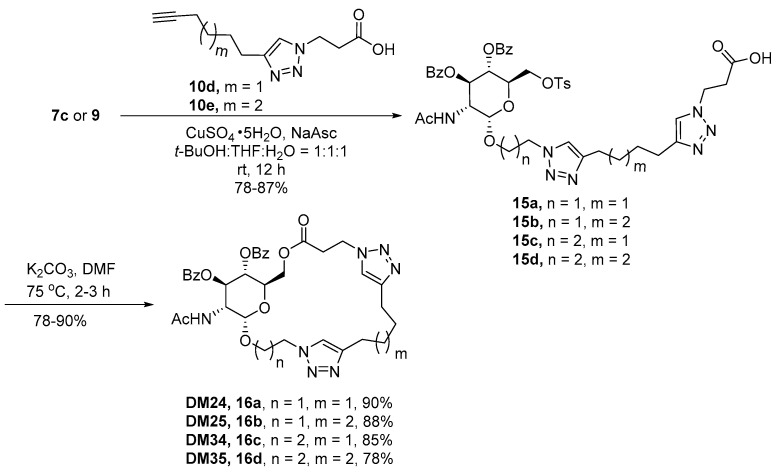
Synthesis of bis-triazole-based macrocycles.

The 4-bromobenzenesulfonate **6a** was converted to the corresponding dibenzoate **7a** followed by a click reaction to afford the intermediate **8a**. The intramolecular macrolactonization reaction led to the monomeric lactone **LM28** in 72% yield, together with 20% dimeric lactonization product **DLM28**. The 4-chlorosulfonate **6b** was converted to chloride during the benzoylation step to afford the chloride **7b**. Using a similar protocol, compound **7b** was converted the **LM28** in 67% yield together with 17% **DLM28**; and the tosylate **8c** led to 82% **LM28** and no dimer was isolated. Based on these, the tosyl group was superior to other leaving groups, and this was selected to synthesize a series of other macrocycles containing monotriazole and bis-triazoles (Scheme 2 and Scheme 3). 

As shown in Scheme 2, treating **7c** and **9** [35] with different alkynoic acids **10a-c** utilizing click reaction gave the carboxylic acids **11a–12c**. The intramolecular S_N_2 reaction was carried out using K_2_CO_3_ as the base in DMF at 75 °C to afford monotriazole macrolactones **13a–13c**, **14a–14c** with different ring sizes in excellent yields. The synthesis of bis-triazole-containing macrolactones is shown in Scheme 3. Using a similar method and the triazole-containing alkynoic acids **10d-e**, the desired macrocycles **16a-d** were obtained in 78–90% yields. The concentrations of the cyclization step for both monotriazole and bis-triazole-containing macrocycles were typically ~10 mM or 0.01 M of the reactants, which are not super dilute conditions; therefore, the method should be practical for possible large-scale synthesis.

Besides using DMF as the solvent, for the intramolecular lactonization reaction, acetonitrile was also used as the solvent; similar macrolactonization results were typically obtained through the reactions that required a longer time to complete. The monotriazole derivative **LM28** was obtained in 77% yield in acetonitrile (9 mM concentration 6 h). The bistriazole-based macrocycles were also prepared in acetonitrile overnight to afford the macrocycle products in 71–78% yields. The typical concentration for cyclization for small scale reactions was about 4–6 mM; though the yields were slightly lower than those reactions when DMF was used, the lower boiling point of acetonitrile makes it easier to remove during work up. In addition, in acetonitrile, no dimerization was observed, perhaps due to reduced nucleophilicities of the carboxylate in acetonitrile than DMF. 

**Scheme 4:**
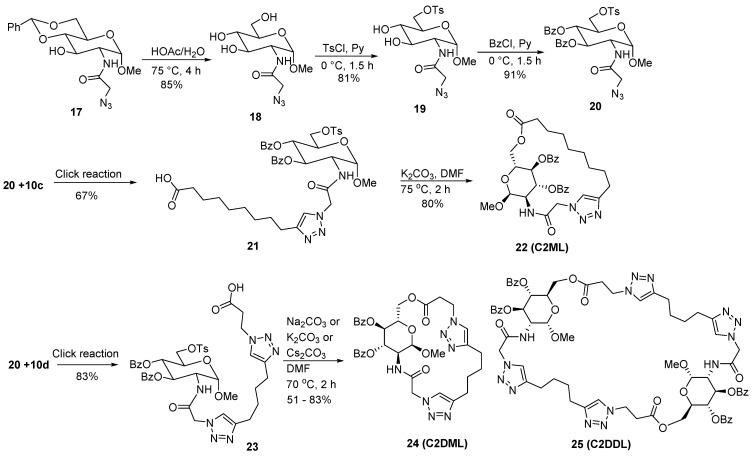
Synthesis of macrocycles **5B** by cyclization from C2 to C-6 positions.

Using the macrolactonization method developed above, several macrocycles with the general structure **5B** can be synthesized. As shown in Scheme 4, the azido sugar derivative **17** [37] was converted to the key intermediate tosylate **20** in three steps. For the benzoylation step of compound **19**, pyridine was found to be a more effective solvent. The yields of the dibenzoylated products improved from 74% in DCM/pyridine to 91% in pyridine. The intermediate azide **20** was treated with different alkyne-containing acids to generate the precursors for macrolactonizations. As a proof of principle, here, only two alkynyl acids **10c** and **10d**, were used. The intermediate **21** was converted to the monolactonziation product **22** in good yield under similar conditions. Which indicated that the macrolactonization methods are applicable to the synthesis of glycomacrolactones of different positions to the C-6. 

The bistriazole derivative **23** was subjected to macrolactonization using different bases to obtain suitable conditions for monocyclization. Three different carbonate bases in anhydrous DMF were screened, and the results are shown in Table 1. It seems that the cations used can affect the ratio of the monomer versus the dimer; all conditions showed 100% conversion of the starting materials based on the disappearance of the tosylate signal. Using sodium and potassium carbonate, the monolactone **24** was obtained, and when using cesium carbonate, mainly the dimeric-lactone **25** was isolated. In 5% MeOH/DCM, the monolactone was slightly more polar than the dilactone, which showed an opposite trend to the C1-series. Using either Na_2_CO_3_ or K_2_CO_3_, the monolactonization products were obtained in good yields for the C2 series macrocycles. 

### 2.1. Anion Binding Studies of the Macrocycles with Tetrabutyl Ammonium Halides 

As mentioned earlier, triazole derivatives in rigid macrocycles have shown strong binding to chloride ions from the C-H bond [38,39]. In order to understand the anion binding capacities of the macrocycles, several representative macrocycles, including the C1 series monotriazole **LM28**, bistriazole **DM35**, and C-2 series monotriazole **22** and bistriazole **24**, were studied. The tetrabutylammonium halides (TBAX), including TBACl, TBABr, and TBAI, were used to study halide binding. The ^1^H NMR spectra of the macrocycles with different amounts of halides were obtained, and possible anion recognitions were analyzed. The structures and ^1^H NMR spectra of several representative macrocycles with TBACl are shown in Figure 2, Figure 3, Figure 4 and Figure 5 and Figures S1–S22 on pages S80–S90.

**Figure 2:**
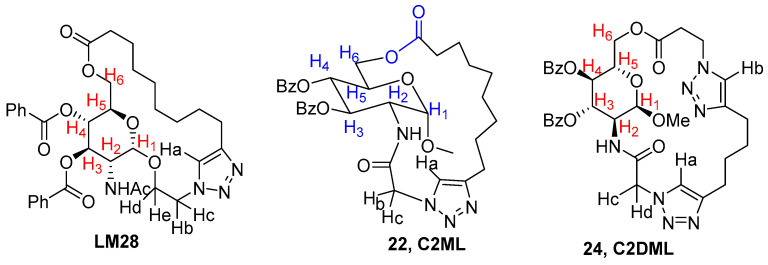
Structures of several macrocycles and the protons assigned.

**Figure 3:**
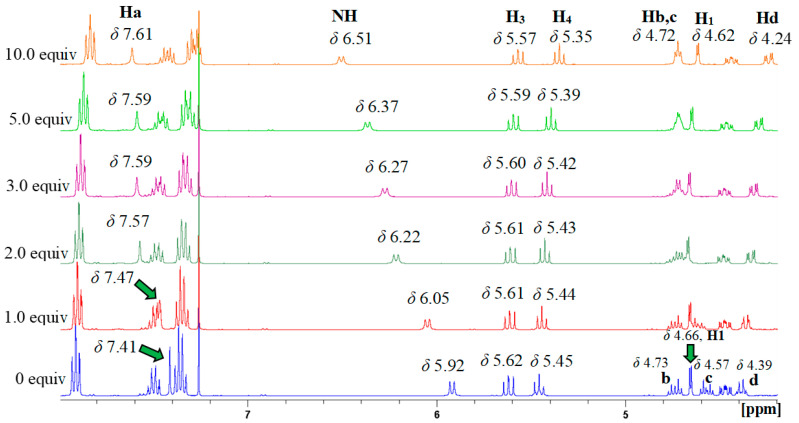
^1^H NMR spectra of **LM28** with different amounts of TBACl from 0 to 10.0 equiv.

The ^1^H NMR spectra of **LM28** with TBACl are shown in Figure 3, Figures S1 and S2. The triazole C-H and the amide NH signals of **LM28** showed significant chemical shift changes upon adding halides. From 0 to 10.0 equiv of TBACl, the NH showed the most significant downfield shift of 0.59 ppm—this indicated strong hydrogen bonding with the chloride. The triazole signal also shifted downfield, with 2.0 equiv of TBACl, the Ha moved downfield by 0.16 ppm; interestingly, this signal only shifted another 0.04 ppm when 10.0 equiv of chloride was added. The significant downfield change of triazole and NH signals with increasing amounts of chloride indicated that they were affected by forming hydrogen bonds with chloride and by anion-π interactions. Other protons were less affected by chloride additions and showed small upfield shifts. The anomeric proton shifted upfield by 0.04 ppm, H_3_ by 0.05 ppm, and H_4_ by 0.10 ppm. Without chloride, the Hb, Hc, and Hd are multiplets centered at *δ* 4.73, 4.57, and 4.39 ppm, respectively, and the Hb and Hc each showed doublet of triplet splitting pattern (dt). However, with 10.0 equiv of TBACl, the Hb and Hc merged to a pseudo triplet. These changes indicated that the chloride binding to the ring caused significant chemical and magnetic environment changes for the macrocycle protons. The anion binding did not affect the conformation of the sugar ring much, but did change the dihedral angles of anomeric methylene groups (Figure 2). 

The ^1^H NMR spectra of **LM28** with TBABr and TBAI are shown in Figures S3–S8; the patterns are similar to those of chloride, but with much smaller chemical shift changes. From 0 to 5.0 equiv of TBABr, the triazole signal showed about 0.10 ppm downfield shift, and the NH signal shifted downfield by 0.19 ppm, the anomeric proton showed a small upfield shift of 0.03 ppm. These results showed that the bromide also forms hydrogen bonds with the triazole hydrogen, amide hydrogen cooperatively. But the bromide binding is apparently weaker than chloride. TBAI had even less influence on chemical shift changes (Figures S6–S8). From 0 to 5.0 equiv of TBAI, the triazole and the NH signals only showed small downfield changes of 0.03 and 0.06 ppm, respectively. This indicated that iodide didn’t form strong interactions with the triazole and the macrocycle. 

The ^1^H NMR spectra of **DM35** with different TBAX are shown in Figures S9–S17. For 0 to 5.0 equiv of TBACl (Figures S9–S11), the amide NH signal showed a 0.14 ppm downfield shift, and the anomeric proton showed a similar upfield change of 0.04 ppm. The triazole signals showed a small downfield shift of 0.04 ppm when adding up to 2 equiv of TBACl (Figure S10); however, not much change was observed after adding additional TBACl. This could be due to the conformation change caused by the anion binding with the sugar ring. A similar trend was observed for TBABr, as shown in Figures S12–S14, from 0 to 5 equiv of TBAB, the NH signal moved downfield by 0.04 ppm, and the anomeric proton showed an upfield change of 0.03 ppm. The triazole signals showed concentration dependence. Upon addition of one equiv of TBABr, the two triazole signals showed 0.08 ppm downfield change; after further addition of TBABr, the triazole signals did not move (Figure S13). This indicates that possibly one bromide is hydrogen bonding with both triazole protons, and bromide formed stronger interactions than chloride did. TBAI did not cause significant chemical shift changes (Figures S15–S17). 

**Figure 4:**
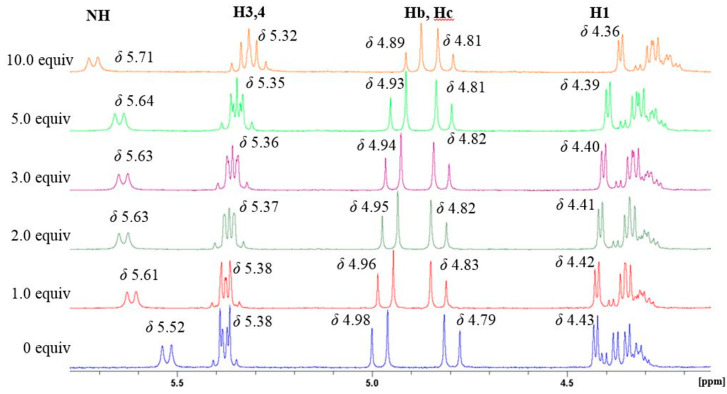
^1^H NMR spectra of C2-monotriazole compound **22** at different concentrations of TBACl.

The ^1^H NMR spectra of the C-2 series macrocycles **22** are shown in Figure 4, Figures S18 and S19. The triazole signal showed a small downfield (0.03 ppm) change after adding one equiv of TBACl. From 0 to 10.0 equiv of TBACl, the amide NH showed a larger downfield change of 0.19 ppm, the other protons from the sugar ring also showed significant changes (Figure 4). The anomeric proton showed an upfield shift of 0.07 ppm. The protons at C-3 and C-4 positions became more resolved into two triplets, and the methylene Hb and Hc merged from two separate doublets to a pseudo quartet. This indicated the chemical environment of the two protons become very similar. 

**Figure 5:**
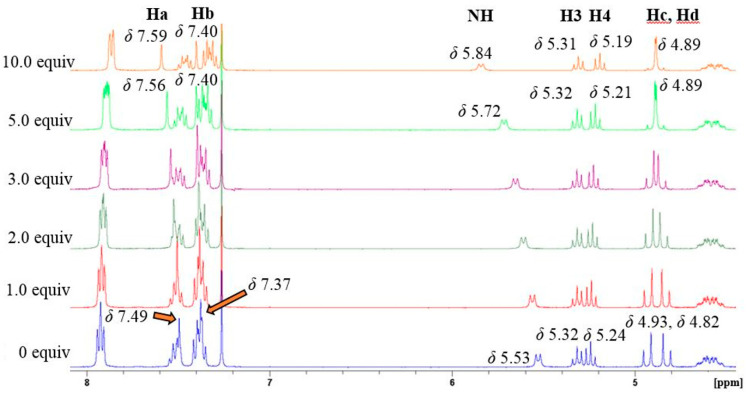
The ^1^H NMR spectra of compound **24** with different amounts of TBACl.

The C2-bistriazole macrocycle **24** binding to chloride was also studied, as shown in Figure 5 and Figures S20–S22. The triazole, anomeric, NH, and many other signals changed significantly upon adding TBACl. From 0 to 10.0 equiv of TBACl, the triazole proton Ha at 7.49 ppm shifted downfield by 0.10 ppm; and Hb at 7.37 shows a small downfield (0.03 ppm) change, the amide NH shifted downfield by 0.31 ppm. This indicated that the amide participated in intermolecular hydrogen bonding significantly, and one of the triazole hydrogen atoms also formed a stronger hydrogen bond with chloride than the other. The H_3_ and H_4_ appeared as a pseudo triplet; H_4_ shifted upfield by 0.05 ppm, while H_3_ stayed the same. The methylene Hc and Hd changed from a pseudo quartet to a broad singlet, indicating that they have a similar chemical and the magnetic environment with 5.0–10.0 equiv of TBACl. These shifts showed that the macrocycle can bind to chloride, and the binding of anions resulted in a conformational change of the macrocycle.

**Figure 6:**
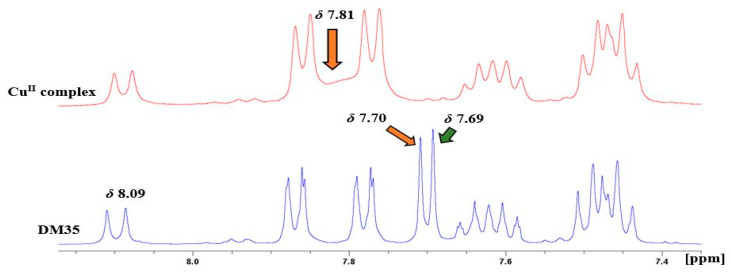
The ^1^H NMR spectra of **DM35** and its Cu^II^ complex with CuSO_4_∙5H_2_O in *d*_6_-DMSO.

The binding properties of the macrocycles **LM28**, **DM35** with Cu^2+^ were also studied using several macrolactones and ^1^H NMR spectroscopy at variable temperatures. The ^1^H NMR spectra of **DM35** and its complex with CuSO_4_∙5H_2_O are shown in Figure 6 and Figures S23–S25. In the complex, the two sharp triazole singlets at 7.70 and 7.69 ppm disappeared or became broadened and shifted downfield to around 7.81 ppm. The triazole signals were very broadened due to the paramagnetic properties of Cu (II) binding to triazole [40]. Typically, protons within 0.9 nm of the Cu (II) were not observed due to fast paramagnetic relaxation, and the protons in the outer shell between 0.9–1.7 nm from the copper showed chemical shift changes and broad resonances [41]. The signals far away from Cu (II) were not affected as much (Figure S23). This showed that the copper ion is in close vicinity to the triazoles. 

**Figure 7:**
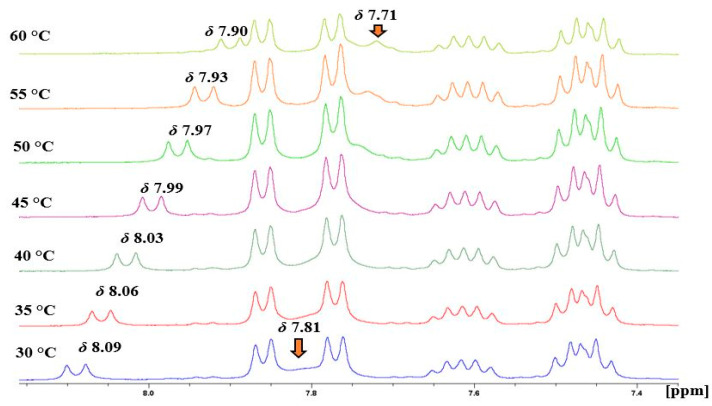
Stacked ^1^H NMR spectra of **DM35** complex with CuSO_4_∙5H_2_O at different temperatures. The signal for triazole and amides are labeled.

The ^1^H NMR spectra of the copper complex with **DM35** were also evaluated at different temperatures, from 30 to 60 °C (Figure 7, Figures S24 and S25), the triazole signals showed upfield shift to a broad signal, but more defined peak at around 7.71 ppm, this is close to the chemical shifts of macrocycle without Cu (II), which indicates that the Cu (II) is more dissociated from the binding to the two triazole nitrogen atoms at elevated temperatures. The anomeric proton appeared broad singlet at 30 °C, at 4.79 ppm, and shifted to 4.81 to a doublet (Figure S24a). The amide NH signal shifted upfield at higher temperature indicates that amide participates in intermolecular hydrogen bonding. The process is reversible as when the same NMR sample was cooled from 60 to 30 °C (Figure S25), the signals for triazoles and amide reverted to the original pattern. 

The ^1^H NMR spectra of the complex of **DM35** with Cu(OAc)_2_ are shown in Figures S26–S28. These are similar to the copper sulfate complex, but showed more significant broadening; this indicated that the macrocycle formed a strong complex with the Cu (II) through interactions with the nitrogen of the triazole ring, and the copper ion is in close proximity with several other hydrogens. The complex of the monotriazole **LM28** with CuSO_4_∙5H_2_O (Figure S29) showed a similar trend; the triazole signal appeared as a broad singlet, but still visible, unlike in the bistriazole complexes. The ^1^H NMR spectra of the copper complex at different temperatures showed some chemical shift changes for the NH and triazole signals (Figure S31). These results showed that for monotriazoles, such as **LM28**, the copper ion was not bonded to the triazole as tightly as comparing to bistriazoles. The complex of the C2 bistriazole derivative **24** with CuSO_4_∙5H_2_O was also prepared and the ^1^H NMR spectra showed similar trends (Figures S32–S34).

### 2.2. Effect of the Macrocycles as Ligands for CuAAC Reactions 

**Scheme 5:**
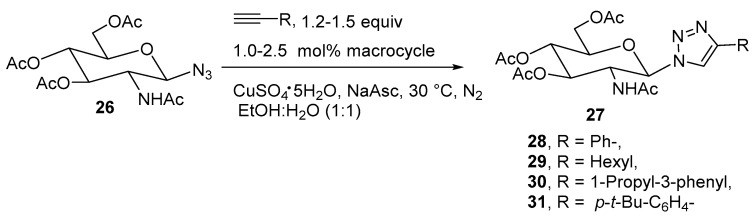
Cycloaddition reactions of sugar azide with different acetylenes.

Besides the binding studies, the effect of these macrocycles on CuAAC reactions was also studied as shown in Scheme 5. Using the anomeric sugar azide **26** as the substrate, a series of reactions were carried out with different alkynes, including the aliphatic alkyne 1-octyne, aromatic alkynes, such as phenylacetylene, 4-*t*-Bu-phenylacetylene, and 5-phenyl-1-pentyne. The results are summarized in Table 2, Table 3, Table 4 and Table 5 and SI Tables S1–S8, Figures S35–S42.

For phenylacetylene, when using 2.5 mol% macrocycle and copper sulfate as the catalyst (Table 2a), at 2 h, all C1-series macrocycles (MCs), including both monotriazole and bis-triazole derivatives, were able to accelerate the reaction to over 50% conversion versus 15% conversion without the macrocycles. The most effective macrocycles are the bistriazole macrocycles **DM25** and **DM35**, which reached 100% conversion within 5 h. The reactions in the presence of the monotriazole derivatives **LM34**, **LM36**, **LM26** reached over 75% conversion at 5 h and almost completed after 9 h. When the monotriazoles (Table 2b) were increased to 5.0 mol%, **LM26** and **LM34** were both effective in catalyzing the reactions, reaching almost full conversions at 5 h. The C2 series macrocycles were not as effective as the C-1 series; only the bistriazole derivative **C2DML** (**24)** showed some acceleration for the reaction.

When using 1-octyne as the substrate, different macrocycles showed very different results. As shown in Table S3, with 2.5 mol% macrocycles, 1.2 equiv of 1-octyne and 0.1 equiv of CuSO_4_, only **DM34** was effective at catalyzing the reaction, reaching 93% conversion at 2 h; the other macrocycles didn’t show improvement over the control experiment. When increasing the 1-octyne from 1.2 to 1.5 equiv, the reaction completed within 1 h in the presence of 2.5 mol% of **DM34**. Increasing the loading of MCs for **LM26**, **LM34**, and **LM36** to 5.0 mol% did not improve the conversions (Table 3 and Table S4). The experiments confirmed that **DM34** was particularly efficient at catalyzing the click reaction of 1-octyne. 

5-Phenyl-1-pentyne was found to be much more reactive in comparison to the other alkynes screened. Several experiments were carried out to analyze the effect of the macrocycles with reduced copper loading. A few summarized results are shown in Table 4 and Tables S5 and S6. Without macrocycles, at 2 h the reaction of the alkyne with **26** reached 100% (0.1 equiv of CuSO_4_) and 51% conversion when using 0.05 equiv copper sulfate. Further reduction of copper to 1 mol%, at 2 h only 5% conversion was observed; however, with added 2.5 mol% of **DM35**, the reaction reached 52% conversion. From these results, we selected either 2.0 or 5.0 mol% of copper to evaluate the MC ligands. As shown in Table 4, 1.0–2.5% mol of the bis-triazole macrocycles were effective at accelerating the reactions significantly, reaching 100% conversion at about 1–5 h. When using 2 mol% Cu(OAc)_2_ as the catalyst, the control reaction reached 13% conversion, but the reaction with **DM35** reached 100% conversion at 2 h (Table S5). 

For 4-*t*-butylphenylacetylene (Table 5 and Tables S7 and S8), using 0.1 equiv of copper sulfate, the reactions were slow; only **DM25** and **DM35** helped the reaction to full conversion at 20 h; when the CuSO_4_ loading was increased, the reactions in the presence of the two MCs reached full conversions at 5 h. **DM34** and **DM24** were not that effective, giving similar conversion compared to the control experiment. **DM25** was much more efficient than the isomer **DM34**—apparently, the positions of the triazoles in the macrocycle had an influence towards the catalysis of the reaction. 

**Figure 8:**
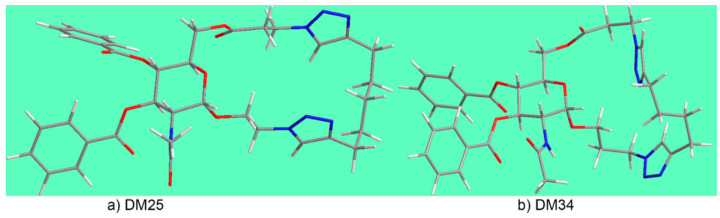
The energy minimized conformations of (**a**) **DM25** and (**b**) **DM34**.

The energy minimized conformations of two macrocycles **DM25** and **DM34** are shown in Figure 8 [35]. These two compounds have the same molecular weight and ring sizes—the only difference is that the triazole is located at a different position to the anomeric center. The two MCs adopted quite different conformations in which the triazoles rings are more parallel in **DM25**, but at a dihedral angle about 30 degrees to each other for **DM34**. The two triazole rings can adopt different conformations upon binding with copper, and possibly have a cooperative effect when both triazole rings are embedded in the macrocycles. The conformation difference of these MCs perhaps is correlating with the selectivity among different alkynes. **DM25** is more selective for phenylacetylene; it is also more effective for *t*-Bu-phenylacetylene, while **DM34** was the most effective MC for 1-octyne, but not as good as other MCs for phenylacetylenes. The interesting selectivity towards different alkynes may be useful in differentiating acetylenes when reacting with the azide; this could be useful for other selective reactions. 

## 3. Experimental Section

### General Methods 

All reactions were carried out under normal conditions, reagents and solvents were obtained commercially from Sigma-Aldrich, VWR, and Fisher and used directly without purifications. All reactions, unless otherwise noted, were carried out in oven-dried glassware under a nitrogen atmosphere. All purifications were conducted by flash chromatography using 230–400 mesh silica gel with a gradient of solvent systems. Thin-layer chromatography (TLC) analysis was performed with aluminum-backed TLC plates with UV and fluorescence indicator and visualized using UV lamp at 254 nm, then stained with PMA solution. ^1^H NMR and proton-decoupled ^13^C NMR spectra were obtained with Bruker 400 MHz NMR spectrometer in DMSO-*d*_6_, D_2_O, or CDCl_3_. The chemical shifts were reported using CDCl_3_/DMSO-*d*_6_ as internal standard at 7.26/2.50 ppm and at 77.0/39.5 ppm, respectively. 2D NMR experiments (HSQC, COSY) were also conducted to assist the compound characterizations. Melting point measurements were carried out using a Fisher Jones melting point apparatus. The molecular mass was measured using LC-MS on an Agilent LC1260 system and 6120B Single Quad Mass Spectrometer or with Shimadzu LCMS-2020. HRMS data were obtained using positive electrospray ionization on a Bruker 12T APEX-Qe FTICR-MS with an Apollo II ion source. 

For compounds synthesized by similar methods, the procedures for the first compound are included in detail. For the rest, only the amount used, purification method, and the characterization data are provided. 

*Synthesis of compound **6a***. Compound **S3** (200.0 mg, 0.69 mmol, 1.0 equiv) was added to a 50 mL round bottomed flask (RBF) with a drying tube and nitrogen balloon, pyridine (4.0 mL) was added and the flask was cooled to 0 °C, then 4-bromobenzenesulfonyl chloride (352.1 mg, 1.37 mmol, 2.0 equiv) was added and the mixture was stirred at 0 °C for 20 min and the ice bath was removed. The mixture was stirred at rt for 20 h, at which time ^1^H NMR spectrum showed about 95% conversion. The reaction was stopped, and solvent was removed, the crude product was purified on silica gel using a gradient of dichloromethane (DCM) and methanol, from DCM up to 10% MeOH/DCM (R_f_ = 0.31 in 10% MeOH/DCM). The desired product **6a** was obtained as a colorless liquid (232.0 mg, 66%). ^1^H NMR (400 MHz, CDCl_3_) *δ* 7.78 (d, *J* = 8.6 Hz, 2H), 7.69 (d, *J* = 8.6 Hz, 2H), 6.21 (d, *J* = 8.8 Hz, 1H), 4.78 (d, *J* = 3.6 Hz, 1H), 4.38–4.29 (m, 2H), 4.11–4.04 (m, 1H), 3.87–3.78 (m, 2H), 3.71–3.65 (m, 1H), 3.60–3.54 (m, 1H), 3.53–3.46 (m, 2H), 3.37–3.29 (m, 1H), 2.02 (s, 3H); ^13^C NMR (100 MHz, CDCl_3_) *δ* 172.3, 134.8, 132.6, 129.5, 129.2, 97.7, 73.4, 70.5, 69.9, 69.7, 67.2, 53.2, 50.5, 23.2. HRMS m/z calcd for C_16_H_21_BrN_4_O_8_SNa [M + Na]^+^ 531.0156, found 531.0155.

*Synthesis of compound **6b**.* Compound **S3** (100.0 mg, 0.35 mmol), pyridine (1.5 mL), 4-chlorobenzene sulfonyl chloride (147.7 mg, 0.69 mmol), 12 h. Purified by flash chromatography (DCM to 2% MeOH/DCM) to obtain a colorless liquid (122.0 mg, 76%) as the desired compound. (R_f_ = 0.27 in 10% MeOH/DCM). ^1^H NMR (400 MHz, CDCl_3_) *δ* 7.86 (d, *J* = 8.6 Hz, 2H), 7.53 (d, *J* = 8.6 Hz, 2H), 6.12 (d, *J* = 8.7 Hz, 1H), 4.79 (d, *J* = 3.6 Hz, 1H), 4.39–4.25 (m, 2H), 4.11–4.03 (m, 1H), 3.90–3.83 (m, 1H), 3.83–3.77 (m, 1H), 3.71–3.64 (m, 1H), 3.61–3.45 (m, 3H), 3.38–3.29 (m, 1H), 2.02 (s, 3H); ^13^C NMR (100 MHz, CDCl_3_) *δ* 172.2, 140.6, 134.3, 129.6, 129.4, 97.7, 72.8, 70.3, 69.9, 69.8, 67.2, 53.2, 50.4, 23.1. LC-MS (ESI+) calcd for C_16_H_22_ClN_4_O_8_S [M+H]^+^ 465 found 465. HRMS (ESI+) ([M + Na]^+^) m/z calcd for C_16_H_21_ClN_4_O_8_SNa, 487.0661, found 487.0665.

*Synthesis of compound **6d**.* Compound **S3** (200.0 mg, 0.69 mmol), pyridine (4.0 mL), 1-naphthalenesulfonyl chloride (312.4 mg, 1.37 mmol), 20 h to see 90% conversion. Purified by flash chromatography (DCM to 10% MeOH/DCM) to obtain a white foam (211.0 mg, 64%) as the desired product. (R_f_ = 0.33 in 10% MeOH/DCM). ^1^H NMR (400 MHz, CDCl_3_) *δ* 8.63 (d, *J* = 7.9 Hz, 1 H), 8.29 (dd, *J* = 8.4, 7.0 Hz, 1H), 8.13 (d, *J* = 8.2 Hz, 1H), 7.95 (d, *J* = 8.0 Hz, 1H), 7.74–7.55 (m, 3H), 5.92 (d, *J* = 8.3 Hz, 1H), 4.61 (d, *J* = 3.7 Hz, 1H), 4.40–4.31 (m, 1H), 4.28–4.20 (m, 1H), 3.98–3.89 (m, 1H), 3.90–3.67 (m, 2H), 3.64–3.54 (m, 1H), 3.49–3.34 (m, 3H), 3.27–3.18 (m, 1H), 2.01 (s, 3H); ^13^C NMR (400 MHz, CDCl_3_) *δ* 172.1, 135.3, 134.1, 131.1, 130.4, 128.8, 128.5, 128.4, 127.2, 125.0, 124.0, 97.4, 72.7, 70.4, 69.9, 69.8, 66.9, 53.1, 50.3, 23.1. HRMS m/z calcd for C_20_H_24_N_4_O_8_SNa [M + Na]^+^ 503.1207, found 503.1208.

*Synthesis of compound **7a**.* Compound **6a** (100.0 mg, 0.2 mmol, 1.0 equiv) was added to a 50 mL round bottom flask with a drying tube and nitrogen balloon attached, the reaction flask was cooled to 0 °C, then dichloromethane (2.5 mL), pyridine (5.0 equiv) and benzoyl chloride (0.06 mL, 0.5 mmol, 2.5 equiv) were added to the solution. The reaction mixture was stirred at to 0 °C and then stirred at rt for about 12 h. At this time, TLC and ^1^H NMR indicated full conversion to the product. The reaction was stopped, and solvent was removed under vacuum using a rotovap. The crude product was purified using flash chromatography on silica gel using a solvent gradient of ethyl acetate and hexane from 1:4 to 3:2 ratio. The desired product was obtained as a colorless viscous liquid (112.0 mg, 79% yield). (R_f_ = 0.38 in 5% MeOH/DCM). ^1^H NMR (400 MHz, CDCl_3_) *δ* 7.90–7.83 (m, 4H), 7.70 (d, *J* = 8.5 Hz, 2H), 7.58 (d, *J* = 8.5 Hz, 2H), 7.55–7.47 (m, 2H), 7.39–7.32 (m, 4H), 5.90 (d, *J* = 9.1 Hz, 1H), 5.64 (t, *J* = 10.9 Hz, 1H), 5.38 (t, *J* = 9.9 Hz, 1H), 4.97 (d, *J* = 3.6 Hz, 1H), 4.56–4.49 (m, 1H), 4.28–4.16 (m, 3H), 4.00–3.94 (m, 1H), 3.72–3.65 (m, 1H), 3.62–3.55 (m, 1H), 3.45–3.38 (m, 1H), 1.85 (s, 3H); ^13^C NMR (100 MHz, CDCl_3_) *δ* 170.1, 166.9, 165.1, 134.5, 133.7, 133.5, 132.5, 129.9, 129.8, 129.4, 129.3, 128.7, 128.5, 128.4, 97.6, 71.0, 68.8, 68.5, 68.4, 67.8, 52.0, 50.4, 23.0. HRMS m/z calcd for C_30_H_29_BrN_4_O_10_SNa [M + Na]^+^ 739.0680, found 739.0676.

*Synthesis of compound **7b***. Compound **6b** (100.0 mg, 0.22 mmol), DCM (3.0 mL) and benzoyl chloride (0.06 mL, 0.54 mmol), 12 h. The crude was purified using flash chromatography on silica gel using a solvent gradient of DCM to 3% MeOH/DCM) to obtain brown oil (96.0 mg, 86%) as the desired product. (R_f_ = 0.32 in 5% MeOH/DCM). ^1^H NMR (400 MHz, CDCl_3_) *δ* 7.96–7.87 (m, 4H), 7.53–7.45 (m, 2H), 7.40–7.30 (m, 4H), 5.96 (d, *J* = 9.3 Hz, 1H), 5.68 (t, *J* = 10.9 Hz, 1H), 5.47 (t, *J* = 9.9 Hz, 1H), 5.06 (d, *J* = 3.6 Hz, 1H), 4.65–4.55 (m, 1H), 4.26–4.18 (m, 1H), 4.12–4.03 (m, 1H), 3.78–3.71 (m, 1H), 3.71–3.63 (m, 2H), 3.63–3.56 (m, 1H), 3.48–3.39 (m, 1H), 1.86 (s, 3H); ^13^C NMR (100 MHz, CDCl_3_) *δ* 170.1, 167.0, 165.2, 133.6, 133.5, 129.83, 129.81, 128.7, 128.6, 128.5, 128.4, 97.5, 71.2, 70.5, 70.3, 67.7, 52.1, 50.4, 43.6, 23.0. LC-HRMS m/z calcd for C_24_H_25_ClN_4_O_7_Na [M + Na]^+^ 539.1304, found 539.1301.

*Synthesis of compound **8a**.* To a 50 mL RBF, **7a** (100.0 mg, 0.14 mmol, 1.0 equiv) in *t*-BuOH: THF: H_2_O (v:v:v 1:1:1, 3.0 mL) and 10-undecynoic acid (33.1 mg, 0.18 mmol, 1.3 equiv), CuSO_4_·5H_2_O (7.0 mg, 0.028 mmol, 0.2 equiv) and sodium ascorbate (NaAsc) (11.1 mg, 0.056 mmol, 0.4 equiv) was added sequentially as described and stirred at rt for 16 h. The reaction was monitored after 16 h by ^1^H NMR to see the consumption of starting material and TLC to see no starting material at all. Further purified by flash chromatography using DCM to 2% MeOH/DCM to obtain a white foam (112.0 mg, 89%) as the desired product. (R_f_ = 0.26 in 5% MeOH/DCM). ^1^H NMR (400 MHz, CDCl_3_) *δ* 7.88–7.79 (m, 4H), 7.68 (d, *J* = 8.6 Hz, 2H), 7.56 (d, *J* = 8.6 Hz, 2H), 7.53–7.44 (m, 3H), 7.38–7.30 (m, 4H), 6.15 (d, *J* = 9.0 Hz, 1H), 5.52 (t, *J* = 10.1 Hz, 1H), 5.32 (t, *J* = 9.8 Hz, 1H), 4.87 (d, *J* = 3.6 Hz, 1H), 4.60 (s, 2H), 4.53–4.44 (m, 1H), 4.20–4.06 (m, 3H), 4.02–3.94 (m, 1H), 3.92–3.84 (m, 1H), 2.70 (s, 2H), 2.30 (t, *J* = 7.3 Hz, 2H), 1.86 (s, 3H), 1.68–1.54 (m, 4H), 1.26 (m, 8H); ^13^C NMR (100 MHz, CDCl_3_) *δ* 177.7, 170.6, 166.8, 165.0, 134.3, 133.6, 133.4, 132.5, 129.8, 129.7, 129.4, 129.2, 128.6, 128.5, 128.40, 128.37, 97.5, 71.1, 68.7, 68.4, 68.3, 66.6, 51.8, 49.6, 33.9, 29.2, 29.0, 28.91, 28.86, 25.5, 24.7, 22.9. HRMS m/z calcd for C_41_H_47_BrN_4_O_12_SNa [M + Na]^+^ 921.1990, found 921.1994.

*Synthesis of compound **8b**.* Compound **7b** (90.0 mg, 0.17 mmol), *t*-BuOH: THF: H_2_O (v:v:v 1:1:1, 2.5 mL), 10-undecynoic acid (31.7 mg, 0.17 mmol), CuSO_4_·5H_2_O (6.7 mg, 0.02 mmol), NaAsc (10.6 mg, 0.05 mmol), 5 h. The crude was purified by flash chromatography (DCM to 3% MeOH/DCM) to obtain a colorless oil (68 mg, 56%) as the desired product. (R_f_ = 0.24 in 5% MeOH/DCM). ^1^H NMR (400 MHz, CDCl_3_) *δ* 7.94–7.86 (m, 4H), 7.54–7.46 (m, 2H), 7.44 (s, 1H), 7.40–7.32 (m, 4H), 6.13 (d, *J* = 9.2 Hz, 1H), 5.58 (dd, *J* = 10.7, 9.6 Hz, 1H), 5.41 (t, *J* = 9.6 Hz, 1H), 4.96 (d, *J* = 3.6 Hz, 1H), 4.70–4.59 (m, 2H), 4.59–4.51 (m, 1H), 4.31–4.20 (m, 1H), 4.03–3.89 (m, 2H), 3.68–3.57 (m, 2H), 2.73 (t, *J* = 7.6 Hz, 2H), 2.33 (t, *J* = 7.3 Hz, 2H), 1.88 (s, 3H), 1.69–1.58 (m, 4H), 1.29 (m, 8H); ^13^C NMR (100 MHz, CDCl_3_) *δ* 170.6, 167.0, 165.2, 133.6, 133.5, 129.9, 129.8, 128.73, 128.65, 128.5, 128.4, 97.5, 71.4, 70.5, 70.3, 66.6, 52.0, 49.5, 43.6, 29.2, 28.9, 28.8, 28.7, 25.6, 23.0. HRMS m/z calcd for C_35_H_43_ClN_4_O_9_Na [M + Na]^+^ 721.2611, found 721.2609.

*Synthesis of compound **8c**.* Compound **7c** (360.0 mg, 0.55 mmol), *t*-BuOH: THF: H_2_O (v:v:v 1:1:1, 6.0 mL), 10-undecynoic acid (138.0 mg, 0.72 mmol), CuSO_4_·5H_2_O (18.0 mg, 0.108 mmol, 0.2 equiv), NaAsc (44.0 mg, 0.22 mmol), 12 h. Purified by flash chromatography (DCM to 5% MeOH/DCM) to afford the colorless a semi-solid (378.0 mg, 82%) as the desired product (R_f_ = 0.65 in 10% MeOH/DCM). ^1^H NMR (400 MHz, CDCl_3_) *δ* 7.89–7.81 (m, 4H), 7.70 (d, *J* = 8.3 Hz, 2H), 7.55–7.43 (m, 3H), 7.40–7.30 (m, 4H), 7.21 (d, *J* = 8.1 Hz, 2H), 6.07 (d, *J* = 9.2 Hz, 1H), 5.54 (dd, *J* = 10.8, 9.6 Hz, 1H), 5.30 (t, *J* = 9.6 Hz, 1H), 4.86 (d, *J* = 3.6 Hz, 1H), 4.65–4.56 (m, 2H), 4.51–4.43 (m, 1H), 4.22–4.04 (m, 4H), 3.93–3.85 (m, 1H), 2.72 (t, *J* = 7.7 Hz, 2H), 2.36 (s, 3H), 2.32 (t, *J* = 7.4 Hz, 2H), 1.86 (s, 3H), 1.71–1.57 (m, 4H), 1.29 (m, 8H); ^13^C NMR (100 MHz, CDCl_3_) *δ* 177.0, 170.5, 166.9, 165.1, 148.8, 145.0, 133.6, 133.4, 132.3, 130.0, 129.9, 129.81, 129.78, 128.7, 128.6, 128.4, 128.0, 121.3, 97.5, 71.3, 68.9, 68.7, 68.2, 66.7, 51.9, 49.5, 33.8, 29.3, 29.0, 28.84, 28.82, 25.6, 24.7, 23.0, 21.6; HRMS m/z calcd for C_42_H_50_N_4_O_21_SNa [M + Na]^+^ 857.3038, found 857.3039.

*Synthesis of compound **LM28 (13c)**.* Compound **8c** (100.0 mg, 0.12 mmol, 1.0 equiv), K_2_CO_3_ (33.0 mg, 0.24 mmol, 2.0 equiv) and DMF (14 mL) were added to a 50 mL RBF. The reaction mixture was stirred at 70 °C for 3 h, at which time ^1^H NMR spectrum and TLC indicated the full conversion of starting materials. The reaction was stopped and solvent was removed. The crude was purified via flash chromatography using an eluent of DCM to 5% MeOH/DCM to obtain the desired product as a white solid (65.0 mg, 82%), R_f_ = 0.29 in 5% MeOH/DCM), m.p. 196.0 °C-198.0 °C; ^1^H NMR (400 MHz, CDCl_3_) *δ* ppm 7.95–7.86 (m, 4H), 7.54–7.45 (m, 2H), 7.42 (s, 1H), 7.40–7.31 (m, 4H), 6.00 (d, *J* = 9.2 Hz, 1H), 5.62 (dd, *J* = 10.9, 9.7 Hz, 1H), 5.45 (t, *J* = 9.7 Hz, 1H), 4.77–4.69 (m, 1H), 4.66 (d, *J* = 3.5 Hz, 1H), 4.62–4.54 (m, 1H), 4.51–4.43 (m, 1H), 4.41–4.34 (m, 1H), 4.13–4.01 (m, 4H), 2.87–2.78 (m, 1H), 2.77–2.67 (m, 1H), 2.31–2.20 (m, 1H), 2.19–2.09 (m, 1H), 1.93 (s, 3H), 1.79–1.60 (m, 2H), 1.56–1.43 (m, 2H), 1.29–1.09 (m, 8H); ^13^C NMR (100 MHz, CDCl_3_) *δ* 173.4, 170.2, 167.2, 165.4, 133.52, 133.47, 129.9, 129.8, 128.9, 128.8, 128.5, 128.4, 121.3, 97.7, 71.4, 70.2, 68.0, 65.0, 63.2, 52.6, 46.6, 33.7, 30.2, 28.20, 28.19, 28.1, 27.7, 27.2, 24.9, 24.4, 23.1; HRMS (ESI+) ([M + Na]^+^) m/z calcd for C_35_H_42_N_4_O_9_Na, 685.2844, found 685.2825.

*Synthesis of compounds **LM28** and **DLM28**.* Compound **8a** (100.0 mg, 0.11 mmol, 1.0 equiv), DMF (7.0 mL), and K_2_CO_3_ (30.7 mg, 0.22 mmol, 2.0 equiv) were added to a 50 mL RBF. The reaction mixture was stirred at 75 °C for 5 h, the ^1^H NMR and TLC samples showed complete conversion of the starting materials. The crude was purified by flash chromatography using DCM to 5% MeOH DCM to obtain the desired compound **LM28** (53.0 mg, 0.080 mmol, 72%) along with some later fraction which was identified as the dimerization product **DLM28** as a white solid (15.0 mg, 0.011 mmol, 20% based on starting material conversion). The chloro compound **8b** was cyclized by similar conditions, using compound **8b** (50.0 mg, 0.07 mmol, 1.0 equiv), DMF (7.0 mL), and K_2_CO_3_ (19.8 mg, 0.14 mmol, 2.0 equiv). The desired compound **LM28** (31.7 mg, 0.048 mmol, 67%) and **DLM28** (8.0 mg, 0.006 mmol, 17% based on starting material conversion) were obtained. Characterization for **DLM28**: R_f_ = 0.18 in 5% MeOH/DCM. m.p. 236.0–238.0 °C; ^1^H NMR (400 MHz, CDCl_3_) *δ* 7.92–7.87 (m, 8H), 7.51–7.46 (m, 6H), 7.37–7.31 (m, 8H), 6.06 (d, *J* = 9.1 Hz, 2H), 5.58 (dd, *J* = 10.3, 9.5 Hz, 2H), 5.43 (t, *J* = 9.8 Hz, 2H), 4.89 (d, *J* = 3.5 Hz, 2H), 4.63–4.50 (m, 6H), 4.21–4.11 (m, 6H), 3.95–3.88 (m, 4H), 2.72 (t, *J* = 7.6 Hz, 4H), 2.27–2.22 (m, 4H), 1.89 (s, 6H), 1.70–1.66 (m, 4H), 1.59–1.54 (m, 4H), 1.33–1.23 (m, 16H); ^13^C NMR (100 MHz, CDCl_3_) *δ* 173.2, 170.3, 166.9, 165.2, 133.5, 133.4, 129.83, 129.77, 128.87, 128.85, 128.4, 121.3, 97.7, 71.4, 69.3, 68.6, 66.8, 62.6, 52.0, 49.5, 34.0, 29.2, 28.93, 28.88, 28.8, 25.6, 24.7, 23.1. HRMS m/z calcd for C_70_H_84_N_8_O_18_Na [M + Na]^+^ 1347.5796, found 1347.5797.

*Synthesis of compound **10d***. 3-azido propionic acid (200.0 mg, 1.74 mmol, 1.0 equiv) and 1,7-octadiyne (276.0 mg, 2.6 mmol, 1.5 equiv) were dissolved in *t*-BuOH: THF: H_2_O (v:v:v 1:1:1, 25.0 mL), then CuSO_4_·5H_2_O (84.8 mg, 0.34 mmol, 0.2 equiv), NaAsc (134.7 mg, 0.68 mmol, 0.4 equiv) were added to the reaction mixture. The reaction was stirred at rt for 24 h, at which time the reaction was completed as indicated by ^1^H NMR and TLC. The reaction was stopped, and solvent was removed using a rotovap, the residue was diluted with EtOAc and acidified using 0.1 N HCl (5.0 mL) followed by water wash. The organic layer was collected and dried over anhydrous Na_2_SO_4_ and solvent was removed under vacuum to obtain the crude, which was further purified with flash chromatography using eluent of hexanes to 60% EtOAc/Hexanes to obtain the desired product as a yellowish solid (272.0 mg, 71%), R_f_ = 0.48 in 80% EtOAc/Hexanes, m.p. 93.5–94.5 °C; ^1^H NMR (400 MHz, CDCl_3_) *δ* 8.98 (br s, 1H), 7.47 (s, 1H), 4.66 (t, *J* = 6.5 Hz, 2H), 3.02 (t, *J* = 6.5 Hz, 2H), 2.75 (t, *J* = 7.6 Hz, 2H), 2.23–2.18 (m, 2H), 1.92 (t, *J* = 2.6 Hz, 1H), 1.82–1.73 (m, 2H), 1.62–1.54 (m, 2H); ^13^C NMR (100 MHz, CDCl_3_) *δ* 173.2, 147.7, 121.8, 84.2, 68.5, 45.7, 34.7, 28.3, 27.9, 24.9, 18.1. HRMS m/z calcd for C_11_H_15_N_3_O_2_Na [M + Na]^+^ 244.1056, found 244.1056.

*Synthesis of compound **10e***. The same procedure for compound **10d** was used, 3-azido propionic acid (200.0 mg, 1.74 mmol), *t*-BuOH: THF: H_2_O (v:v:v 1:1:1, 25.0 mL), 1,8-nonadiyne (313.3 mg, 2.6 mmol), CuSO_4_·5H_2_O (84.8 mg, 0.34 mmol), NaAsc (134.7 mg, 0.68 mmol), 24 h. The product was purified using flash chromatography (Hexanes to 30% EtOAc/Hexanes) to obtain a yellowish solid (306.0 mg, 75%) as the desired product (R_f_ = 0.5 in 80% EtOAc/Hexanes). m.p. 137.5–139.0 °C; ^1^H NMR (400 MHz, CDCl_3_) 7.39 (s, 1H), 4.62 (t, *J* = 6.5 Hz, 2H), 3.02 (t, *J* = 6.5 Hz, 2H), 2.71 (t, *J* = 7.7 Hz, 2H), 2.23–2.13 (m, 2H), 1.93 (t, *J* = 2.7 Hz, 1H), 1.72–1.64 (m, 2H), 1.59–1.51 (m, 2H), 1.50–1.43 (m, 2H); ^13^C NMR (100 MHz, CDCl_3_) *δ* 173.0, 148.0, 121.6, 84.5, 68.3, 45.5, 34.6, 28.8, 28.3, 28.2, 25.4, 18.3. HRMS (ESI+) m/z calcd for C_12_H_17_N_3_O_2_Na [M + Na]^+^ 258.1213, found 258.1212.

*Synthesis of compound **11a**.* Compound **7c** (100.0 mg, 0.15 mmol), *t*-BuOH: THF: H_2_O (v:v:v 1:1:1, 3.0 mL), 6-heptynoic acid (25.2 mg, 0.2 mmol), CuSO_4_·5H_2_O (7.7 mg, 0.03 mmol), NaAsc (12.1 mg, 0.06 mmol), 16 h. Purified by flash chromatography (DCM to 3% MeOH/DCM) to obtain a colorless slurry (96.0 mg, 81%) as the desired product (R_f_ = 0.32 in 5% MeOH/DCM). ^1^H NMR (400 MHz, CDCl_3_) *δ* 7.88–7.83 (m, 4H), 7.70 (d, *J* = 8.2 Hz, 2H), 7.67 (s, 1H), 7.55–7.47 (m, 3H), 7.40–7.32 (m, 5H), 7.22 (d, *J* = 8.0 Hz, 2H), 6.45 (d, *J* = 8.8 Hz, 1H), 5.62 (t, *J* = 10.1 Hz, 1H), 5.31 (t, *J* = 9.4 Hz, 1H), 4.90 (d, *J* = 3.6 Hz, 1H), 4.70–4.59 (m, 2H), 4.57–4.49 (m, 1H), 4.22–4.07 (m, 4H), 3.97–3.90 (m, 1H), 2.85–2.77 (m, 2H), 2.43–2.34 (m, 5H), 1.85 (s, 3H), 1.81–1.68 (m, 4H); ^13^C NMR (100 MHz, CDCl_3_) *δ* 176.5, 170.6, 167.5, 165.1, 145.1, 133.8, 133.6, 132.3, 129.9, 129.8, 128.53, 128.46, 128.0, 97.2, 71.6, 69.1, 68.5, 68.3, 66.4, 51.8, 32.8, 27.8, 24.9, 23.5, 22.9, 21.6. HRMS m/z calcd for C_38_H_42_N_4_O_12_SNa [M + Na]^+^ 801.2412, found 801.2406.

*Synthesis of compound **11b**.* Compound **7c** (100.0 mg, 0.15 mmol), *t*-BuOH: THF: H_2_O (v:v:v 1:1:1, 3.0 mL), 8-nonynoic acid (30.7 mg, 0.2 mmol), CuSO_4_·5H_2_O (7.7 mg, 0.03 mmol), NaAsc (12.1 mg, 0.06 mmol), 16 h. Purified by flash chromatography (DCM to 3% MeOH/DCM) to obtain a colorless slurry (109.0 mg, 88%) as the desired product (R_f_ = 0.38 in 5% MeOH/DCM). ^1^H NMR (400 MHz, CDCl_3_) *δ* 7.88–7.82 (m, 4H), 7.70 (d, *J* = 8.0 Hz, 2H), 7.54–7.45 (m, 3H), 7.38–7.30 (m, 4H), 7.21 (d, *J* = 7.9 Hz, 2H), 6.15 (d, *J* = 6.9 Hz, 1H), 5.55 (t, *J* = 10.1 Hz, 1H), 5.29 (t, *J* = 8.6 Hz, 1H), 4.86 (d, *J* = 2.4 Hz, 1H), 4.60 (br s, 2H), 4.52–4.43 (m, 1H), 4.20–4.05 (m, 4H), 3.90–3.84 (m, 1H), 2.73 (br s, 2H), 2.35 (s, 3H), 2.31 (t, *J* = 7.2 Hz, 2H), 1.86 (s, 3H), 1.72–1.58 (m, 4H), 1.38–1.33 (m, 4H); ^13^C NMR (100 MHz, CDCl_3_) *δ* 176.6, 169.6, 165.9, 164.1, 144.1, 132.6, 132.5, 131.3, 128.9, 128.8, 127.7, 127.6, 127.4, 127.0, 96.5, 70.3, 67.9, 67.6, 67.3, 65.5, 50.8, 32.7, 27.47, 27.45, 23.5, 20.6. HRMS m/z calcd for C_40_H_46_N_4_O_12_SNa [M + Na]^+^ 829.2725, found 829.2722.

*Synthesis of compound **12a**.* Compound **9** (100.0 mg, 0.15 mmol), *t*-BuOH: THF: H_2_O (v:v:v 1:1:1, 2.5 mL), 6-heptynoic acid (24.6 mg, 0.19 mmol), CuSO_4_·5H_2_O (7.5 mg, 0.03 mmol), NaAsc (11.8 mg, 0.06 mmol), 24 h. Purified by flash chromatography (DCM to 2% MeOH/DCM) to obtain a colorless slurry (98.0 mg, 82%) as the desired product (R_f_ = 0.22 in 5% MeOH/DCM). ^1^H NMR (400 MHz, CDCl_3_) *δ* 7.92–7.83 (m, 4H), 7.69 (d, *J* = 8.3 Hz, 2H), 7.56–7.46 (m, 3H), 7.40–7.32 (m, 4H), 7.21 (d, *J* = 8.2 Hz, 2H), 6.43 (d, *J* = 9.2 Hz, 1H), 5.61 (t, *J* = 10.1 Hz, 1H), 5.32 (t, *J* = 10.0 Hz, 1H), 4.88 (d, *J* = 3.6 Hz, 1H), 4.66–4.57 (m, 1H), 4.54–4.42 (m, 2H), 4.31–4.23 (m, 1H), 4.21–4.08 (m, 2H), 3.82–3.74 (m, 1H), 3.43–3.33 (m, 1H), 2.79 (t, *J* = 6.7 Hz, 2H), 2.42–2.33 (m, 5H), 2.29–2.19 (m, 2H), 1.93 (s, 3H), 1.81–1.64 (m, 4H); ^13^C NMR (100 MHz, CDCl_3_) *δ* 176.4, 170.9, 167.1, 165.2, 148.1, 145.0, 133.6, 132.3, 129.9, 129.8, 128.7, 128.6, 128.5, 128.4, 128.0, 121.3, 97.1, 71.5, 69.1, 68.5, 68.4, 64.5, 52.1, 46.8, 33.1, 29.6, 28.2, 25.0, 23.8, 23.0, 21.6. HRMS m/z calcd for C_39_H_44_N_4_O_12_SNa [M + Na]^+^ 815.2569, found 815.2564.

*Synthesis of compound **12b**.* Compound **9** (100.0 mg, 0.15 mmol), *t*-BuOH: THF: H_2_O (v:v:v 1:1:1, 2.5 mL), 8-nonynoic acid (30.1 mg, 0.19 mmol), CuSO_4_·5H_2_O (7.5 mg, 0.03 mmol), NaAsc (11.8 mg, 0.06 mmol), 24 h. Purified by flash chromatography (DCM to 2% MeOH/DCM) to obtain a colorless slurry (106.0 mg, 86%) as the desired product (R_f_ = 0.23 in 5% MeOH/DCM). ^1^H NMR (400 MHz, CDCl_3_) *δ* 7.92–7.84 (m, 4H), 7.69 (d, *J* = 8.2 Hz, 2H), 7.56–7.46 (m, 2H), 7.41–7.31 (m, 5H), 7.21 (d, *J* = 8.2 Hz, 2H), 6.38 (d, *J* = 9.2 Hz, 1H), 5.60 (t, *J* = 10.0 Hz, 1H), 5.31 (t, *J* = 9.9 Hz, 1H), 4.84 (d, *J* = 3.5 Hz, 1H), 4.67–4.57 (m, 1H), 4.55–4.43 (m, 2H), 4.31–4.24 (m, 1H), 4.20–4.07 (m, 2H), 3.87–3.78 (m, 1H), 3.40–3.31 (m, 1H), 2.74 (t, *J* = 7.5 Hz, 2H), 2.38–2.18 (m, 7H), 1.95 (s, 3H), 1.74–1.59 (m, 4H), 1.42–1.32 (m, 4H); ^13^C NMR (100 MHz, CDCl_3_) *δ* 176.9, 170.8, 167.0, 165.2, 148.5, 145.0, 133.54, 133.48, 132.3, 129.9, 129.8, 128.8, 128.7, 128.4, 128.0, 120.9, 97.5, 71.5, 69.2, 68.6, 68.5, 64.8, 52.0, 46.9, 33.5, 29.9, 28.9, 28.44, 28.37, 25.2, 24.5, 23.0, 21.6. HRMS m/z calcd for C_41_H_48_N_4_O_12_SNa [M + Na]^+^ 843.2882, found 843.2876.

*Synthesis of compound**12c***. Compound **9** (100.0 mg, 0.15 mmol), *t*-BuOH: THF: H_2_O (v:v:v 1:1:1, 2.5 mL), 10-undecynoic acid (35.5 mg, 0.19 mmol), CuSO_4_·5H_2_O (7.5 mg, 0.03 mmol), NaAsc (11.8 mg, 0.06 mmol), 24 h. Purified by flash chromatography using (DCM to 2% MeOH/DCM) to obtain a colorless slurry (98.0 mg, 77%) as the desired product (R_f_ = 0.27 in 5% MeOH/DCM). ^1^H NMR (400 MHz, CDCl_3_) *δ* 7.91–7.84 (m, 4H), 7.69 (d, *J* = 8.2 Hz, 2H), 7.55–7.44 (m, 2H), 7.41–7.30 (m, 5H), 7.20 (d, *J* = 8.2 Hz, 2H), 6.44 (d, *J* = 9.2 Hz, 1H), 5.59 (t, *J* = 10.1 Hz, 1H), 5.31 (t, *J* = 9.8 Hz, 1H), 4.83 (d, *J* = 3.5 Hz, 1H), 4.70–4.57 (m, 1H), 4.56–4.43 (m, 2H), 4.32–4.23 (m, 1H), 4.20–4.06 (m, 2H), 3.89–3.80 (m, 1H), 3.40–3.28 (m, 1H), 2.72 (t, *J* = 7.6 Hz, 2H), 2.37–2.17 (m, 7H), 1.94 (s, 3H), 1.71–1.55 (m, 4H), 1.39–1.24 (m, 8H); ^13^C NMR (100 MHz, CDCl_3_) *δ* 177.8, 170.8, 166.9, 165.2, 148.9, 145.0, 133.5, 133.4, 132.3, 129.84, 129.79, 129.77, 128.8, 128.6, 128.4, 128.1, 128.0, 120.7, 97.5, 71.4, 69.2, 68.5, 68.4, 64.8, 51.9, 46.8, 33.9, 29.9, 29.2, 28.92, 28.89, 28.85, 25.5, 24.6, 23.0, 21.6; HRMS m/z calcd for C_43_H_52_N_4_O_12_SNa [M + Na]^+^ 871.3195, found 871.3192.

*Synthesis of compound **13a (LM24)**.* Compound **11a** (74.0 mg, 0.095 mmol, 1.0 equiv), DMF (13.0 mL), K_2_CO_3_ (26.3 mg, 0.19 mmol, 2.0 equiv, 75 °C for 2 h. Purified by flash chromatography using (DCM to 3% MeOH/DCM) to obtain a white solid (51.0 mg, 88%) as the desired product (R_f_ = 0.32 in 5% MeOH/DCM). m.p. 123.0–125.0 °C; ^1^H NMR (400 MHz, CDCl_3_) *δ* 7.98–7.86 (m, 4H), 7.54–7.46 (m, 3H), 7.41–7.32 (m, 4H), 6.18 (d, *J* = 8.6 Hz, 1H), 5.62 (dd, *J* = 10.9, 9.6 Hz, 1H), 5.36 (t, *J* = 9.9 Hz, 1H), 5.02 (d, *J* = 3.6 Hz, 1H), 4.87–4.77 (m, 1H), 4.64–4.55 (m, 1H), 4.50–4.42 (m, 1H), 4.22 (dd, *J* = 11.9, 4.4 Hz, 1H), 4.13–3.94 (m, 3H), 3.63–3.55 (m, 1H), 2.96–2.86 (m, 1H), 2.72–2.61 (m, 1H), 2.30–2.21 (m, 1H), 2.01–1.93 (m, 1H), 1.91 (s, 3H), 1.63–1.48 (m, 4H); ^13^C NMR (100 MHz, CDCl_3_) *δ* 172.3, 170.2, 167.2, 165.5, 148.6, 133.6, 133.5, 129.91, 128.87, 128.8, 128.7, 128.5, 128.4, 120.6, 97.2, 71.0, 70.4, 69.1, 68.6, 64.5, 52.8, 50.0, 33.7, 28.2, 24.7, 23.2, 23.1; HRMS m/z calcd for C_31_H_34_N_4_O_9_Na [M + Na]^+^ 629.2218, found 629.2222.

*Synthesis of compound* ***13b (LM26)***. Compound **11b** (100.0 mg, 0.12 mmol), DMF (10.0 mL), K_2_CO_3_ (34.3 mg, 0.24 mmol), 75 °C for 5 h. Purified by flash chromatography (DCM to 5% MeOH/DCM) to obtain a white solid (60.0 mg, 76%) as the desired product (R_f_ = 0.29 in 5% MeOH/DCM). m.p. 108.0–110.0 °C; ^1^H NMR (400 MHz, CDCl_3_) *δ* 7.97–7.87 (m, 4H), 7.54–7.45 (m, 3H), 7.41–7.31 (m, 4H), 6.21 (d, *J* = 9.1 Hz, 1H, -N*H*), 5.62 (dd, *J* = 10.9, 9.5 Hz, 1H, H-3), 5.37 (t, *J* = 9.8 Hz, 1H, H-4), 4.99 (d, *J* = 3.6 Hz, 1H, H–1), 4.72–4.67 (m, 2H, -O-CH_2_-C*H*_2_-N), 4.59–4.50 (m, 1H, H-2), 4.22–4.02 (m, 3H, H-6_a_, H-6_b,_ -O-C*H*_a_-CH_2_-N), 4.00–3.93 (m, 1H, -O-C*H*_b_-CH_2_-N), 3.87–3.78 (m, 1H, H-5), 2.90–2.71 (m, 2H, -HC=C-C*H*_2_-CH_2_-), 2.16–1.98 (m, 2H, -OOC-C*H*_2_-CH_2_-), 1.90 (s, 3H), 1.80–1.68 (m, 1H), 1.67–1.55 (m, 1H), 1.54–1.40 (m, 2H), 1.28–1.07 (m, 4H); ^13^C NMR (100 MHz, CDCl_3_) *δ* 173.3, 170.1, 167.0, 165.3, 148.5 (HC=*C*-), 133.5, 129.83, 128.79, 128.7, 128.44, 128.42, 120.8 (H*C*=C-), 97.3 (C-1), 71.1 (C-3), 70.4 (C-4), 68.5 (C-5), 67.1 (-O-*C*H_2_-CH_2_-N), 64.1 (C-6), 52.3 (C-2), 49.6 (-O-CH_2_-*C*H_2_-N), 32.9, 27.9, 27.4, 25.9, 24.7, 23.7, 23.1; HRMS m/z calcd for C_33_H_38_N_4_O_9_Na [M + Na]^+^ 657.2531, found 657.2529.

*Synthesis of compound **14a (LM34)**.* Compound **12a** (96.0 mg, 0.12 mmol), DMF (10.0 mL), K_2_CO_3_ (33.5 mg, 0.24 mmol), 75 °C for 2 h. Purified by flash chromatography (DCM to 3% MeOH/DCM) to obtain a colorless liquid which turns into a white solid over time (66.3 mg, 88%) as the desired product (R_f_ = 0.2 in 5% MeOH/DCM). m.p. 117.0–118.0 °C; ^1^H NMR (400 MHz, CDCl_3_) *δ* 7.97–7.85 (m, 4H), 7.54–7.45 (m, 2H), 7.42–7.31 (m, 5H), 5.88 (d, *J* = 9.2 Hz, 1H), 5.65 (t, *J* = 10.2 Hz, 1H), 5.31 (t, *J* = 9.6 Hz, 1H), 4.93 (d, *J* = 3.6 Hz, 1H), 4.75–4.66 (m, 1H), 4.57–4.42 (m, 2H), 4.19–4.01 (m, 3H), 3.55–3.37 (m, 2H), 2.90–2.70 (m, 2H), 2.43–2.16 (m, 4H), 1.87 (s, 3H), 1.79–1.72 (m, 2H), 1.69–1.62 (m, 2H), 1.53–1.45 (m, 2H); ^13^C NMR (100 MHz, CDCl_3_) *δ* 172.4, 169.9, 167.2, 165.3, 147.3, 133.5, 129.9, 129.8, 128.7, 128.5, 126.9, 121.2, 97.4, 71.4, 69.5, 68.6, 64.8, 64.0, 52.3, 46.6, 41.0, 34.3, 29.8, 27.3, 24.4, 23.3, 23.2; HRMS m/z calcd for C_32_H_36_N_4_O_9_Na [M + Na]^+^ 643.2375, found 643.2374.

*Synthesis of compound **14b (LM36)**.* Compound **12b** (86.0 mg, 0.10 mmol), DMF (10.0 mL), K_2_CO_3_ (28.9 mg, 0.20 mmol), 75 °C for 2 h. Purified by flash chromatography (DCM to 3% MeOH/DCM) to obtain a white solid (56.6 mg, 83%) as the desired product (R_f_ = 0.3 in 5% MeOH/DCM). m.p. 97.0–98.0 °C; ^1^H NMR (400 MHz, CDCl_3_) *δ* 7.98–7.86 (m, 4H), 7.53–7.46 (m, 2H), 7.40–7.31 (m, 5H), 5.95 (d, *J* = 9.1 Hz, 1H), 5.67 (t, *J* = 10.2 Hz, 1H), 5.35 (t, *J* = 9.7 Hz, 1H), 4.98 (d, *J* = 3.6 Hz, 1H), 4.69–4.60 (m, 1H), 4.55–4.48 (m, 1H), 4.47–4.39 (m, 1H), 4.24–4.09 (m, 3H), 3.66–3.51 (m, 2H), 2.86–2.68 (m, 2H), 2.43–2.24 (m, 2H), 2.08 (t, *J* = 7.0 Hz, 2H), 1.88 (s, 3H), 1.74–1.64 (m, 2H), 1.61–1.47 (m, 2H), 1.33–1.16 (m, 4H); ^13^C NMR (100 MHz, CDCl_3_) *δ* 173.2, 169.9, 167.2, 165.4, 148.1, 133.53, 133.51, 130.0, 129.84, 128.79, 128.7, 128.46, 128.45, 120.9, 98.0, 71.4, 70.0, 68.5, 65.5, 63.8, 52.6, 46.6, 32.4, 30.3, 27.4, 26.6, 25.7, 24.6, 23.5, 23.2; HRMS m/z calcd for C_34_H_40_N_4_O_9_Na [M + Na]^+^ 671.2688, found 671.2686.

*Synthesis of compound **14c (LM38)**.* Compound **12c** (90.0 mg, 0.11 mmol), DMF (10.0 mL), K_2_CO_3_ (30.4 mg, 0.20 mmol), 75 °C for 2 h. Purified by flash chromatography (DCM to 2% MeOH/DCM) to obtain a white solid (59.5 mg, 83%) as the desired product (R_f_ = 0.32 in 5% MeOH/DCM). m.p. 87.0–89.0 °C; ^1^H NMR (400 MHz, CDCl_3_) *δ* 7.97–7.88 (m, 4H), 7.54–7.47 (m, 2H), 7.41–7.31 (m, 5H), 6.12 (d, *J* = 8.8 Hz, 1H), 5.62 (t, *J* = 10.6 Hz, 1H), 5.44 (t, *J* = 9.8 Hz, 1H), 4.91 (d, *J* = 3.6 Hz, 1H), 4.61–4.42 (m, 3H), 4.33–4.12 (m, 3H), 3.71–3.61 (m, 1H), 3.46–3.36 (m, 1H), 2.88–2.71 (m, 2H), 2.33–2.04 (m, 4H), 1.93 (s, 3H), 1.75–1.68 (m, 4H), 1.56–1.47 (m, 2H), 1.28–1.15 (m, 8H); ^13^C NMR (100 MHz, CDCl_3_) *δ* 173.4, 170.2, 167.2, 165.4, 133.52, 133.47, 129.9, 129.8, 128.9, 128.8, 128.5, 128.4, 121.3, 97.7, 71.4, 70.2, 68.0, 65.0, 63.2, 52.6, 46.6, 33.7, 30.2, 28.20, 28.19, 28.14, 27.7, 27.2, 24.9, 24.4, 23.1; HRMS m/z calcd for C_36_H_44_N_4_O_9_Na [M + Na]^+^ 699.3001, found 699.3000.

Compounds **15a-b and 16a-b** were prepared similarly as compound **8a**.

*Synthesis of compound **15a**.* Compound **7c** (200.0 mg, 0.31 mmol, 1.0 equiv), *t*-BuOH: THF: H_2_O (v:v:v 1:1:1, 3.0 mL), **10d** (89.2 mg, 0.4 mmol, 1.3 equiv), CuSO_4_·5H_2_O (15.5 mg, 0.062 mmol, 0.2 equiv), NaAsc (24.6 mg, 0.124 mmol, 0.4 equiv), 24 h. Purified by flash chromatography (DCM to 3% MeOH/DCM) to obtain a yellowish slurry (209.0 mg, 78%) as the desired product (R_f_ = 0.5 in 5% MeOH/DCM). ^1^H NMR (400 MHz, CDCl_3_) *δ* 7.86–7.80 (m, 4H), 7.68 (d, *J* = 8.3 Hz, 2H), 7.54–7.46 (m, 2H), 7.45 (s, 1H), 7.41 (s, 1H), 7.38–7.29 (m, 4H), 7.20 (d, *J* = 8.0 Hz, 2H), 6.26 (d, *J* = 9.1 Hz, 1H), 5.54 (t, *J* = 10.8 Hz, 1H), 5.28 (t, *J* = 9.6 Hz, 1H), 4.84 (d, *J* = 3.6 Hz, 1H), 4.63–4.55 (m, 4H), 4.49–4.42 (m, 1H), 4.20–4.04 (m, 4H), 3.89–3.83 (m, 1H), 2.91 (t, *J* = 6.2 Hz, 2H), 2.76–2.66 (m, 4H), 2.34 (s, 3H), 1.82 (s, 3H), 1.75–1.62 (m, 4H); ^13^C NMR (100 MHz, CDCl_3_) *δ* 172.6, 171.0, 166.7, 165.1, 148.2, 147.3, 145.1, 133.6, 133.4, 132.3, 129.9, 129.81, 129.75, 128.7, 128.6, 128.4, 128.0, 122.2, 121.6, 97.5, 71.2, 68.9, 68.6, 68.2, 66.7, 51.9, 49.6, 45.9, 35.1, 28.5, 28.3, 25.2, 25.0, 22.8, 21.6. HRMS m/z calcd for C_42_H_47_N_7_O_12_SNa [M + Na]^+^ 896.2896, found 896.2886.

*Synthesis of compound **15b**.* Compound **7c** (100.0 mg, 0.15 mmol, 1.0 equiv), *t*-BuOH: THF: H_2_O (v:v:v 1:1:1, 3.0 mL), **10e** (46.8 mg, 0.19 mmol, 1.3 equiv), CuSO_4_·5H_2_O (7.3 mg, 0.03 mmol, 0.2 equiv), NaAsc (12.3 mg, 0.06 mmol, 0.4 equiv), 24 h. Purified by flash chromatography (DCM to 2% MeOH/DCM) to obtain a yellowish slurry (108.0 mg, 79%) as the desired product (R_f_ = 0.41 in 5% MeOH/DCM). ^1^H NMR (400 MHz, CDCl_3_) *δ* 7.88–7.81 (m, 4H), 7.69 (d, *J* = 8.3 Hz, 2H), 7.54–7.47 (m, 2H), 7.45 (s, 1H), 7.40 (s, 1H), 7.38–7.30 (m, 4H), 7.21 (d, *J* = 7.9 Hz, 2H), 6.11 (d, *J* = 9.1 Hz, 1H), 5.53 (t, *J* = 10.8 Hz, 1H), 5.28 (t, *J* = 9.6 Hz, 1H), 4.86 (d, *J* = 3.6 Hz, 1H), 4.64–4.58 (m, 4H), 4.50–4.42 (m, 1H), 4.21–4.07 (m, 4H), 3.93–3.86 (m, 1H), 2.91 (t, *J* = 6.2 Hz, 2H), 2.73–2.65 (m, 4H), 2.35 (s, 3H), 1.83 (s, 3H), 1.70–1.59 (m, 4H), 1.36–1.28 (m, 2H); ^13^C NMR (100 MHz, CDCl_3_) *δ* 172.5, 170.8, 166.8, 165.1, 148.4, 147.5, 145.1, 133.6, 133.5, 132.3, 129.83, 129.77, 128.7, 128.6, 128.4, 128.0, 122.2, 121.6, 97.5, 71.2, 68.9, 68.7, 68.2, 66.6, 51.9, 49.6, 45.8, 35.1, 29.0, 28.4, 28.0, 25.3, 25.0, 22.9, 21.6. HRMS m/z calcd for C_43_H_49_N_7_O_12_SNa [M + Na]^+^ 910.3052, found 910.3043.

*Synthesis of compound **15c**.* To a 50 mL RBF, compound **9** (100.0 mg, 0.15 mmol, 1.0 equiv), *t*-BuOH: THF: H_2_O (v:v:v 1:1:1, 3.0 mL), **10d** (43.1 mg, 0.19 mmol, 1.3 equiv), CuSO_4_·5H_2_O (7.3 mg, 0.03 mmol, 0.2 equiv), NaAsc (12.3 mg, 0.06 mmol, 0.4 equiv), 16 h. Purified by flash chromatography (DCM to 5% MeOH/DCM) to obtain a yellowish slurry (116.0 mg, 87%) as the desired product (R_f_ = 0.41 in 5% MeOH/DCM). ^1^H NMR (400 MHz, CDCl_3_) *δ* 7.91–7.85 (m, 4H), 7.68 (d, *J* = 8.3 Hz, 2H), 7.56–7.46 (m, 2H), 7.43 (s, 1H), 7.40–7.31 (m, 5H), 7.20 (d, *J* = 8.1 Hz, 2H), 6.39 (d, *J* = 9.1 Hz, 1H), 5.59 (t, *J* = 9.7 Hz, 1H), 5.31 (t, *J* = 9.7 Hz, 1H), 4.84 (d, *J* = 3.5 Hz, 1H), 4.68–4.56 (m, 3H), 4.54–4.42 (m, 2H), 4.33–4.24 (m, 1H), 4.20–4.07 (m, 2H), 3.86–3.78 (m, 1H), 3.34–3.26 (m, 1H), 2.88 (t, *J* = 5.9 Hz, 2H), 2.78–2.66 (m, 4H), 2.35 (s, 3H), 2.32–2.16 (m, 2H), 1.94 (s, 3H), 1.80–1.59 (m, 4H); ^13^C NMR (100 MHz, CDCl_3_) *δ* 171.2, 166.9, 165.2, 148.3, 147.3, 145.0, 133.5, 133.4, 132.3, 129.9, 129.8, 128.8, 128.7, 128.4, 128.0, 122.4, 120.9, 97.5, 71.4, 69.1, 68.6, 68.4, 64.8, 52.1, 46.8, 46.0, 35.4, 29.7, 28.5, 28.3, 25.04, 24.96, 23.0, 21.6. HRMS m/z calcd for C_43_H_49_N_7_O_12_SNa [M + Na]^+^ 910.3052, found 910.3043.

*Synthesis of compound **15d**.* Compound **9** (100.0 mg, 0.15 mmol, 1.0 equiv), *t*-BuOH: THF: H_2_O 1:1:1 (v:v:v 1:1:1, 3.0 mL), **10e** (45.9 mg, 0.19 mmol, 1.3 equiv), CuSO_4_·5H_2_O (7.3 mg, 0.03 mmol, 0.2 equiv), NaAsc (11.9 mg, 0.06 mmol, 0.4 equiv), 16 h. Purified by flash chromatography (DCM to 5% MeOH/DCM) to obtain a yellowish slurry (113.0 mg, 83%) as the desired product (R_f_ = 0.46 in 5% MeOH/DCM). ^1^H NMR (400 MHz, CDCl_3_) *δ* 7.90–7.84 (m, 4H), 7.69 (d, *J* = 8.3 Hz, 2H), 7.56–7.45 (m, 2H), 7.44–7.30 (m, 6H), 7.21 (d, *J* = 8.2 Hz, 2H), 6.36 (d, *J* = 9.1 Hz, 1H), 5.59 (t, *J* = 9.7 Hz, 1H), 5.31 (t, *J* = 9.7 Hz, 1H), 4.84 (d, *J* = 3.5 Hz, 1H), 4.66–4.57 (m, 3H), 4.54–4.46 (m, 2H), 4.31–4.25 (m, 1H), 4.20–4.08 (m, 2H), 3.87–3.80 (m, 1H), 3.38–3.20 (m, 1H), 2.90 (t, *J* = 6.0 Hz, 2H), 2.73–2.65 (m, 4H), 2.35 (s, 3H), 2.32–2.19 (m, 2H), 1.93 (s, 3H), 1.73–1.59 (m, 4H), 1.36–1.25 (m, 2H); ^13^C NMR (100 MHz, CDCl_3_) *δ* 170.8, 167.0, 165.2, 148.6, 147.5, 145.0, 133.54, 133.46, 132.3, 129.9, 129.8, 128.8, 128.7, 128.4, 128.0, 122.2, 121.0, 97.5, 71.5, 69.1, 68.6, 68.4, 64.8, 52.0, 47.0, 45.9, 35.2, 29.9, 29.0, 28.4, 28.0, 25.2, 25.0, 23.0, 21.6. HRMS m/z calcd for C_44_H_51_N_7_O_12_SNa [M + Na]^+^ 924.3209, found 924.3213.

Compounds **16a-d**, **22**, **24** were prepared similarly as for compound **13c**. The amount of all chemicals, reaction time, yield, R_f_ value, and characterization of the product are listed, respectively. The macrocycles were typically obtained first as a clear waxy liquid, which upon standing for a few days, turned to a white solid.

*Synthesis of compound **16a (DM24)**.* Compound **15a** (75.0 mg, 0.086 mmol), DMF (12.0 mL), K_2_CO_3_ (23.7 mg, 0.17 mmol), 85 °C for 4.0 h. The crude was purified by flash chromatography (DCM to 2% MeOH/DCM) to obtain a white solid (54.0 mg, 90%) as the desired product **16a** (R_f_ = 0.14 in 5% MeOH/DCM). The reaction was also carried out using CH_3_CN as the solvent: Compound **15a** (50.0 mg, 0.057 mmol) was dissolved in CH_3_CN (12.0 mL), then K_2_CO_3_ (15.8 mg, 0.11 mmol) was added, the reaction mixture was stirred at 75 °C for 11.0 h. The solvent was removed using a rotavap, and the crude was purified using flash column similarly to give the pure product (31.0 mg, 77%). m.p. 127.0–129.0 °C; ^1^H NMR (400 MHz, CDCl_3_) *δ* 7.94–7.86 (m, 4H), 7.54–7.44 (m, 2H), 7.39–7.32 (m, 5H), 7.30 (s, 1H), 5.96 (d, *J* = 9.3 Hz, 1H), 5.55 (t, *J* = 10.8 Hz, 1H), 5.38 (t, *J* = 9.9 Hz, 1H), 4.73 (d, *J* = 3.5 Hz, 1H), 4.70–4.58 (m, 4H), 4.51–4.43 (m, 1H), 4.21–4.14 (m, 1H), 4.13–4.00 (m, 3H), 3.88–3.78 (m, 1H), 3.02–2.93 (m, 1H), 2.89–2.82 (m, 1H), 2.80–2.70 (m, 2H), 2.66–2.53 (m, 2H) 1.92 (s, 3H), 1.67–1.55 (m, 4H); ^13^C NMR (100 MHz, CDCl_3_) *δ* 170.3, 170.1, 166.8, 165.2, 148.8, 147.9, 133.6, 133.4, 129.8, 129.7, 128.8, 128.7, 128.5, 128.4, 121.6, 120.7, 98.2, 70.9, 69.2, 68.1, 66.9, 62.8, 51.9, 50.3, 45.5, 34.8, 27.7, 24.8, 24.7, 23.2; HRMS m/z calcd for C_35_H_39_N_7_O_9_Na [M + Na]^+^ 724.2701, found 724.2704.

*Synthesis of compound **16b (DM25)**.* Compound **15b** (68.0 mg, 0.077 mmol), DMF (12.0 mL), K_2_CO_3_ (21.2 mg, 0.15 mmol), 80 °C for 2.0 h. Purified by flash chromatography (DCM to 2% MeOH/DCM) to obtain a white solid (48.0 mg, 88%) as the desired product (R_f_ = 0.2 in 5% MeOH/DCM). The reaction was also carried out in acetonitrile: Compound **15b** (50.0 mg, 0.05 mmol, 1.0 equiv), CH_3_CN (10.0 mL), K_2_CO_3_ (15.6 mg, 0.11 mmol, 2.0 equiv), 75 °C for 14.0 h, yield 28.5 mg, 71%. M.p. 239.0–240.0 °C; ^1^H NMR (400 MHz, CDCl_3_) *δ* 7.92–7.85 (m, 4H), 7.62 (s, 1H), 7.54–7.47 (m, 2H), 7.46 (s, 1H), 7.39–7.30 (m, 4H), 5.91 (d, *J* = 9.3 Hz, 1H), 5.56 (t, *J* = 9.5 Hz, 1H), 5.30 (t, *J* = 9.9 Hz, 1H), 4.67 (d, *J* = 3.5 Hz, 1H), 4.65–4.55 (m, 4H), 4.51–4.45 (m, 1H), 4.36–4.30 (m, 1H), 4.16–4.08 (m, 2H), 4.02–3.95 (m, 1H), 3.93–3.87 (m, 1H), 3.03–2.90 (m, 2H), 2.79–2.70 (m, 4H), 1.91 (s, 3H), 1.75–1.66 (m, 4H), 1.32–1.25 (m, 2H); ^13^C NMR (100 MHz, CDCl_3_) *δ* 170.1, 169.9, 166.9, 165.4, 148.2, 147.6, 133.6, 133.5, 129.83, 129.81, 128.8, 128.6, 128.5, 128.4, 122.2, 121.6, 97.9, 71.2, 69.6, 68.6, 66.8, 63.4, 52.0, 49.6, 45.2, 34.3, 27.5, 27.3, 26.1, 24.5, 23.2; HRMS m/z calcd for C_36_H_41_N_7_O_9_Na [M + Na]^+^ 738.2858, found 738.2862.

*Synthesis of compound **16c (DM34)**.* Compound **15c** (120.0 mg, 0.14 mmol), DMF (15.0 mL), K_2_CO_3_ (37.3 mg, 0.27 mmol), 75 °C for 2.0 h. Purified by flash chromatography (DCM to 3% MeOH/DCM) to obtain a white solid (82.0 mg, 85%) as the desired product, R_f_ = 0.21 in 5% MeOH/DCM. The reaction was also carried out in acetonitrile: Compound **15c** (50.0 mg, 0.05 mmol, 1.0 equiv), CH_3_CN (8.0 mL), K_2_CO_3_ (15.7 mg, 0.11 mmol, 2.0 equiv), 75 °C for 14.0 h, yield 31.5 mg, 78%, m.p. 99.0–101.0 °C; ^1^H NMR (400 MHz, CDCl_3_) *δ* 7.93–7.89 (m, 4H), 7.54–7.47 (m, 2H), 7.39–7.34 (m, 6H), 6.09 (d, *J* = 8.8 Hz, 1H), 5.54 (t, *J* = 10.6 Hz, 1H), 5.46 (t, *J* = 9.6 Hz, 1H), 4.77 (d, *J* = 3.7 Hz, 1H), 4.60–4.44 (m, 5H), 4.33–4.26 (m, 1H), 4.17–4.03 (m, 2H), 3.59–3.49 (m, 1H), 3.30–3.21 (m, 1H), 2.83–2.70 (m, 6H), 2.33–2.20 (m, 2H), 1.94 (s, 3H), 1.80–1.69 (m, 2H), 1.69–1.59 (m, 2H); ^13^C NMR (100 MHz, CDCl_3_) *δ* 170.3, 170.1, 167.1, 165.3, 148.2, 147.8, 133.6, 133.5, 129.9, 129.7, 128.8, 128.76, 128.5, 128.4, 121.7, 121.2, 97.7, 71.3, 69.7, 67.7, 64.8, 63.2, 52.4, 46.4, 45.4, 34.8, 29.6, 28.0, 25.1, 24.8, 23.1; HRMS m/z calcd for C_36_H_41_N_7_O_9_Na [M + Na]^+^ 738.2858, found 738.2855.

*Synthesis of compound **16d (DM35)**.* Compound **15d** (90.0 mg, 0.10 mmol), DMF (12.0 mL), K_2_CO_3_ (27.5 mg, 0.19 mmol), 80 °C for 3.0 h. The crude was purified by flash chromatography (DCM to 3% MeOH/DCM) to obtain a white solid upon standing (57.0 mg, 78%) as the desired product (R_f_ = 0.26 in 5% MeOH/DCM). The reaction was also carried out using CH_3_CN as the solvent: Compound **15d** (50.0 mg, 0.055 mmol), CH_3_CN (8.0 mL), K_2_CO_3_ (15.3 mg, 0.11 mmol), 75 °C for 15.0 h, yield 30.0 mg, 74%. m.p. 92.0–94.0 °C; ^1^H NMR (400 MHz, CDCl_3_) *δ* 7.94–7.89 (m, 4H), 7.55–7.46 (m, 2H), 7.42 (s, 1H), 7.41 (s, 1H), 7.38–7.32 (m, 4H), 6.10 (d, *J* = 9.0 Hz, 1H), 5.57 (t, *J* = 9.6 Hz, 1H), 5.40 (t, *J* = 9.8 Hz, 1H), 4.84 (d, *J* = 3.7 Hz, 1H), 4.61–4.44 (m, 5H), 4.36–4.28 (m, 1H), 4.25–4.15 (m, 2H), 3.76–3.65 (m, 1H), 3.44–3.33 (m, 1H), 2.99–2.81 (m, 2H), 2.80–2.73 (m, 2H), 2.73–2.67 (m, 2H), 2.36–2.23 (m, 2H), 1.93 (s, 3H), 1.74–1.65 (m, 4H), 1.34–1.27 (m, 2H); ^13^C NMR (100 MHz, CDCl_3_) *δ* 170.3, 169.8, 167.0, 165.4, 148.3, 147.7, 133.6, 133.5, 129.9, 129.83, 128.80, 128.7, 128.5, 128.4, 122.0, 121.4, 97.6, 71.5, 69.7, 68.4, 65.6, 63.7, 52.3, 46.9, 45.3, 34.5, 29.9, 28.1, 27.8, 26.7, 24.8, 24.8, 23.1; HRMS m/z calcd for C_37_H_43_N_7_O_9_Na [M + Na]^+^ 752.3014, found 752.3011.

*Synthesis of compound****18***. To a 50 mL RBF, compound **17** (200.0 mg, 0.55 mmol) was dissolved in HOAc: H_2_O (v:v 4:1, 5.0 mL) and heated at 75 °C for 4 h. The reaction was stopped, and solvent was dried under vacuum to afford the crude, which was purified by flash chromatography using eluent from DCM to 3% MeOH/DCM to obtain a colorless liquid (129 mg, 85%) as the desired product (R_f_ = 0.3 in 10% MeOH/DCM). ^1^H NMR (400 MHz, DMSO-*d*_6_) *δ* 7.97 (d, *J* = 8.4 Hz, 1H), 4.99 (d, *J* = 5.6 Hz, 1H), 4.80 (d, *J* = 5.7 Hz, 1H), 4.56 (d, *J* = 3.5 Hz, 1H), 4.50 (t, *J* = 6.0 Hz, 1H), 3.87–3.77 (m, 2H), 3.73–3.61 (m, 2H), 3.52–3.40 (m, 2H), 3.35–3.31 (m, 1H), 3.25 (s, 3H), 3.18–3.10 (m, 1H); ^13^C NMR (100 MHz, DMSO-*d*_6_) *δ* 167.5, 97.7, 72.7, 70.73, 70.65, 60.7, 54.2, 53.9, 50.5. LC-MS (ESI+) calcd for C_9_H_16_N_4_O_6_ [M + H]^+^ 277.1, found 277.1.

*Synthesis of compound****19***. In a 50 mL RBF, compound **18** (500 mg, 1.81 mmol) was taken and dissolved in pyridine (5.0 mL). To this solution, tosyl chloride (862.7 mg, 4.52 mmol) dissolved in pyridine (5.0 mL) was added dropwise at 0 °C. The reaction was monitored in 1.5 h to see full conversion. Pyridine was dried under vacuum and crude was coated on silica gel and isolated by flash chromatography using eluent from DCM to 2% MeOH/DCM to obtain a sticky colorless slurry (628 mg, 81%) as the desired product (R_f_ = 0.51 in 10% MeOH/DCM). ^1^H NMR (400 MHz, CDCl_3_) *δ* 7.80 (d, *J* = 8.3 Hz, 2H), 7.34 (d, *J* = 8.0 Hz, 2H), 6.63 (d, *J* = 9.0 Hz, 1H), 4.63 (d, *J* = 3.7 Hz, 1H), 4.36–4.27 (m, 2H), 4.11–3.99 (m, 3H), 3.78–3.71 (m, 1H), 3.67 (t, *J* = 9.6 Hz, 1H), 3.49 (t, *J* = 9.4 Hz, 1H), 3.35 (s, 3H), 3.06 (br s, 2H), 2.44 (s, 3H); ^13^C NMR (100 MHz, CDCl_3_) *δ* 168.3, 145.0, 132.8, 129.9, 128.0, 98.2, 73.4, 70.6, 69.5, 69.0, 55.4, 53.3, 52.4, 21.6. LC-MS (ESI+) calcd for C_16_H_22_N_4_O_8_S [M + H]^+^ 431.1, found 431.1.

*Synthesis of compound **20**.* In a 50 mL RBF, compound **19** (628 mg, 1.46 mmol, 1.0 equiv), pyridine (3.0 mL) and benzoyl chloride (0.51 mL, 4.38 mmol, 3.0 equiv) was added at 0 °C dropwise and let it stir for 1.5 h to see full conversion. The reaction mixture was extracted with DCM (25.0 mL) and washed with NH_4_Cl (10.0 mL) and NaHCO_3_ (10.0 mL) followed by brine (10.0 mL). The organic layer was then dried over anhydrous Na_2_SO_4_ and concentrated under reduced pressure to obtain the crude. Crude was purified by flash chromatography using eluent from pure hexanes to 40% EtOAc/Hexanes to obtain a colorless liquid (850 mg, 91%) as the desired product (R_f_ = 0.35 in 40% EtOAc/Hexanes). ^1^H NMR (400 MHz, CDCl_3_) *δ* 7.91–7.81 (m, 4H), 7.72 (d, *J* = 8.3 Hz, 2H), 7.55–7.45 (m, 2H), 7.39–7.31 (m, 4H), 7.22 (d, *J* = 8.0 Hz, 2H), 6.63 (d, *J* = 9.4 Hz, 1H), 5.56 (dd, *J* = 10.8, 9.5 Hz, 1H), 5.36 (t, *J* = 9.6 Hz, 1H), 4.78 (d, *J* = 3.5 Hz, 1H), 4.50–4.42 (m, 1H), 4.23–4.16 (m, 2H), 4.15–4.09 (m, 1H), 3.85 (d, *J* = 16.6 Hz, 1H), 3.73 (d, *J* = 16.6 Hz, 1H), 3.45 (s, 3H), 2.37 (s, 3H); ^13^C NMR (100 MHz, CDCl_3_) *δ* 166.72, 166.69, 165.1, 144.9, 133.52, 133.46, 132.5, 129.9, 129.7, 128.7, 128.6, 128.4, 128.0, 97.9, 71.3, 68.8, 68.11, 68.05, 55.7, 52.5, 52.2, 21.6. LC-MS (ESI+) calcd for C_30_H_31_N_4_O_10_S [M + H]^+^ 639.2, found 639.2.

*Synthesis of compound****21***. Compound **20** (110.0 mg, 0.17 mmol, 1.0 equiv) in *t*-BuOH: THF: H_2_O (v:v:v 1:1:1, 3.0 mL) and 10-undecynoic acid **10c** (40.8 mg, 0.22 mmol, 1.3 equiv), CuSO_4_∙5H_2_O (8.6 mg, 0.034 mmol, 0.2 equiv), NaAsc (13.7 mg, 0.069 mmol, 0.4 equiv) and Tris[(1-benzyl-1H-1,2,3-triazol-4-yl)methyl]amine (TBTA) (18.3 mg, 0.03 mmol, 0.2 equiv) was added and the reaction mixture was stirred at rt for 7 h. The reaction was stopped, and solvent was dried under vacuum to afford the crude, which was purified by flash chromatography using eluent from DCM to 2% MeOH/DCM to obtain white solid (95 mg, 67%) as the desired product (R_f_ = 0.5 in 5% MeOH/DCM). m.p. 74.5–77.0 °C. ^1^H NMR (400 MHz, CDCl_3_) *δ* 7.89–7.79 (m, 4H), 7.71 (d, *J* = 8.3 Hz, 2H), 7.54–7.47 (m, 2H), 7.38–7.32 (m, 4H), 7.21 (d, *J* = 7.9 Hz, 2H), 7.11 (s, 1H), 6.20 (d, *J* = 9.3 Hz, 1H), 5.54–5.48 (m, 1H), 5.34 (t, *J* = 9.6 Hz, 1H), 4.91 (d, *J* = 16.5 Hz, 1H), 4.83 (d, *J* = 16.5 Hz, 1H), 4.74 (d, *J* = 3.6 Hz, 1H), 4.49–4.41 (m, 1H), 4.22–4.09 (m, 3H), 3.40 (s, 3H), 2.72–2.61 (m, 2H), 2.36 (s, 3H), 2.33 (t, *J* = 7.4 Hz, 2H), 1.69–1.54 (m, 4H), 1.36–1.29 (m, 8H); ^13^C NMR (100 MHz, CDCl_3_) *δ* 178.4, 178.1, 166.5, 165.5, 165.0, 149.0, 144.9, 133.5, 132.5, 129.9, 129.8, 128.61, 128.58, 128.5, 128.4, 128.0, 121.9, 97.8, 71.5, 68.7, 68.1, 68.0, 55.8, 52.6, 52.4, 33.91, 33.88, 29.1, 29.01, 28.97, 28.96, 28.93, 28.89, 28.87, 28.6, 28.4, 25.5, 24.71, 24.66, 21.6, 18.4. LC-MS (ESI+) calcd for C_41_H_49_N_4_O_12_S [M + H]^+^ 821.3, found 821.4.

*Synthesis of compound **23**.* Compound **20** (100 mg, 0.16 mmol) was mixed with THF: H_2_O: *t*-BuOH (v:v:v 1:1:1, 3.0 mL). To this solution, **10d** (45.0 mg, 0.20 mmol), CuSO_4_∙5H_2_O (7.8 mg, 0.031 mmol) and NaAsc (12.4 mg, 0.063 mmol) were added and the reaction mixture was stirred at rt for 12 h. The reaction was stopped, and solvent was dried under vacuum, and the crude was purified by flash chromatography using eluent from DCM to 2% DCM/MeOH to obtain a white solid (112 mg, 83%) as the desired product (R_f_ =0.27 in 5% MeOH/DCM). m.p. 93.5–95.5 °C; ^1^H NMR (400 MHz, CDCl_3_) *δ* 7.84–7.77 (m, 4H), 7.70 (d, *J* = 8.3 Hz, 2H), 7.53–7.44 (m, 3H), 7.37–7.28 (m, 4H), 7.20 (d, *J* = 8.1 Hz, 2H), 7.14 (br s, 1H), 6.60 (d, *J* = 8.7 Hz, 1H), 5.55 (t, *J* = 10.3 Hz, 1H), 5.37 (t, *J* = 9.7 Hz, 1H), 4.99–4.84 (m, 2H), 4.77 (d, *J* = 3.3 Hz, 1H), 4.66–4.57 (m, 2H), 4.49–4.42 (m, 1H), 4.21–4.15 (m, 2H), 4.14–4.08 (m, 1H), 3.39 (s, 3H), 2.92 (t, *J* = 5.3 Hz, 2H), 2.73 (t, *J* = 6.0 Hz, 2H), 2.62–2.53 (m, 2H), 2.34 (s, 3H), 1.72–1.47 (m, 4H); ^13^C NMR (100 MHz, CDCl_3_) *δ* 172.4, 166.5, 165.7, 165.0, 148.3, 147.1, 145.0, 133.6, 132.4, 129.81, 129.76, 129.7, 128.6, 128.53, 128.45, 128.4, 128.0, 122.6, 122.3, 97.8, 71.6, 68.6, 68.0, 55.8, 52.5, 52.4, 45.9, 34.9, 28.3, 27.7, 24.9, 24.8, 21.6. LC-MS (ESI+) calcd for C_41_H_46_N_7_O_12_S [M + H]^+^ 860, found 860.

*Synthesis of compound **22 (C2ML)**.* Compound **21** (70.0 mg, 0.085 mmol), DMF (14.0 mL) and K_2_CO_3_ (23.5 mg, 0.17 mmol), 75 °C, 2 h. Purification by flash chromatography (DCM to 1% MeOH/DCM) to obtain **22** as a white solid (80%, 44 mg). R_f_ = 0.64 in 5% MeOH/DCM. m.p. 207.5–210.0 °C. ^1^H NMR (400 MHz, CDCl_3_) *δ* 7.98–7.88 (m, 4H), 7.55–7.48 (m, 3H), 7.41–7.34 (m, 4H), 5.55 (d, *J* = 9.3 Hz, 1H), 5.42–5.34 (m, 2H), 4.98 (d, *J* = 16.0 Hz, 1H), 4.80 (d, *J* = 16.0 Hz, 1H), 4.47–4.27 (m, 4H), 3.91–3.81 (m, 1H), 3.37 (s, 3H), 2.84 (t, *J* = 6.1 Hz, 2H), 2.29–2.07 (m, 2H), 1.78–1.67 (m, 2H), 1.67–1.49 (m, 2H), 1.39–1.19 (m, 8H); ^13^C NMR (100 MHz, CDCl_3_) *δ* 173.3, 166.6, 166.0, 165.6, 149.6, 133.5, 129.9, 129.8, 129.7, 129.0, 128.9, 128.5, 128.4, 122.5, 97.8, 72.6, 70.2, 68.7, 62.4, 56.0, 53.7, 53.4, 52.3, 34.2, 31.5, 30.4, 29.9, 29.6, 28.7, 25.4, 25.0. LC-MS (ESI+) calcd for C_34_H_41_N_4_O_9_ [M + H]^+^ 649, found 649.

*Synthesis of compound **24 (C2DLM)**.* Compound **23** (75.0 mg, 0.087 mmol), K_2_CO_3_ (24.1 mg, 0.17 mmol), DMF (10.0 mL), 70 °C, 2.0 h. Purification by flash chromatography (DCM to 2% MeOH/DCM) to obtain a white solid (46 mg, 77%) as the desired product. Using Na_2_CO_3_ as the base: Compound **23** (100.0 mg, 0.12 mmol, 1.0 equiv), Na_2_CO_3_ (24.6 mg, 0.23 mmol, 2.0 equiv), DMF (12 mL), 70 °C, 2.0 h. The pure compound **24** was obtained in 83% yield, 66 mg. R_f_ = 0.4 in 5% MeOH/DCM. m.p. 83.5–85.0 °C; ^1^H NMR (400 MHz, CDCl_3_) *δ*7.97–7.87 (m, 4H), 7.57–7.46 (m, 3H), 7.43–7.32 (m, 5H), 5.54 (d, *J* = 9.2 Hz, 1H), 5.35–5.28 (m, 1H), 5.24 (t, *J* = 9.9 Hz, 1H), 4.93 (d, *J* = 16.2 Hz, 1H), 4.83 (d, *J* = 16.2 Hz, 1H), 4.67–4.51 (m, 2H), 4.38–4.25 (m, 4H), 3.70–3.62 (m, 1H), 3.01–2.90 (m, 5H), 2.89–2.71 (m, 4H), 2.07–1.92 (m, 2H), 1.83–1.71 (m, 2H); ^13^C NMR (100 MHz, CDCl_3_) *δ* 170.7, 166.5, 165.7, 165.6, 149.2, 147.3, 133.6, 133.4, 129.88, 129.85, 128.9, 128.7, 128.5, 128.4, 122.2, 121.8, 97.4, 72.2, 69.7, 68.3, 63.6, 55.3, 53.4, 52.0, 45.5, 34.8, 29.4, 27.6, 26.1, 25.2. LC-MS (ESI+) calcd for C_34_H_38_N_7_O_9_ [M + H]^+^ 688, found 688.

*Synthesis of compound **25 (C2DDL)***. Compound **23** (100.0 mg, 0.12 mmol), Cs_2_CO_3_ (75.8 mg, 0.23 mmol), DMF (12 mL), 70 °C, 2.0 h. After solvent was removed, the crude was purified by flash chromatography (DCM to 5% MeOH/DCM) to obtain the dimeric macrodilactone compound **25** as a white solid (41 mg, 51 %). R_f_ = 0.32 in 5% MeOH/DCM). m.p. 257.0–258.0 °C; ^1^H NMR (400 MHz, CDCl_3_) *δ* 8.03–7.97 (m, 4H), 7.96–7.91 (m, 4H), 7.54–7.48 (m, 6H), 7.47 (s, 2H), 7.42–7.33 (m, 8H), 5.31–5.21 (m, 4H), 5.16 (t, *J* = 9.9 Hz, 2H), 5.09 (d, *J* = 16.8 Hz, 2H), 4.73–4.64 (m, 4H), 4.56–4.49 (m, 2H), 4.47 (d, *J* = 4.1 Hz, 2H), 4.38–4.27 (m, 6H), 3.56–3.47 (m, 2H), 3.07 (s, 6H), 2.99–2.92 (m, 4H), 2.88–2.69 (m, 8H), 2.06–1.95 (m, 4H), 1.77–1.68 (m, 4H); ^13^C NMR (100 MHz, CDCl_3_) *δ* 169.9, 166.7, 165.7, 165.4, 149.3, 148.8, 133.5, 133.4, 132.1, 130.0, 129.9, 128.86, 128.85, 128.5, 128.4, 122.8, 97.4, 72.7, 70.0, 68.6, 63.6, 55.5, 53.1, 51.8, 50.4, 33.9, 29.8, 27.1, 26.1, 25.3. LC-MS (ESI+) calcd for C_68_H_74_N_14_O_18_Na [M + Na]^+^ 1398 found 1398.

*Synthesis of compound **31** and a general procedure and example in Table 5*.

Sugar azide **26** (40.0 mg, 0.11 mmol, 1.0 equiv) and 4-*t*-butylphenylacetylene (29.0 µL, 0.16 mmol, 1.5 equiv) were dissolved in EtOH:H_2_O (v 1:1, 2.0 mL), then CuSO_4_·5H_2_O (5.36 mg, 0.021 mmol, 0.2 equiv) and sodium ascorbate (8.51 mg, 0.043 mmol, 0.4 equiv) were added to the reaction mixture. To this mixture, macrocycle **DM25** (3.86, 5.0 mol%) was added. The reaction was stirred rt for 5.0 h to at which time the starting material was full converted to product, as indicated by ^1^H NMR. The reaction was stopped, and solvent was removed using a rotavap, to obtain the crude, which was further purified with flash chromatography using eluent of DCM to 1% MeOH/DCM to obtain the desired product as white solid (54 mg, 95%), R_f_ = 0.35 in 5% MeOH/DCM. ^1^H NMR (400 MHz, CDCl_3_) *δ* 8.08 (s, 1H), 7.76 (d, *J* = 8.5 Hz, 2H), 7.44 (d, *J* = 8.5 Hz, 2H), 6.41 (d, *J* = 9.1 Hz, 1H), 6.11 (d, *J* = 9.9 Hz, 1H), 5.52 (dd, *J* = 10.3, 9.5 Hz, 1H), 5.26 (t, *J* = 9.7 Hz, 1H), 4.64 (q, *J* = 9.8 Hz, 1H), 4.35–4.27 (m, 1H), 4.19–4.12 (m, 1H), 4.05–3.97 (m, 1H), 2.07 (s, 9H), 1.76 (s, 3H), 1.34 (s, 9H); ^13^C NMR (100 MHz, CDCl_3_) *δ* 170.70, 170.66, 170.6, 169.3, 151.8, 148.0, 126.9, 125.9, 125.7, 118.8, 85.9, 75.0, 72.4, 68.0, 61.7, 53.6, 34.7, 31.3, 22.8, 20.7, 20.60, 20.58. LC-MS (ESI+) calcd for C_26_H_34_N_4_O_8_Na [M + Na]^+^ 553 found 553.

## 4. Conclusions

In summary, we have synthesized several series of novel *N*-acetyl-D-glucosamine and triazole-derived macrolactones through intramolecular S_N_2 reactions in short steps and high efficiency. The macrocyclization did not require high dilution and was carried out at about 10 mM concentrations, affording the mono-macrolactones in 76–90% yields. Eleven macrolactones cyclized from C1 to C6, and three macrocycles cyclized from C2 to C6 were synthesized and characterized. The macrocycles showed interesting anion binding properties with tetrabutylammonium halides. TBACl showed the strongest complexation with the macrocycles. Moreover, several bistriazole-containing macrolactones also formed complexes with Cu (II) ions. As indicated by ^1^H NMR spectra, the amide proton and triazole signals exhibited significant chemical shift changes, which indicated that the Cu (II) or anions are binding to the macrolactones in a specific manner and may be useful for molecular recognition. The applications of these macrocycles as ligands for copper ions for the cycloaddition reactions of azide and alkynes (AAC) were explored. Interestingly the bis-triazole-containing macrocycles were highly efficient ligands for accelerating the AAC reactions significantly, and the monotriazole-based macrolactones were not as effective. The rate accelerating effects of macrocycles were also selective towards different acetylenes. The methods of synthesizing these novel sugar-based macrocycles should apply to other carbohydrate derivatives.

## Figures and Tables

**Table 1:** Macrolactonization of the intermediate **23** using different bases.

Entry	Amount (mg)	Concentration (mM)	Base 2.0 equiv	DMF (mL)	Temp (°C)	Yield %	
						Mono 24	Di 25
1	100	9.7	Cs_2_CO_3_	12	70	0	51
2	75	8.7	K_2_CO_3_	10	70	77	0
3	100	9.7	Na_2_CO_3_	12	70	83	0

**Table 2:** Effect of the macrocycles on catalyzing the click reactions of phenylacetylene.

**Entry**	**MC Code**	**mol% of MC**	**Conversion (%)**				**Isolated Yields for 28**
			(a) 1 h, (b) 0.5 h	2 h	5 h	9 h	
(a)	None	0		15	21	N/A	
1	LM24	2.5	38	50	57	68	
2	LM26	2.5	57	61	78	85	70%
3	LM28	2.5	57	61	67	79	
4	LM34	2.5	55	66	79	93	82%
5	LM36	2.5	56	60	77	89	80%
6	LM38	2.5	49	49	51	51	
7	DM24	2.5	51	51	54	58	
8	DM25	2.5	68	84	100		92%
9	DM34	2.5	53	60	61	65	
10	DM35	2.5	68	81	100		96%
(b)	None	0		18	21		
11	LM26	5.0	51	74	100		88%
12	LM34	5.0	42	51	94		79%
13	LM36	5.0	27	34	87		
14	DM24	2.5	43	56	70		
15	DM25	2.5	46	59	100		93%
16	DM34	2.5	46	54	68		
17	DM35	2.5	35	42	100		94%
18	C2ML	2.5		10	20	40, 10 h	
19	C2DLM	2.5		27	56	79, 10 h	

Condition (a): Sugar azide (1.0 equiv, 40.0 mg), CuSO_4_∙5H_2_O (0.2 equiv, 5.4 mg), phenylacetylene (1.2 equiv, 14.0 μL), EtOH/H_2_O (*v*/*v* 1:1, 2.0 mL), macrocycle, NaAsc (0.4 equiv, 8.5 mg). Condition (b) The same as (a) except 1.5 equiv of alkynes and 0.5 equiv of NaAsc were used.

**Table 3:** Effect of the macrocycle on catalyzing the click reaction of 1-octyne (R = *n*-Hexyl).

Entry	MC Code	mol% MC	Conversion (%) at 1 h	Conversion (%) at 2 h	Conversion (%) at 5 h	Isolated Yield 29
1	None	0	11	14	20	
2	LM26	5.0	8	12	25	
3	LM34	5.0	16	18	28	
4	LM36	5.0	12	14	23	
5	DM24	2.5	27	35	46	
6	DM25	2.5	12	25	57	
7	DM34	2.5	100			87%
8	DM35	2.5	20	22	37	

Condition: Sugar azide (1 equiv, 40.0 mg), CuSO_4_∙5H_2_O (0.1 equiv, 2.7 mg), 1-octyne (1.5 equiv, 23.7 μL), EtOH/H_2_O (*v*/*v* 1:1, 2.0 mL), NaAsc (0.3 equiv, 6.4 mg).

**Table 4:** Effect of the macrocycles on CuAAC of sugar azide with 5-phenyl-1-pentyne.

Entry	Macrocycle	mol% of MC	CuSO_4_ (equiv)	Conversion (%)			Isolated Yield 30
				1 h	2 h	5 h	
1	None	0	0.01	2	5	27	
2	None	0	0.05	33	51		
3	DM35	2.5	0.01	29	52	74	
4	DM35	1.0	0.02	58	88	100	86%
5	DM24	2.5	0.02	62	88	100	79%
6	DM25	1.0	0.05	77	98		86%
7	DM34	2.5	0.05	100			77%
8	DM35	2.5	0.05	100			87%

Conditions: Sugar azide (1.0 equiv, 40.0 mg), CuSO_4_∙5H_2_O (0.05 equiv, 1.3 mg or other amount), 5-phenyl-1-pentyne (1.5 equiv, 24.5 μL), EtOH/H_2_O (*v*/*v* 1:1, 2.0 mL), macrocycle (1.0–2.5 mol%), NaAsc (three folds of the molar amount of copper sulfate).

**Table 5:** Effect of the macrocycles on copper-mediated click reaction of sugar azide with 4-tert-butylphenylacetylene (R = *p*-*t*-Bu-C_6_H_4_-).

Entry	Macrocycle	mol% of MC	CuSO_4_ (equiv)	Conversion (%)				Isolated Yield 31
				2 h	5 h	10 h	20 h	
1	None	0	0.1 ^a^	4	10		17	
2	DM25	2.5	0.1 ^a^	47	80		100	91%
3	DM34	2.5	0.1 ^a^	15	34		70	
4	DM35	2.5	0.1 ^a^	28	45		100	86%
5	None	0	0.2 ^b^	42	62	83		
6	DM24	5.0	0.2 ^b^	61	78	87		
7	DM25	2.5	0.2 ^b^	30	100			93%
8	DM25	5.0	0.2 ^b^	43	100			95%
9	DM34	5.0	0.2 ^b^	48	75	84		
10	DM35	5.0	0.2 ^b^	68	98	100		89%

Condition **^a^**: Sugar azide (1.0 equiv), CuSO_4_∙5H_2_O (0.1 equiv), p-*t*-butyl-phenylacetylene (1.5 equiv), EtOH/H_2_O (*v*/*v* 1:1, 2.0 mL), NaAsc (0.3 equiv); Condition **^b^**: The same as “a” except 0.2 equiv of CuSO_4_·H_2_O and 0.4 equiv of NaAsc were used.

## Data Availability

Not applicable.

## Supplementary Materials

The following are available online. Synthesis of compounds **S2-S5**, **6c**, **6e**, **7c** and **9**; ^1^H and ^13^C NMR spectra for all compounds, 2D HSQC and COSY NMR spectra for compounds **8c**, **11a-b**, **12a-c**, **13a-c**, **14a-c**, **15a**, **16a-d**, **DLM28**, **18**, **19**, **20**, **21**, **22**, **23**, **24**, **25** and **31**; anion binding studies for TBAX are included in Figures S1–S22; stacked NMR spectra for copper complexes are included in Figures S23–S34; screening reactions conditions and stacked NMR spectra are included on page S97, Tables S1–S8 and Figures S35–S42; FTIR spectra for macrocycles are on pages S111–S118.

## References

[1] Yu X., Sun D. (2013). Macrocyclic drugs and synthetic methodologies toward macrocycles. Molecules.

[2] Madsen C.M., Clausen M.H. (2011). Biologically Active Macrocyclic Compounds—From Natural Products to Diversity-Oriented Synthesis. Eur. J. Org. Chem..

[3] Xie J., Bogliotti N. (2014). Synthesis and Applications of Carbohydrate-Derived Macrocyclic Compounds. Chem. Rev..

[4] Singh K., Tripathi R.P. (2020). An Overview on Glyco-Macrocycles: Potential New Lead and their Future in Medicinal Chemistry. Curr. Med. Chem..

[5] Zong G., Barber E., Aljewari H., Zhou J., Hu Z., Du Y., Shi W.Q. (2015). Total Synthesis and Biological Evaluation of Ipomoeassin F and Its Unnatural 11R-Epimer. J. Org. Chem..

[6] Toure S., Barthelemy M., Sorres J., Genta-Jouve G., Dusfour I., Eparvier V., Stien D. (2018). Mucorolactone, a Macrolactone from *Mucor* sp. SNB-VECD13A, a Fungus Isolated from the Cuticle of a Vespidae Species. Org. Lett..

[7] Fürstner A. (2004). Total Syntheses and Biological Assessment of Macrocyclic Glycolipids. Eur. J. Org. Chem..

[8] Wu J., Lu J., Liu J., Zheng C., Gao Y., Hu J., Ju Y. (2017). A deoxycholic acid-based macrocycle: Recognition of mercury ion and cascade enantioselective sensing toward amino acids. Sens. Actuatorsb.

[9] Xiao W., Mo Y., Guo J., Su Z., Dong S., Feng X. (2021). Catalytic enantioselective synthesis of macrodiolides and their application in chiral recognition. Chem. Sci..

[10] Peng D., Lin X., Jiang L., Huang J., Zeng G., Deng X., Zhou Y. (2019). Five macrocyclic glycosides from Schoenoplectus tabernaemontani. Nat Prod Res.

[11] Desmond R.T., Magpusao A.N., Lorenc C., Alverson J.B., Priestley N., Peczuh M.W. (2014). De novo macrolide-glycolipid macrolactone hybrids: Synthesis, structure and antibiotic activity of carbohydrate-fused macrocycles. Beilstein J. Org. Chem..

[12] Li Y., Sun J., Gong Y., Yu B. (2011). Synthesis of Oligomeric 4-(Glycosyloxy)benzoate Macrocyclic Glycosides. J. Org. Chem..

[13] Zong G., Hu Z., O’Keefe S., Tranter D., Iannotti M.J., Baron L., Hall B., Corfield K., Paatero A.O., Henderson M.J. (2019). Ipomoeassin F Binds Sec61α to Inhibit Protein Translocation. J. Am. Chem. Soc..

[14] Fuerstner A., Ruiz-Caro J., Prinz H., Waldmann H. (2004). Structure Assignment, Total Synthesis, and Evaluation of the Phosphatase Modulating Activity of Glucolipsin A. J. Org. Chem..

[15] Si D., Peczuh M.W. (2016). Synthesis and structure of a carbohydrate-fused [15]-macrodilactone. Carbohydr. Res..

[16] Lin C., Maisonneuve S., Theulier C., Xie J. (2019). Synthesis and Photochromic Properties of Azobenzene-Derived Glycomacrolactones. Eur. J. Org. Chem..

[17] Lin C., Maisonneuve S., Metivier R., Xie J. (2017). Photoswitchable Carbohydrate-Based Macrocyclic Azobenzene: Synthesis, Chiroptical Switching, and Multi-stimuli-Responsive Self-Assembly. Chem. Eur. J..

[18] Tsai C.Y., Huang X., Wong C.H. (2000). Design and synthesis of cyclic sialyl Lewis X mimetics: A remarkable enhancement of inhibition by pre-organizing all essential functional groups. Tetrahedron Lett..

[19] Dheer D., Singh V., Shankar R. (2017). Medicinal attributes of 1,2,3-triazoles: Current developments. Bioorg. Chem..

[20] Thirumurugan P., Matosiuk D., Jozwiak K. (2013). Click Chemistry for Drug Development and Diverse Chemical-Biology Applications. Chem. Rev..

[21] Barlow T.M.A., Tourwe D., Ballet S. (2017). Cyclisation To Form Small, Medium and Large Rings by Use of Catalysed and Uncatalysed Azide-Alkyne Cycloadditions (AACs). Eur. J. Org. Chem..

[22] Singh M.S., Chowdhury S., Koley S. (2016). Advances of azide-alkyne cycloaddition-click chemistry over the recent decade. Tetrahedron.

[23] Haldon E., Nicasio M.C., Perez P.J. (2015). Copper-catalysed azide-alkyne cycloadditions (CuAAC): An update. Org. Biomol. Chem..

[24] Zhang X., Liu P., Zhu L. (2016). Structural determinants of alkyne reactivity in copper-catalyzed azide-alkyne cycloadditions. Molecules.

[25] Tiwari V.K., Mishra B.B., Mishra K.B., Mishra N., Singh A.S., Chen X. (2016). Cu-Catalyzed Click Reaction in Carbohydrate Chemistry. Chem. Rev..

[26] Wang G., Wang D., Bietsch J., Chen A., Sharma P. (2020). Synthesis of Dendritic Glycoclusters and Their Applications for Supramolecular Gelation and Catalysis. J. Org. Chem..

[27] Singh A., Khatri V., Malhotra S., Prasad A.K. (2016). Sugar-based novel chiral macrocycles for inclusion applications and chiral recognition. Carbohydr. Res..

[28] Pathigoolla A., Sureshan K.M. (2014). Reverse-CD mimics with flexible linkages offer adaptable cavity sizes for guest encapsulation. Chem. Commun..

[29] Bodine K.D., Gin D.Y., Gin M.S. (2005). Highly Convergent Synthesis of C3-or C2-Symmetric Carbohydrate Macrocycles. Org. Lett..

[30] Peng R., Xu Y., Cao Q. (2018). Recent advances in click-derived macrocycles for ions recognition. Chin. Chem. Lett..

[31] Picot S.C., Mullaney B.R., Beer P.D. (2012). Ion-pair recognition by a heteroditopic triazole-containing receptor. Chem.-Eur. J..

[32] Yu Y., Bogliotti N., Tang J., Xie J. (2013). Synthesis and Properties of Carbohydrate-Based BODIPY-Functionalized Fluorescent Macrocycles. Eur. J. Org. Chem..

[33] Chandrasekhar S., Rao C.L., Nagesh C., Reddy C.R., Sridhar B. (2007). Inter and intramolecular copper(I)-catalyzed 1,3-dipolar cycloaddition of azido-alkynes: Synthesis of furanotriazole macrocycles. Tetrahedron Lett..

[34] Maurya S.K., Rana R. (2017). An eco-compatible strategy for the diversity-oriented synthesis of macrocycles exploiting carbohydrate-derived building blocks. Beilstein J. Org. Chem..

[35] Chen A., Samankumara L.P., Dodlapati S., Wang D., Adhikari S., Wang G. (2019). Syntheses of Bis-Triazole Linked Carbohydrate Based Macrocycles and Their Applications for Accelerating Copper Sulfate Mediated Click Reaction. Eur. J. Org. Chem..

[36] Nie X., Wang G. (2005). Synthesis of a Ring-Oxygenated Variant of the 2-Carboxy-6-hydroxyoctahydroindole Core of Aeruginosin 298-A from Glucose. J. Org. Chem..

[37] Wang G., Chen A., Mangunuru H.P.R., Yerabolu J.R. (2017). Synthesis and characterization of amide linked triazolyl glycolipids as molecular hydrogelators and organogelators. Rsc Adv..

[38] Liu Y., Zhao W., Chen C.-H., Flood A.H. (2019). Chloride capture using a C-H hydrogen-bonding cage. Sci. (Wash. Dcu. S.).

[39] Chen Y., Wu G., Chen L., Tong L., Lei Y., Shen L., Jiao T., Li H. (2020). Selective Recognition of Chloride Anion in Water. Org. Lett..

[40] Deraedt C., Pinaud N., Astruc D. (2014). Recyclable Catalytic Dendrimer Nanoreactor for Part-Per-Million CuI Catalysis of “Click” Chemistry in Water. J. Am. Chem. Soc..

[41] Ubbink M., Lian L.Y., Modi S., Evans P.A., Bendall D.S. (1996). Analysis of the 1H-NMR chemical shifts of Cu(I)-, Cu(II)- and Cd-substituted pea plastocyanin. Metal-dependent differences in the hydrogen-bond network around the copper site. Eur. J. Biochem..

