# Supplementary material for: Synthesis of Carbohydrate Based Macrolactones and Their Applications as Receptors for Ion Recognition and Catalysis
Source: Molecules. 2021 Jun 3;26(11):3394. doi: 10.3390/molecules26113394 (PMC8199946; doi:10.3390/molecules26113394)
Supplement: Supplementary file 1 [file molecules-26-03394-s001.zip › molecules-1205503-supplementary.pdf]

Supplementary Materials

Synthesis of Carbohydrate Based Macrolactones and Their Applications as Receptors for Ion Recognition and Catalysis

Surya Adhikari, Anji Chen, Guijun Wang*

Department of Chemistry and Biochemistry, Old Dominion University, Norfolk, VA 23529, USA, email: g1wang@odu.edu

*Corresponding Author

Table of Contents

I. **Synthesis of compound S2-S5, 6c, 6e, 7c and 9……………………………...S2**

II. **^{1}H and ^{13}C NMR Spectra for Compounds Synthesized.……………………... S6**

III. **2D HSQC and COSY NMR spectra…………………………………………… S53**

IV. **TBAX Binding Studies…………………………………………….…………. S80**

V. **Copper Binding Studies………………………………………………………… S91**

VI. **Screening Macrocycles for Azide Alkyne Cycloadditions …………………… S99**

VII. **IR Spectra for Macrolactones …………………………….…………………… S111**

I. Synthesis of compound S2-S5, 6c, 6e, 7c and 9

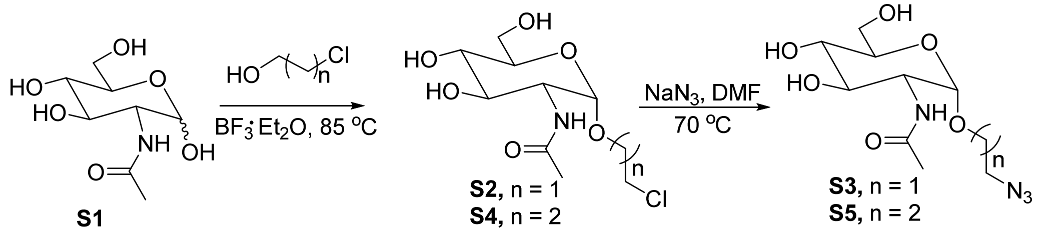

Scheme S1: Synthesis of intermediates from NAG starting material.

Synthesis of compound S2:[1,2] *N*-acetyl-D-glucosamine **S1** (5.0 g, 22.6 mmol, 1.0 equiv) was added to a 100 mL round bottomed flask (RBF) with 2-chloroethanol (7.75 mL, 113.05 mmol, 5.0 equiv) and BF_3•Et_2O (0.71 mL, 5.65 mmol, 0.25 equiv). The reaction mixture was stirred at 85 ºC for 4 h. The heating was turned off and the mixture was cooled to room temperature. The mixture was then treated with water (15 mL) and washed with DCM (20 mL x 2) to remove excess 2-chloroethanol. The aqueous layer was then collected and evaporated. The crude product was obtained as dark brown oily crude. It was then dissolved in MeOH and coated on to SiO_2 (dry loading) for purification. The crude was then purified by chromatography on silica gel using a gradient of dichloromethane (DCM) and methanol from pure DCM to up to 10% MeOH/DCM (R_f = 0.13 in 10% MeOH/DCM). The desired product was obtained as a white solid (4.01 g, 63%). ^{1}H NMR (400 MHz, D_2O + 1 drop MeOH) δ 4.91 (d, J =3.6 Hz, 1H), 3.99-3.70 (m, 9H), 3.49-3.40 (m, 1H), 2.02 (s, 3H); ^{13}C NMR (100 MHz, D_2O + 1 drop MeOH) δ 175.1, 97.7, 72.7, 71.5, 70.6, 68.9, 61.2, 54.3, 44.2, 22.5. LC-MS (ESI+) m/z calcd for $C_{10}H_{19}ClNO_6$ $[M + H]^+$ 284.1, found 284.1.

Synthesis of compound S3: [1-3] Compound **S2** (4.0 g, 14.09 mmol, 1.0 equiv) was taken in DMF (8.0 mL) and NaN_3 (3.7 g, 56.39 mmol, 4.0 equiv) was added to the mixture and the reaction was heated at 85 °C for 5 h. At which point the ^{1}H NMR spectrum of the reaction mixture indicated the complete consumption of the starting material. The heating was then turned off and the resulted suspension was filtered, and the filtrate was collected and concentrated to afford the crude product, which was purified by flash chromatography (DCM to 10% MeOH/DCM) to obtain a white solid (3.5 g, 86%) as the desired product. Compound **S3** was synthesized in a one-pot reaction from the *N*-acetyl-D-glucosamine **S1** in 74% yield following literature procedure.[3] (R_f = 0.25 in 10% MeOH/DCM). m.p. 120.0-122.0 °C. ^{1}H NMR (400 MHz, D_2O + 1 drop MeOH) δ 4.91 (d, J = 3.6

Hz, 1H), 3.95-3.81 (m, 3H), 3.80-3.61 (m, 4H), 3.55-3.42 (m, 3H), 2.02 (s, 3H); ^{13}C NMR (100 MHz, D_2O + 1 drop MeOH) δ 175.2, 97.6, 72.7, 71.5, 70.6, 67.2, 61.2, 54.2, 50.9, 22.5; LC-MS (ESI+) m/z calcd for $C_{10}H_{19}N_4O_6$ $[M + H]^+$ 291.1, found 291.1.

Synthesis of compound 6c:[3] Compound **S3** (160.0 mg, 0.55 mmol, 1.0 equiv), pyridine (1.5 mL), TsCl (210.2 mg, 1.10 mmol, 2.0 equiv), 10 h. Purified by flash chromatography (DCM to 10% MeOH/DCM) to obtain a white foam (186.0 mg, 76%) as the desired product. (R_f = 0.38 in 10% MeOH/DCM). ^{1}H NMR (400 MHz, $CDCl_3$) δ 7.80 (d, *J* = 8.3 Hz, 2H), δ 7.34 (d, *J* = 8.3 Hz, 2H), 6.08 (d, *J* = 8.6 Hz, 1H), 4.81 (d, *J* = 3.8 Hz, 1H), 4.33 (dd, *J* = 11.0, 2.1 Hz, 1H), 4.26 (dd, *J* = 11.0, 5.7 Hz, 1H), 4.11-4.03 (m, 1H), 3.93-3.86 (m, 1H), 3.83-3.76 (m, 1H), 3.71-3.63 (t, *J* = 9.6 Hz, 1H), 3.62-3.43 (m, 3H), 3.35-3.28 (m, 1H), 2.57 (br s, 2H), 2.45 (s, 3H), 2.03 (s, 3H). ^{13}C NMR (100 MHz, $CDCl_3$) δ 172.4, 145.0, 132.8, 129.9, 128.0, 97.6, 73.7, 70.9, 69.9, 69.0, 67.3, 53.4, 50.5, 23.1, 21.7. LC-MS (ESI+) m/z calcd for $C_{17}H_{25}N_4O_8S$ $[M + H]^+$ 445, found 445.

Synthesis of compound 7c:[3] To a 50 mL RBF, compound **6c** (500.0 mg, 1.12 mmol, 1.0 equiv), DCM (5.0 mL), pyridine (0.45 mL, 5.62 mmol, 5.0 equiv) were added in the given order and the mixture was cooled to 0 °C. To the stirring mixture benzoyl chloride (0.32 mL, 2.81 mmol, 2.5 equiv) was added dropwise. The mixture was allowed to warm up to rt and the stirring was continued for 4 more hours. ^{1}H NMR spectrum indicated the completion of the reaction. Work up was performed with H_2O (25 mL) and DCM (25 mL x 3). The combined organic layer was dried over Na_2SO_4 (anhydrous), filtered and concentrated to afford the crude, which was purified by column chromatography using eluent from pure DCM to 2% MeOH/DCM to give a colorless oil (572 mg, 78%) as the desired product (R_f = 0.6 in 5% MeOH/DCM). Compound **7c** was also synthesized in a one-pot reaction from compound **S3** following the literature procedure.[3] ^{1}H NMR (400 MHz, $CDCl_3$) δ 7.91-7.83 (m, 4H), 7.71 (d, *J* = 8.3 Hz, 2H), 7.55-7.45 (m, 2H), 7.39-7.31 (m, 4H), 7.22 (d, *J* = 8.1 Hz, 2H), 5.91 (d, *J* = 9.5 Hz, 1H), 5.62 (dd, *J* = 10.9, 9.5 Hz, 1H), 5.36 (t, *J* = 9.5 Hz, 1H), 4.96 (d, *J* = 3.6 Hz, 1H), 4.56-4.48 (m, 1H), 4.29-4.10 (m, 3H), 4.03-3.97 (m, 1H), 3.71-3.64 (m, 1H), 3.63-3.55 (m, 1H), 3.44-3.36 (m, 1H), 2.37 (s, 3H), 1.85 (s, 3H); ^{13}C NMR (100 MHz, $CDCl_3$) δ 170.1, 166.9, 165.1, 145.0, 133.6, 133.5, 132.4, 130.0, 129.84, 129.78, 128.7, 128.6, 128.4, 128.3, 128.0, 97.5, 71.1, 68.9, 68.6, 68.3, 67.8, 52.0, 50.4, 23.0, 21.6; LC-MS (ESI+) m/z calcd for $C_{31}H_{33}N_4O_{10}S$ $[M + H]^+$ 653.2, found 653.2.

Synthesis of compound S4:[4] *N*-acetyl-D-glucosamine **S1** (5.0 g, 22.6 mmol, 1.0 equiv) was added to a 100 mL RBF with 3-chloro-1-propanol (7.56 mL, 90.4 mmol, 4.0 equiv) and $BF_3•Et_2O$ (0.56 mL, 4.5 mmol, 0.2 equiv). The reaction mixture was stirred at 85 ºC for 6 h. The heating was turned off and the mixture was cooled to room temperature. The mixture was then treated with water (15 mL) and washed with DCM (20 mL x 2) to remove excess 3-chloro-1-propanol. The aqueous layer was then collected and evaporated. The crude product was obtained as a dark brown oil. It was then dissolved in MeOH and loaded to SiO_2 (dry loading) for purification. The crude was then purified by chromatography on silica gel using a gradient solvent from pure DCM to up to 10% MeOH/DCM. The desired product was obtained as a white solid (4.63 g, 69%); $R_f = 0.16$ in 10% MeOH/DCM; m.p. 146.0-148.0 °C. ^1H NMR (400 MHz, D_2O) δ 4.85 (d, J =3.6 Hz, 1H), 3.91-3.81 (m, 3H), 3.78-3.66 (m, 5H), 3.59-3.53 (m, 1H), 3.50-3.42 (m, 1H), 2.08-1.99 (m, 5H); ^{13}C NMR (100 MHz, D_2O) δ 174.5, 97.0, 71.9, 71.0, 70.0, 64.6, 60.6, 53.8, 42.1, 31.4, 21.9. LC-MS (ESI+) m/z calcd for $C_{11}H_{21}ClNO_6$ $[M + H]^+$, 298.2, found 298.1.

Synthesis of compound S5:[3,4] To a solution of compound **S4** (5.0 g, 16.79 mmol, 1.0 equiv) in DMF (10.0 mL) was added NaN_3 (3.3 g, 50.38 mmol, 3.0 equiv) and the mixture was heated at 85 °C for 5.0 h. At which point the ^1H NMR spectrum of the reaction mixture indicated the complete consumption of the starting material. DMF was removed under reduced pressure to obtain the crude which was further purified by flash chromatography (DCM to 7% MeOH/DCM) to obtain a white solid (4.2 g, 82%) as the desired product ($R_f = 0.25$ in 10% MeOH/DCM). Compound **S5** was also synthesized in a one-pot reaction from **S1** following the literature.[3] m.p. 125.0-127.0 °C. ^1H NMR (400 MHz, D_2O) δ 4.84 (d, J = 3.6 Hz, 1H), 3.91-3.63 (m, 6H), 3.54-3.40 (m, 4H), 2.01 (s, 3H), 1.91-1.83 (m, 2H); ^{13}C NMR (100 MHz, D_2O) δ 175.0, 97.5, 72.5, 71.6, 70.6, 65.6, 61.2, 54.4, 48.8, 28.6, 22.5; LC-MS (ESI+) m/z calcd for $C_{11}H_{21}N_4O_6$ $[M + H]^+$ 305.1, found 305.1.

Synthesis of compound 6e:[3] To a stirring solution of compound **S5** (1.50 g, 4.91 mmol) in dry pyridine (7 mL) at 0 °C, a solution of TsCl (1.88 g, 9.82 mmol) in pyridine (2 mL) was added dropwise and the mixture was left stirring at rt for 12 h. The reaction was quenched with MeOH (2.0 mL) and the solvent was evaporated. The crude product was purified by chromatography on SiO_2 using eluent from 1% MeOH/DCM to 5% MeOH/DCM to obtain an off-white foam (1.47 g, 65%) as the desired product ($R_f = 0.32$ in 5% MeOH/DCM). ^1H NMR (400 MHz, $CDCl_3$) 7.81 (d,

J = 8.3 Hz, 2H), 7.36 (d, J = 8.2 Hz, 2H), 6.49 (d, J = 8.8 Hz, 1H), 4.73 (d, J = 3.5 Hz, 1 H), 4.35-4.29 (m, 2H), 4.02 (m, 1H), 3.85-3.67 (m, 5H), 3.53-3.34 (m, 4H), 2.45 (s, 3H), 2.04 (s, 3H), 1.86 (pentet, J = 6.2 Hz, 2H); ^{13}C NMR (400 MHz, $CDCl_3$) 172.1, 145.0, 132.8, 129.9, 128.0, 97.5, 73.1, 70.4, 69.9, 69.4, 65.1, 53.5, 48.6, 28.5, 23.1, 21.6. LC-MS (ESI+) m/z calcd for $C_{18}H_{27}N_4O_8S$ $[M + H]^+$ 459, found 459.

Synthesis of compound 9:[3] To a 50 mL RBF, compound **6e** (1.0 g, 2.18 mmol, 1.0 equiv), DCM (8.0 mL), pyridine (0.88 mL, 10.90 mmol, 5.0 equiv) were added in the given order and the mixture was cooled to 0 °C. To the stirring mixture benzoyl chloride (0.63 mL, 5.45 mmol, 2.5 equiv) was added dropwise. The mixture was left stirring at room temperature for additional 8 hours. ^{1}H NMR spectrum indicated the completion of the reaction. Work up was performed with H_2O (40 mL) and DCM (50 mL x 3). The combined organic layer was dried over Na_2SO_4 (anhydrous), filtered and concentrated to afford the crude, which was purified by column chromatography using eluent from DCM to 2% MeOH/DCM to give a colorless oil (1.13 g, 78%) as the desired product (R_f = 0.65 in 5% MeOH/DCM). Compound **9** was also synthesized using a one-pot reaction from compound **S5** directly following the literature procedure. ^{1}H NMR ($CDCl_3$, 400 MHz) δ 7.90-7.83 (m, 4H), 7.71 (d, J = 8.3 Hz, 2H), 7.55-7.48 (m, 2H), 7.40-7.31 (m, 4H), 7.22 (d, J = 8.1 Hz, 2H), 5.89 (d, J = 9.2 Hz, 1H), 5.59 (dd, J = 10.9, 9.5 Hz, 1H), 5.35 (t, J = 9.5 Hz, 1H), 4.90 (d, J = 3.6 Hz, 1H), 4.48 (ddd, J = 10.9, 9.2, 3.6 Hz, 1H), 4.25-4.10 (m, 3H), 3.87 (dt, J = 10.1, 6.0 Hz, 1H), 3.59-3.41 (m, 3H), 2.37 (s, 3H), 2.00-1.92 (m, 2H), 1.85 (s, 3H); ^{13}C NMR ($CDCl_3$, 100 MHz) δ 169.9, 167.1, 165.1, 145.0, 133.53, 133.49, 132.5, 129.84, 129.78, 129.76, 128.7, 128.6, 128.43, 128.42, 128.0, 97.3, 71.4, 68.9, 68.4, 68.2, 65.6, 52.2, 48.6, 28.6, 23.1, 21.6. LC-MS (ESI+) m/z $C_{32}H_{35}N_4O_{10}S$ $[M + H]^+$ 667.2, found 667.2.

Cyclization Reactions for LM28 and DLM28

Compound **8a** macrolactonization was also carried at more concentrated conditions in an attempt to obtain more dimer. However, the formation of the monomer macrolactone is preferred.

Entry	**8a** (mg)	Concentration (mM)	Base 2.0 equiv	DMF (mL)	Temp (°C)	Yield % Mono	Yield % Di
1	100	15.8	K_2CO_3	7.0	75	73	20
2	100	22.2	K_2CO_3	5.0	75	68	23

II. ^{1}H and ^{13}C Spectra for Compounds Synthesized (400MHz Bruker)

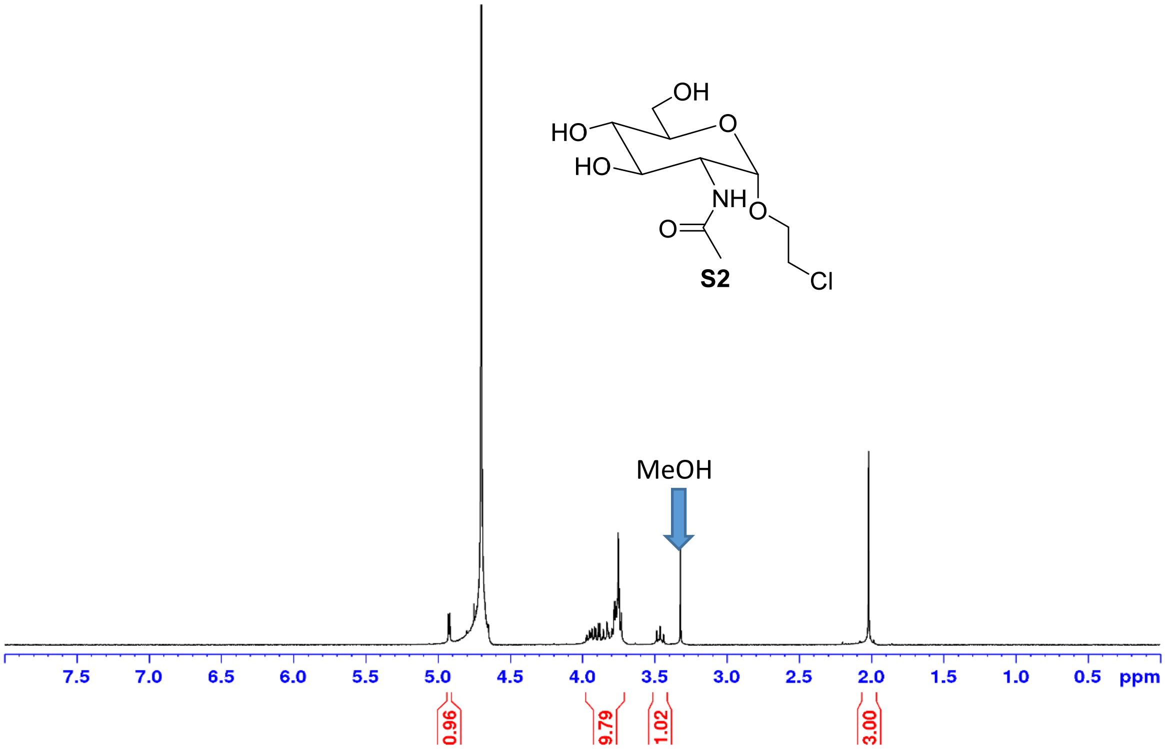

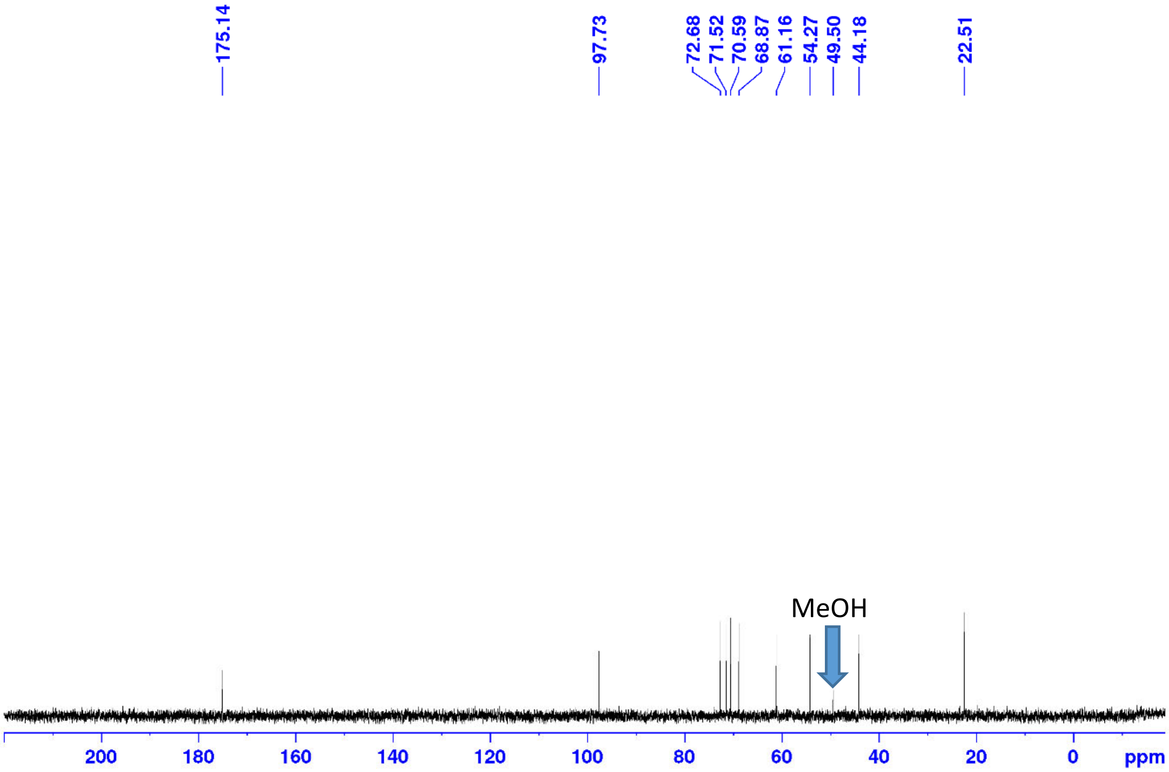

^{1}H NMR and ^{13}C NMR spectra of compound **S2** in D_2O and MeOH (1 drop)

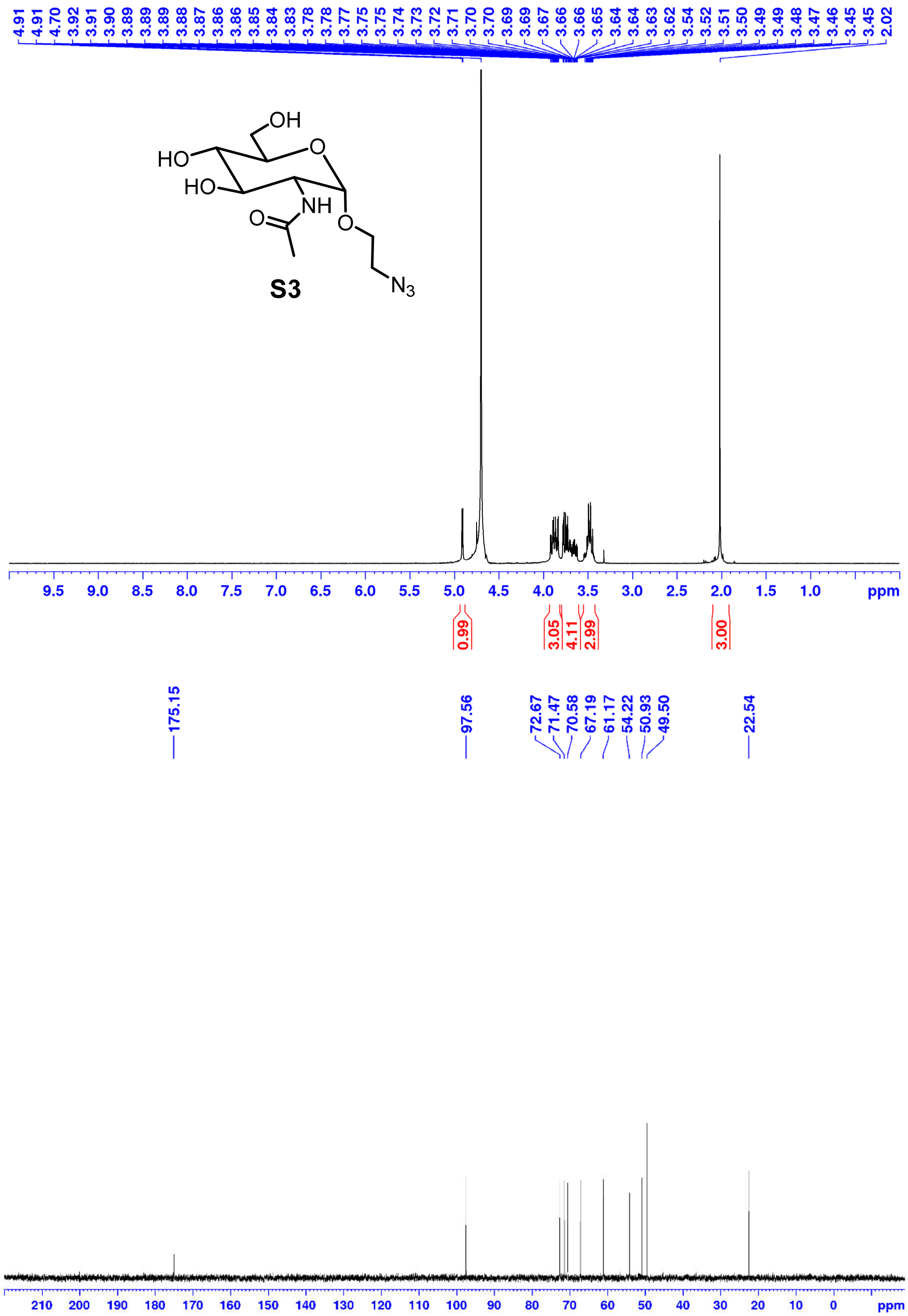

^{1}H NMR and ^{13}C NMR spectra of compound **S3** in D_2O and MeOH (1 drop)

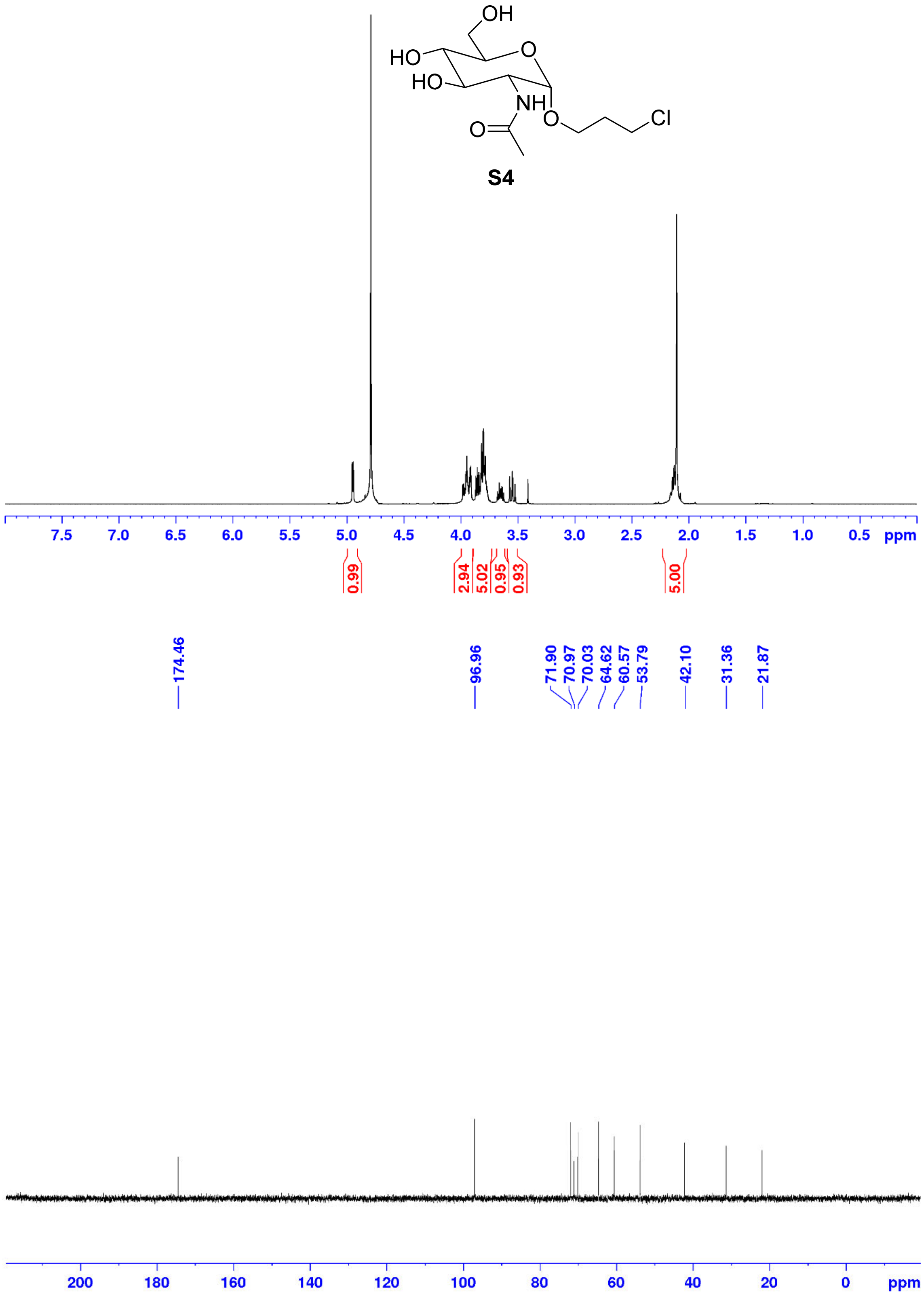

^{1}H NMR and ^{13}C NMR spectra of compound **S4** in D_2O

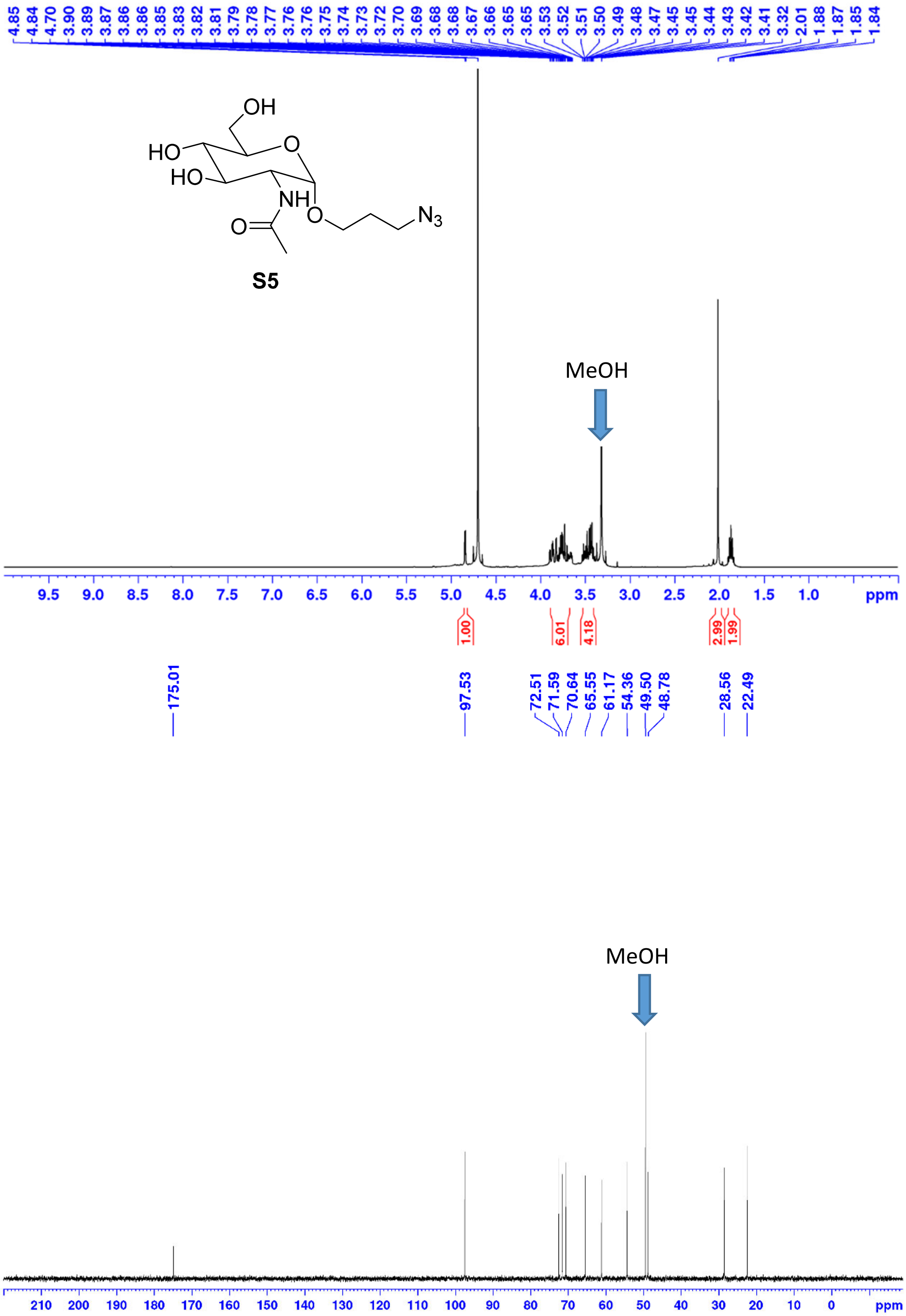

^{1}H NMR and ^{13}C NMR spectra of compound **S5** in D_2O and MeOH (1 drop)

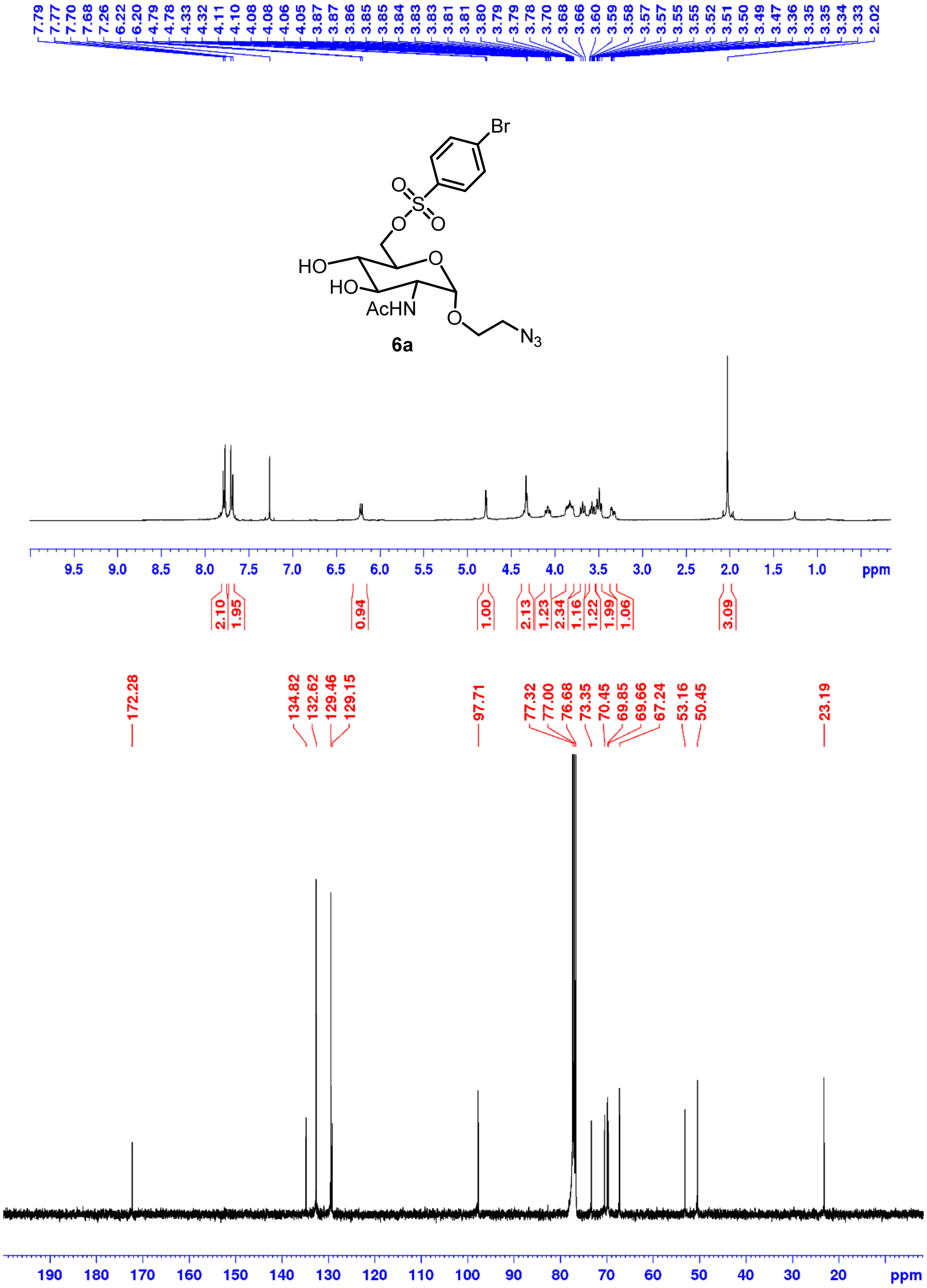

^{1}H NMR and ^{13}C NMR spectra of compound **6a** in $CDCl_3$

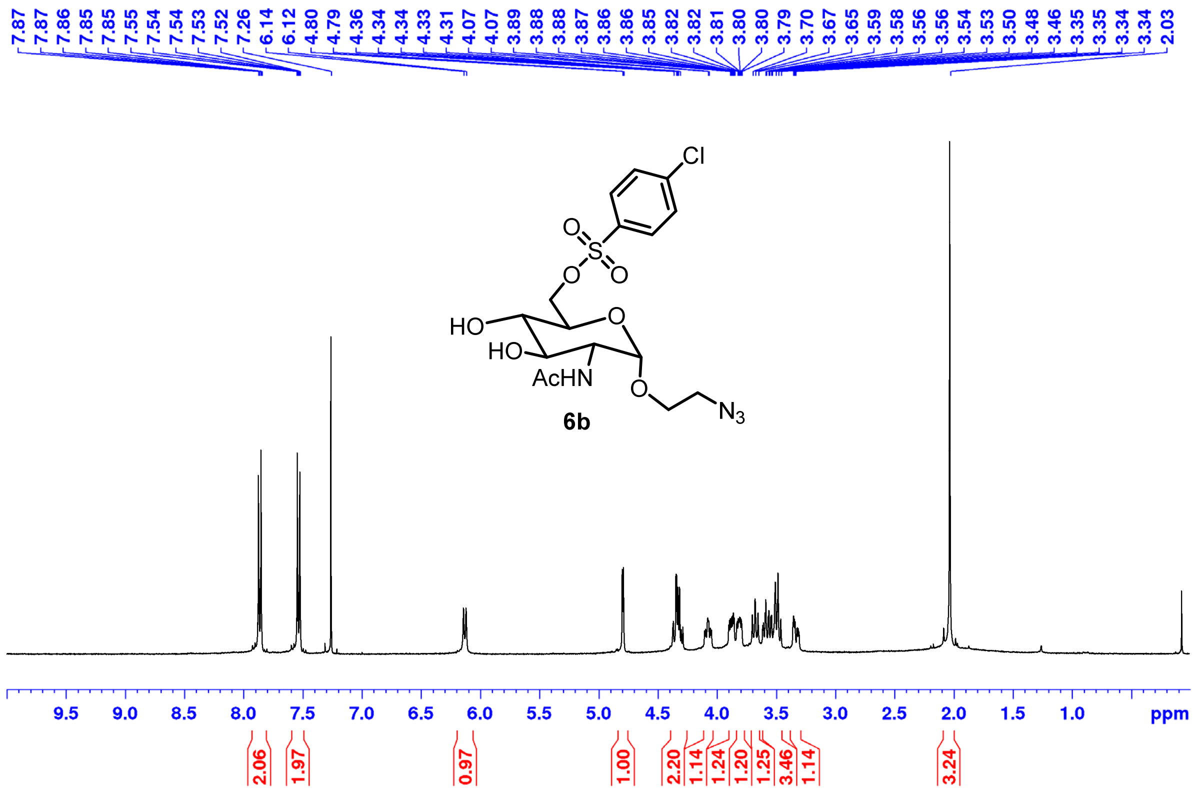

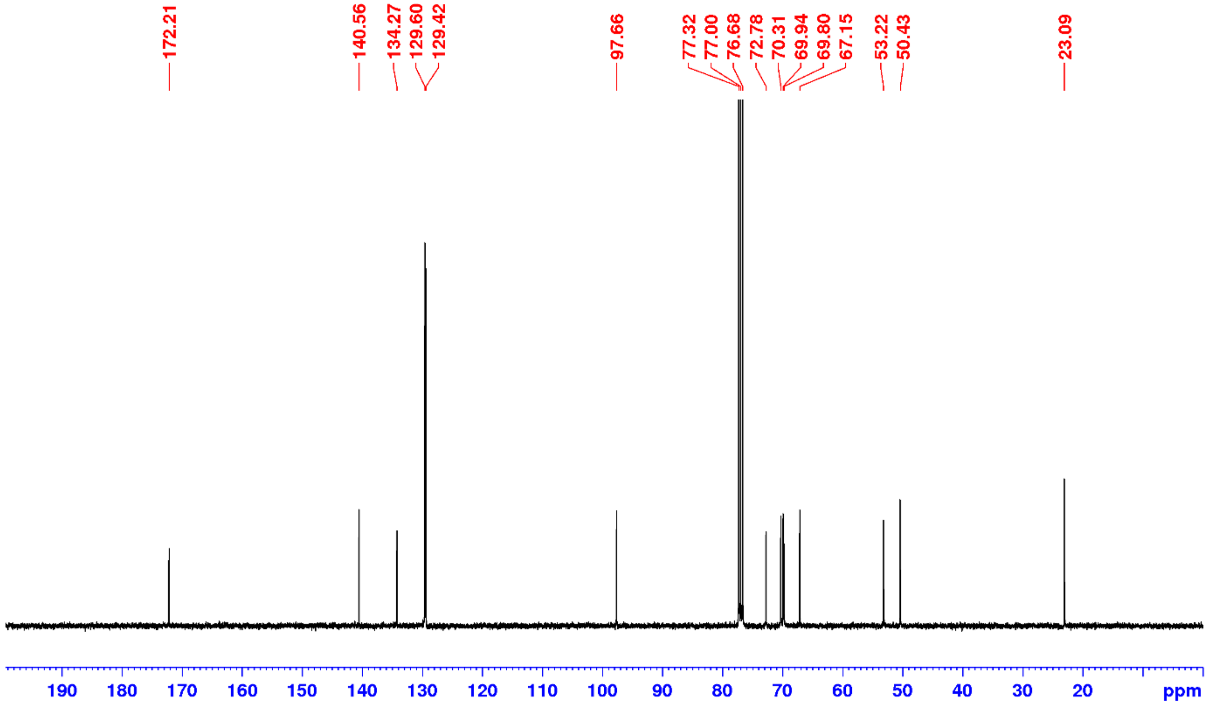

^{1}H NMR and ^{13}C NMR spectra of compound **6b** in $CDCl_3$

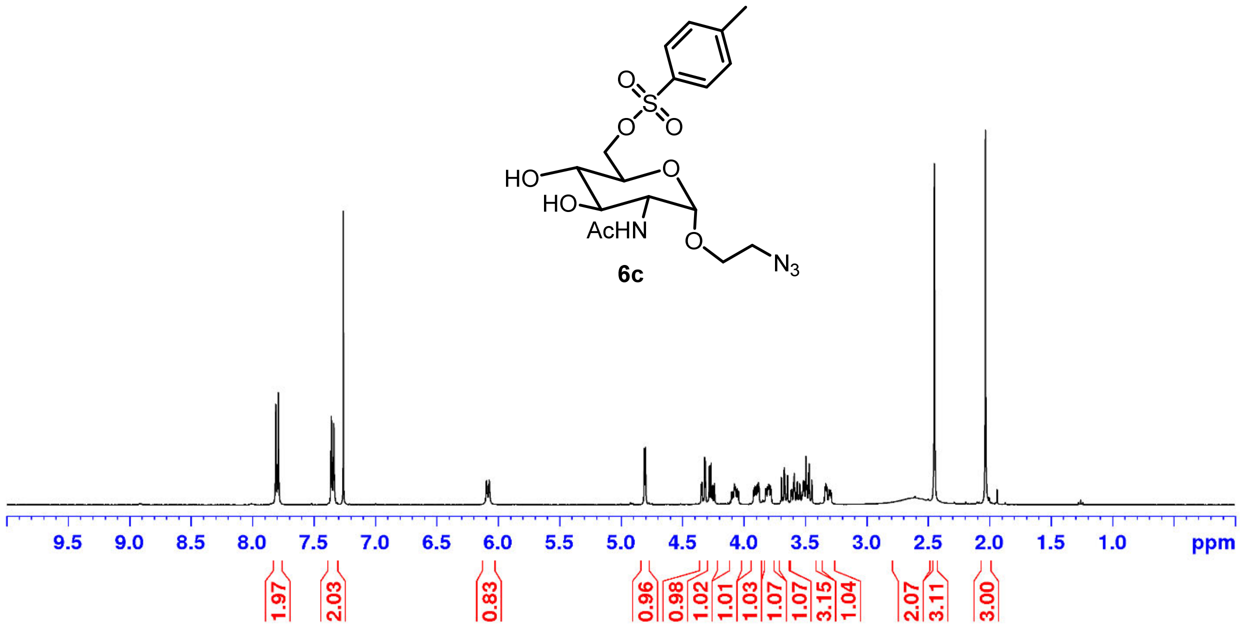

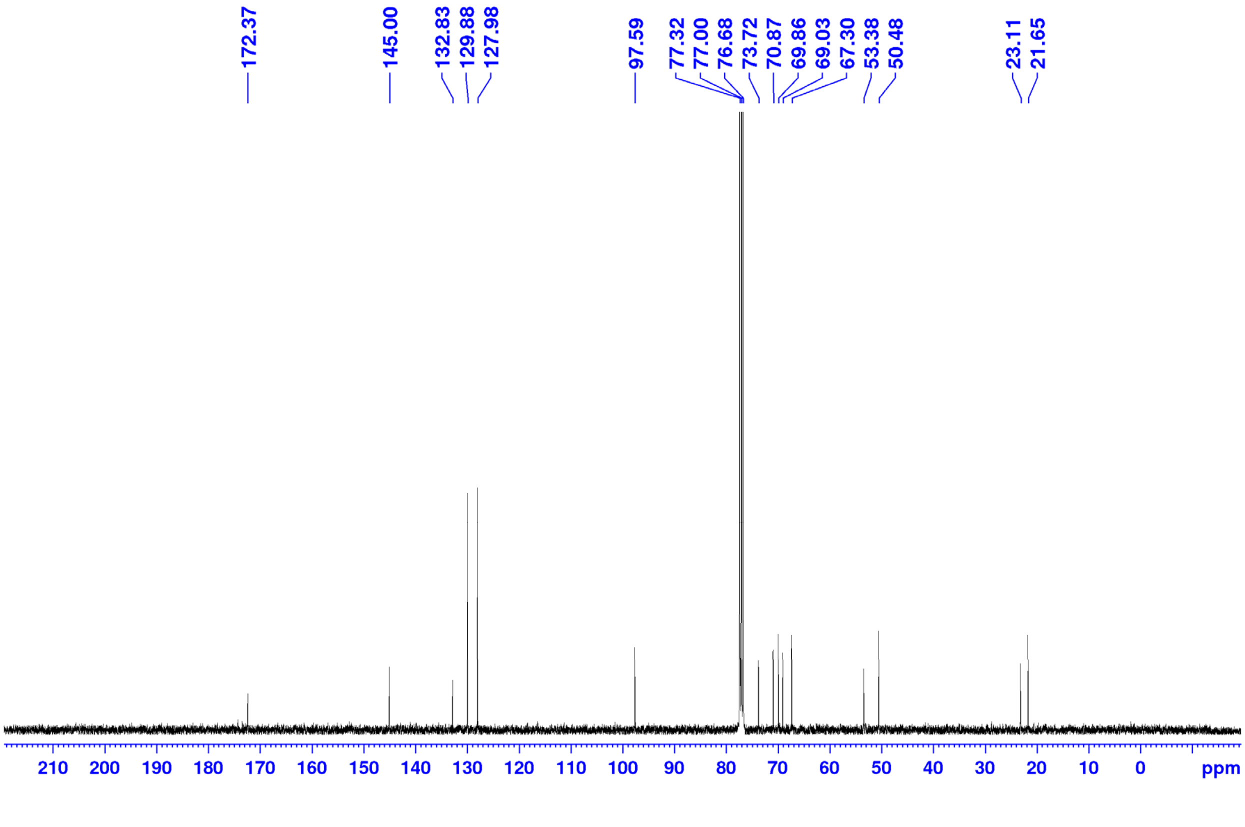

^{1}H NMR and ^{13}C NMR spectra of compound **6c** in $CDCl_3$

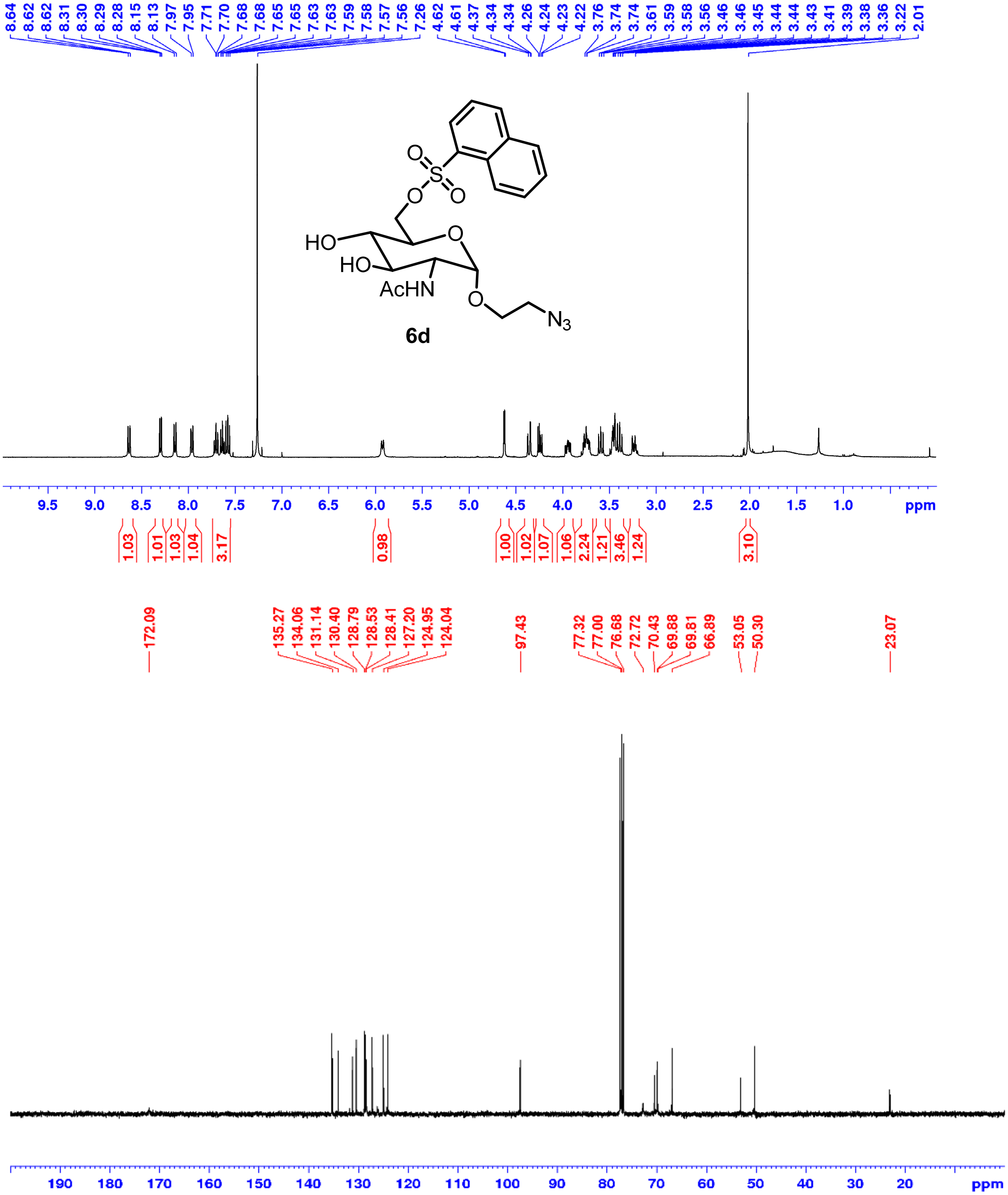

^{1}H NMR and ^{13}C NMR spectra of compound **6d** in $CDCl_3$

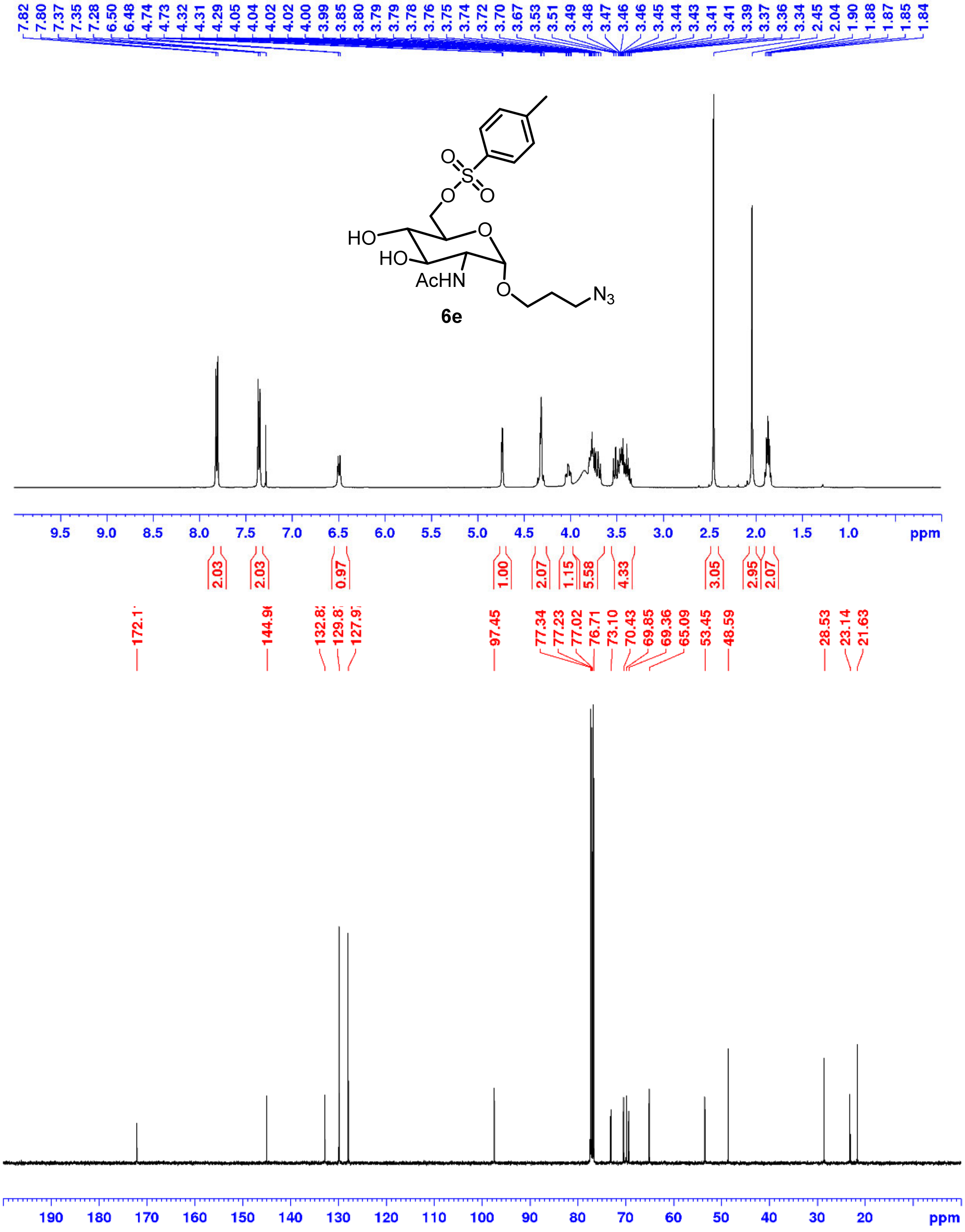

^{1}H NMR and ^{13}C NMR spectra of compound **6e** in $CDCl_3$

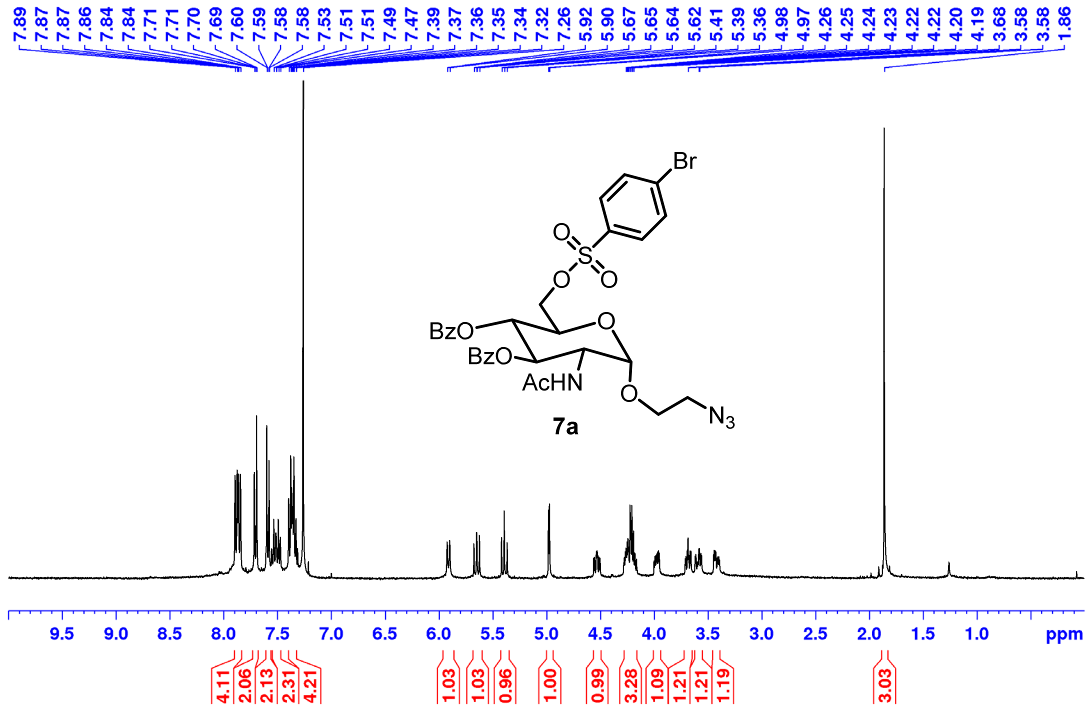

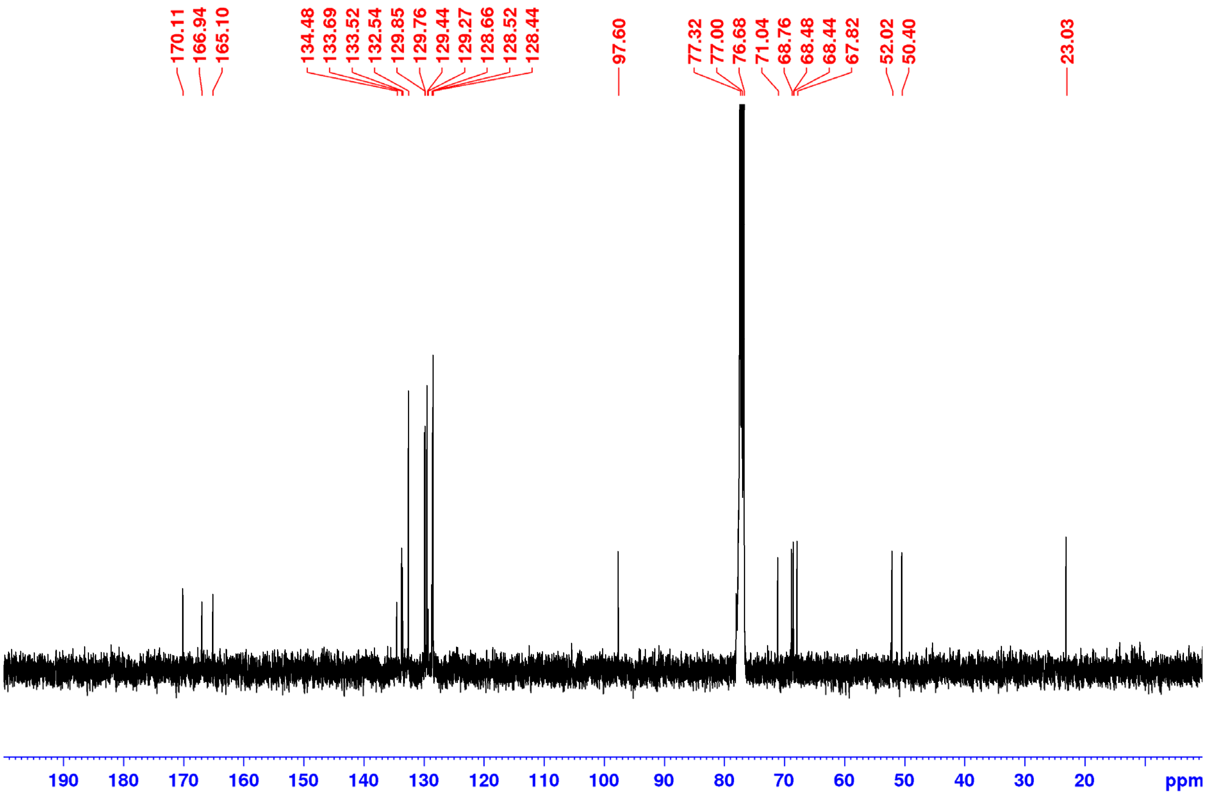

^{1}H NMR and ^{13}C NMR spectra of compound **7a** in $CDCl_3$

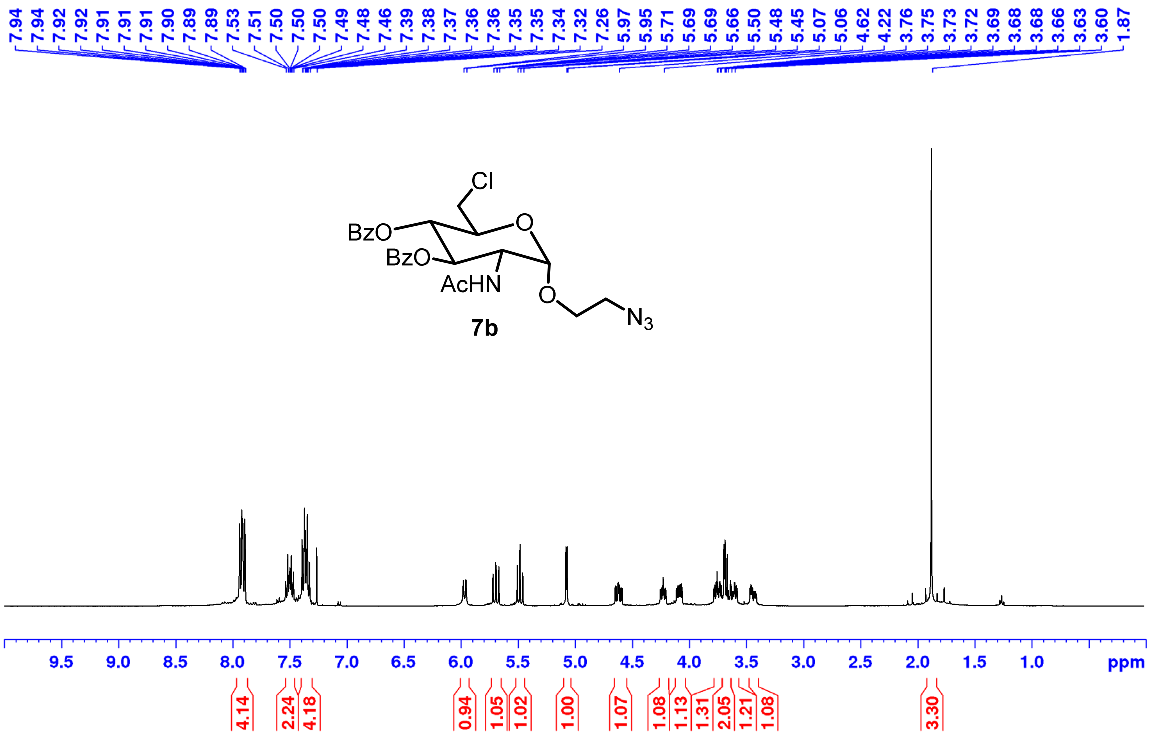

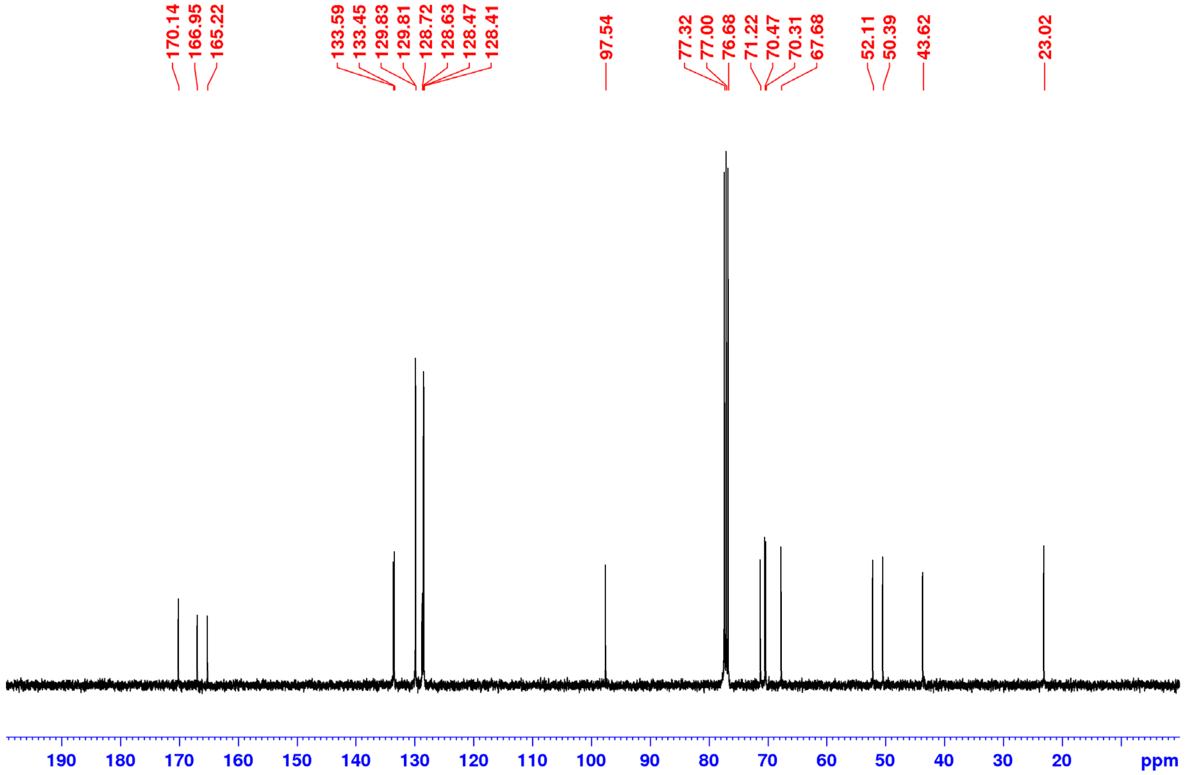

^{1}H NMR and ^{13}C NMR spectra of compound **7b** in $CDCl_3$

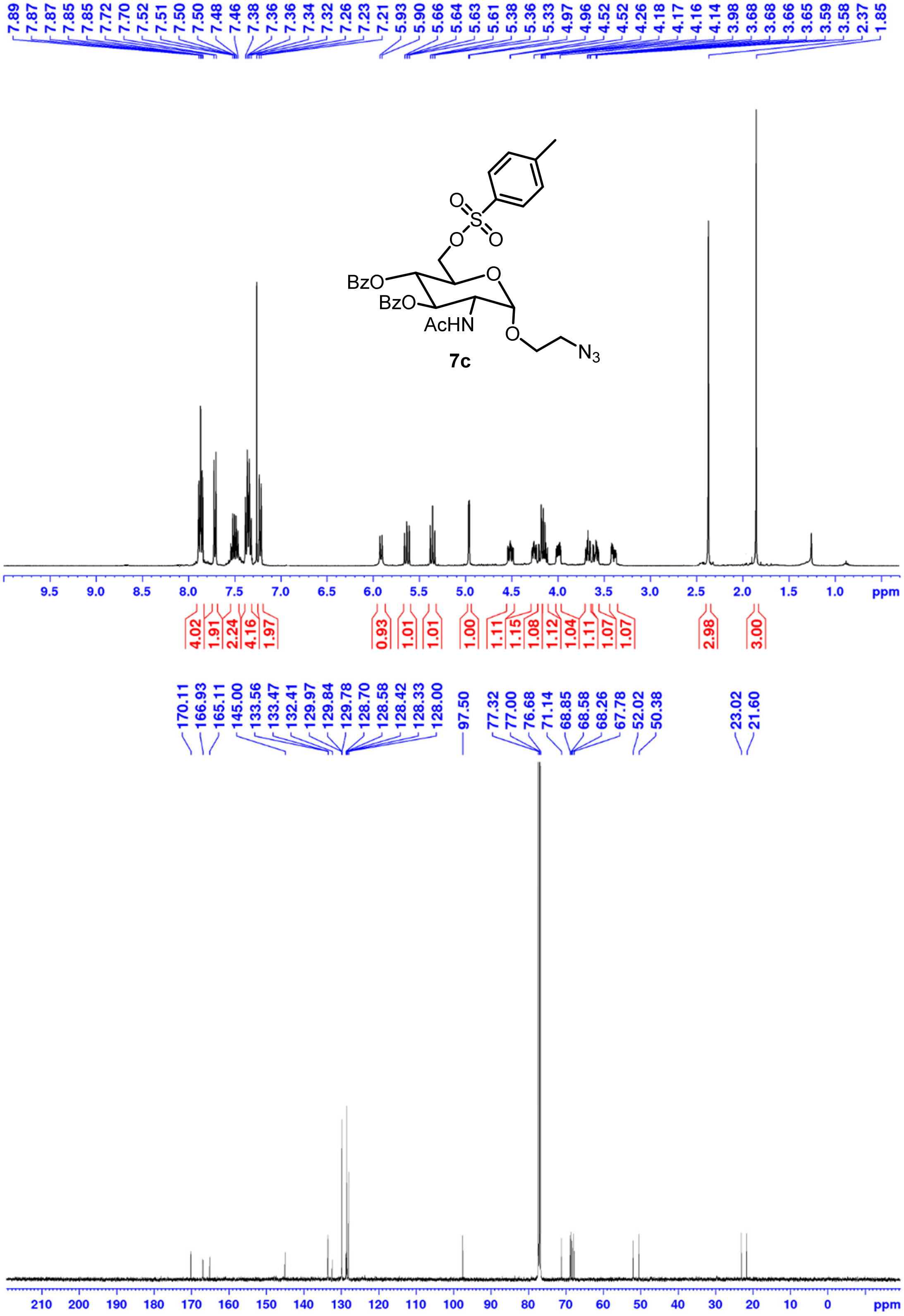

^{1}H NMR and ^{13}C NMR spectra of compound **7c** in $CDCl_3$

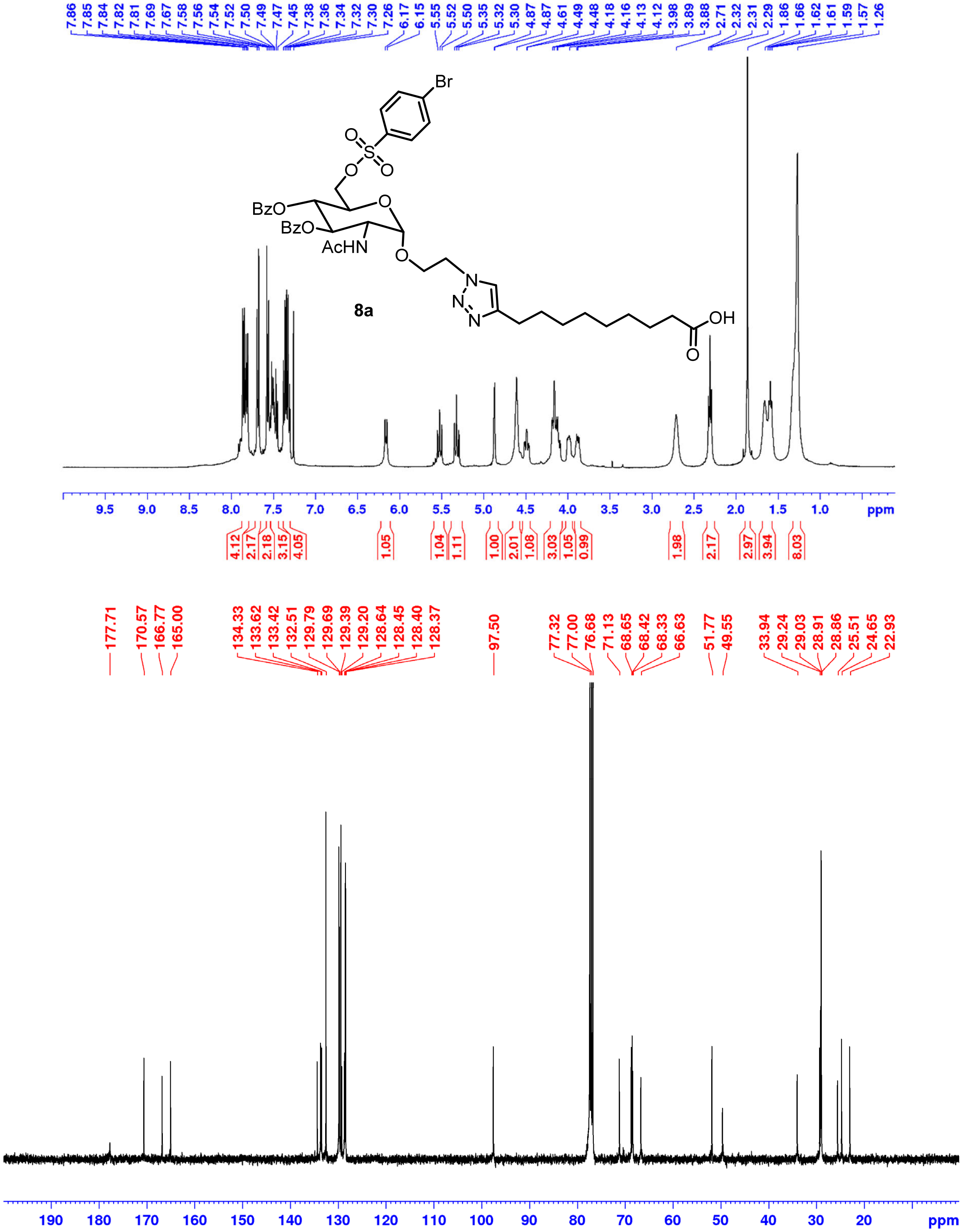

^{1}H NMR and ^{13}C NMR spectra of compound **8a** in $CDCl_3$

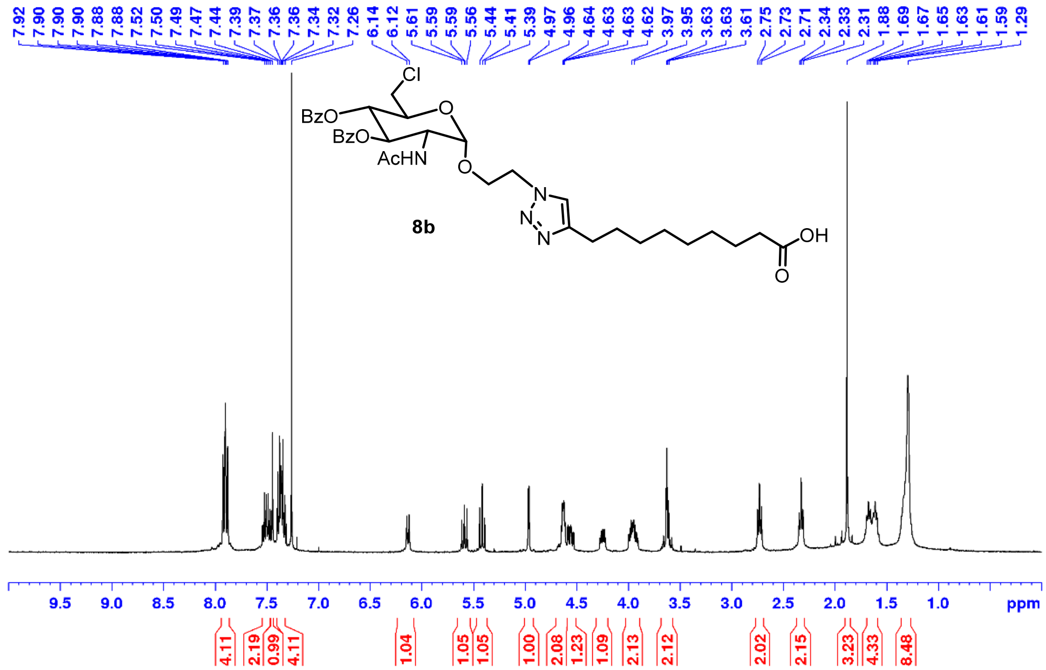

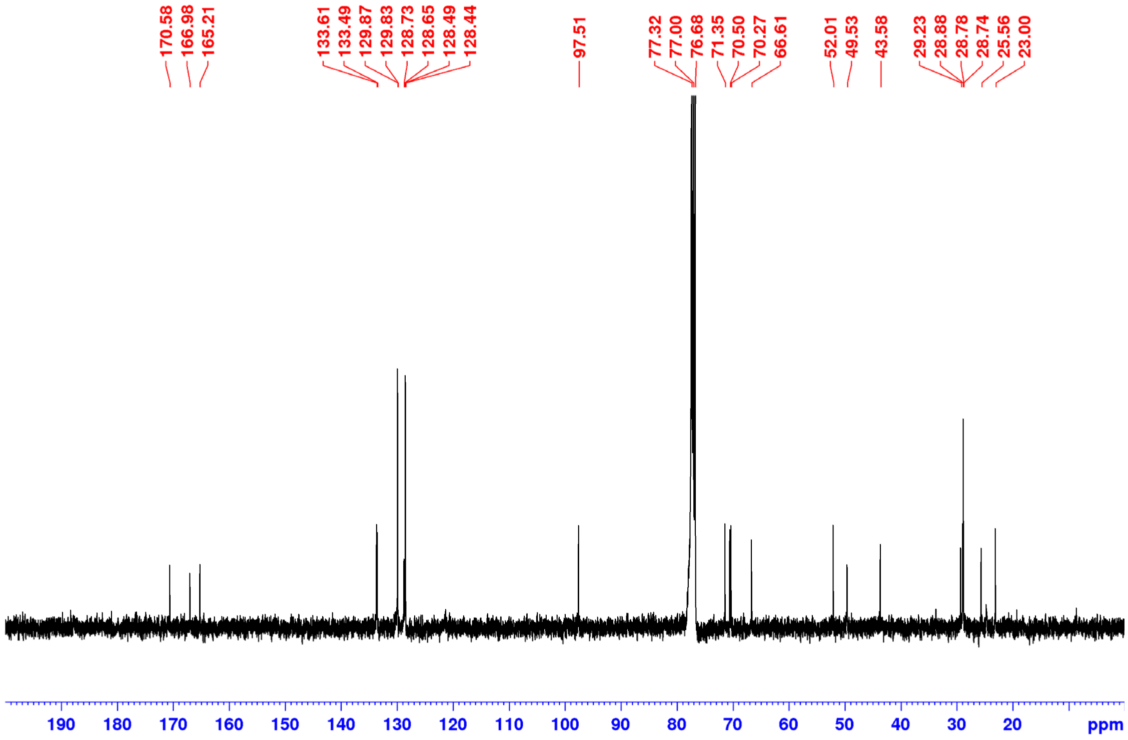

^{1}H NMR and ^{13}C NMR spectra of compound **8b** in $CDCl_3$

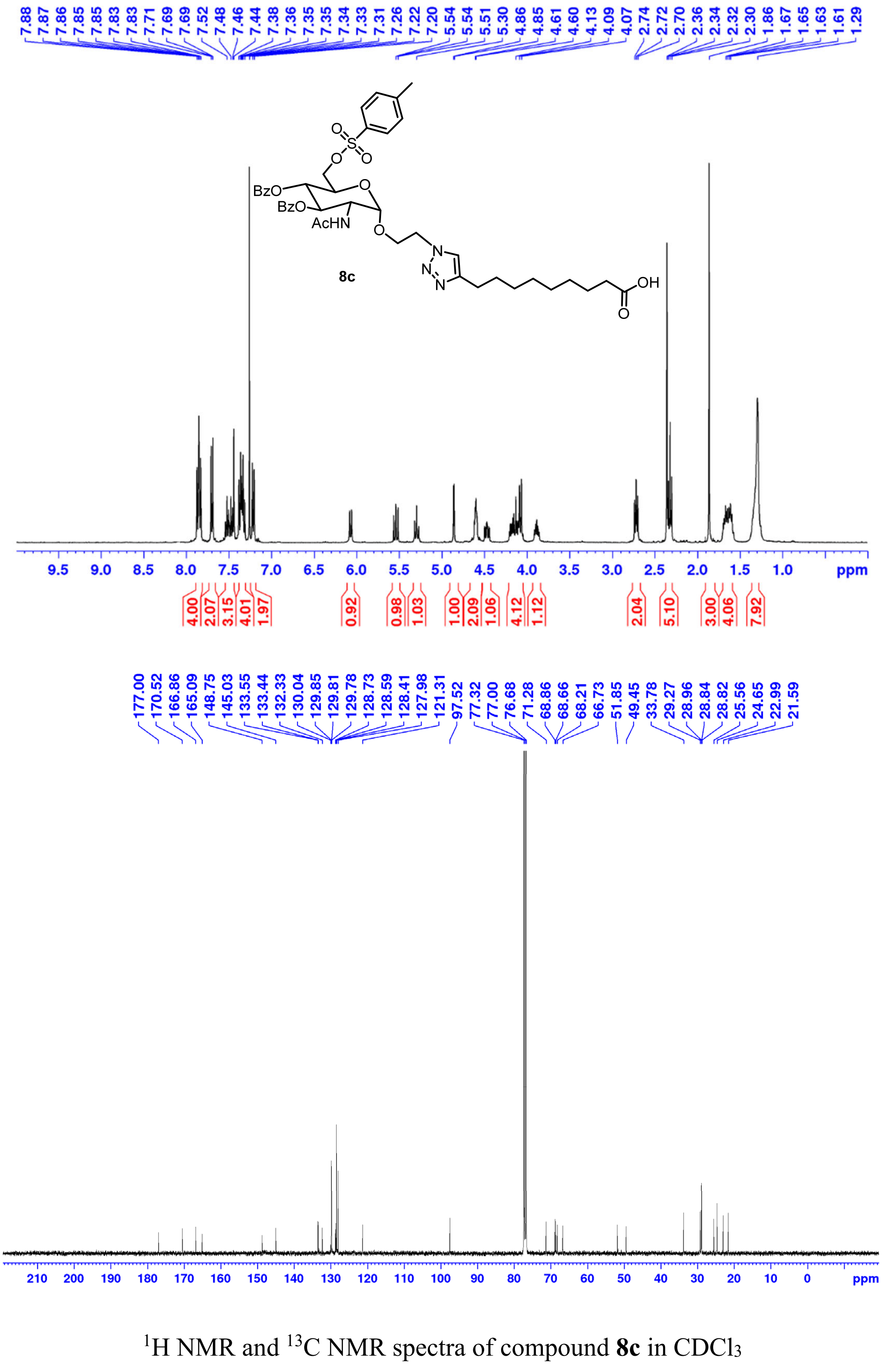

^{1}H NMR and ^{13}C NMR spectra of compound **8c** in $CDCl_3$

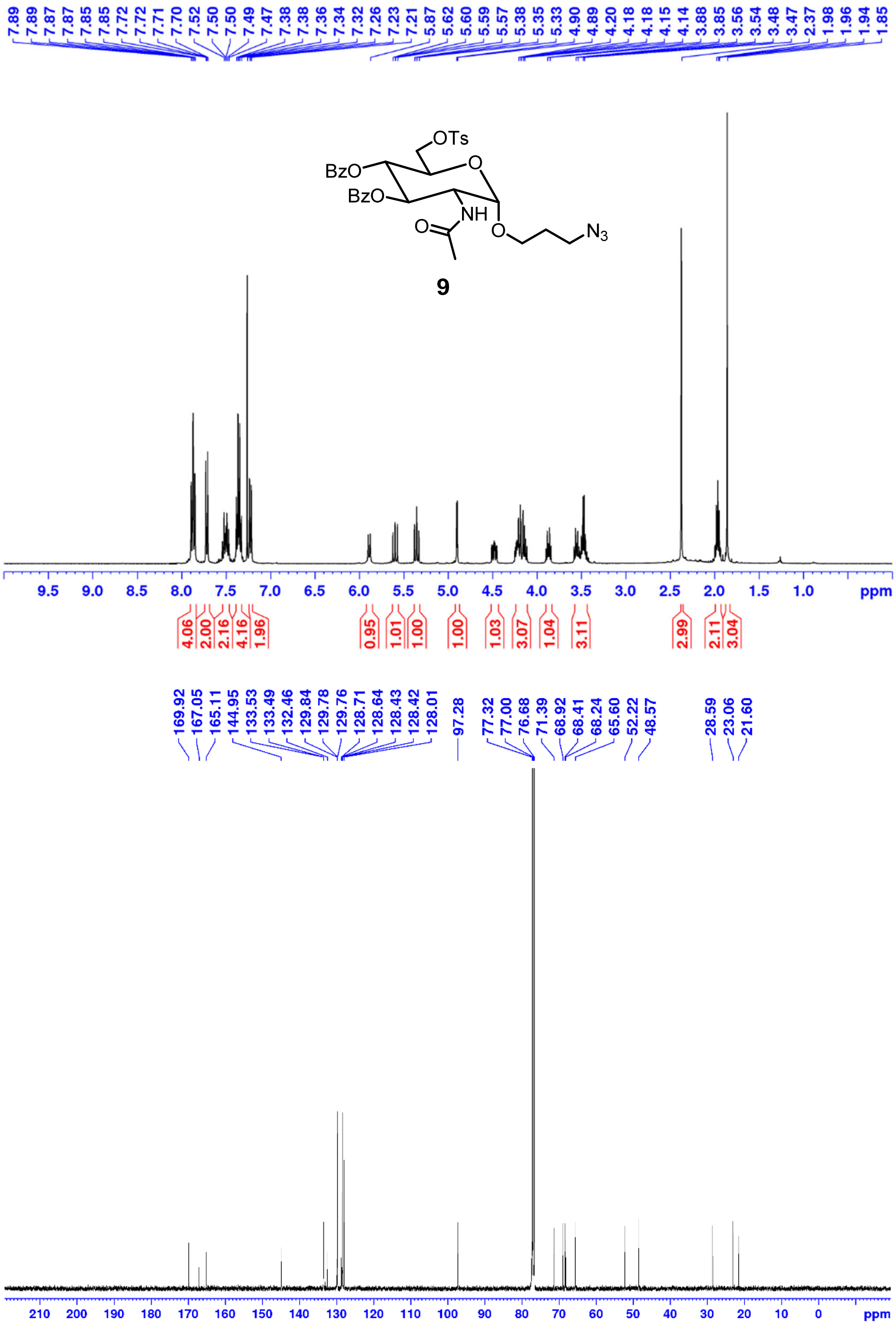

^{1}H NMR and ^{13}C NMR spectra of compound **9** in $CDCl_3$

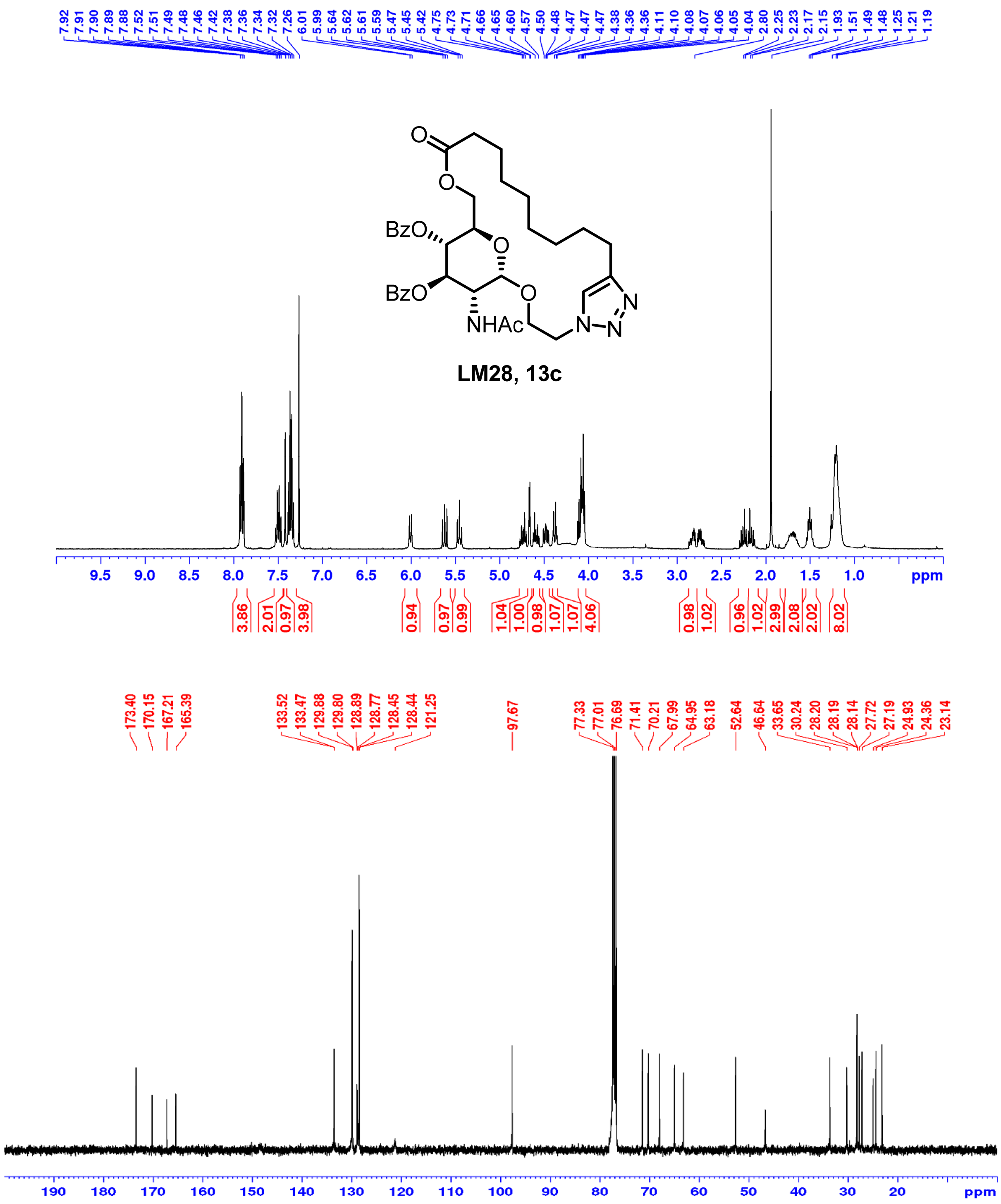

^{1}H NMR and ^{13}C NMR spectra of compound **LM28, 13c** in $CDCl_3$

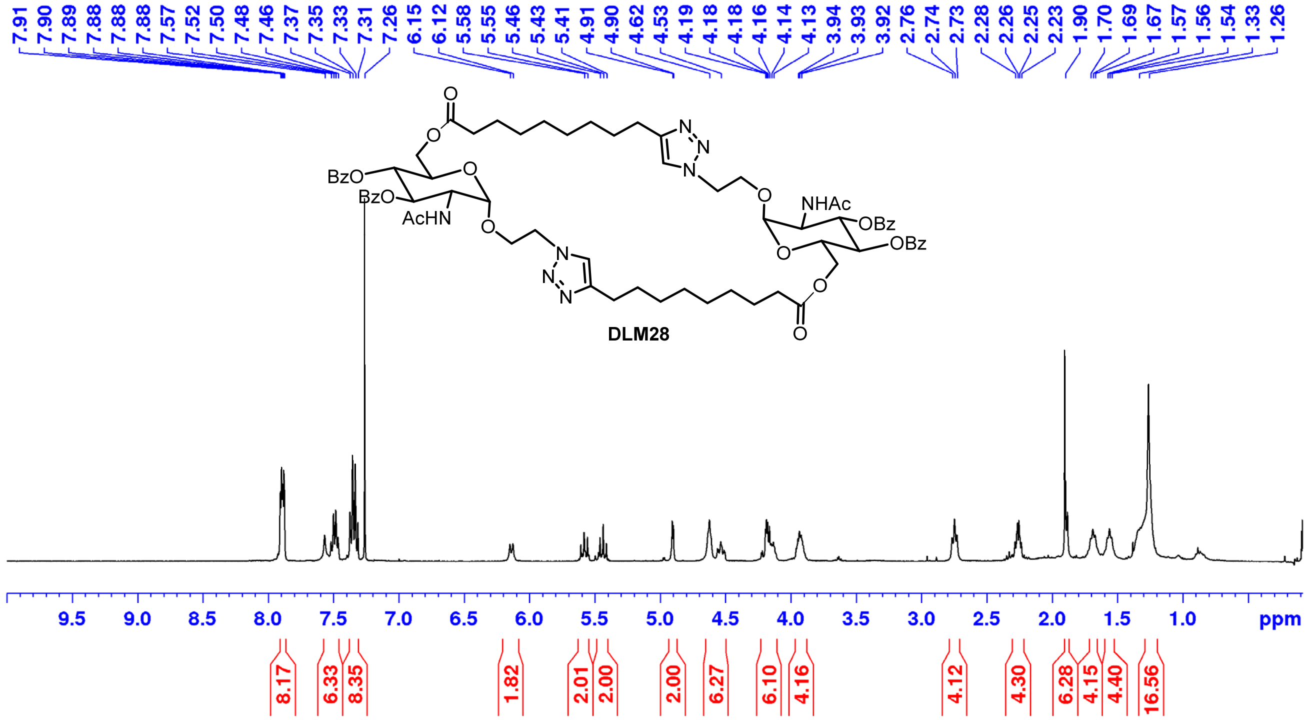

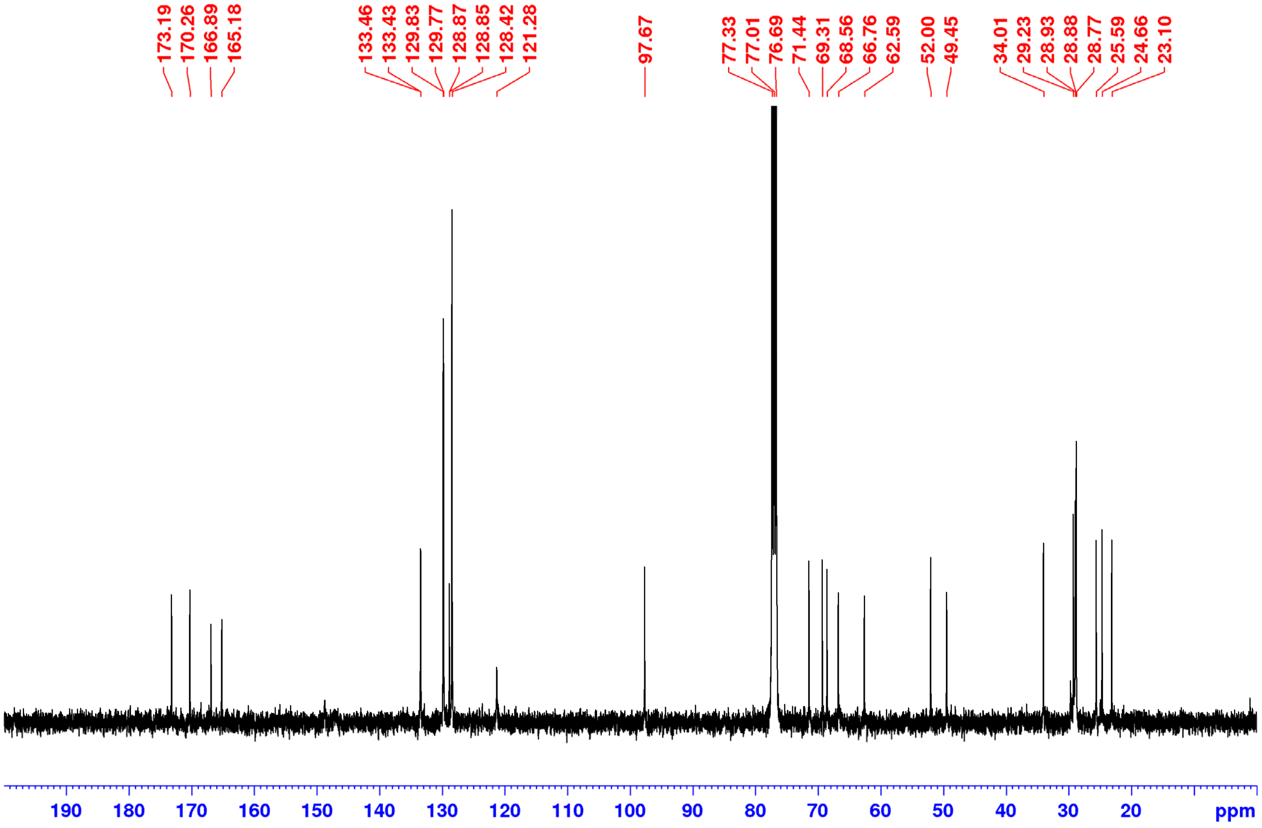

^{1}H NMR and ^{13}C NMR spectra of compound **DLM28** in $CDCl_3$

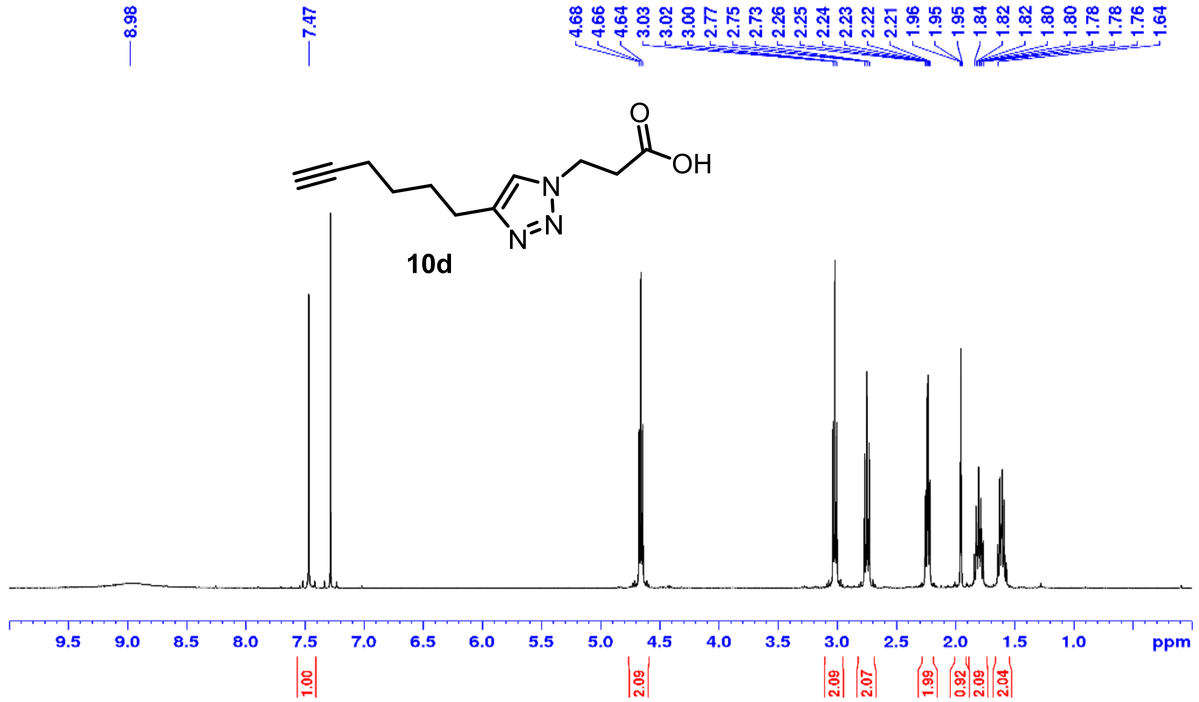

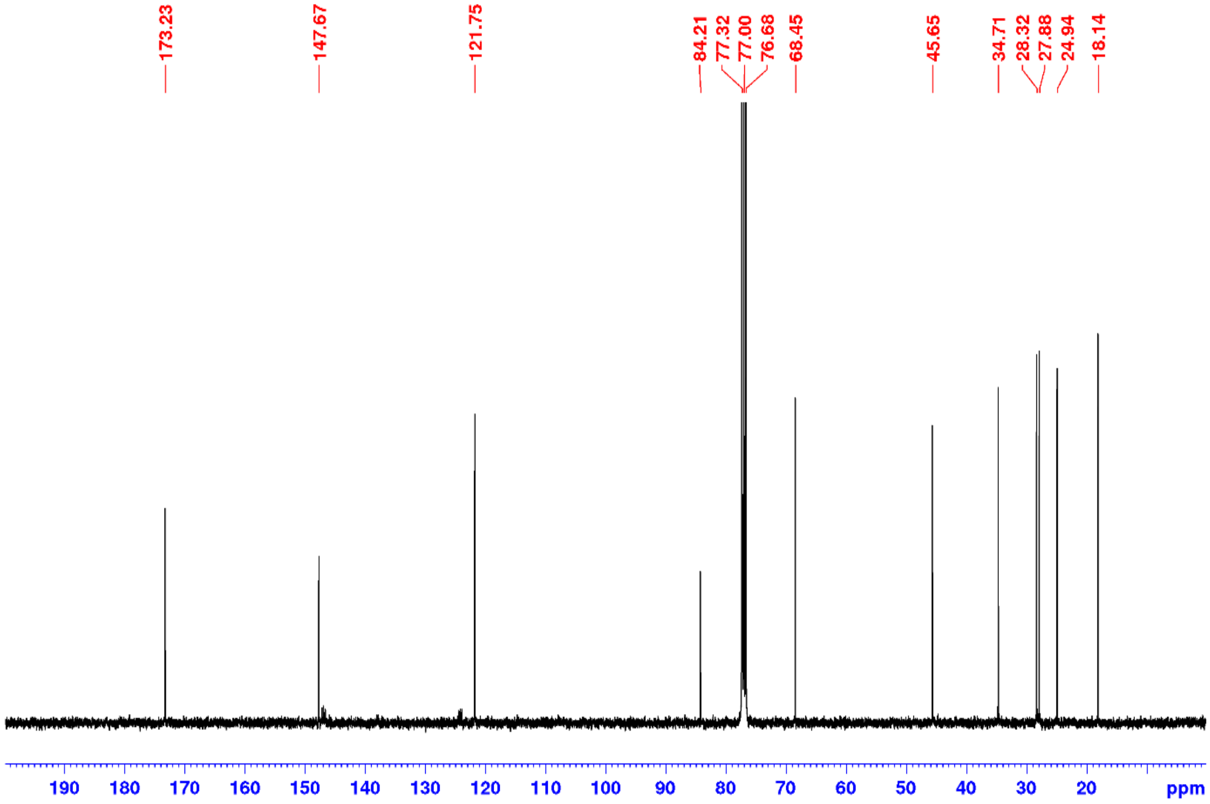

^{1}H NMR and ^{13}C NMR spectra of compound **10d** in $CDCl_3$

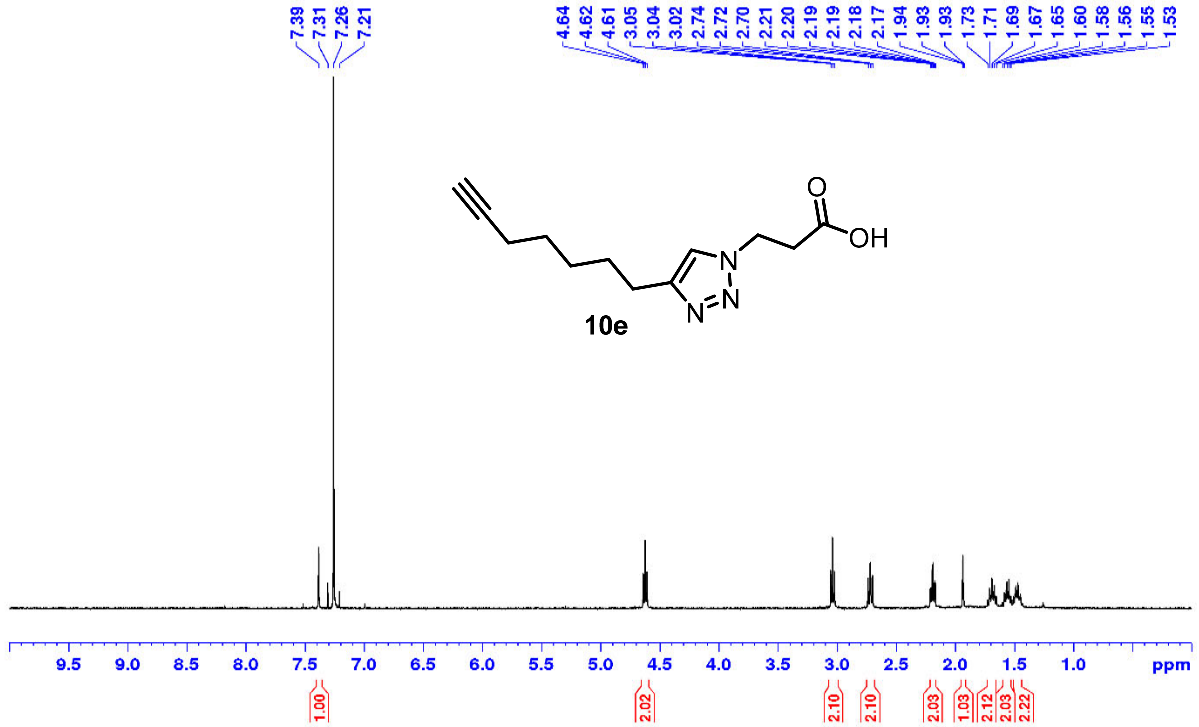

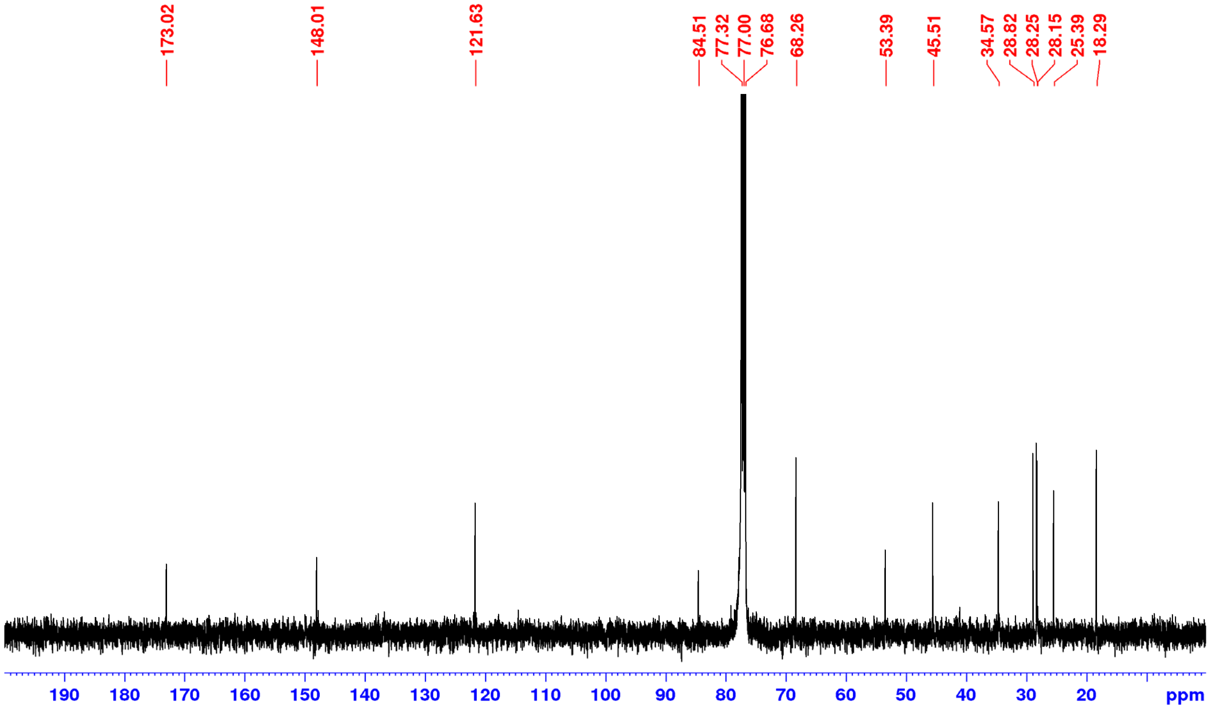

^{1}H NMR and ^{13}C NMR spectra of compound **10e** in $CDCl_3$

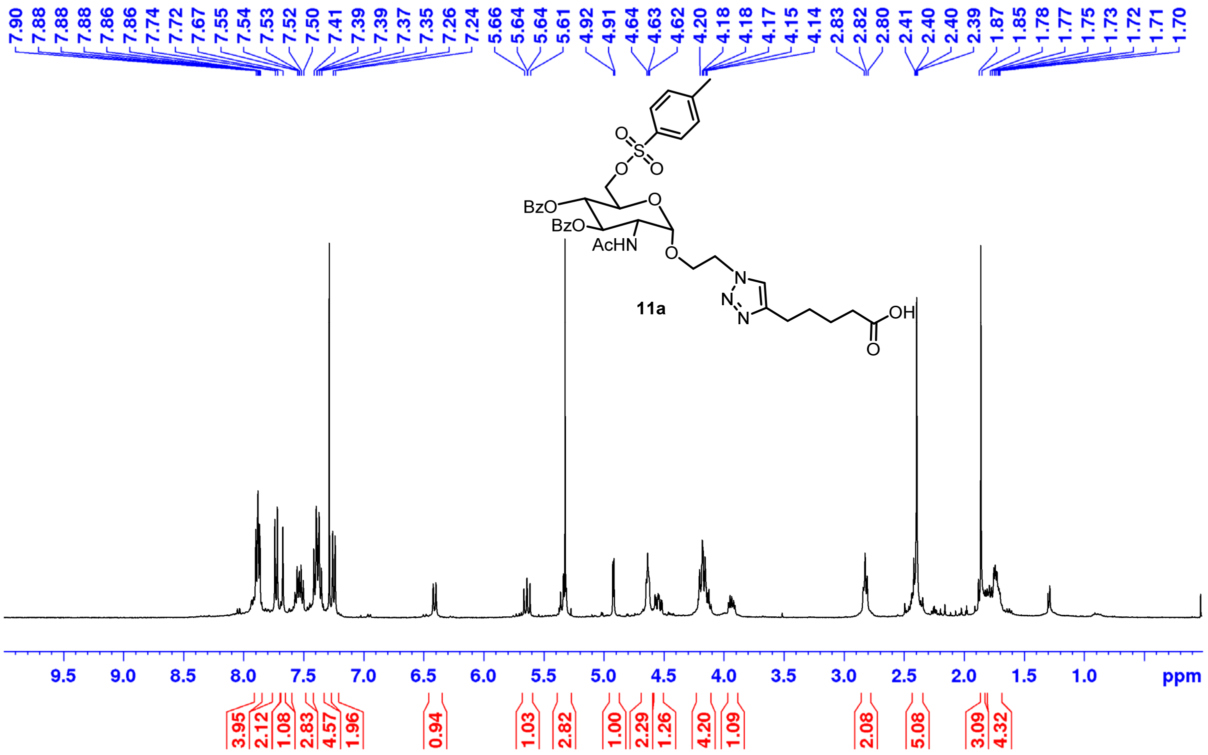

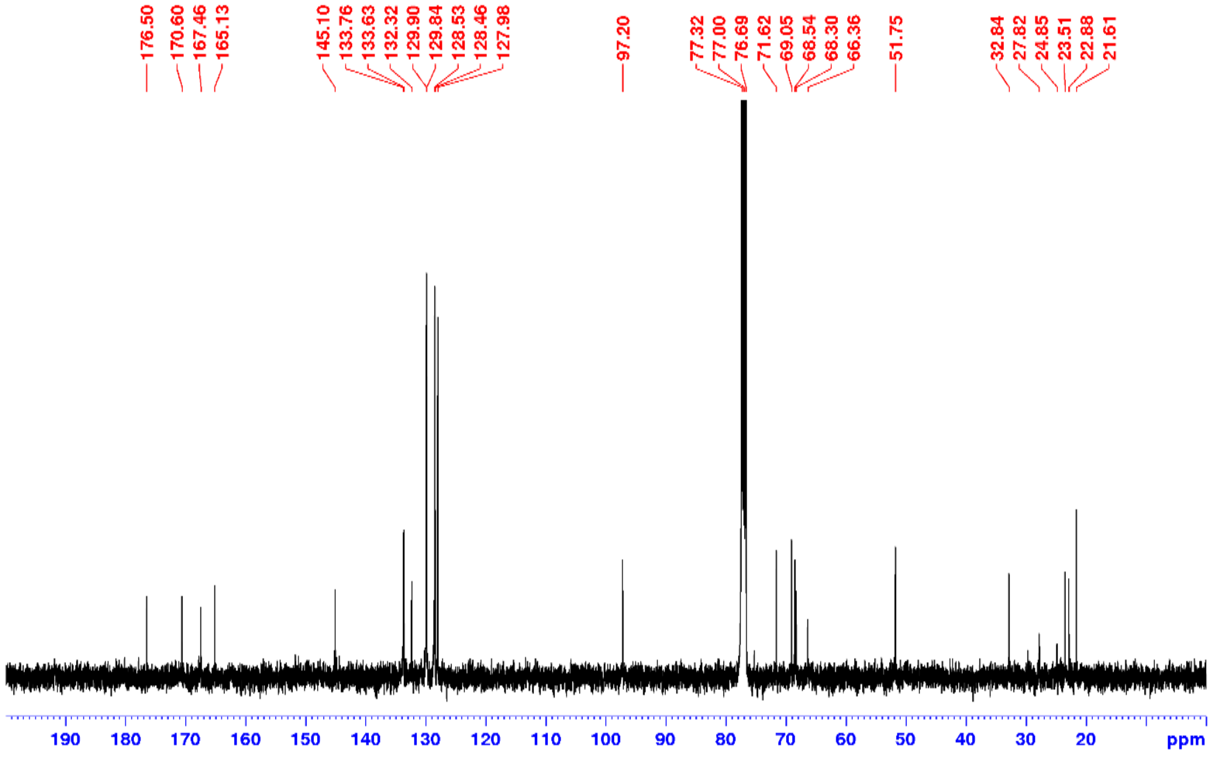

^{1}H NMR and ^{13}C NMR spectra of compound **11a** in $CDCl_3$

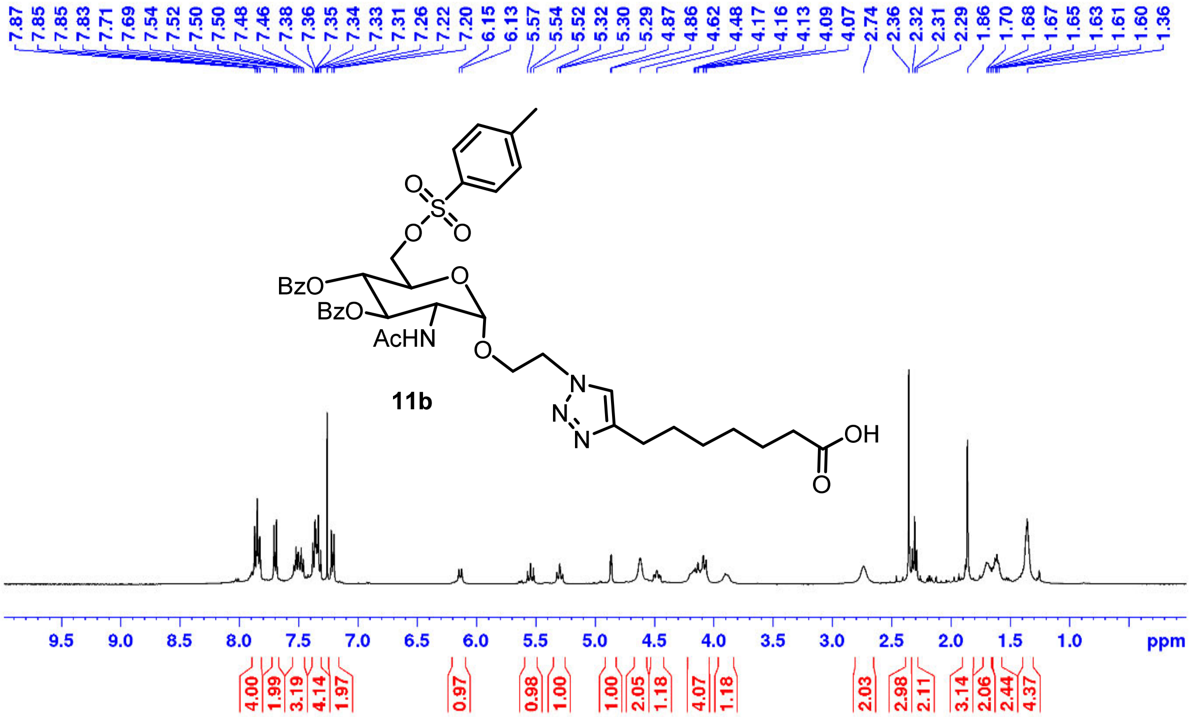

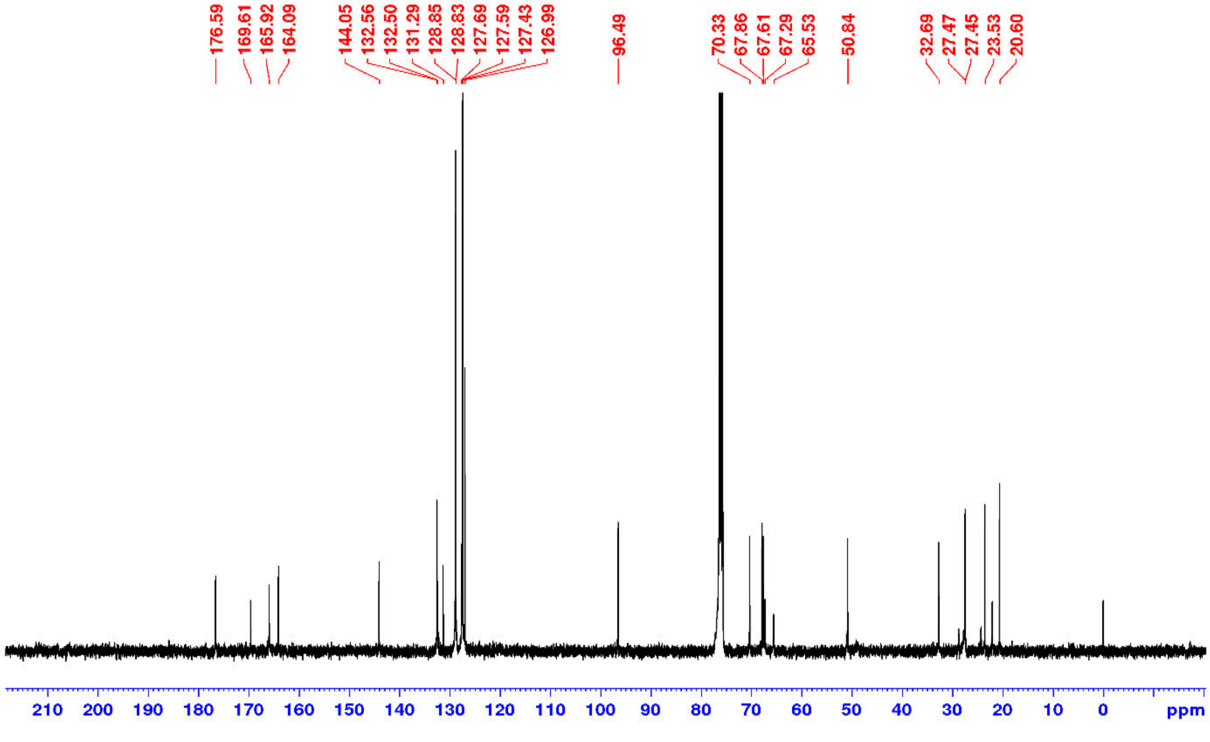

^{1}H NMR and ^{13}C NMR spectra of compound **11b** in $CDCl_3$

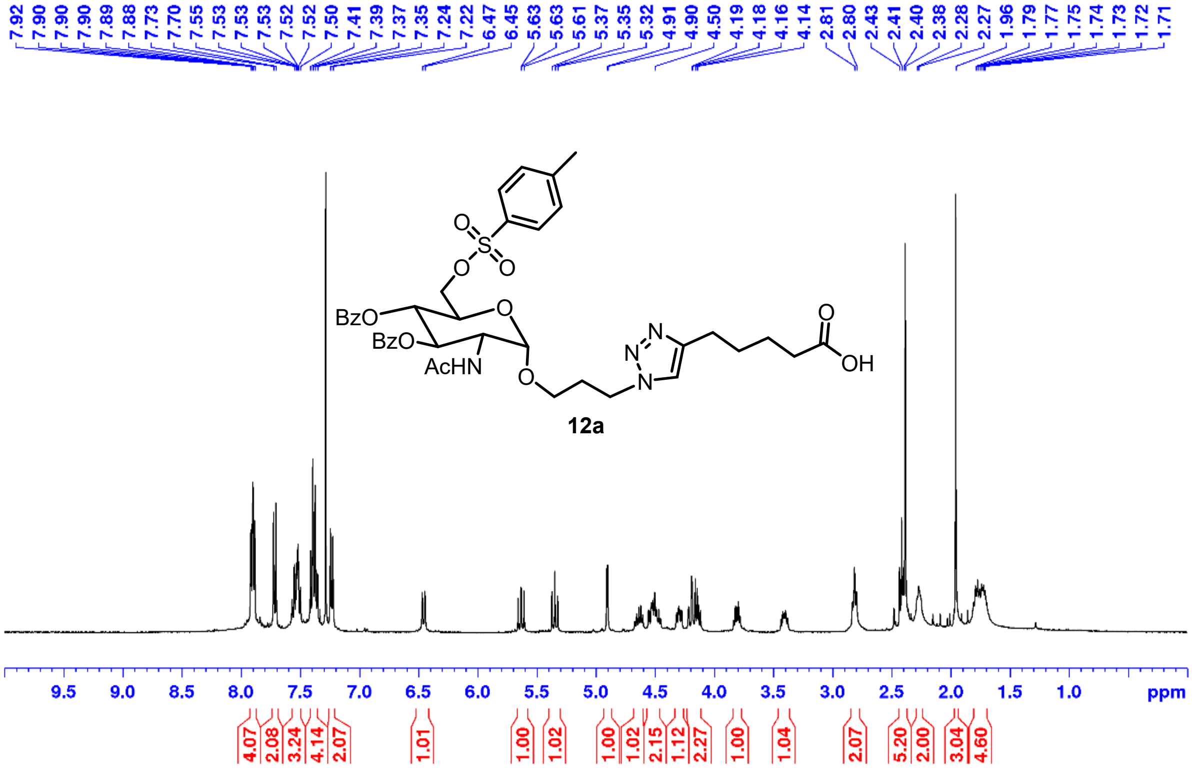

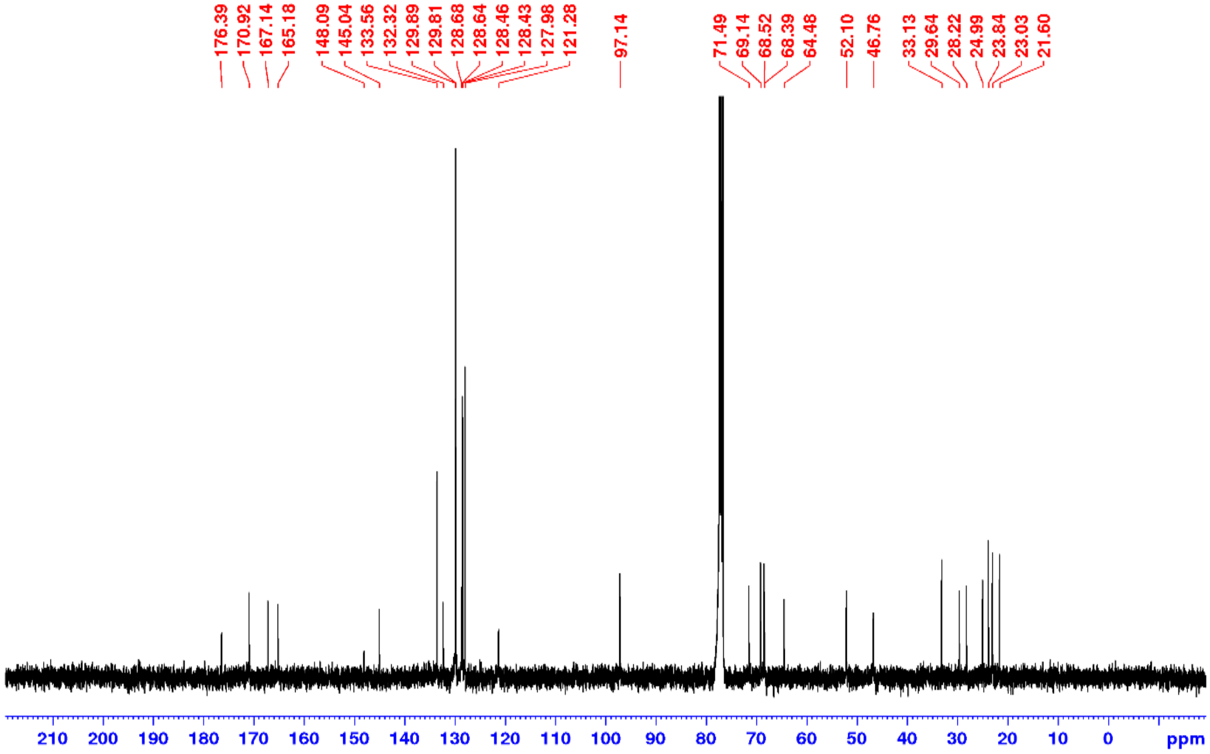

^{1}H NMR and ^{13}C NMR spectra of compound **12a** in $CDCl_3$

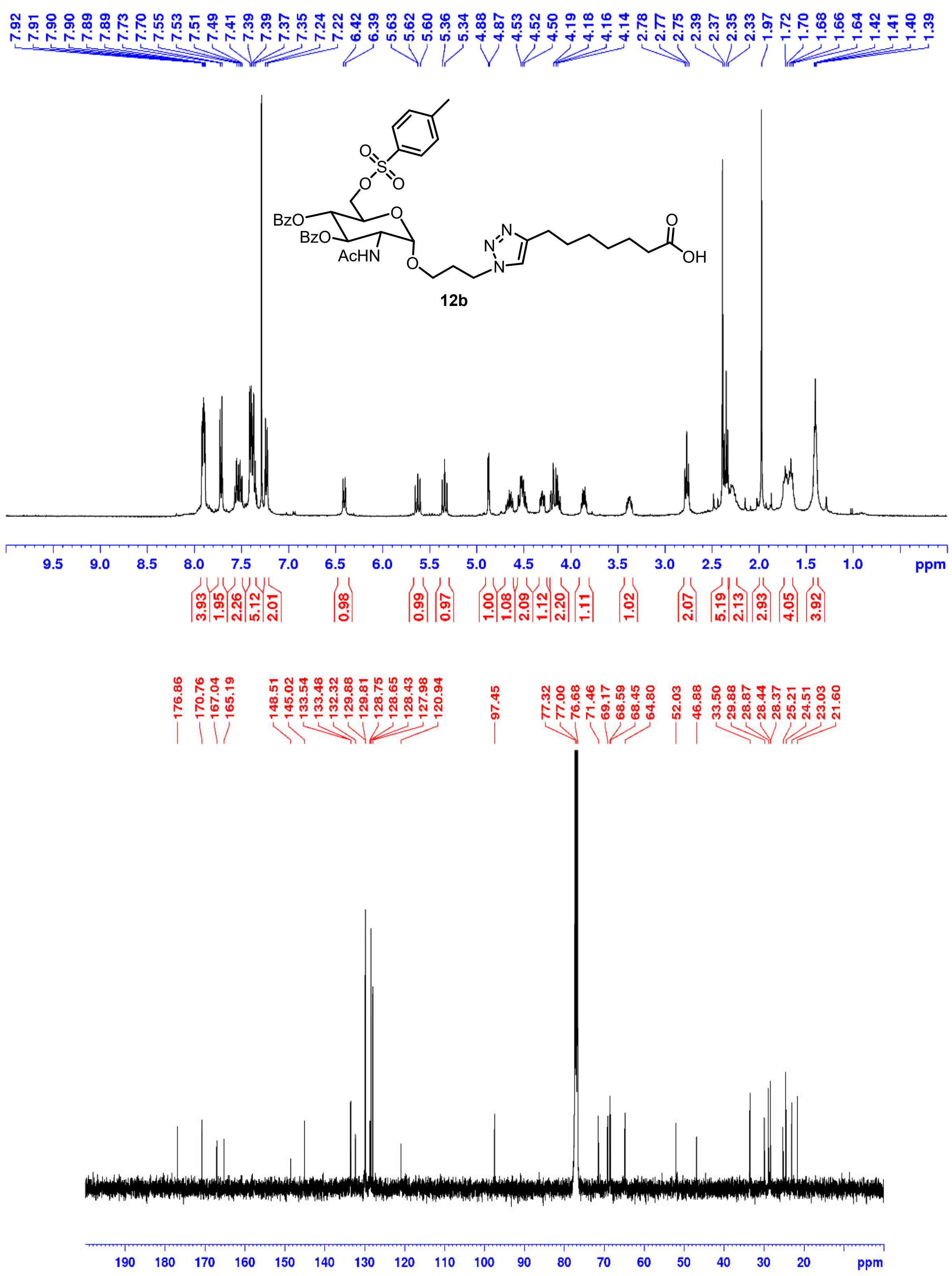

^{1}H NMR and ^{13}C NMR spectra of compound **12b** in $CDCl_3$

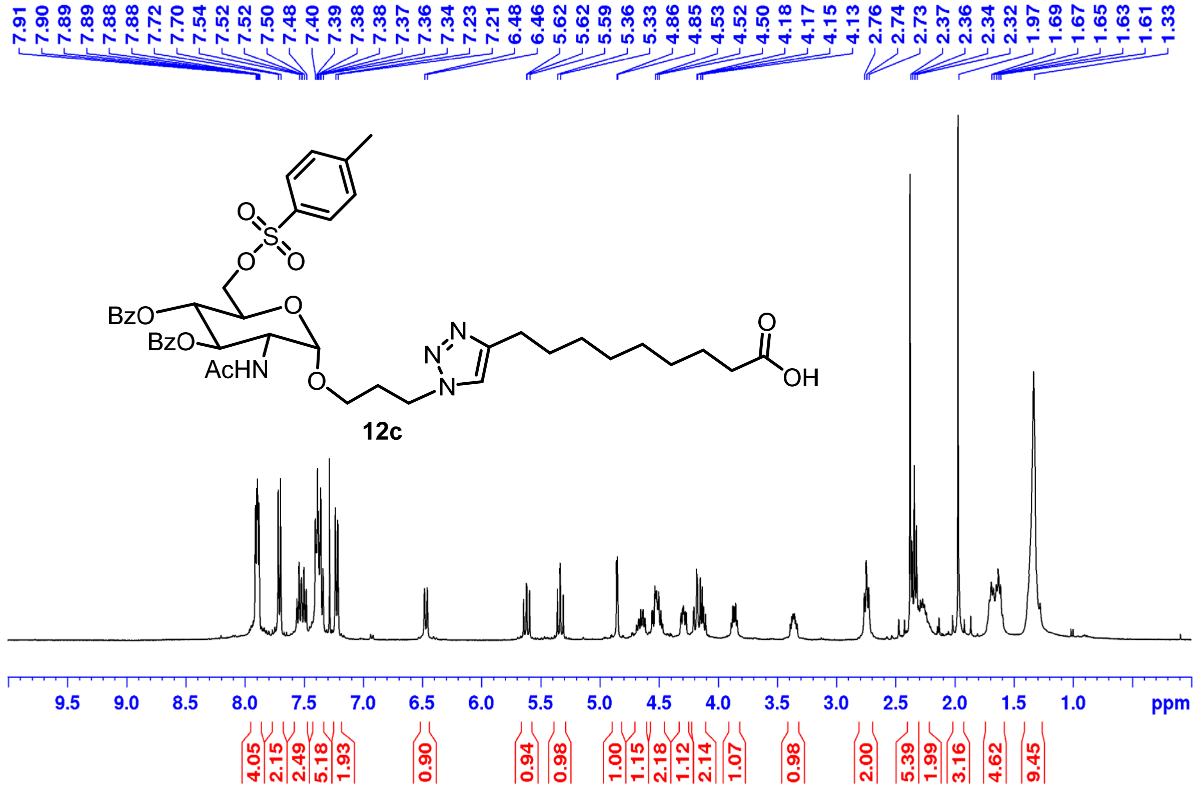

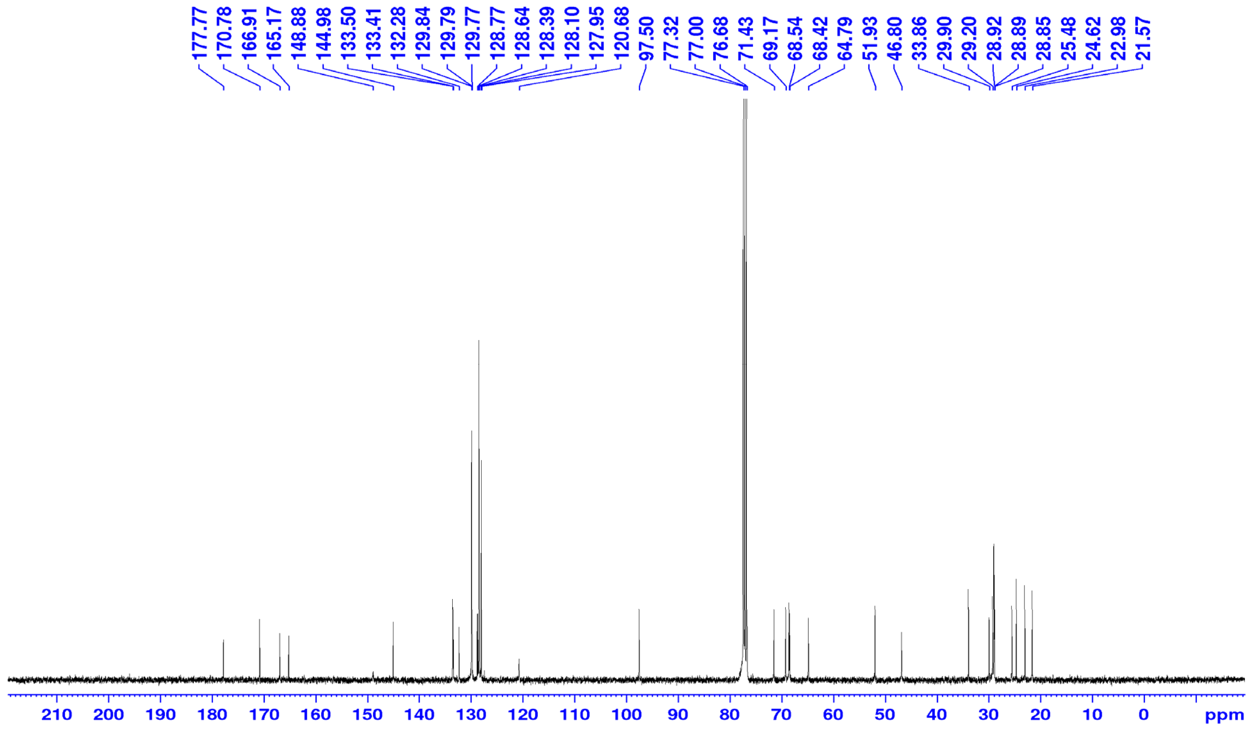

^{1}H NMR and ^{13}C NMR spectra of compound **12c** in $CDCl_3$

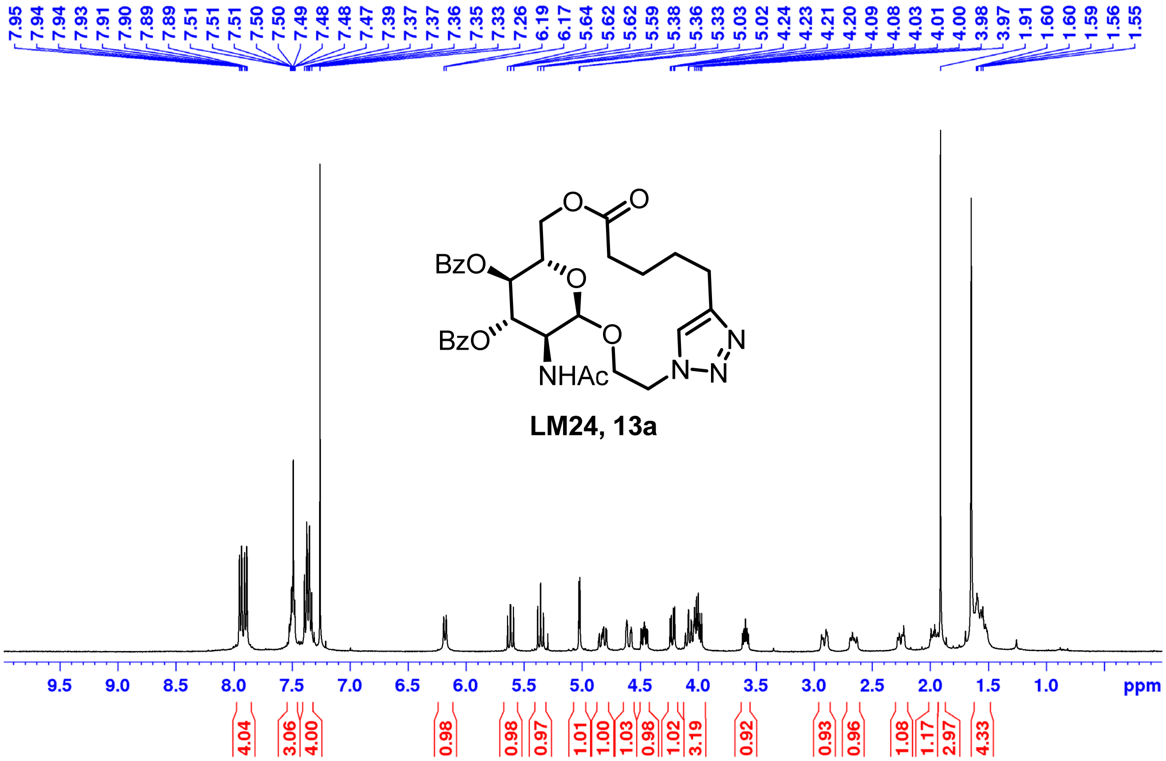

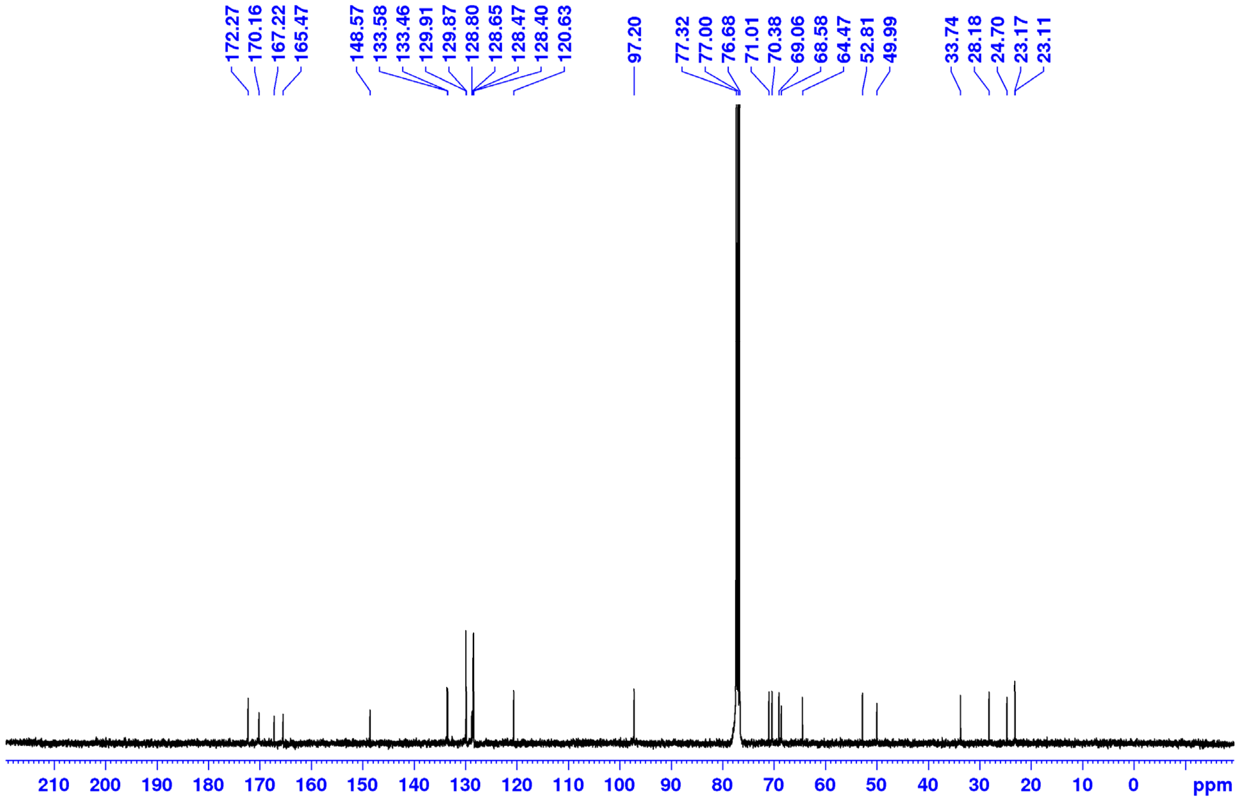

^{1}H NMR and ^{13}C NMR spectra of compound **LM24**, **13a** in $CDCl_3$

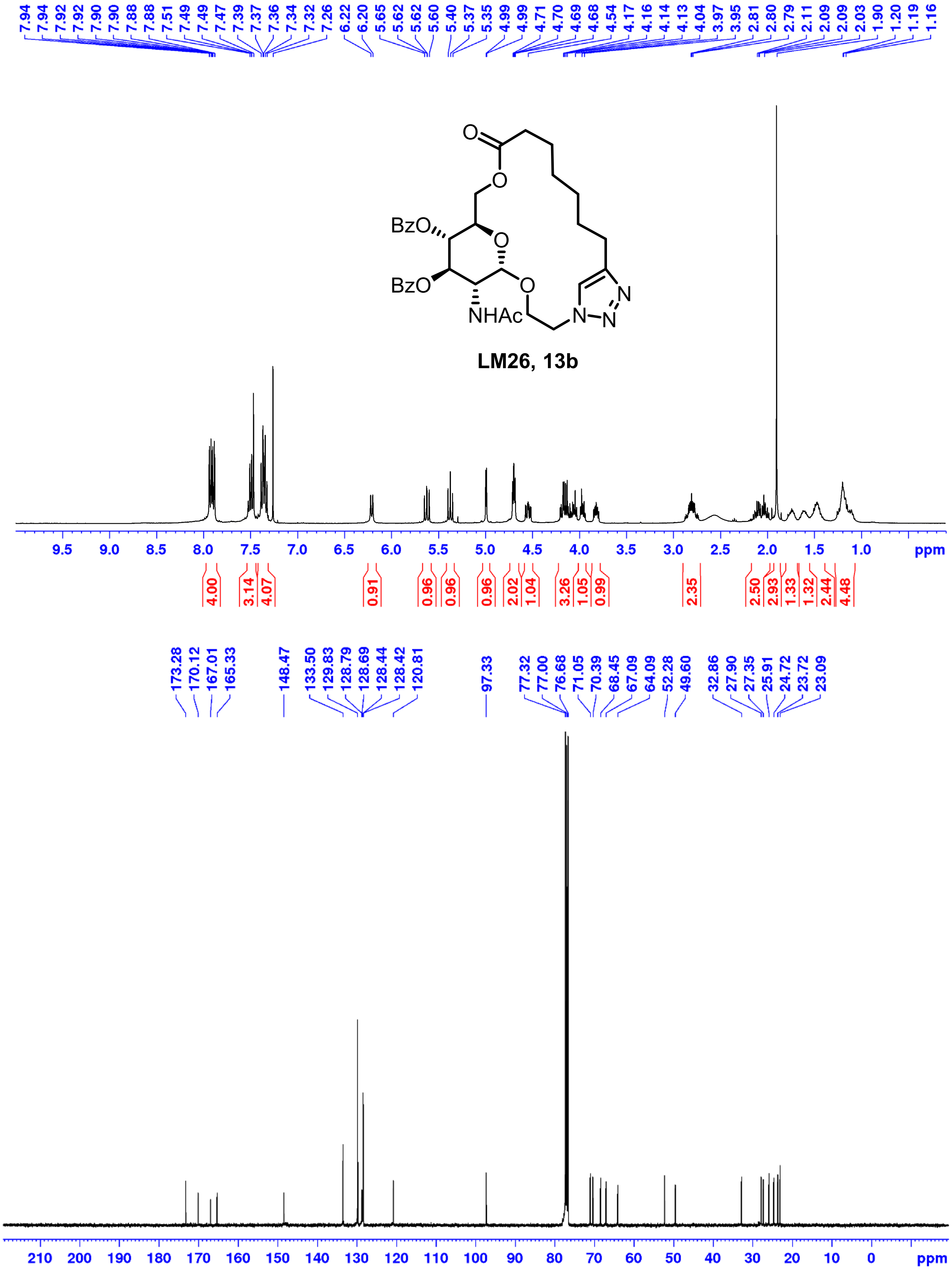

^{1}H NMR and ^{13}C NMR spectra of compound **LM26**, **13b** in $CDCl_3$

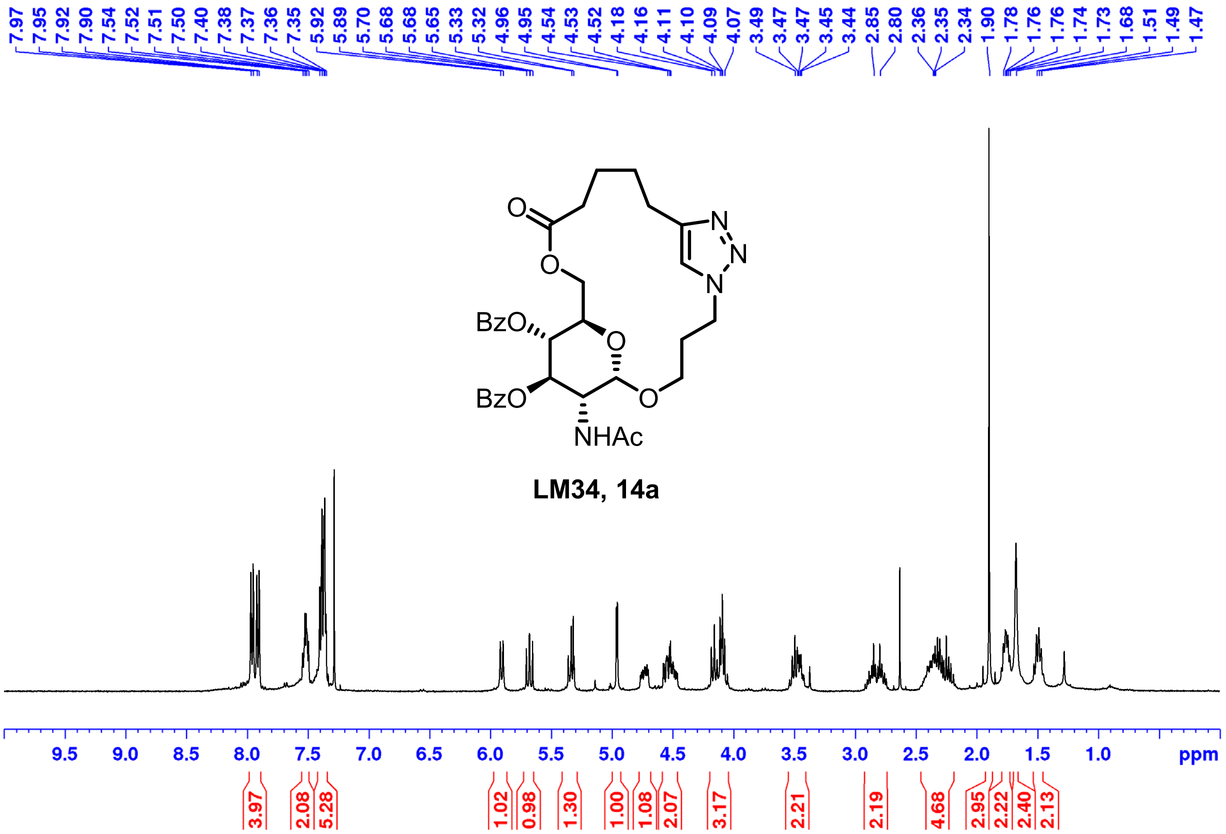

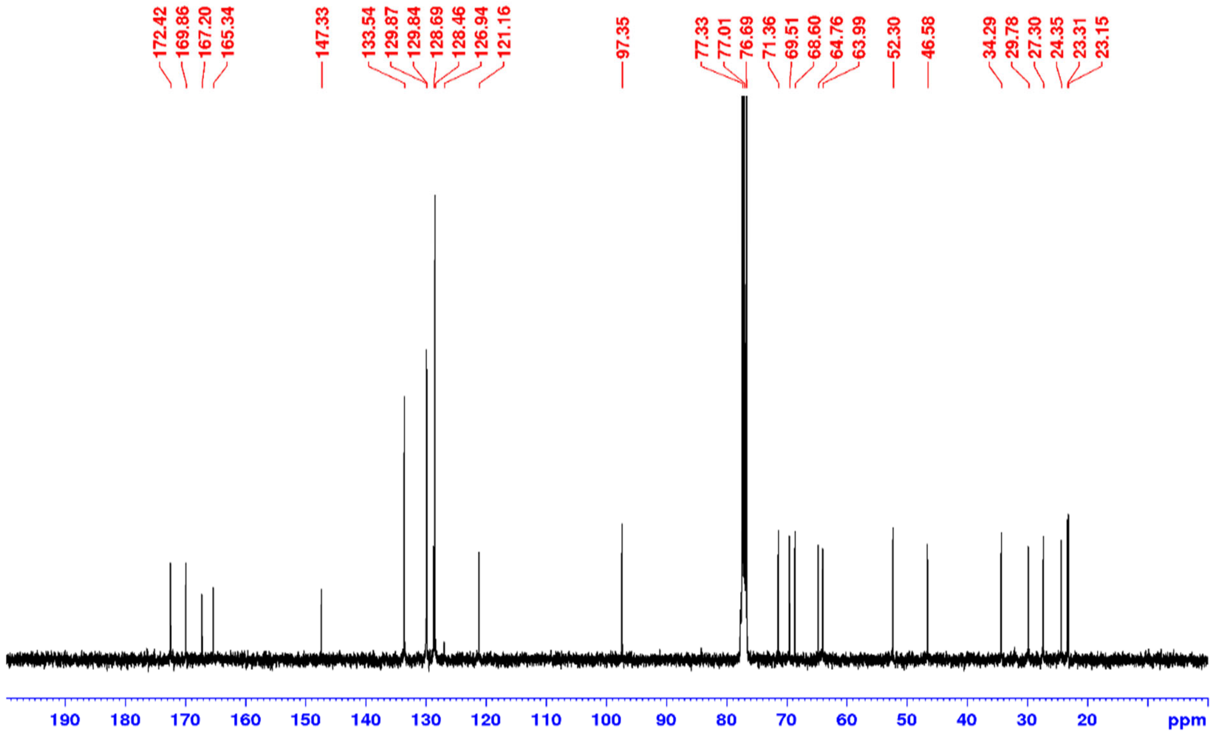

^{1}H NMR and ^{13}C NMR spectra of compound **LM34**, **14a** in $CDCl_3$

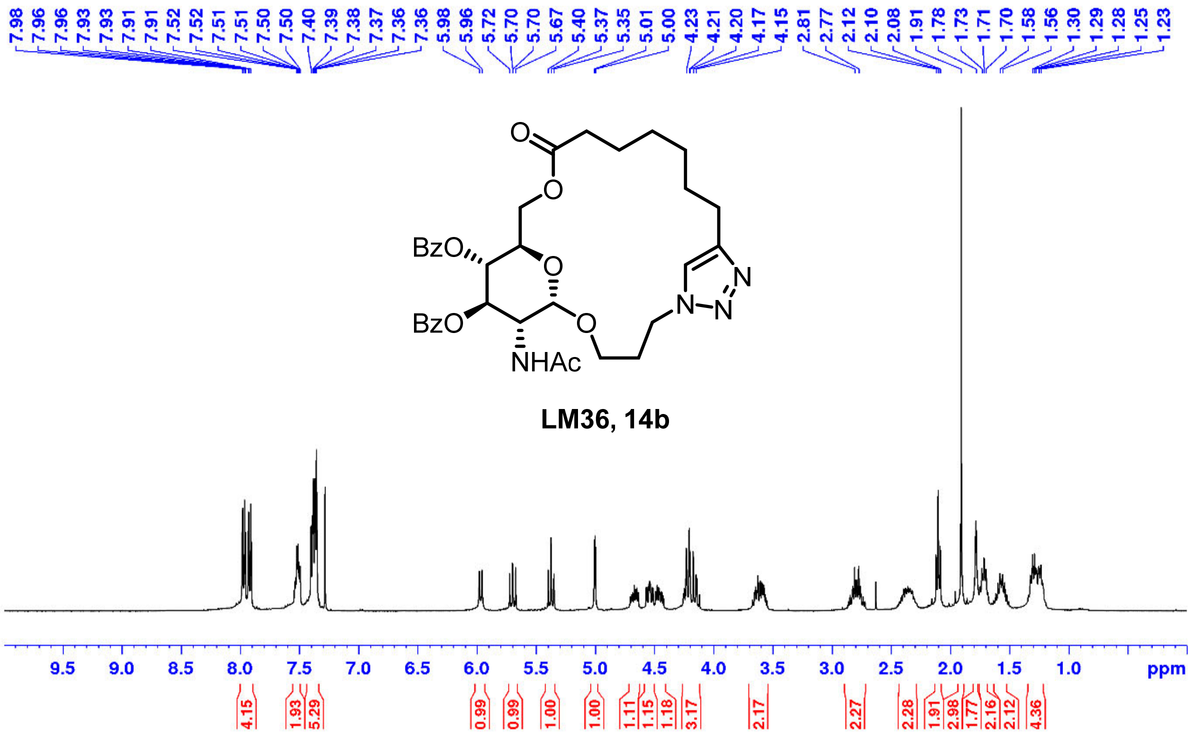

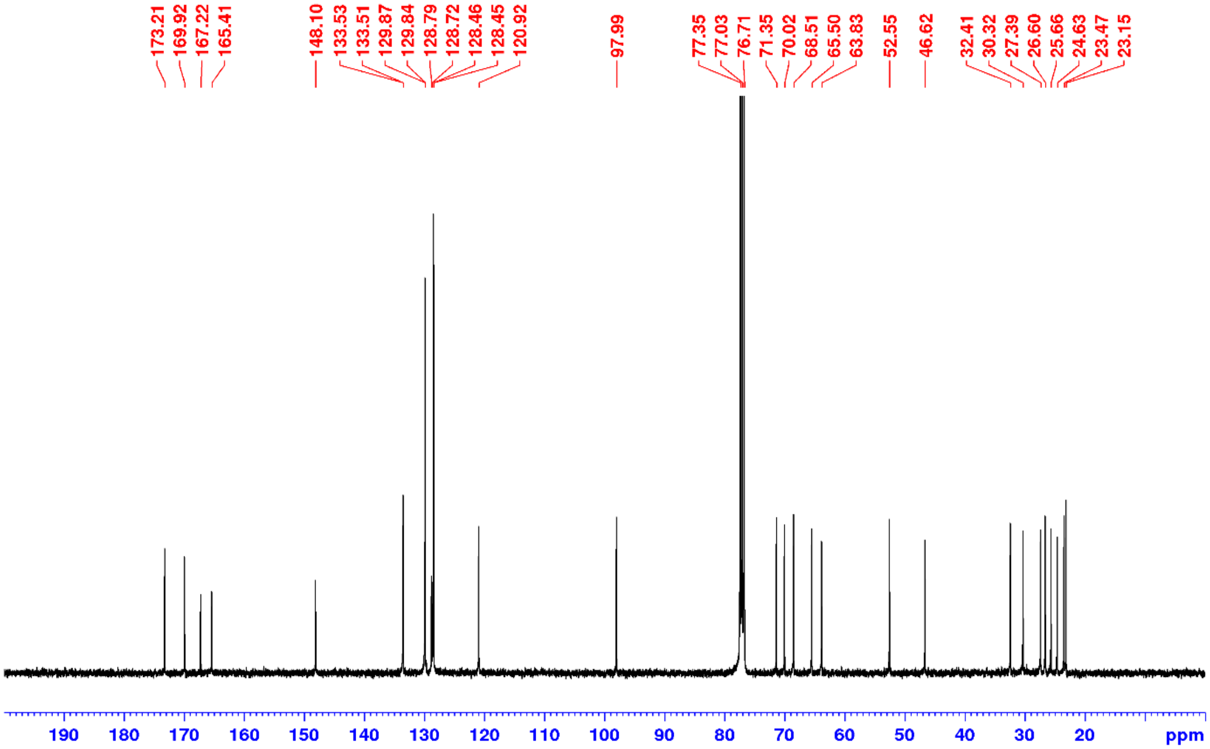

^{1}H NMR and ^{13}C NMR spectra of compound **LM36**, **14b** in $CDCl_3$

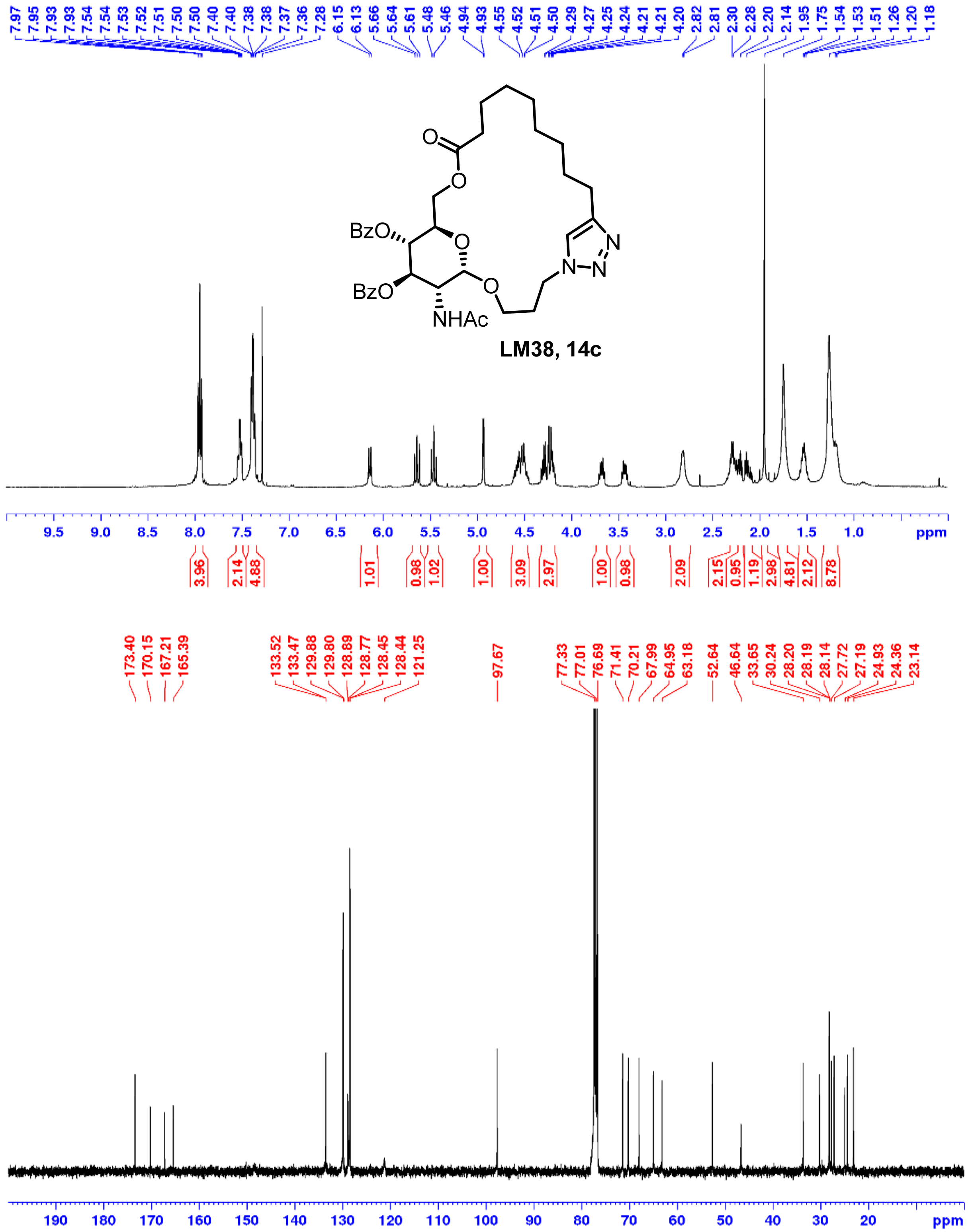

^{1}H NMR and ^{13}C NMR spectra of compound **LM38**, **14c** in $CDCl_3$

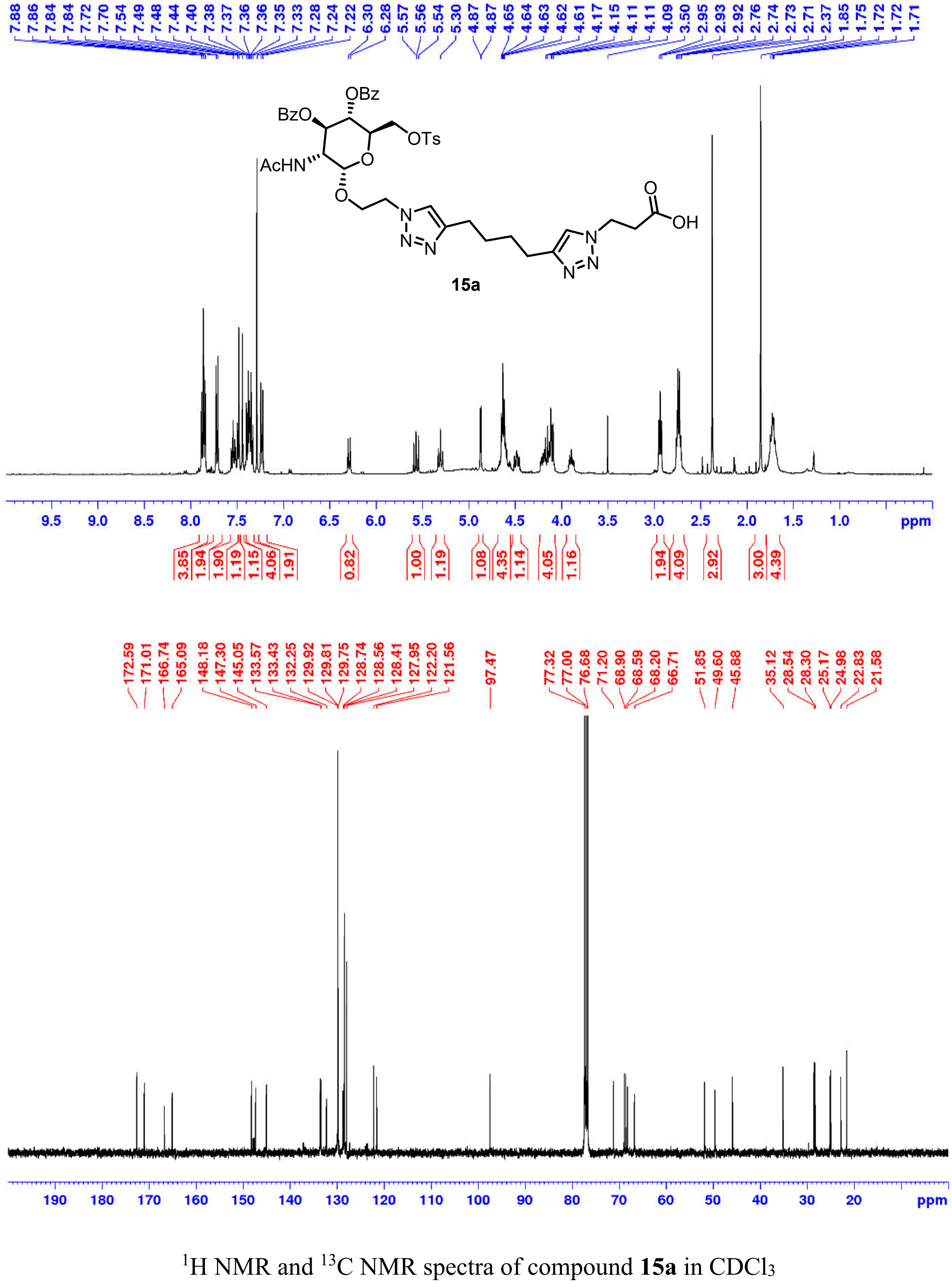

^{1}H NMR and ^{13}C NMR spectra of compound **15a** in $CDCl_3$

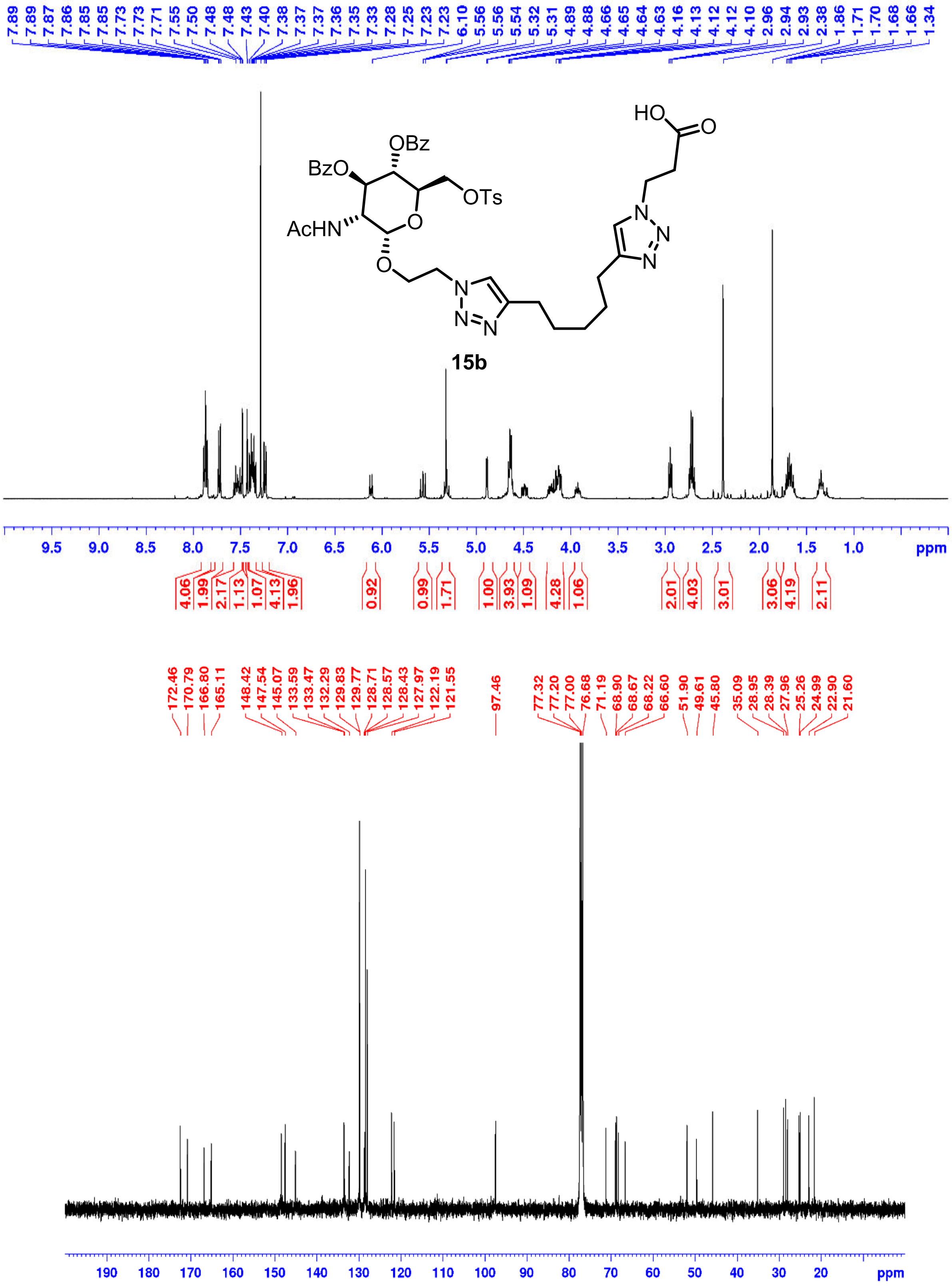

^{1}H NMR and ^{13}C NMR spectra of compound **15b** in $CDCl_3$

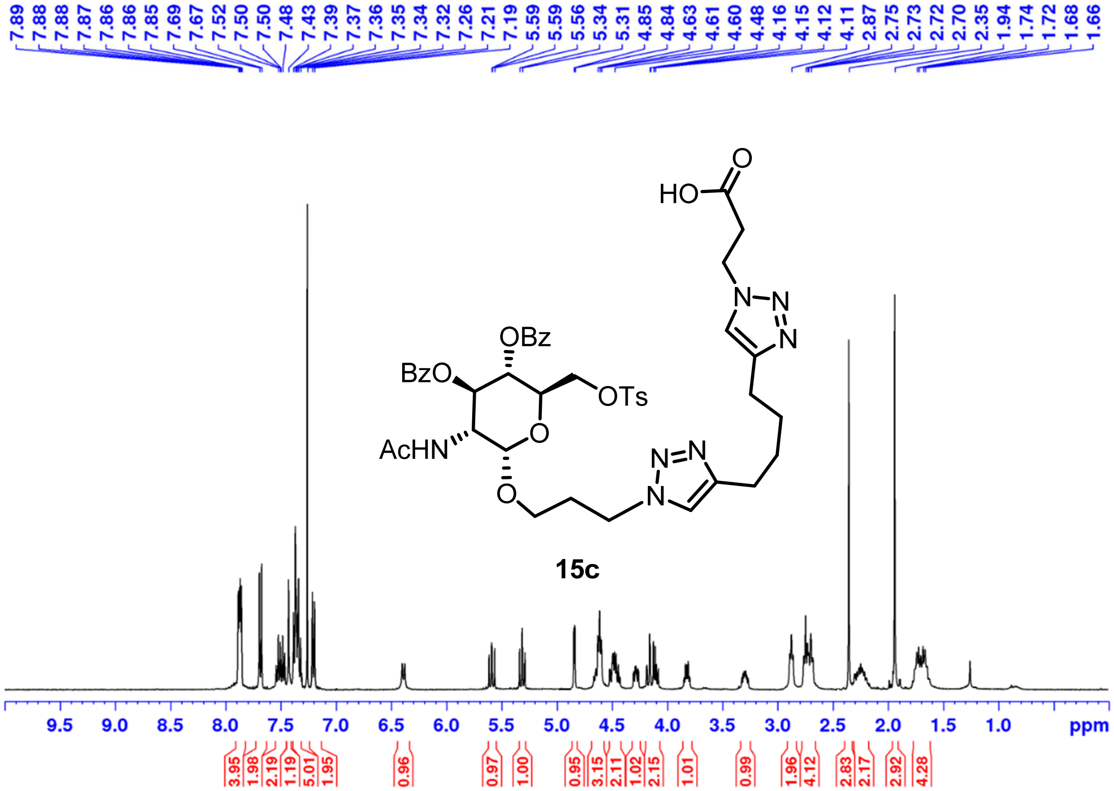

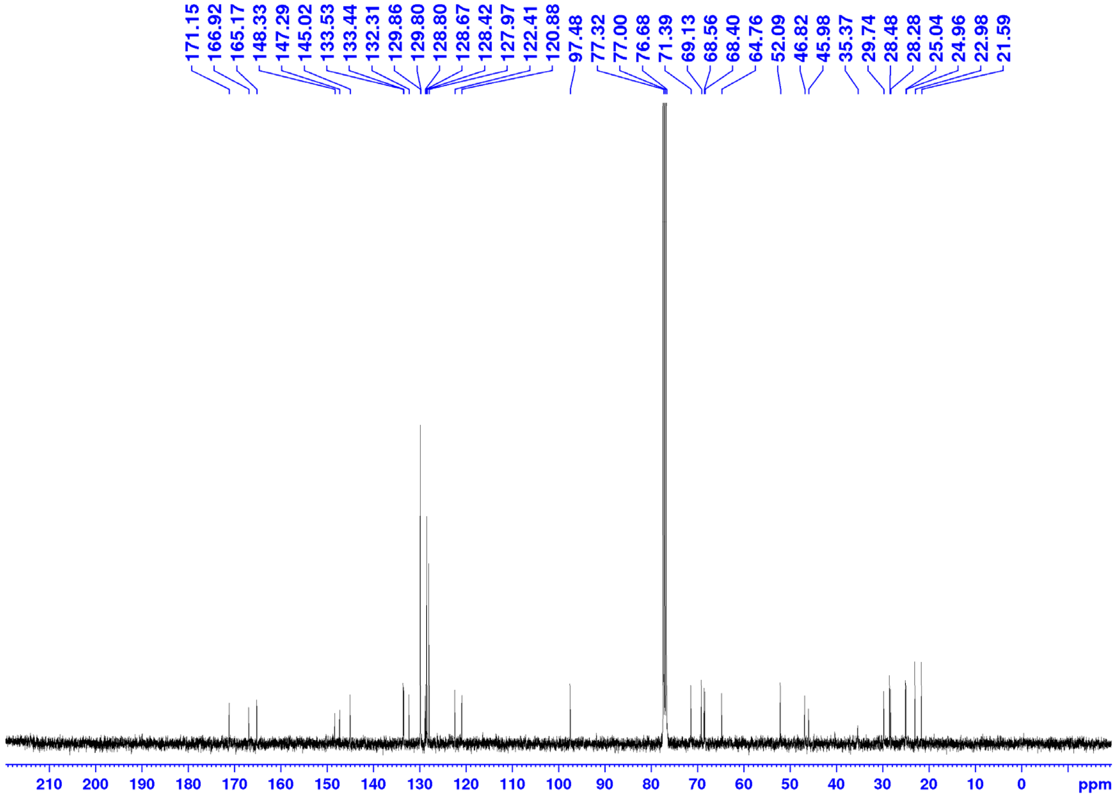

^{1}H NMR and ^{13}C NMR spectra of compound **15c** in $CDCl_3$

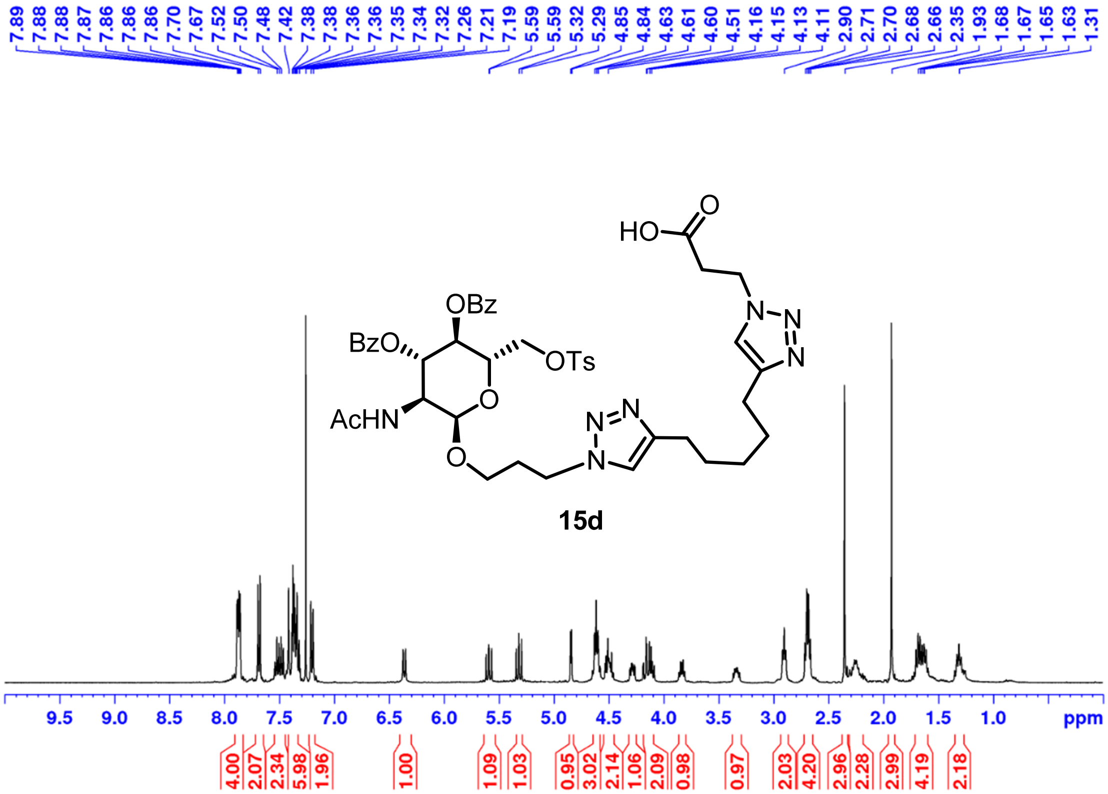

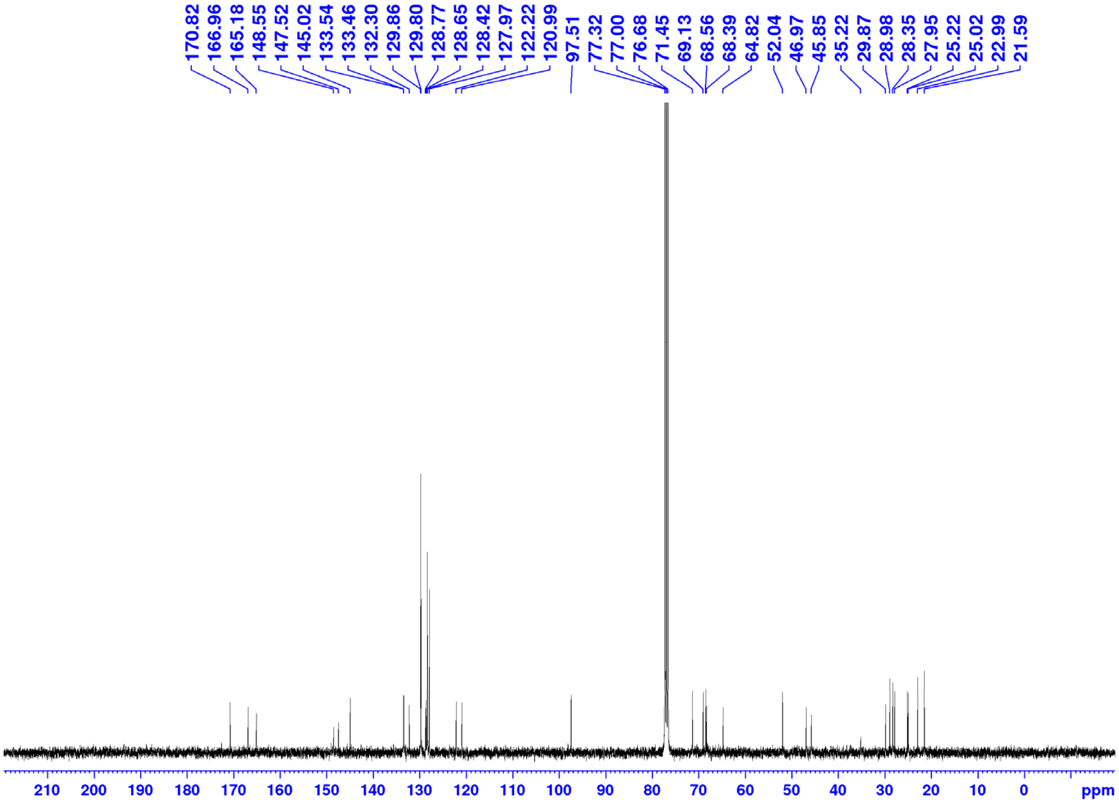

^{1}H NMR and ^{13}C NMR spectra of compound **15d** in $CDCl_3$

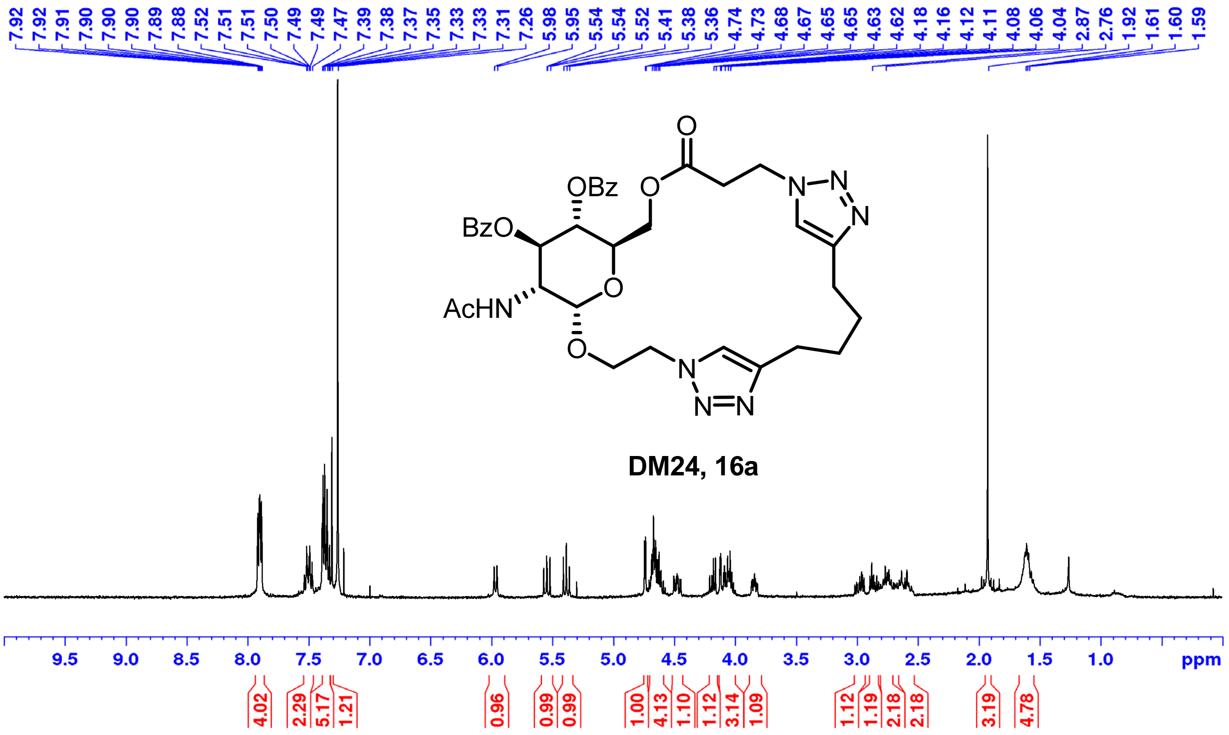

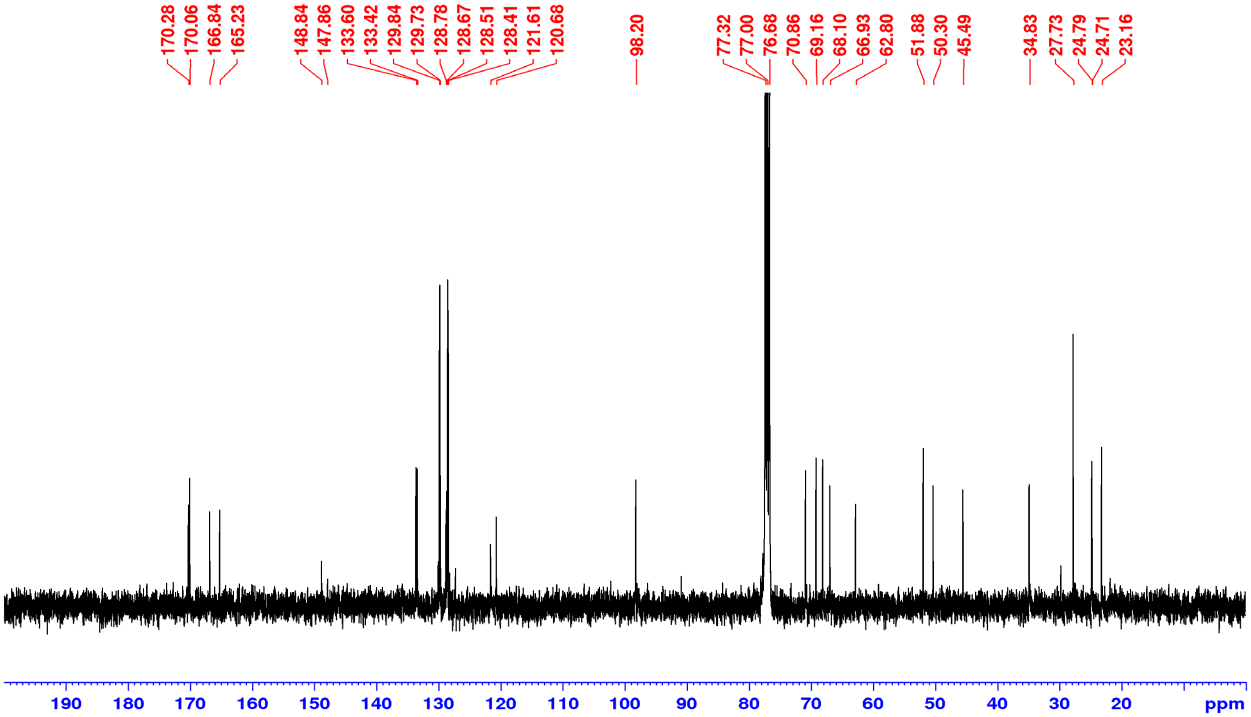

^{1}H NMR and ^{13}C NMR spectra of compound **DM24**, **16a** in $CDCl_3$

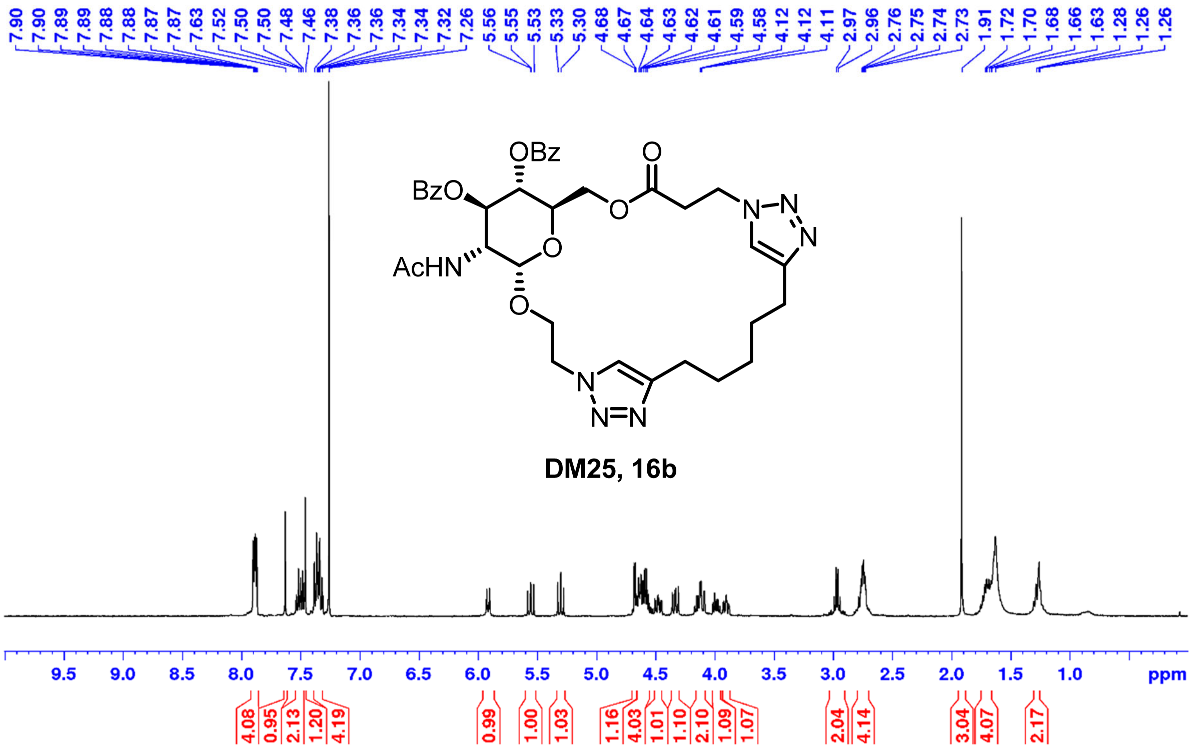

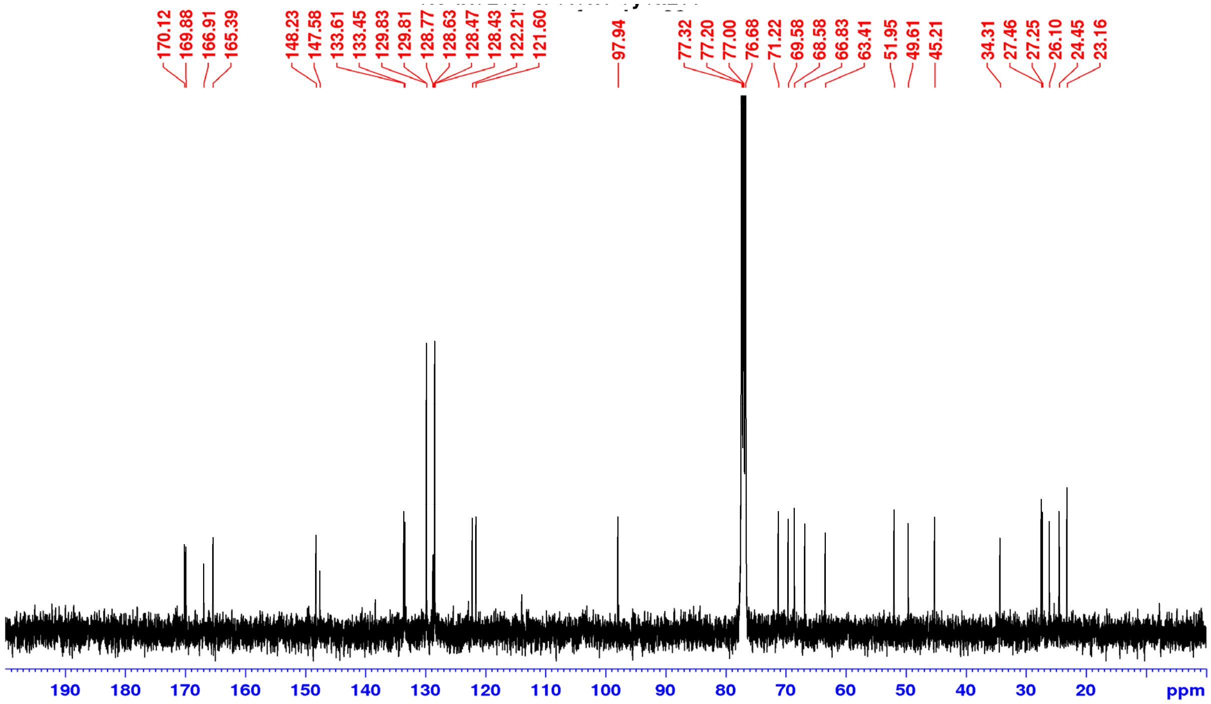

^{1}H NMR and ^{13}C NMR spectra of compound **DM25**, **16b** in $CDCl_3$

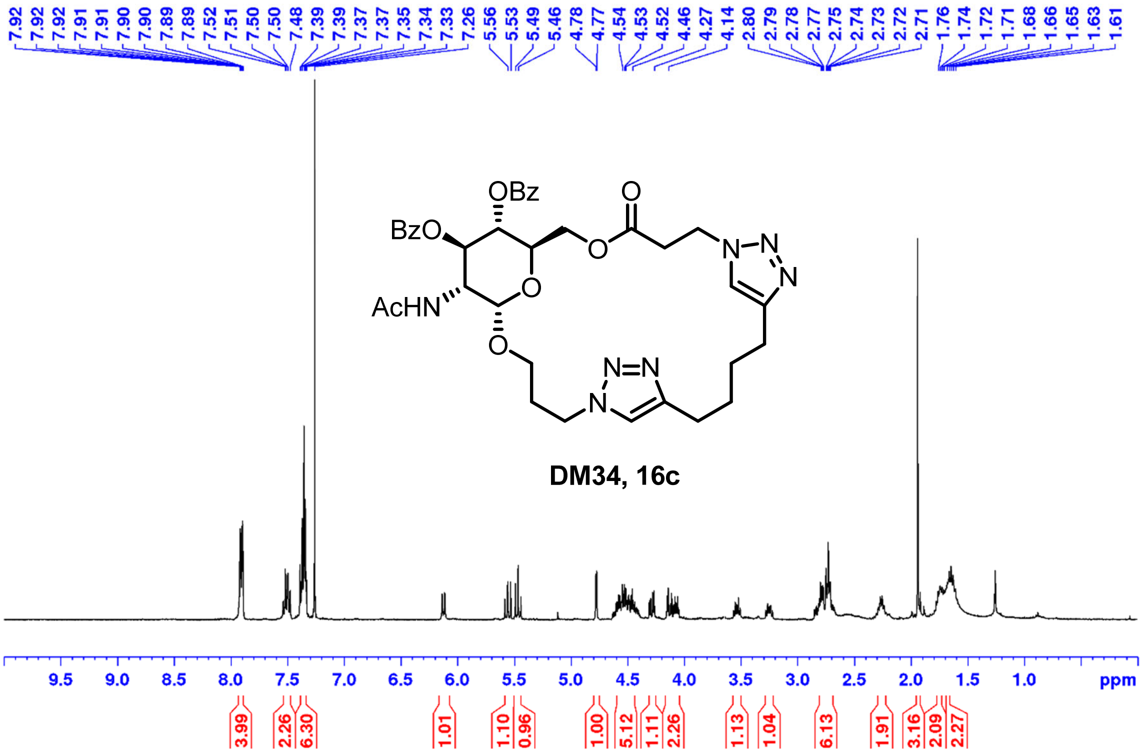

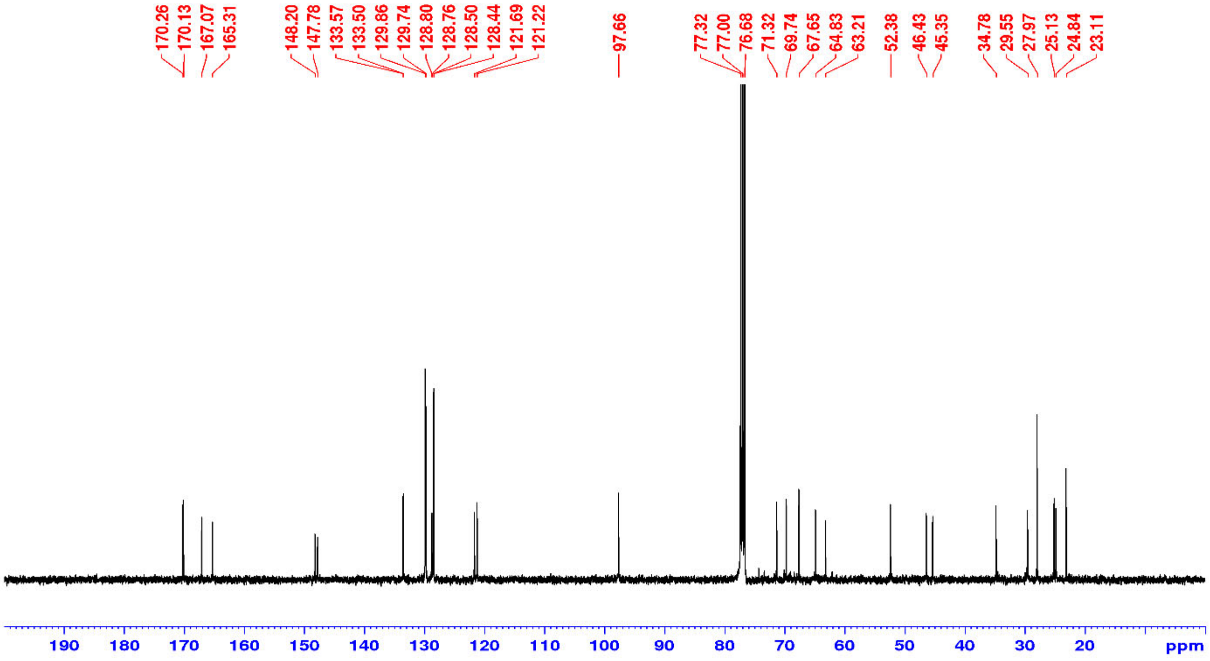

^{1}H NMR and ^{13}C NMR spectra of compound **DM34**, **16c** in $CDCl_3$

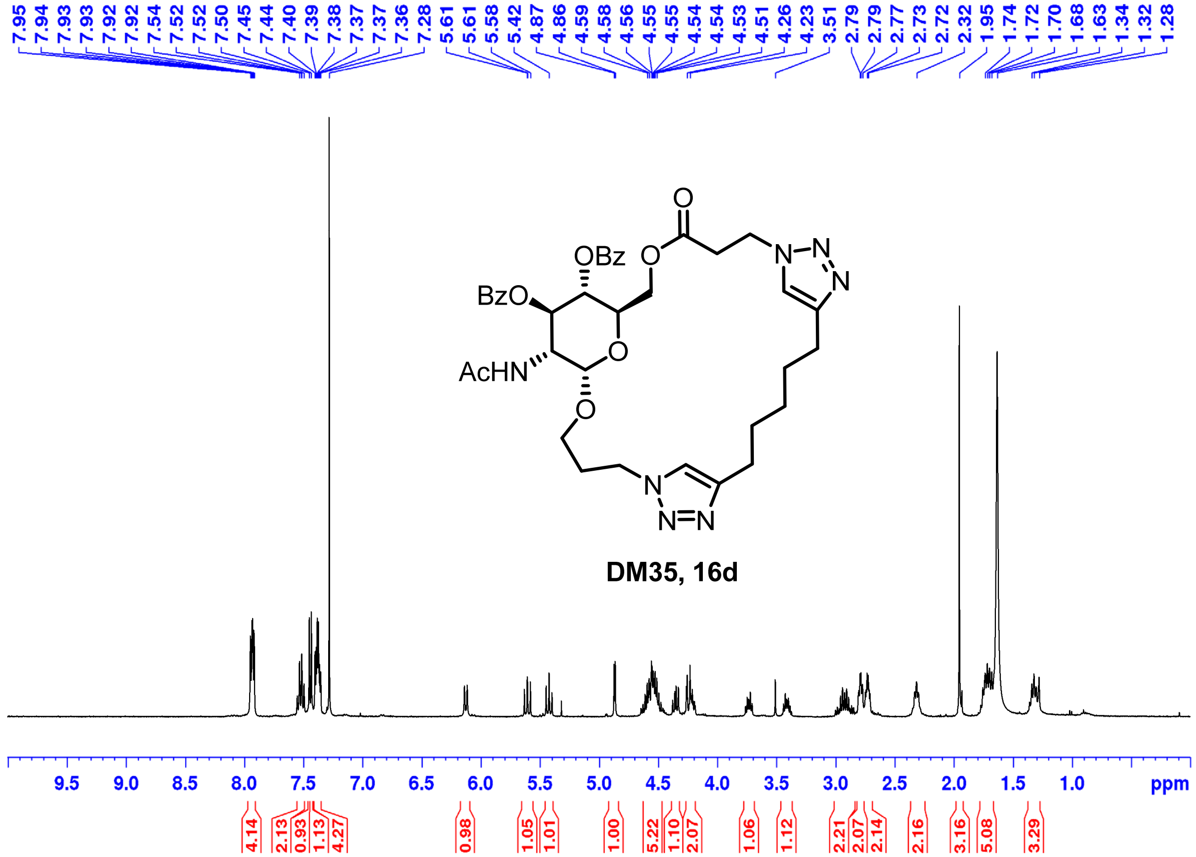

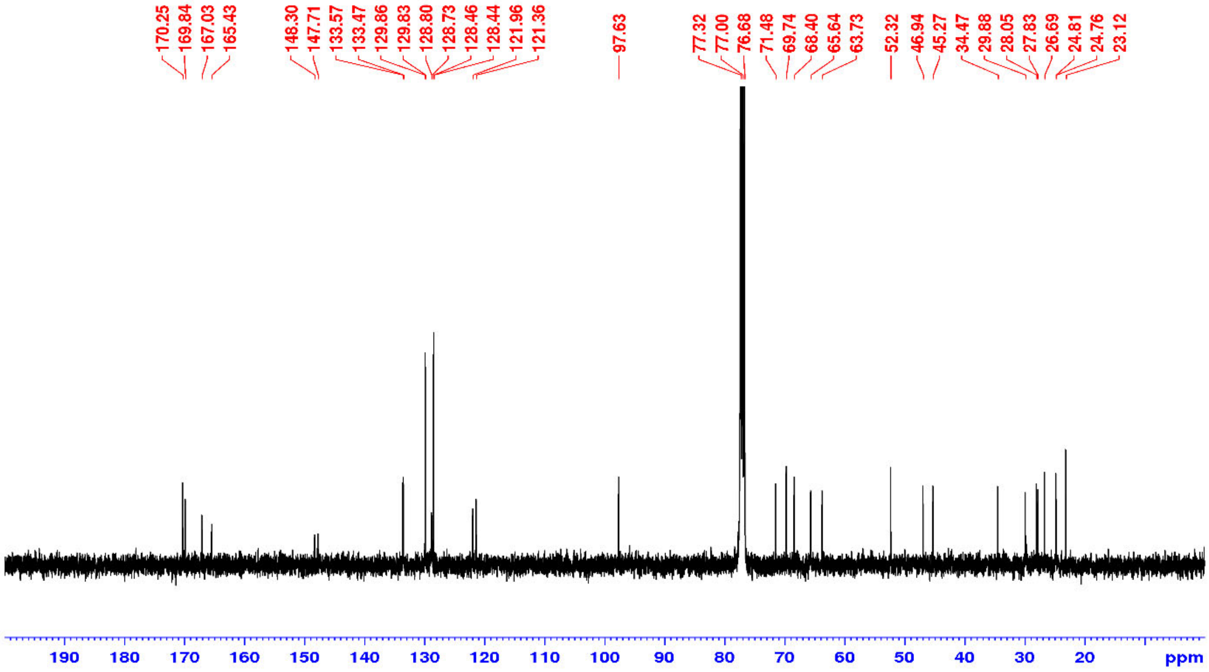

^{1}H NMR and ^{13}C NMR spectra of compound **DM35**, **16d** in $CDCl_3$

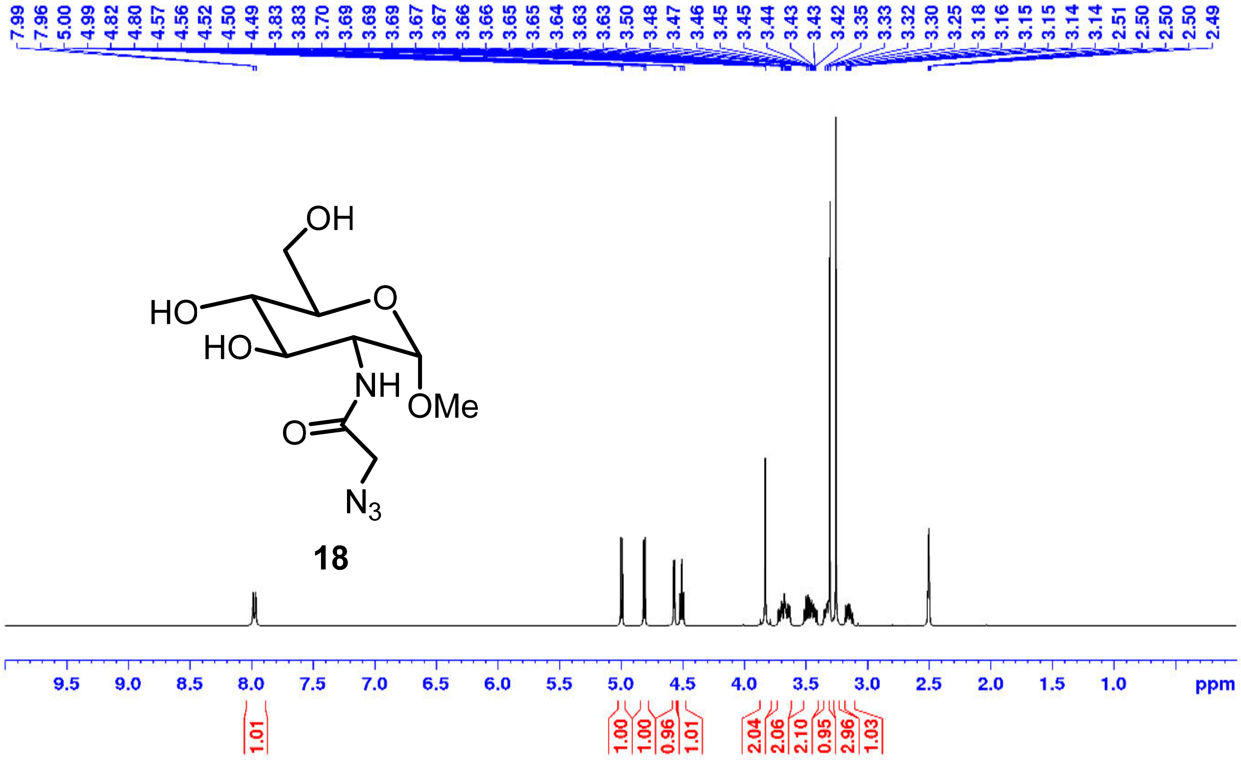

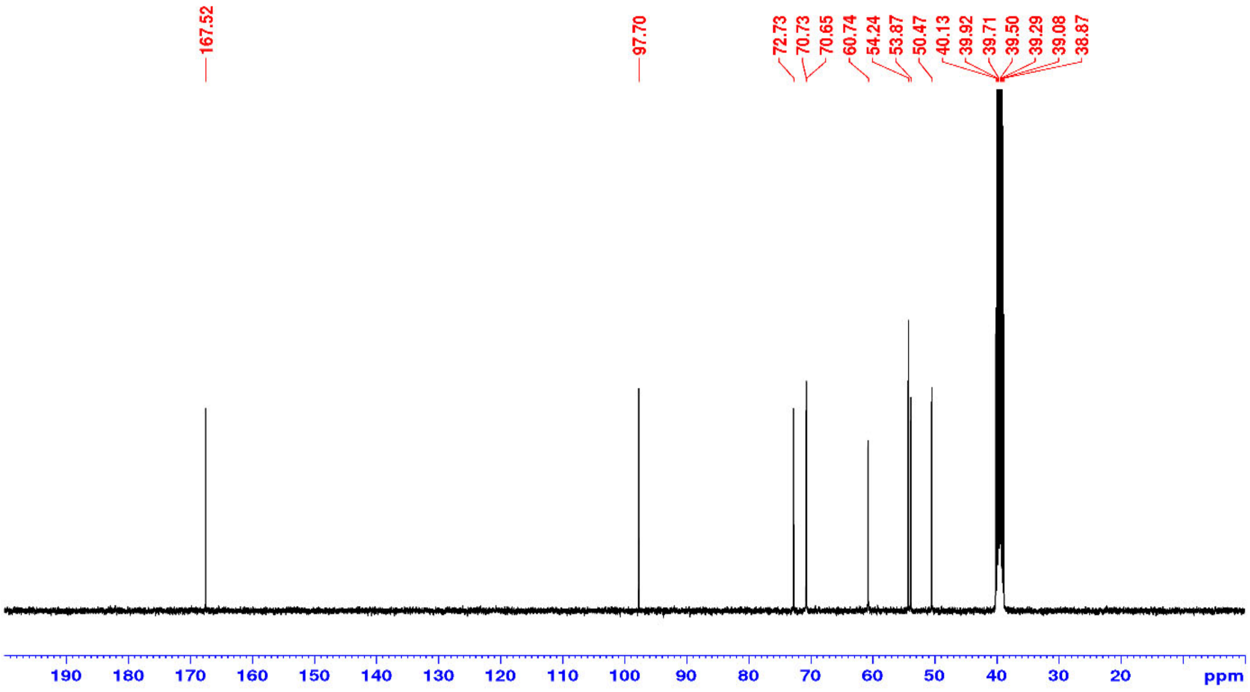

^{1}H NMR and ^{13}C NMR spectra of compound **18** in $CDCl_3$

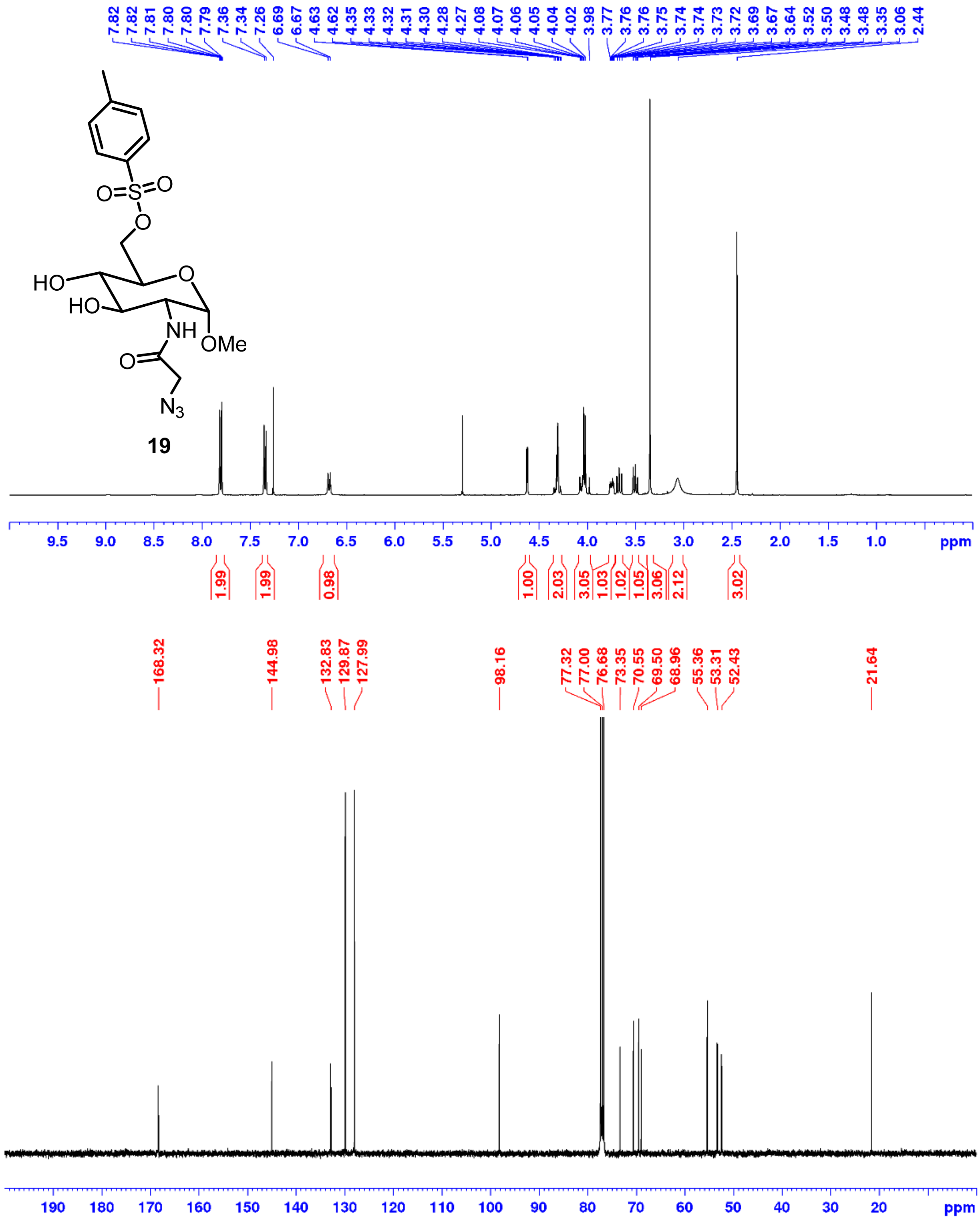

^{1}H NMR and ^{13}C NMR spectra of compound **19** in $CDCl_3$

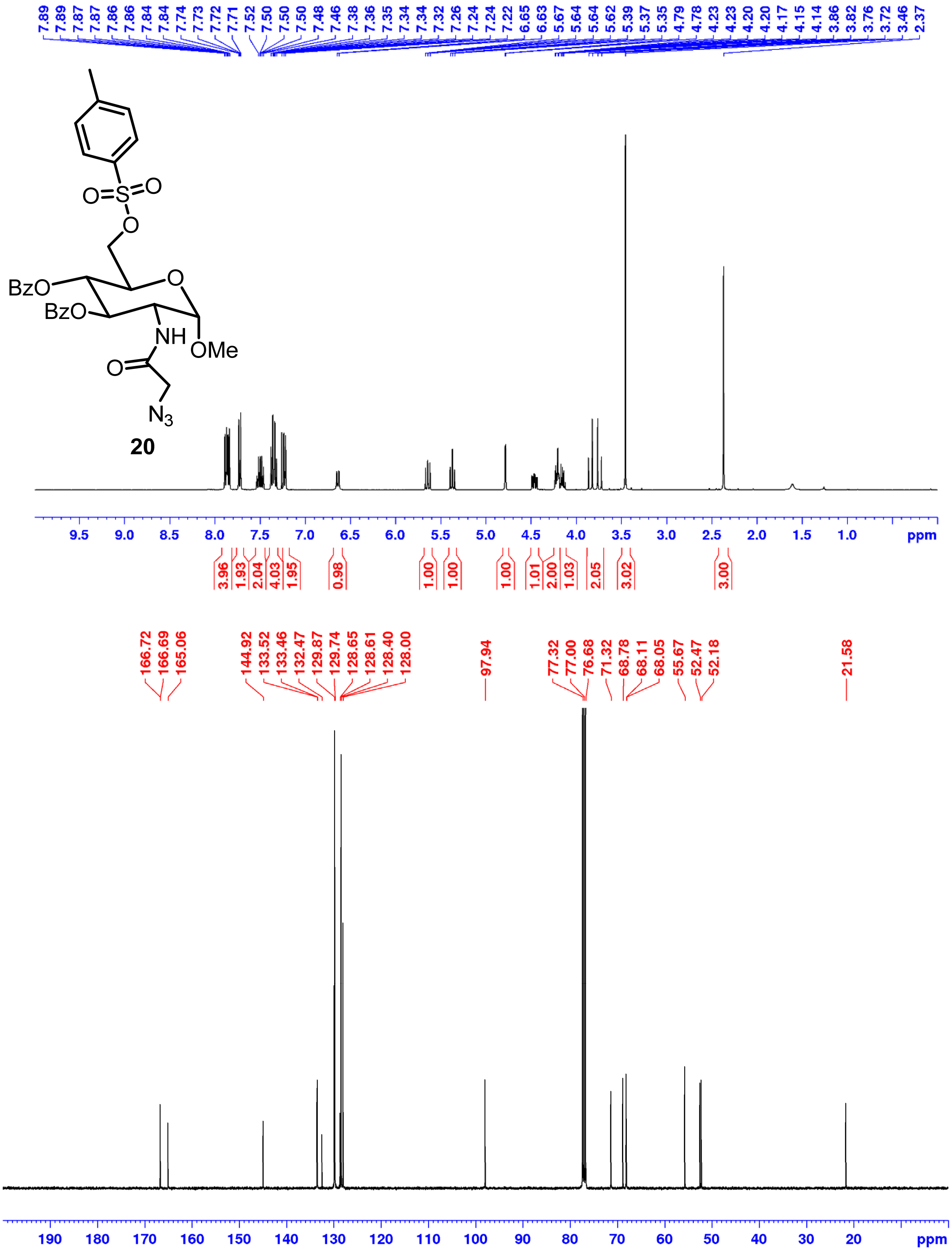

^{1}H NMR and ^{13}C NMR spectra of compound **20** in $CDCl_3$

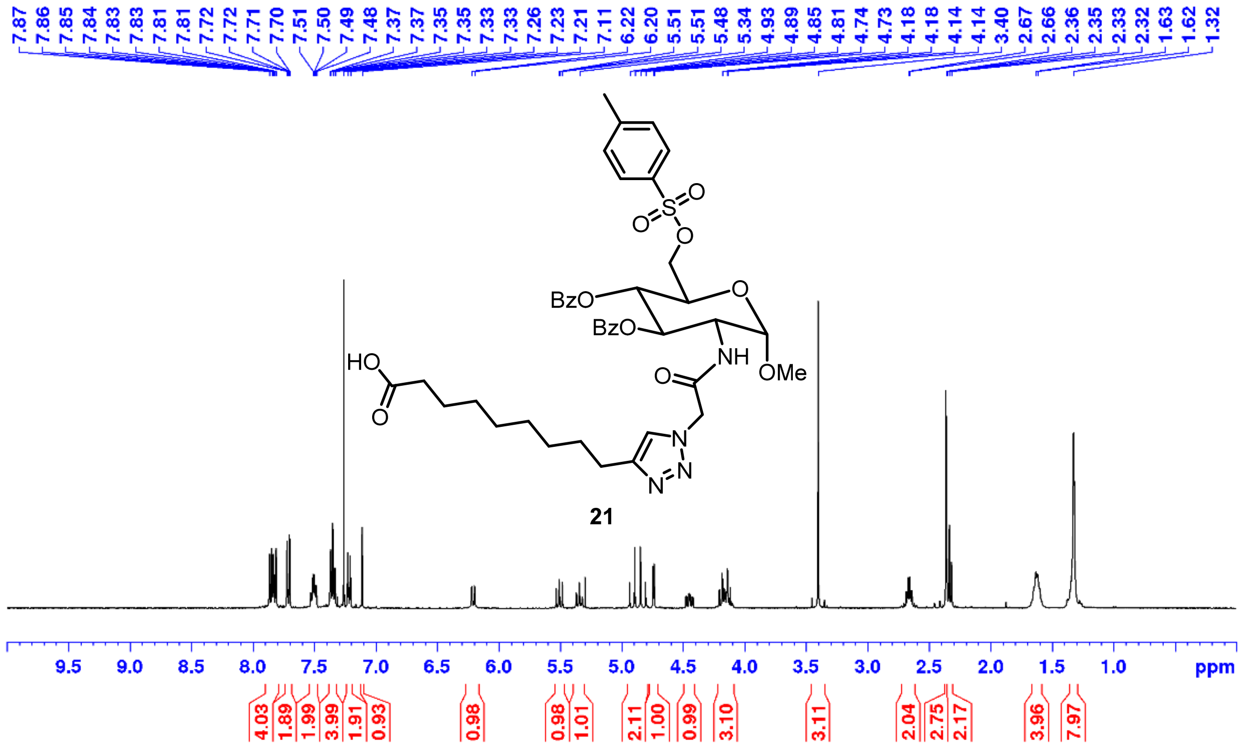

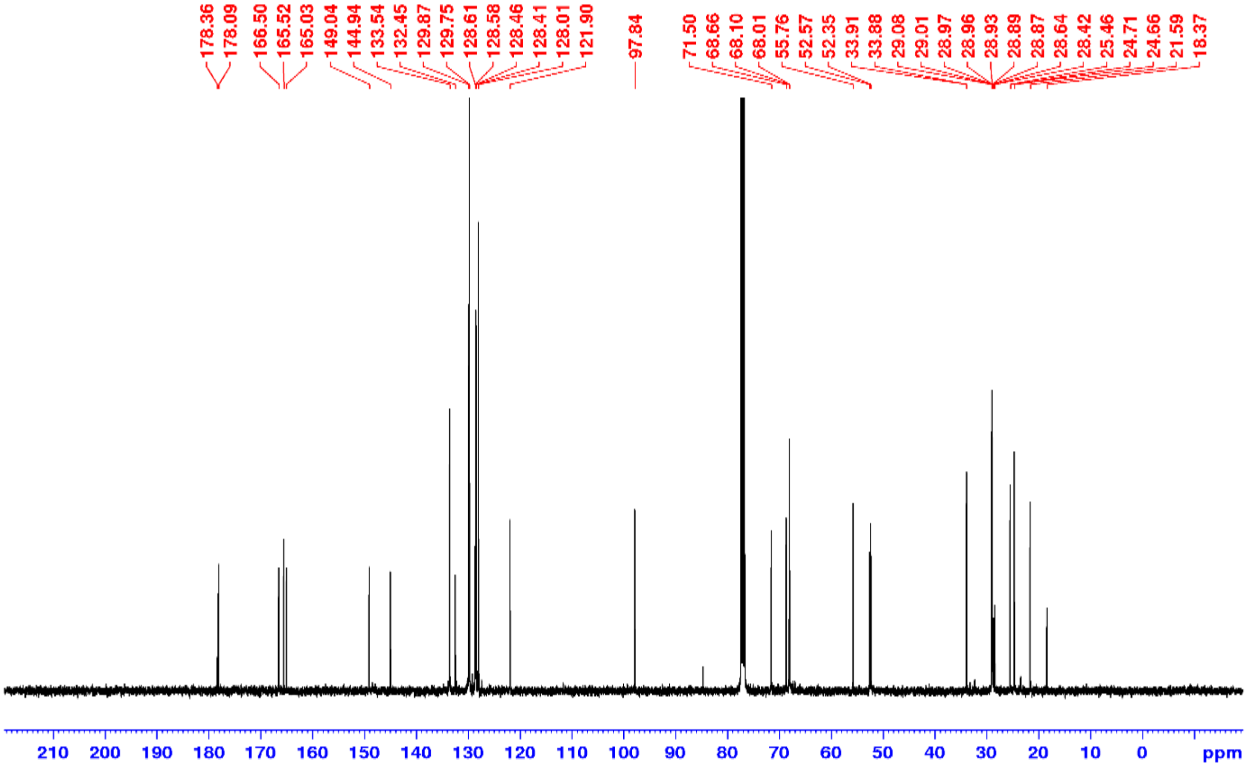

^{1}H NMR and ^{13}C NMR spectra of compound **21** in $CDCl_3$

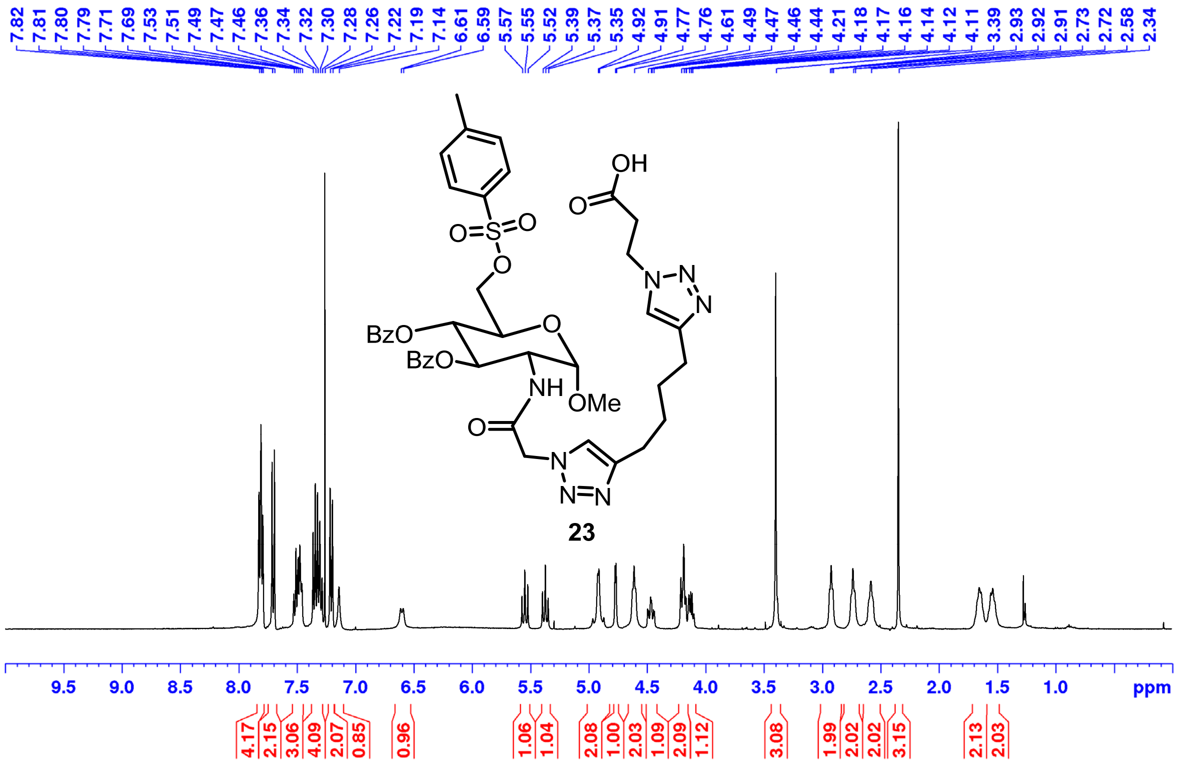

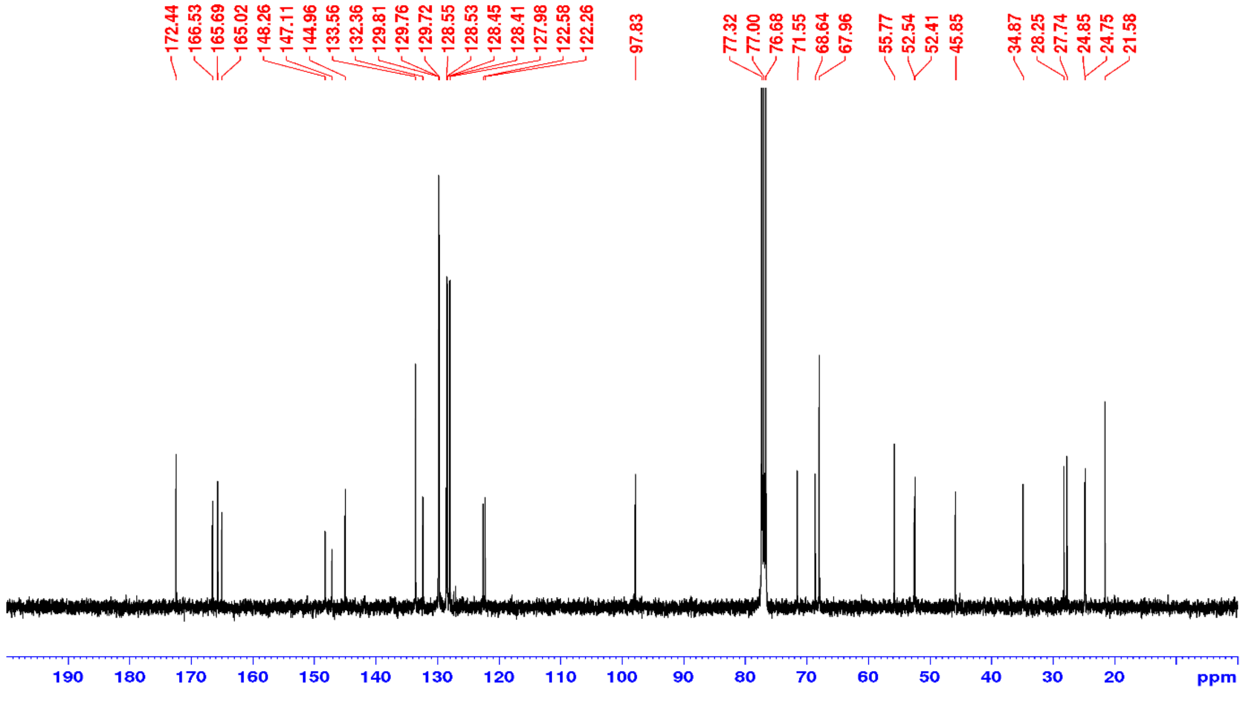

^{1}H NMR and ^{13}C NMR spectra of compound **23** in $CDCl_3$

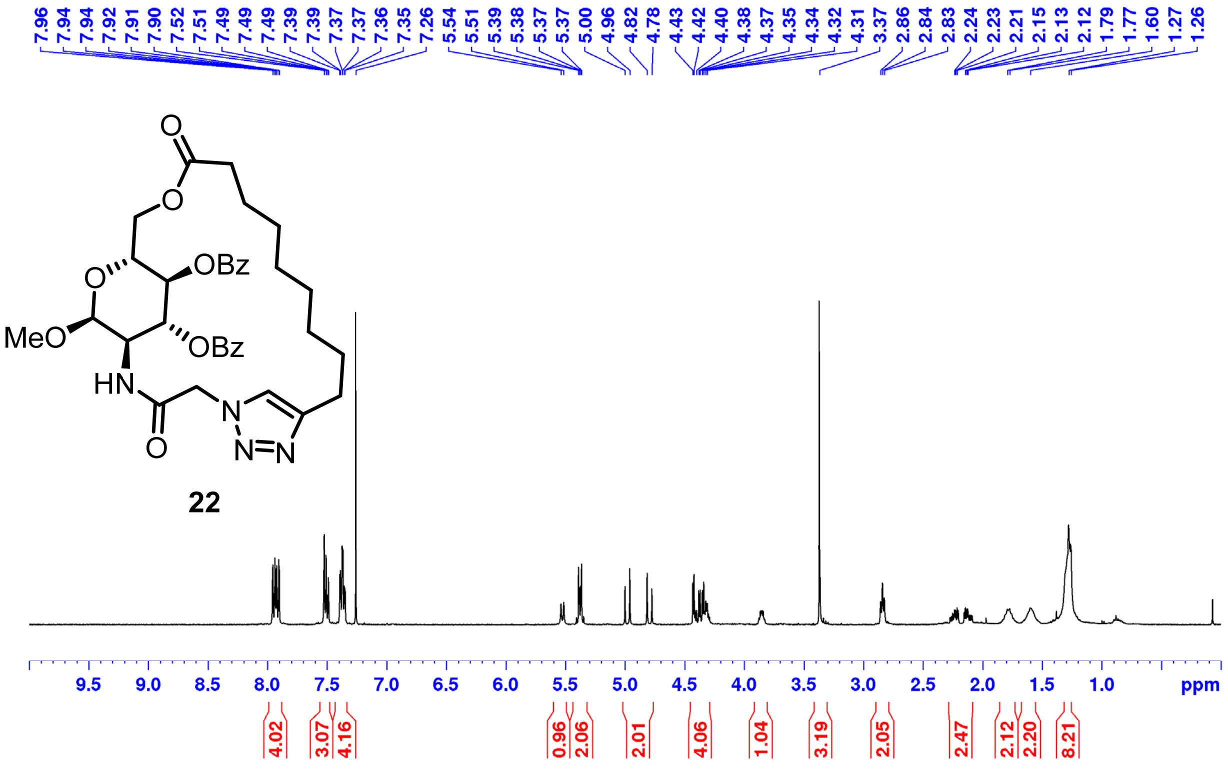

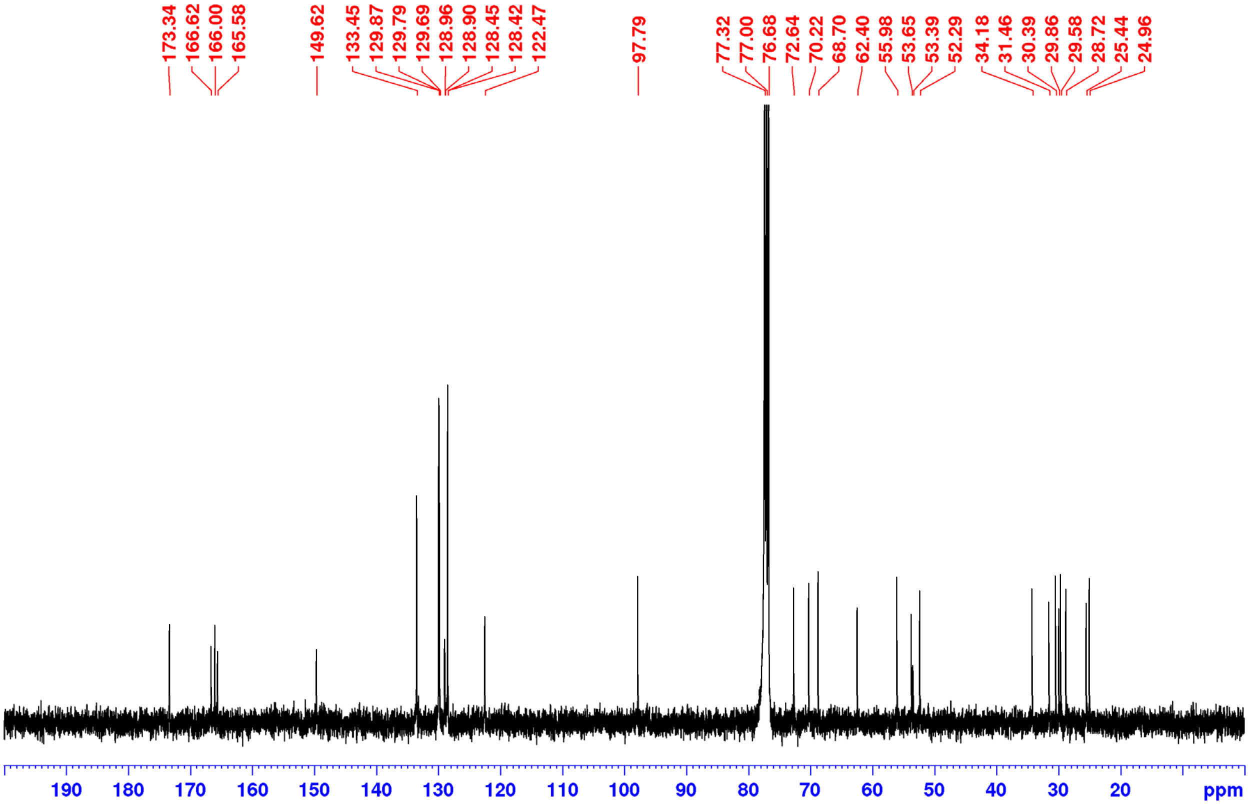

^{1}H NMR and ^{13}C NMR spectra of compound **22** in $CDCl_3$

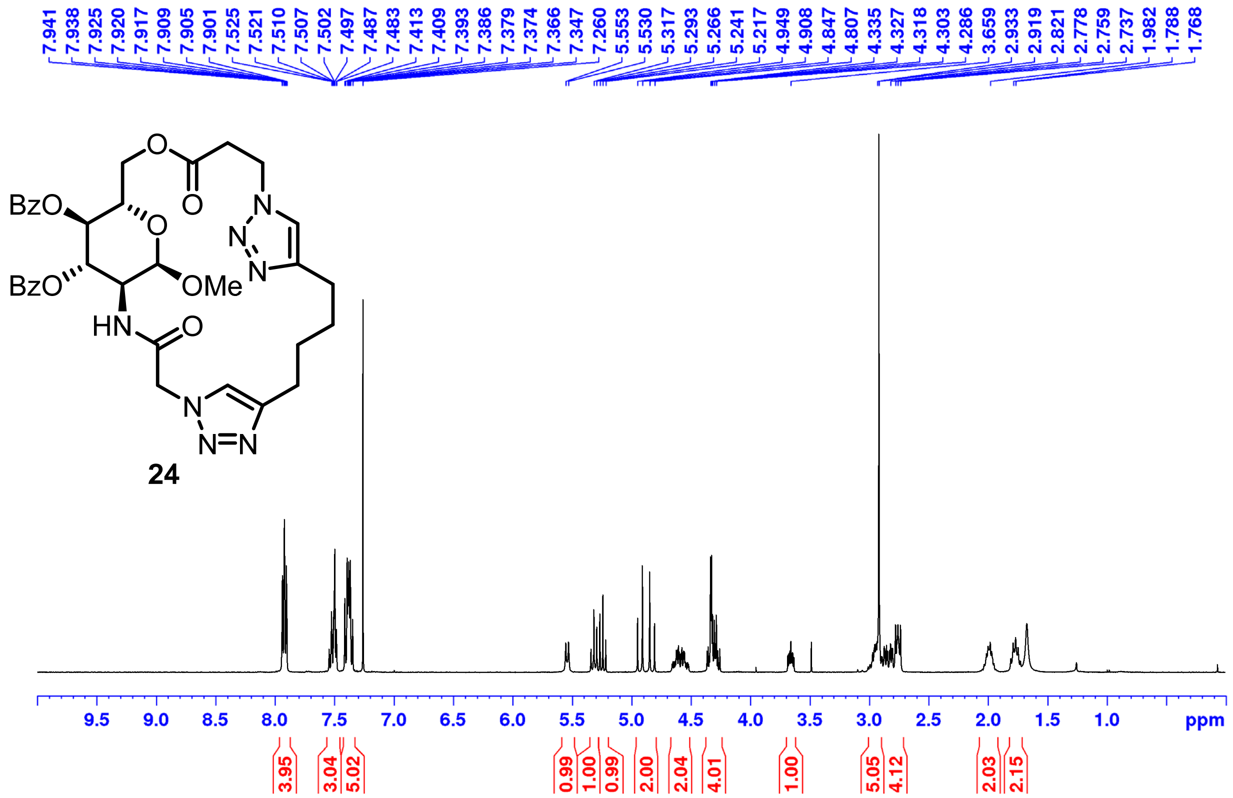

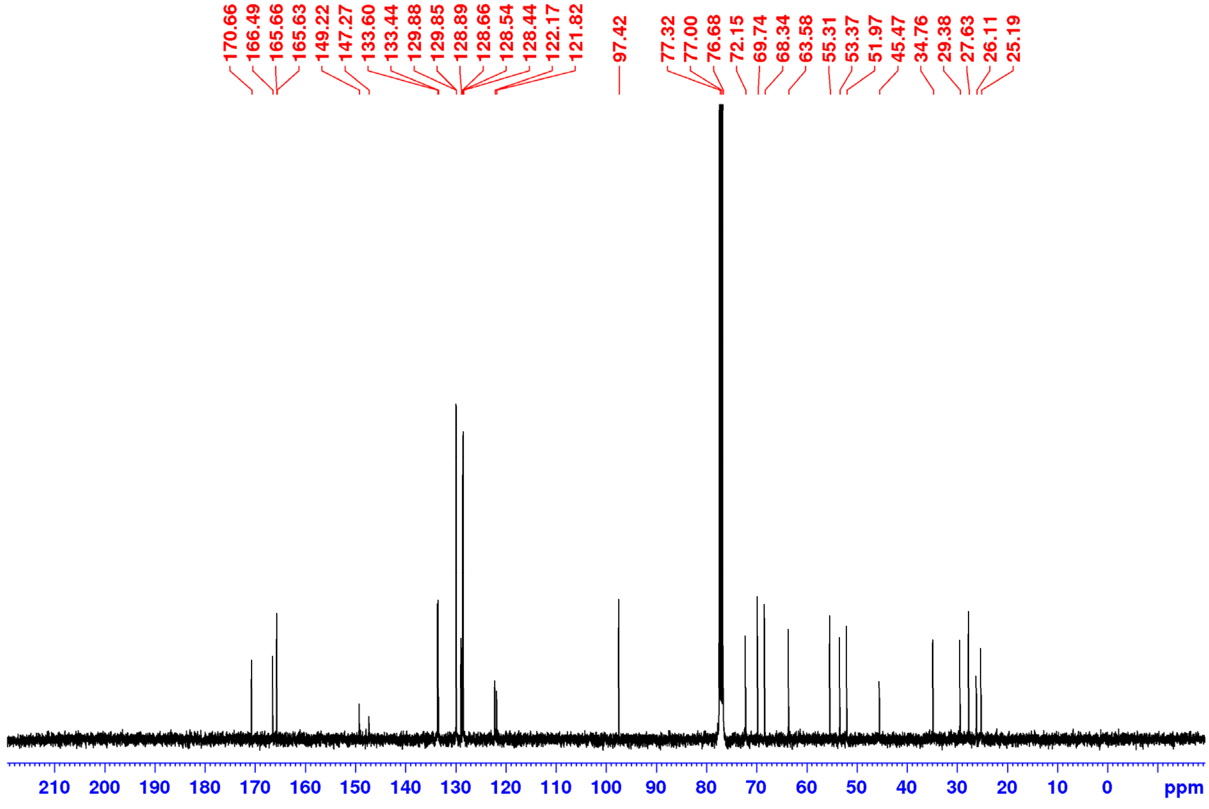

^{1}H NMR and ^{13}C NMR spectra of compound **24** in $CDCl_3$

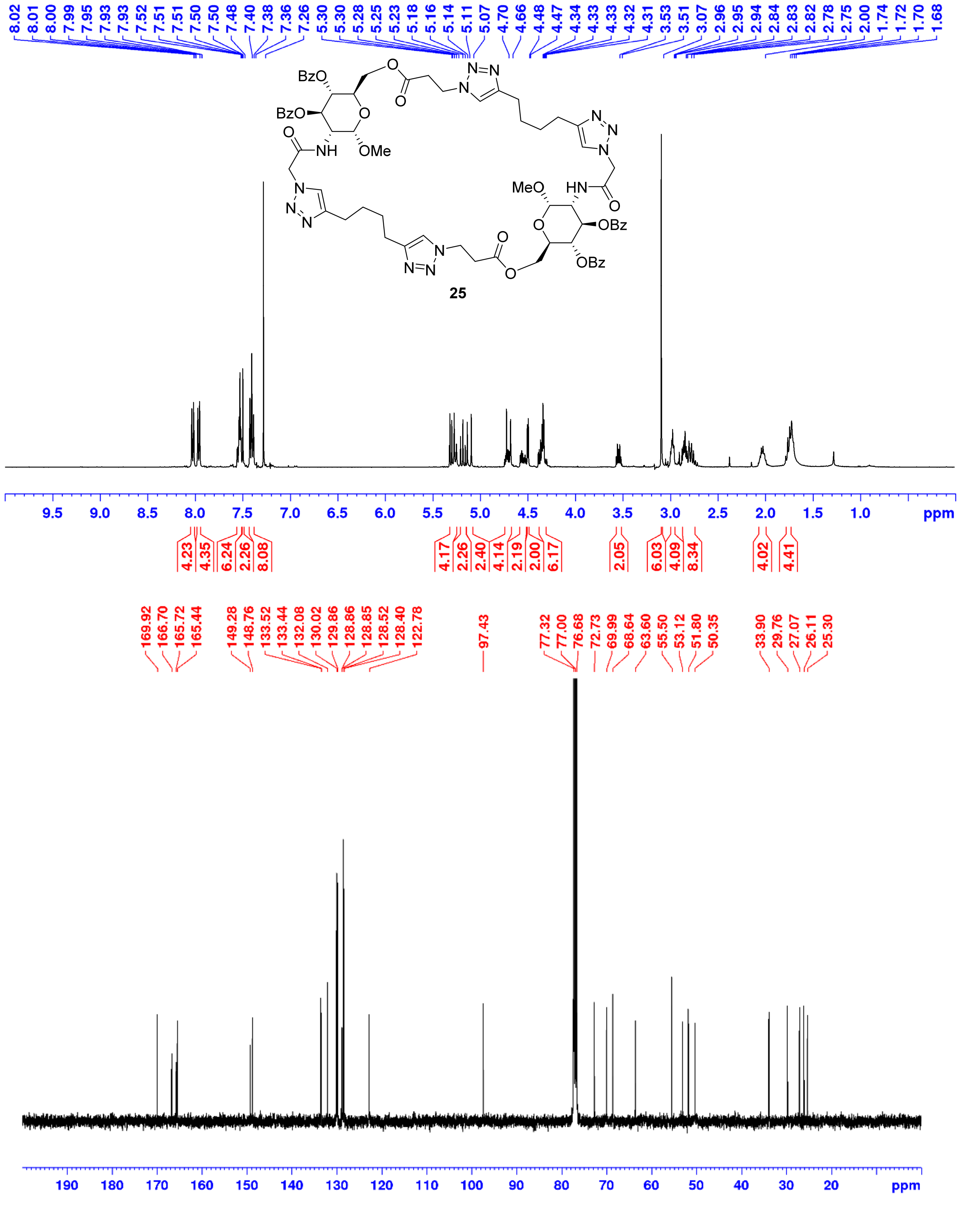

^{1}H NMR and ^{13}C NMR spectra of compound **25** in $CDCl_3$

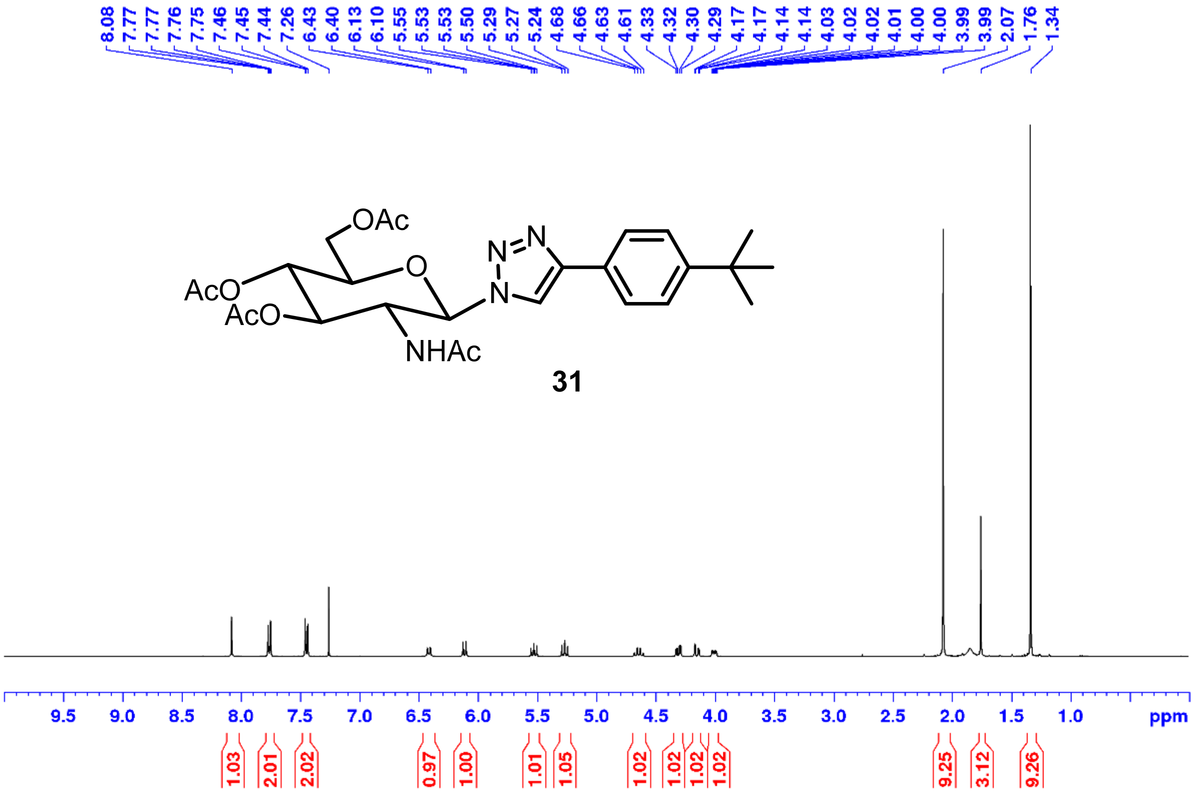

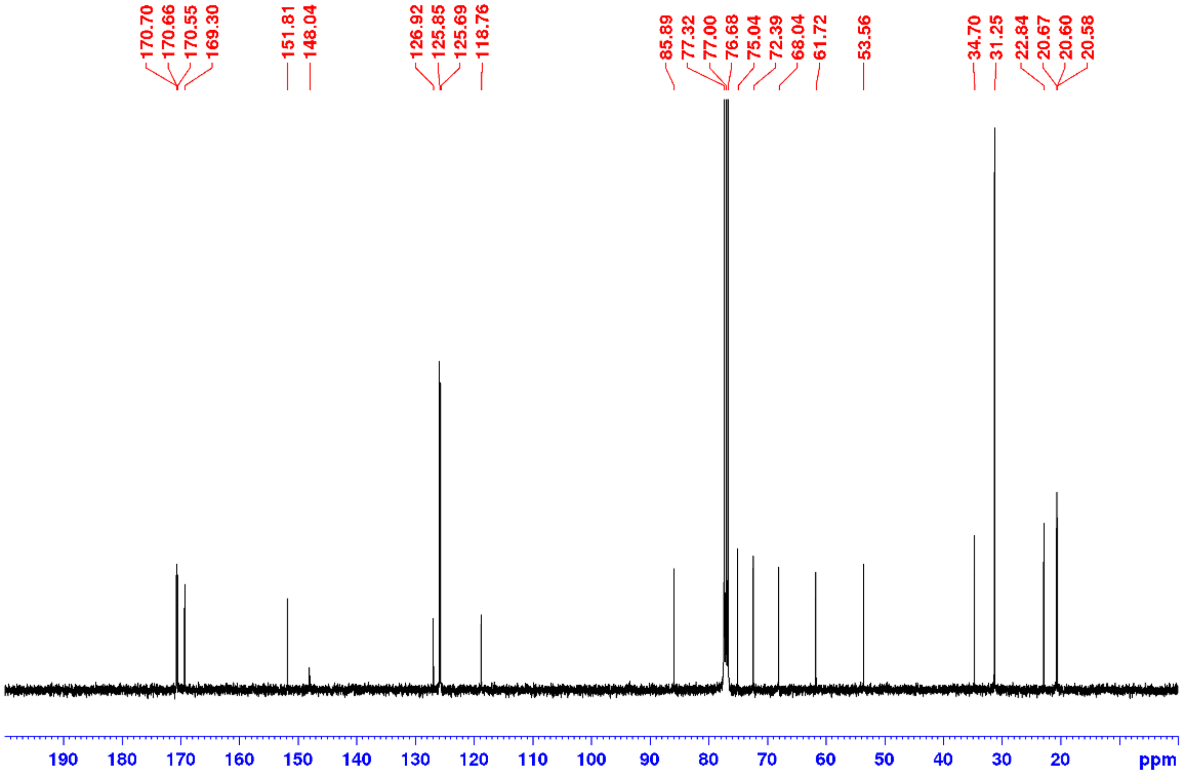

^{1}H NMR and ^{13}C NMR spectra of compound **31** in $CDCl_3$

III. 2D HSQC and COSY NMR Spectra of Selected Compounds

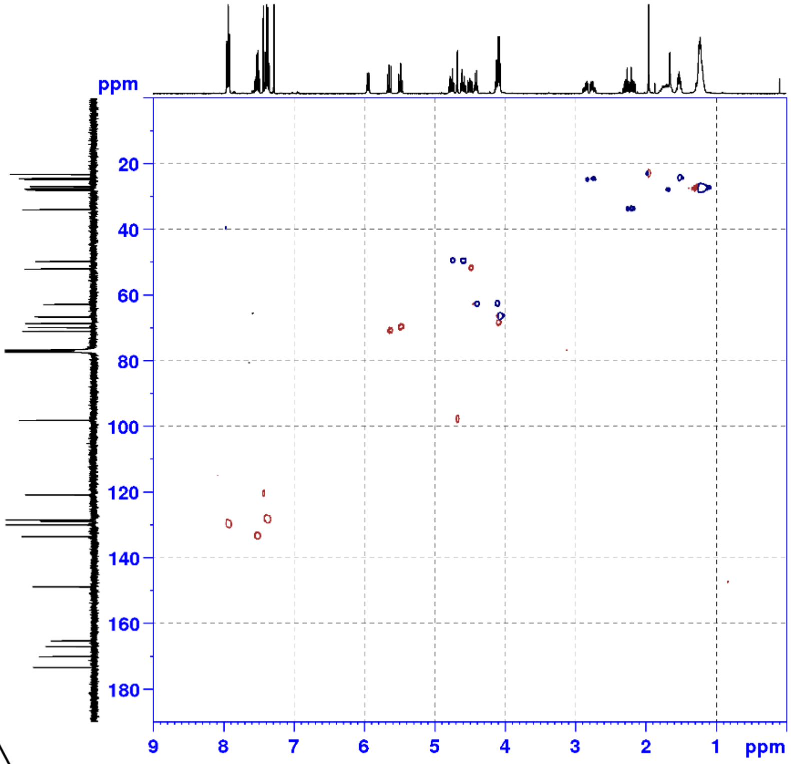

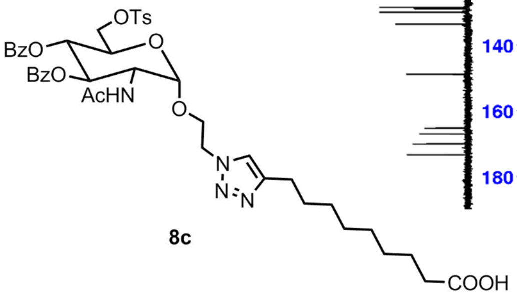

8c

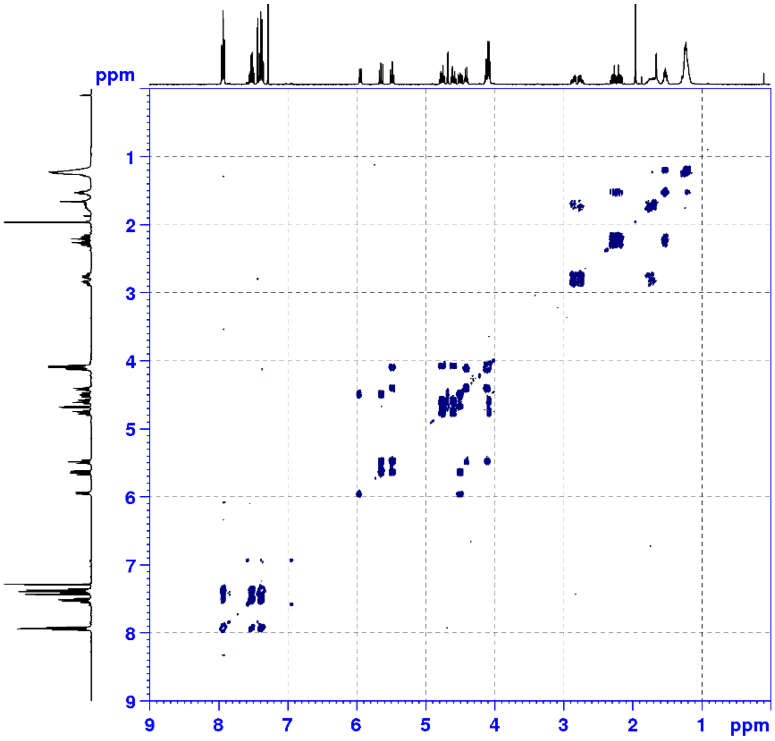

HSQC and COSY NMR spectra of compound **8c** in $CDCl_3$

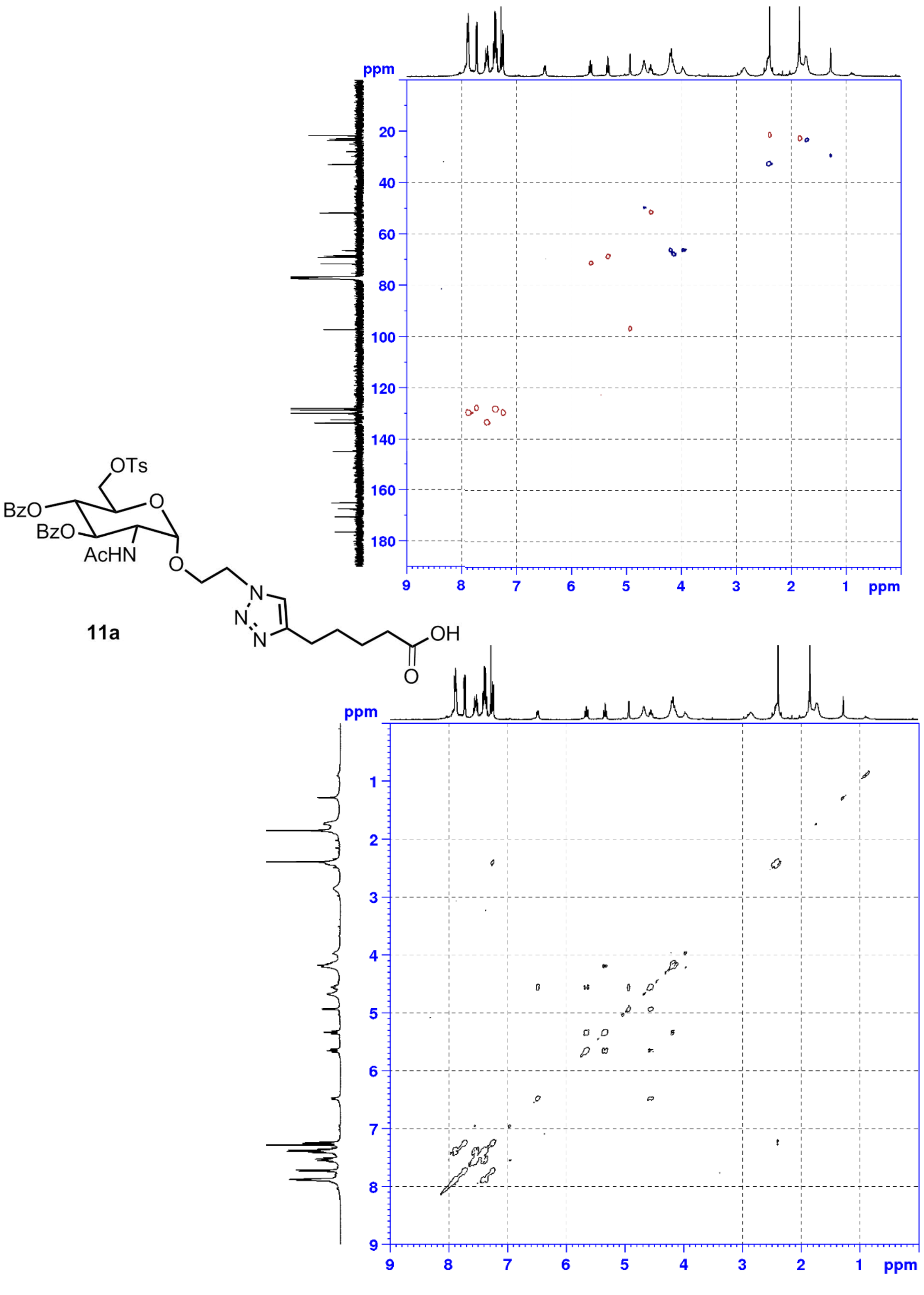

HSQC and COSY NMR spectra of compound **11a** in $CDCl_3$

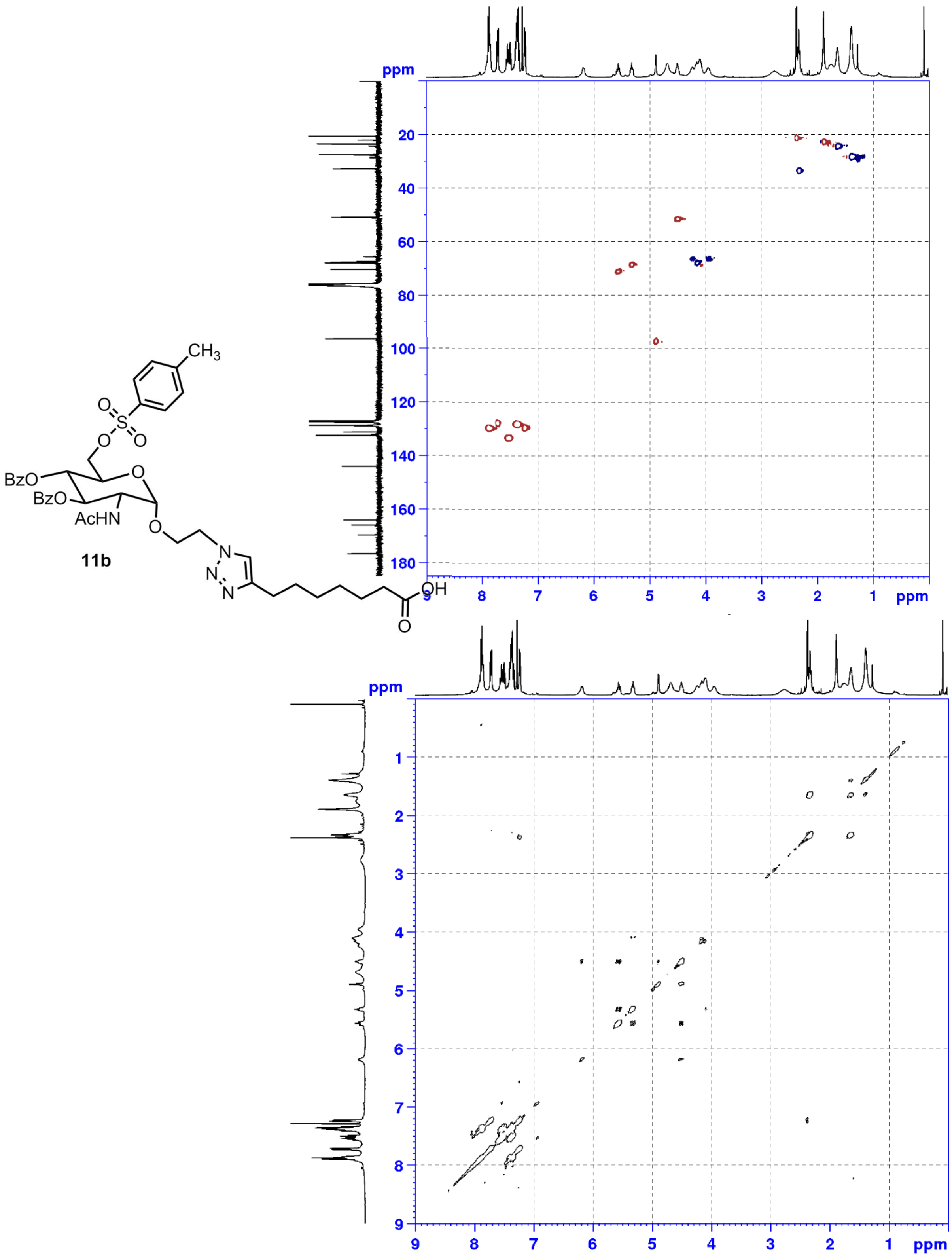

HSQC and COSY NMR spectra of compound **11b** in $CDCl_3$

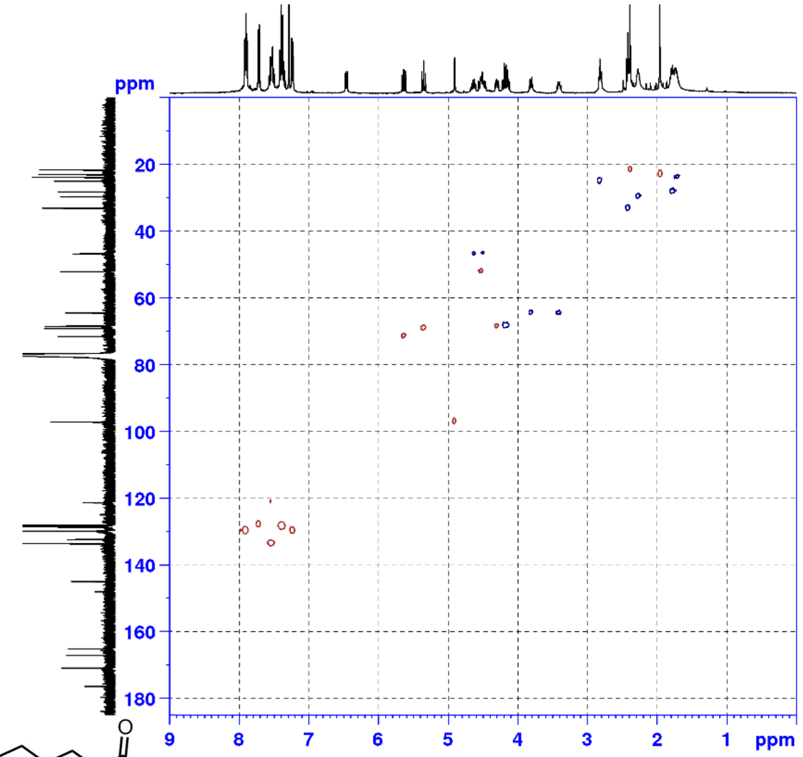

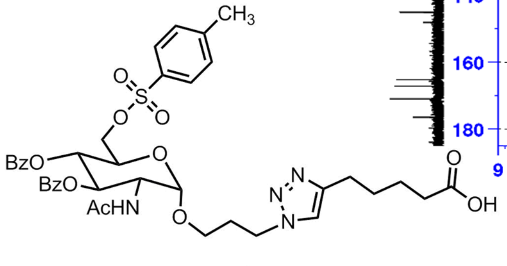

12a

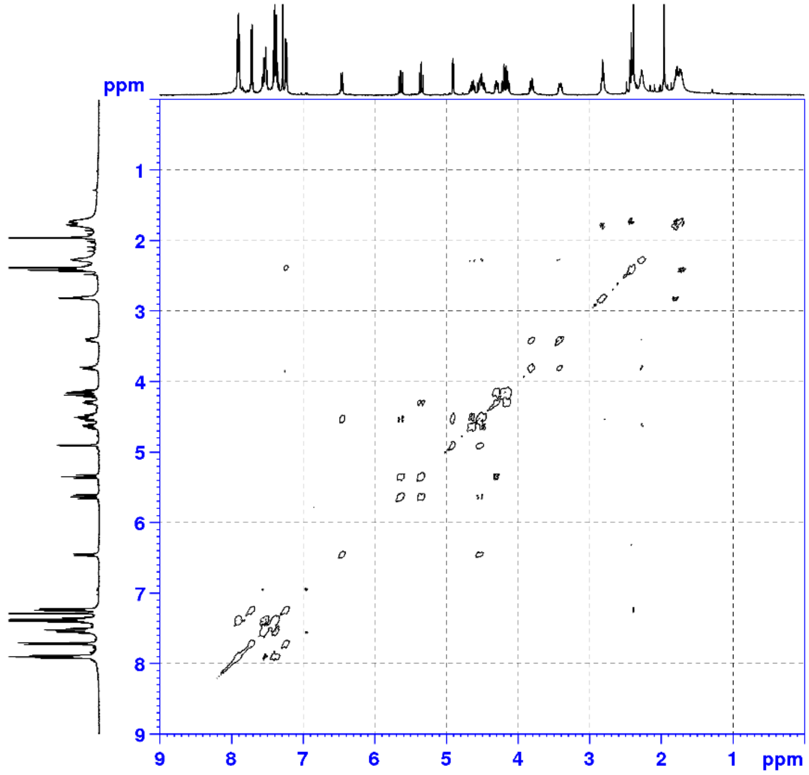

HSQC and COSY NMR spectra of compound **12a** in $CDCl_3$

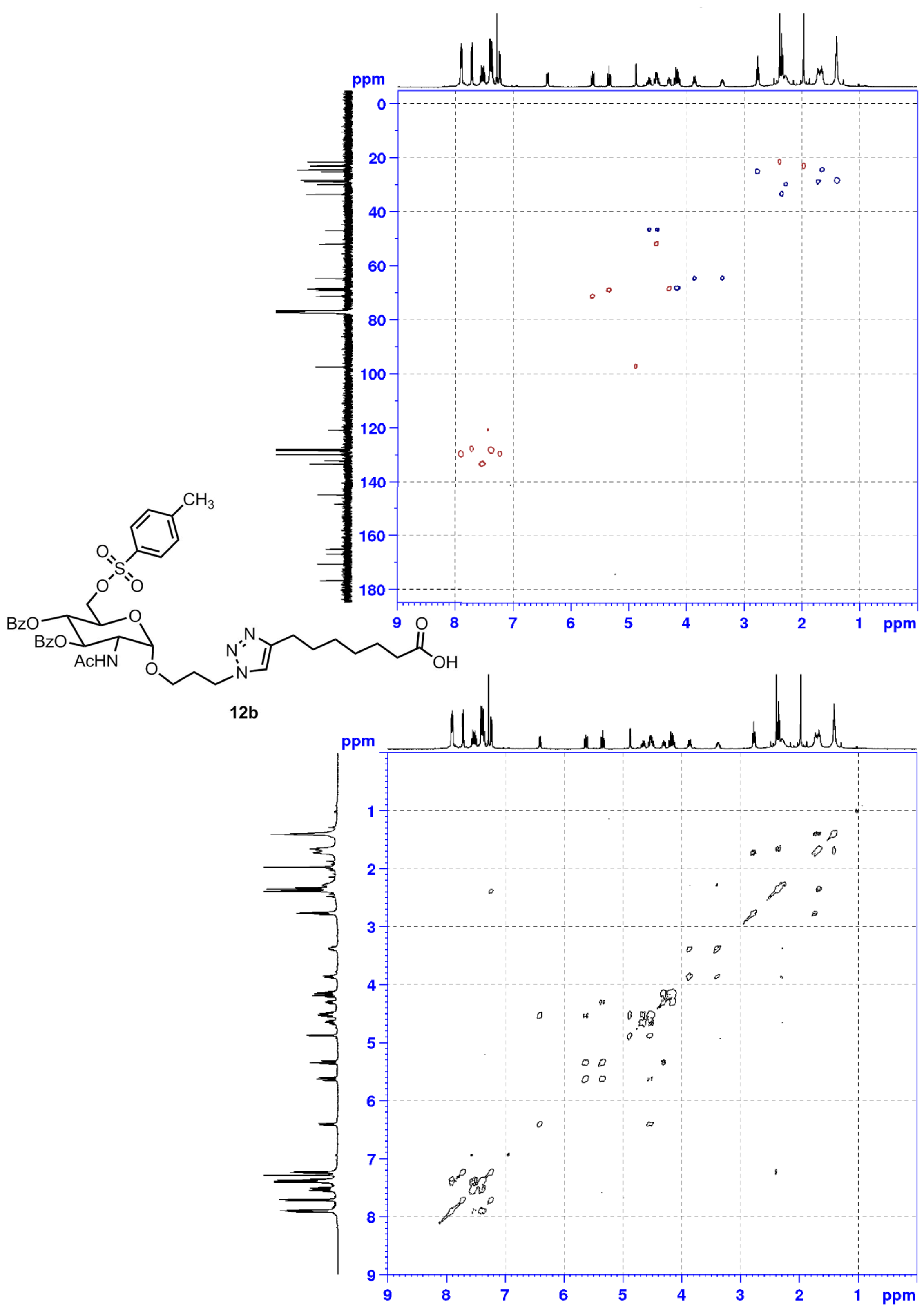

HSQC and COSY NMR spectra of compound **12b** in $CDCl_3$

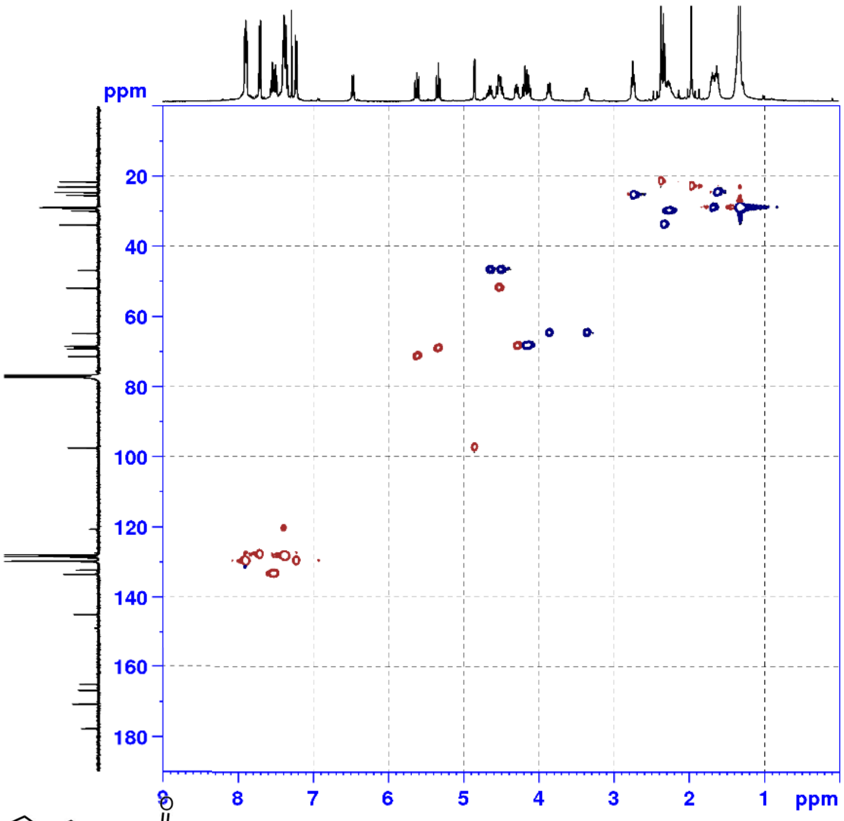

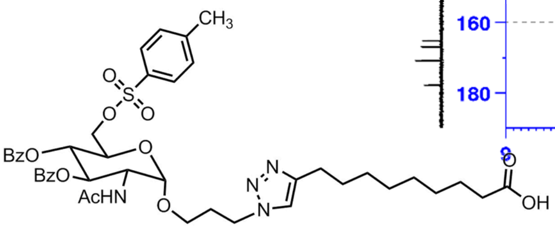

12c

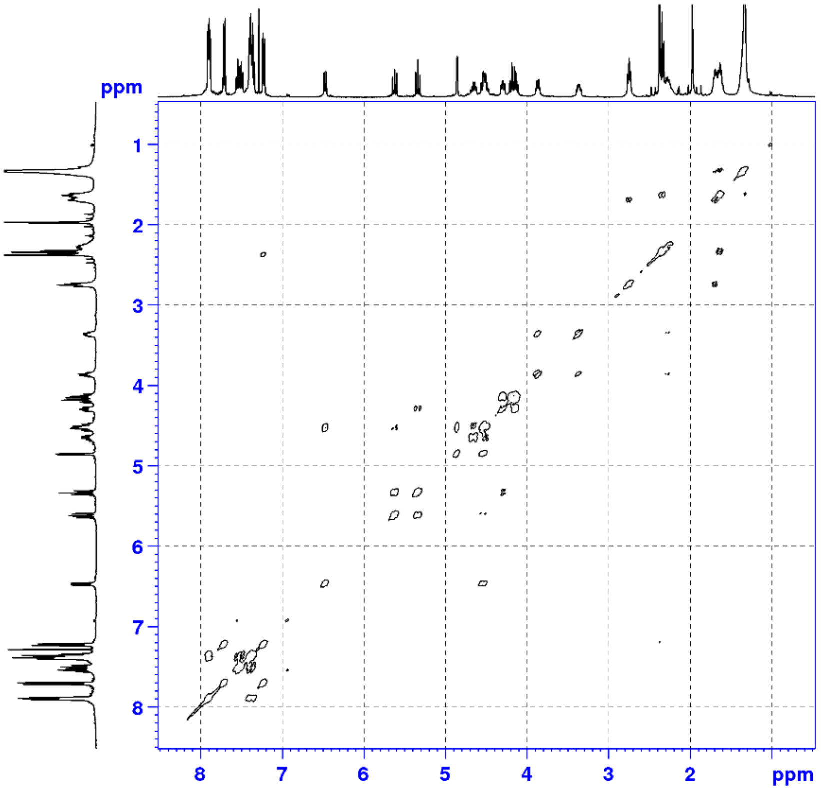

HSQC and COSY NMR spectra of compound **12c** in $CDCl_3$

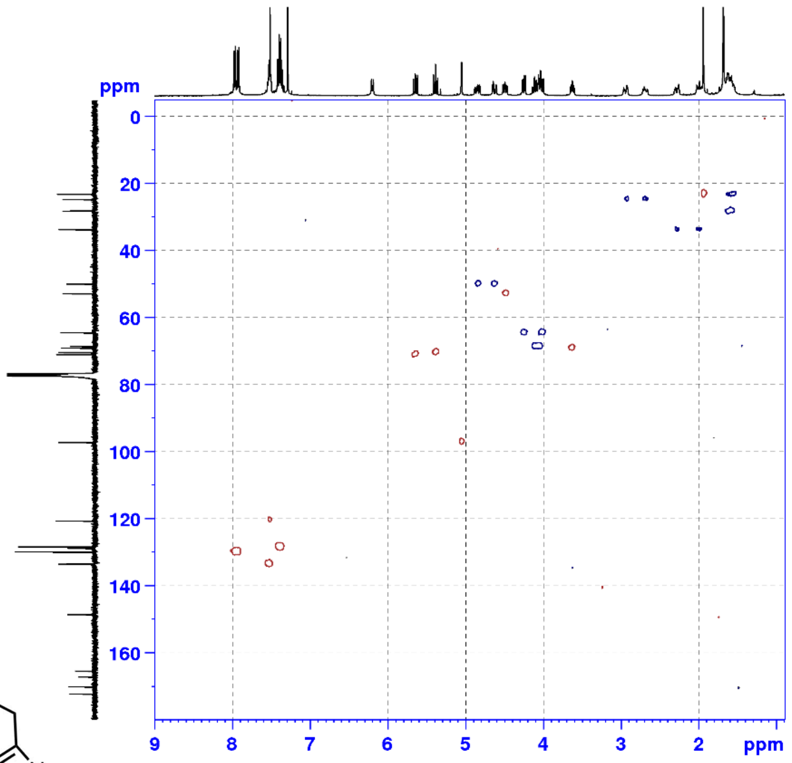

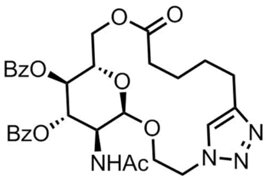

LM24, 13a

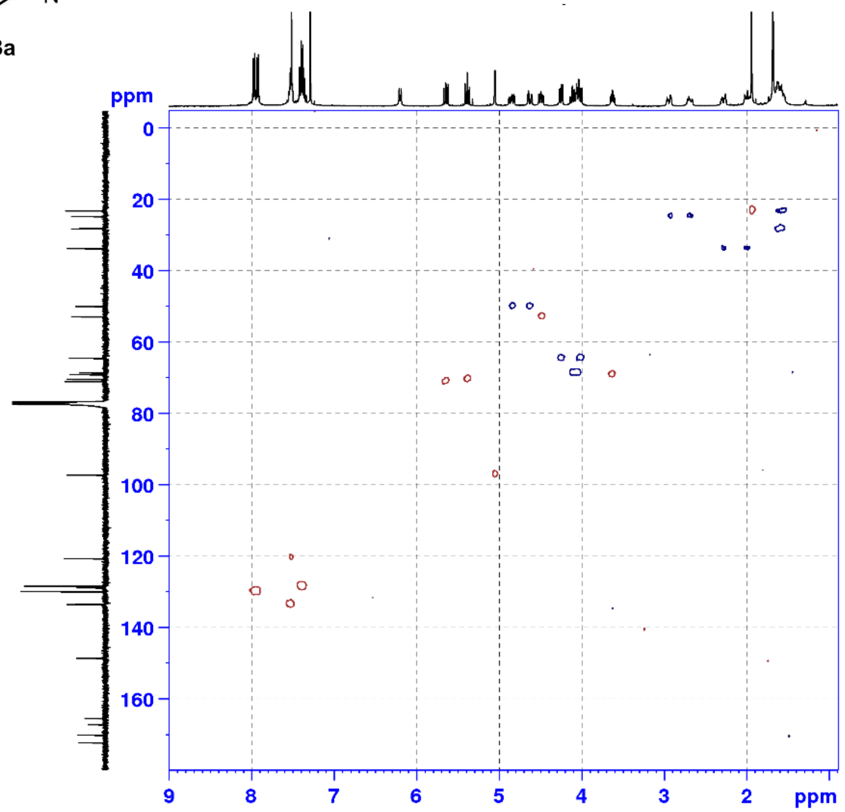

HSQC and COSY NMR spectra of compound **13a** in $CDCl_3$

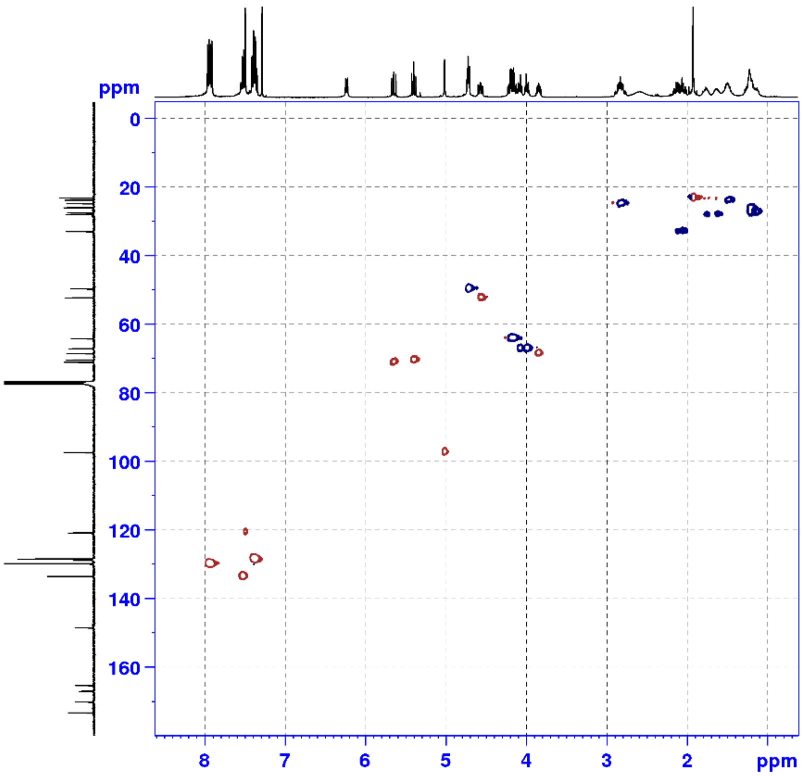

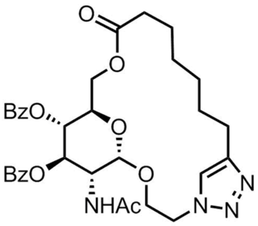

LM26, 13b

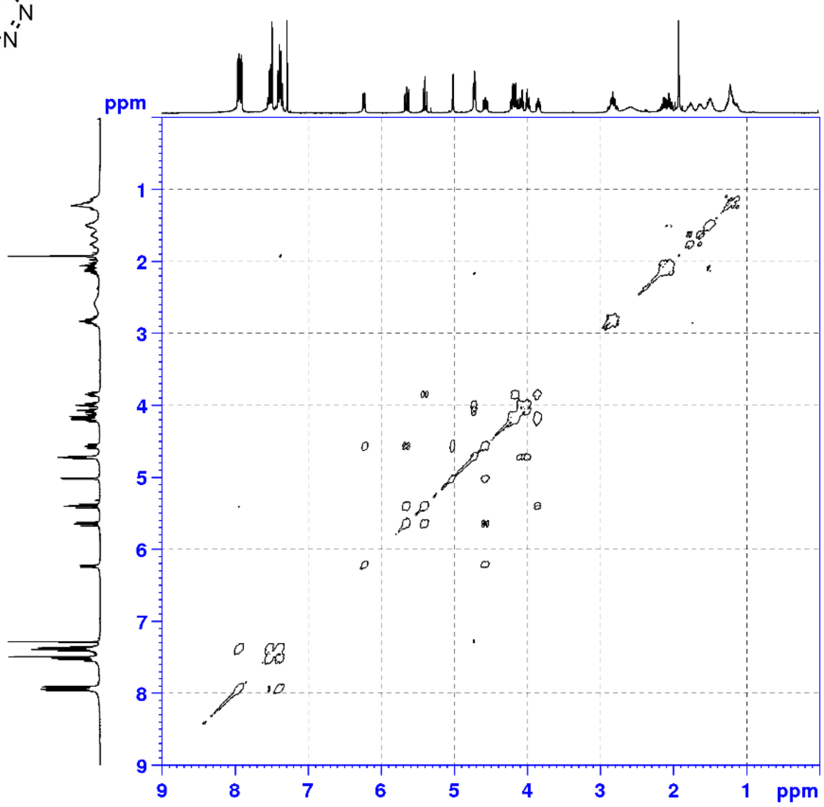

HSQC and COSY NMR spectra of compound **13b** in $CDCl_3$

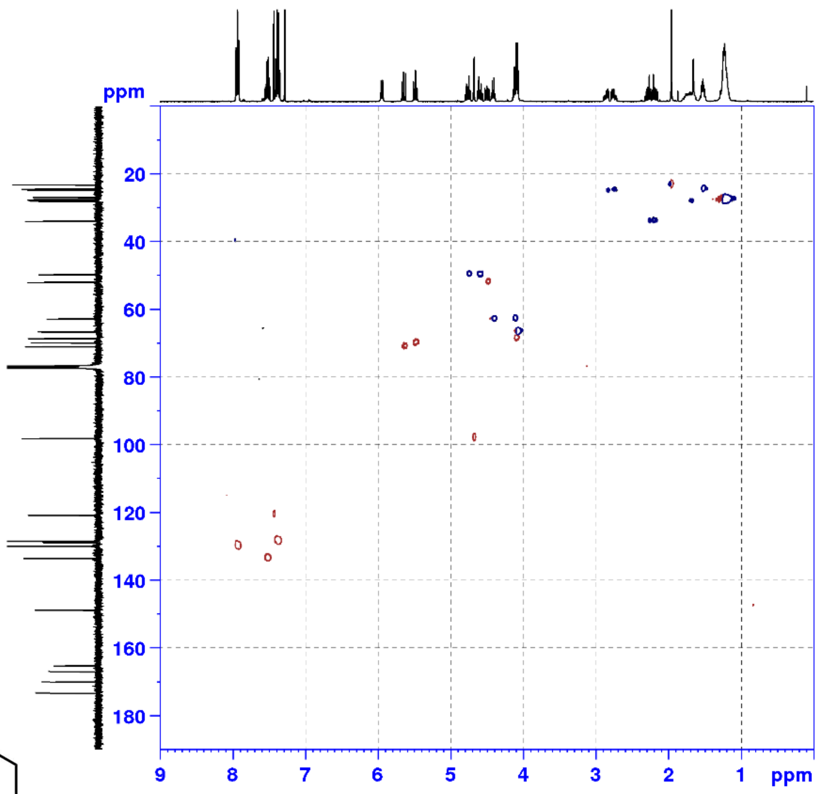

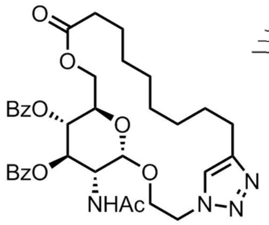

LM28, 13c

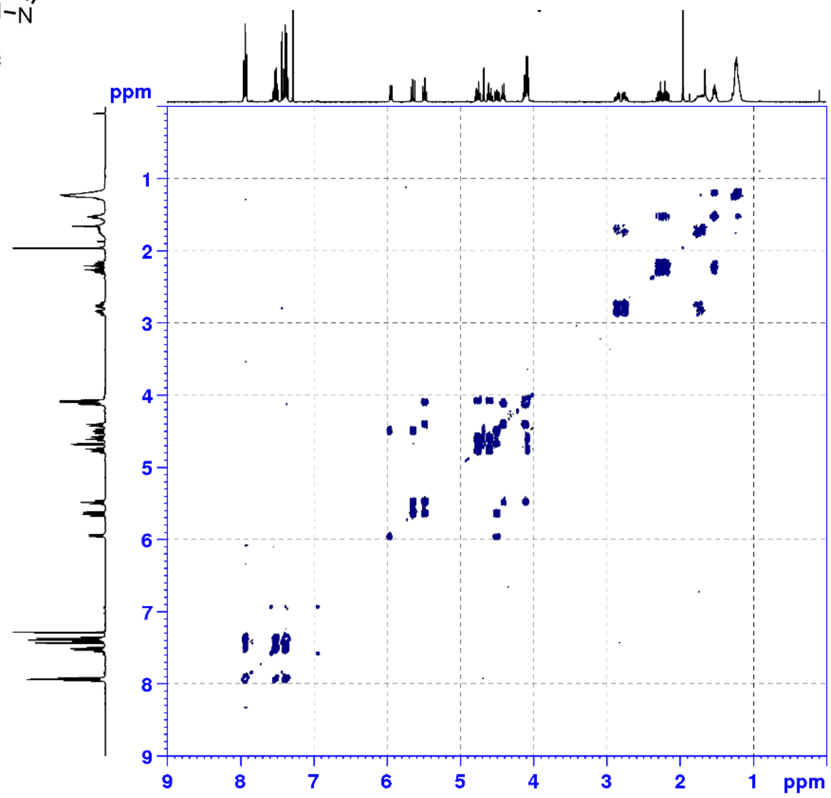

^{1}H NMR and ^{13}C NMR spectra of compound **13c** in $CDCl_3$

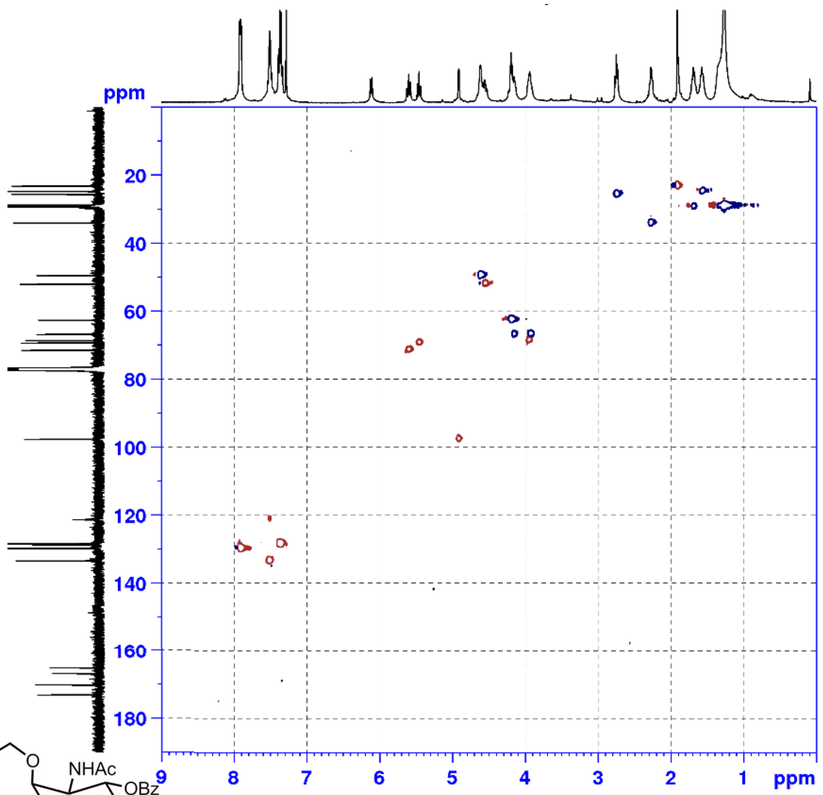

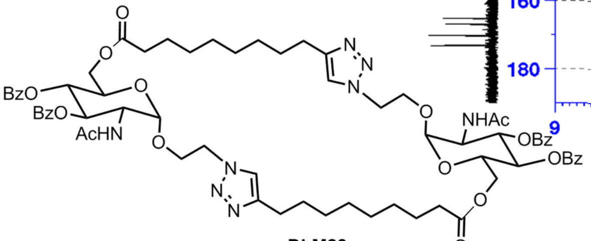

DLM28

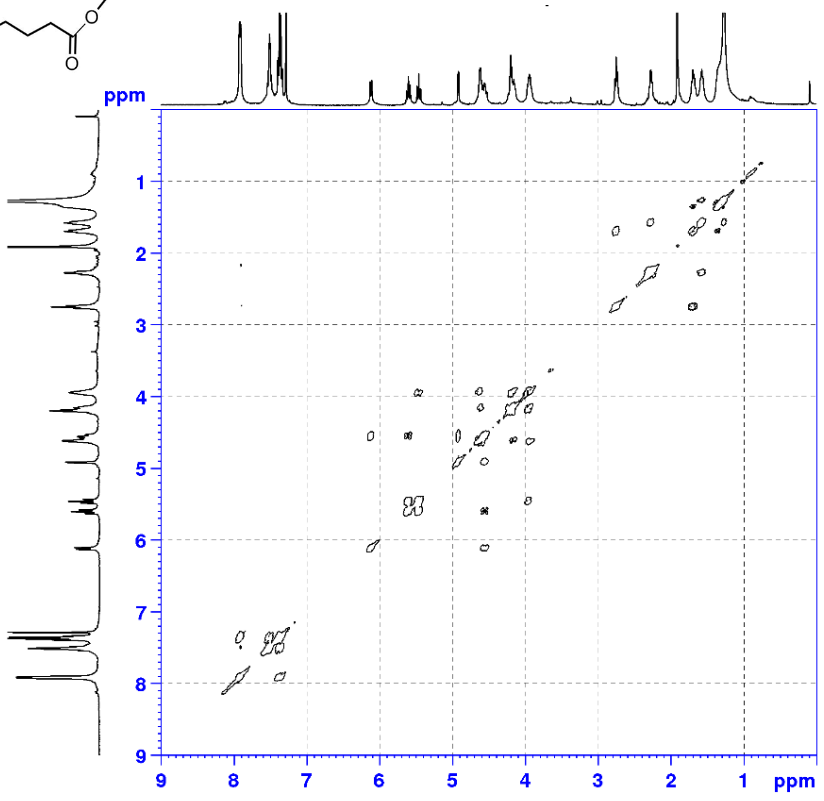

HSQC and COSY NMR spectra of compound **DLM28** in $CDCl_3$

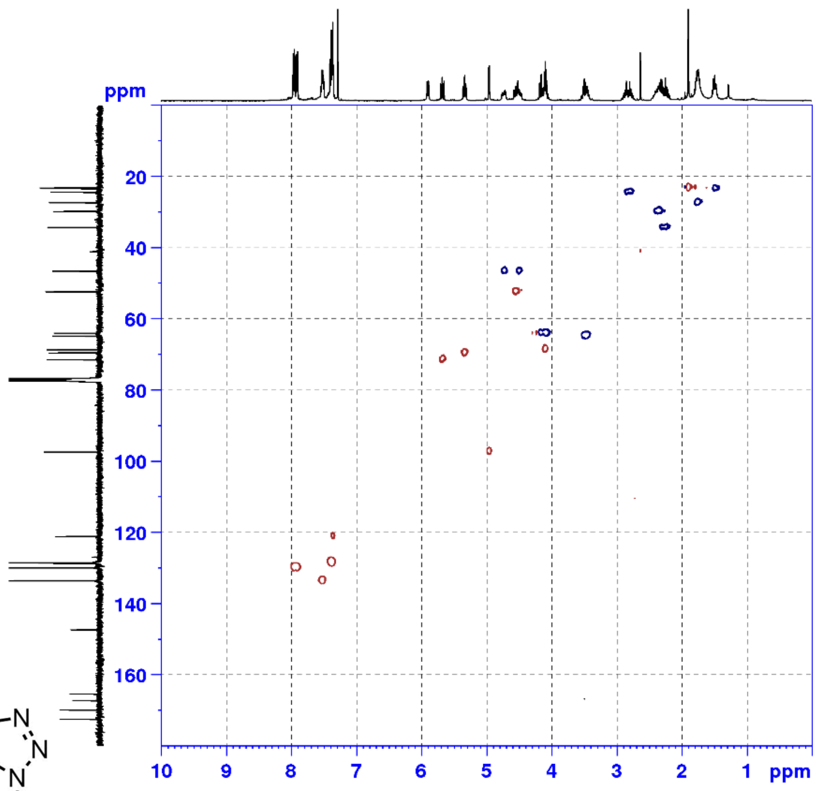

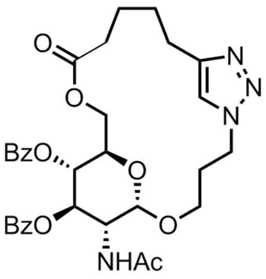

LM34, 14a

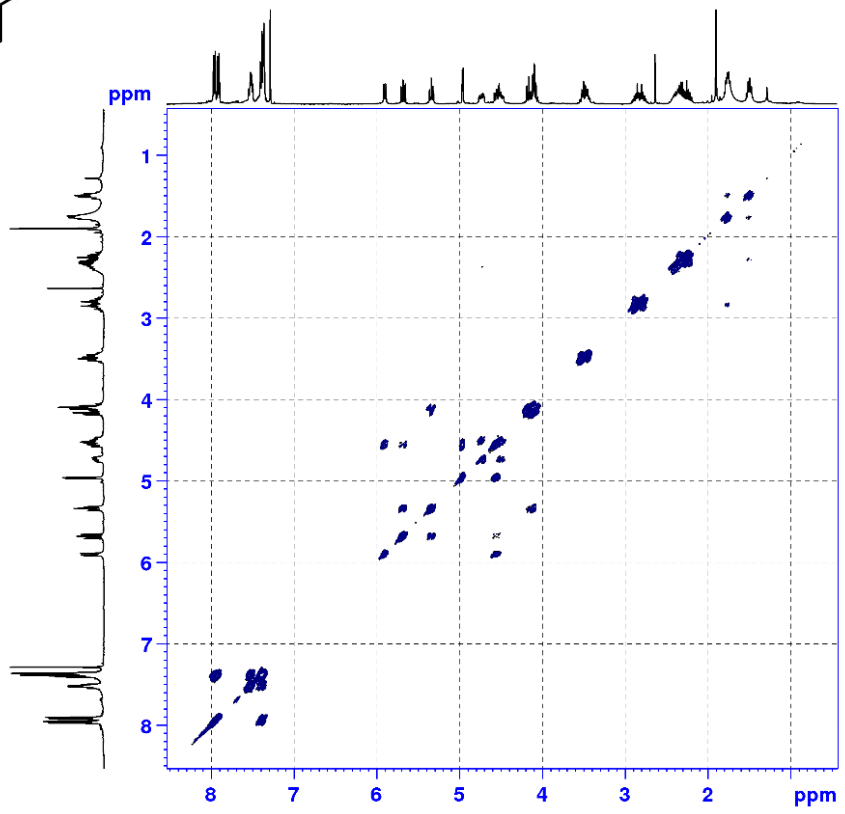

HSQC and COSY NMR spectra of compound **14a** in $CDCl_3$

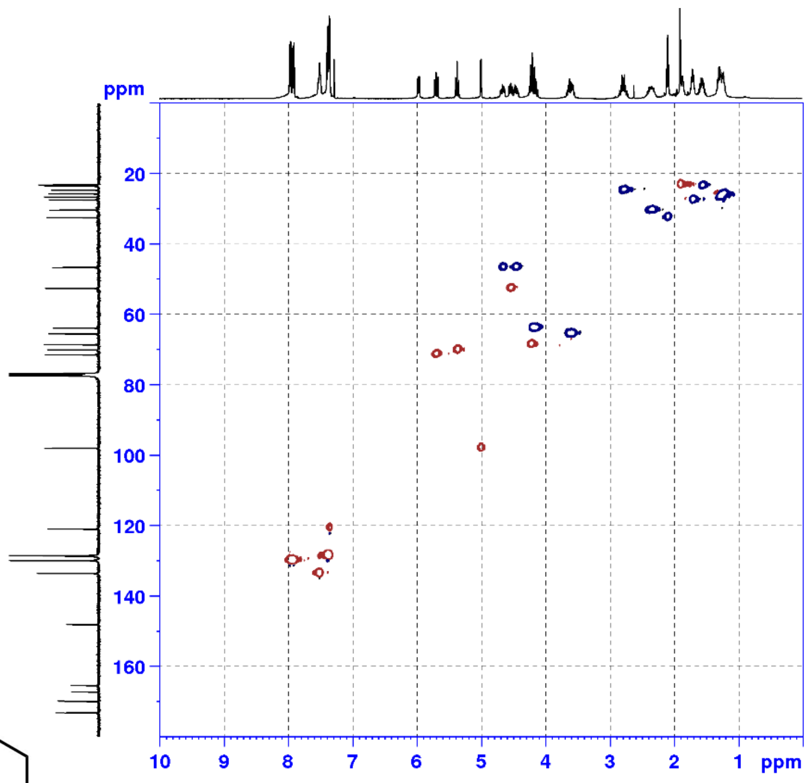

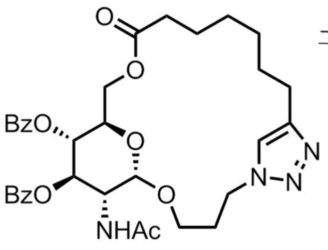

LM36, 14b

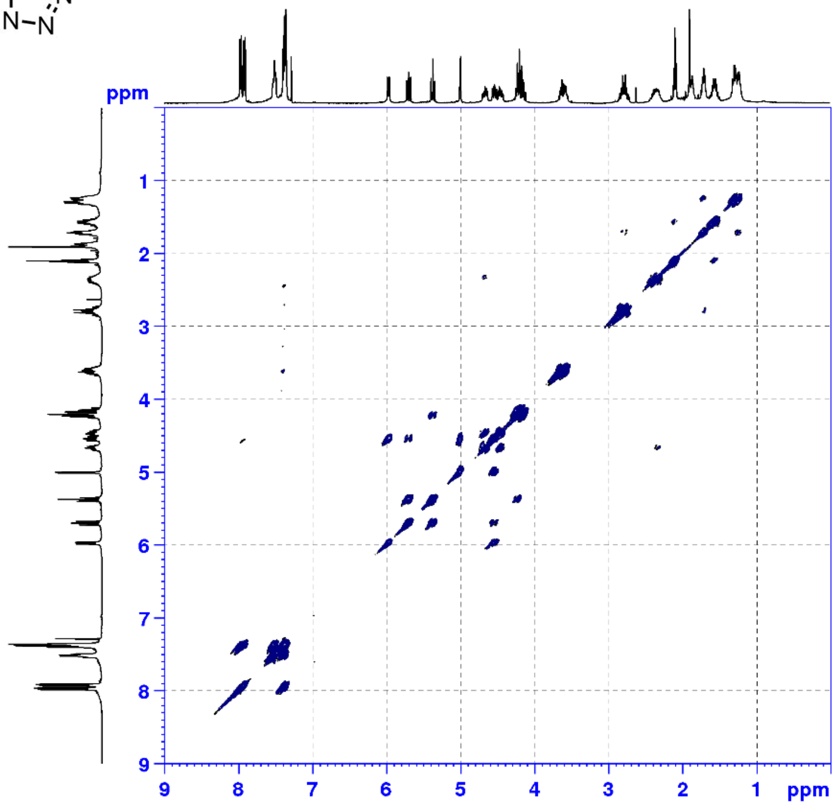

HSQC and COSY NMR spectra of compound **14b** in $CDCl_3$

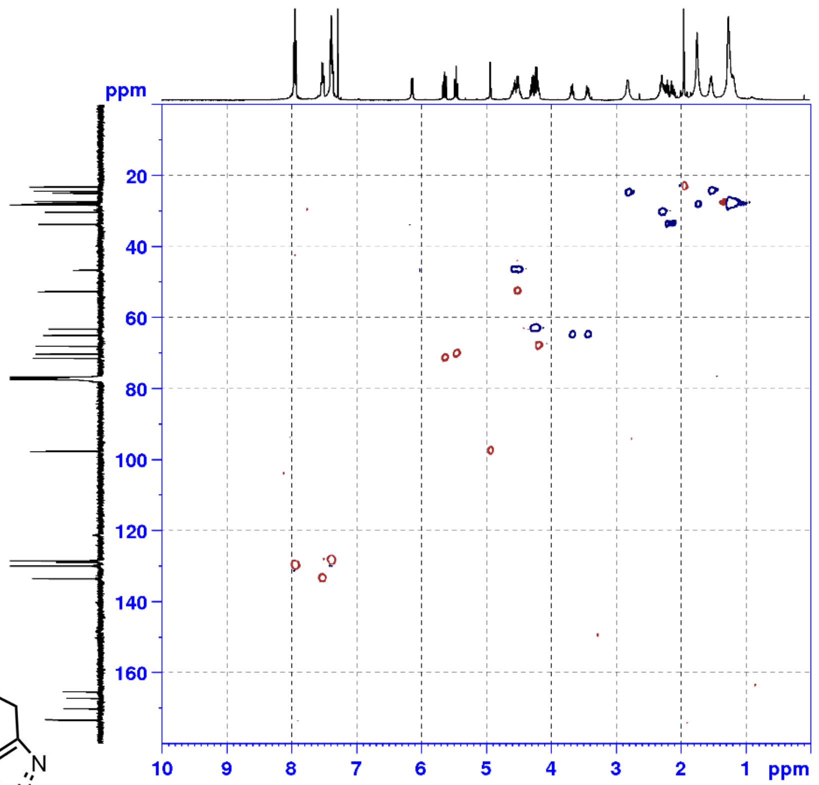

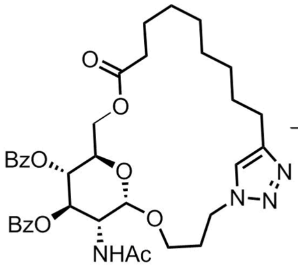

LM38, 14c

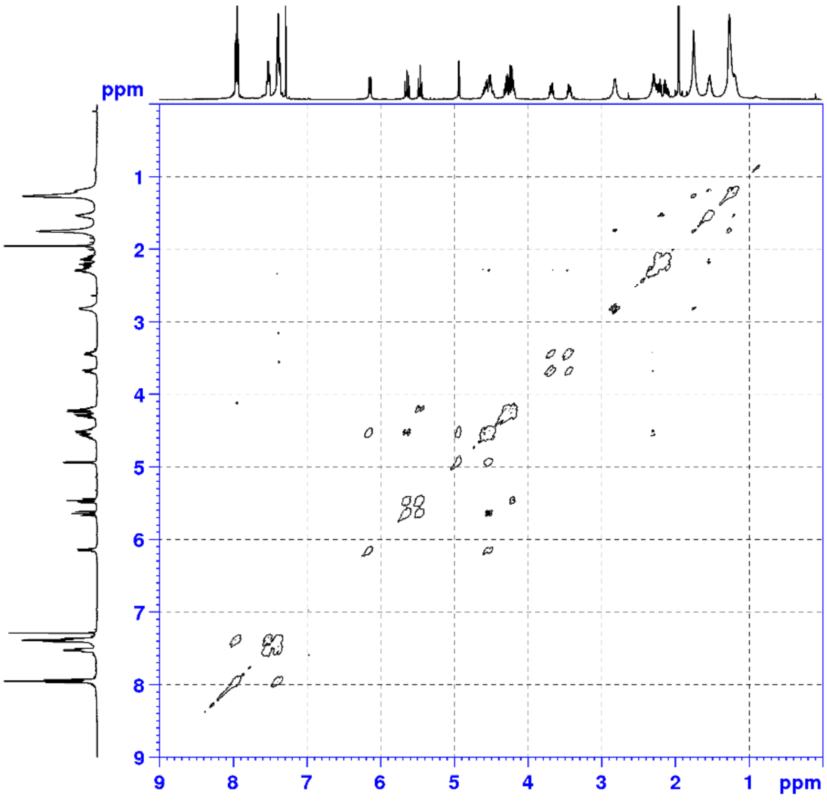

HSQC and COSY NMR spectra of compound **14c** in $CDCl_3$

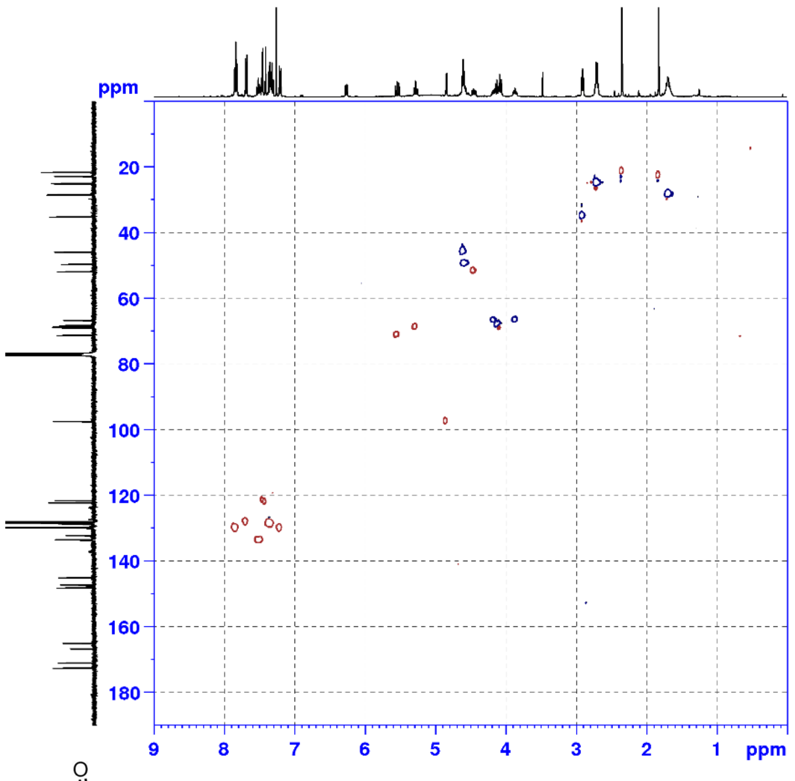

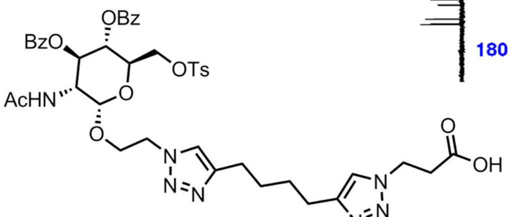

15a

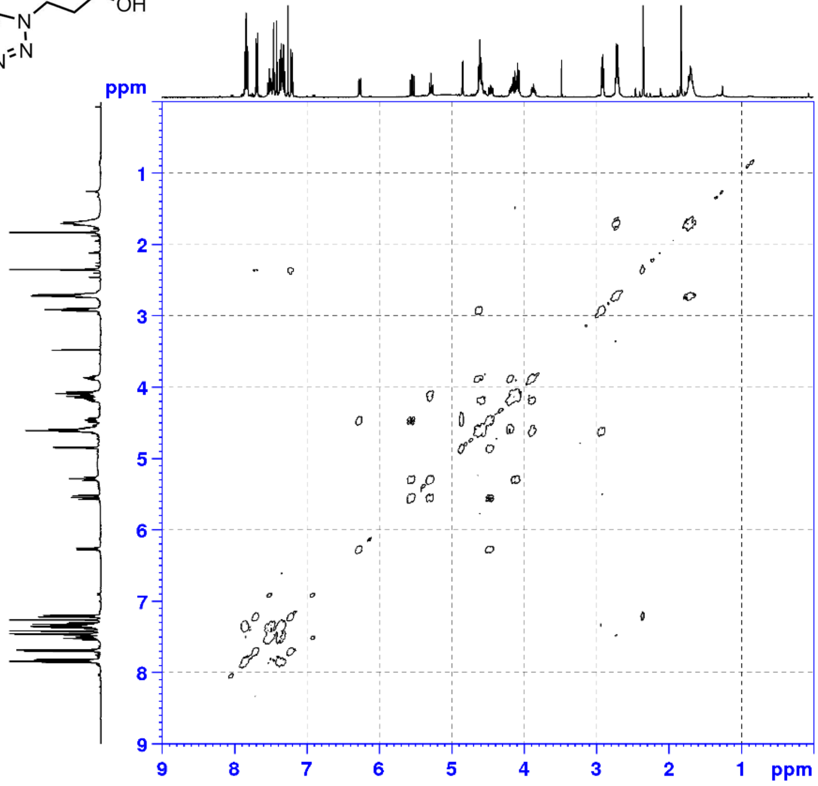

HSQC and COSY NMR spectra of compound **15a** in $CDCl_3$

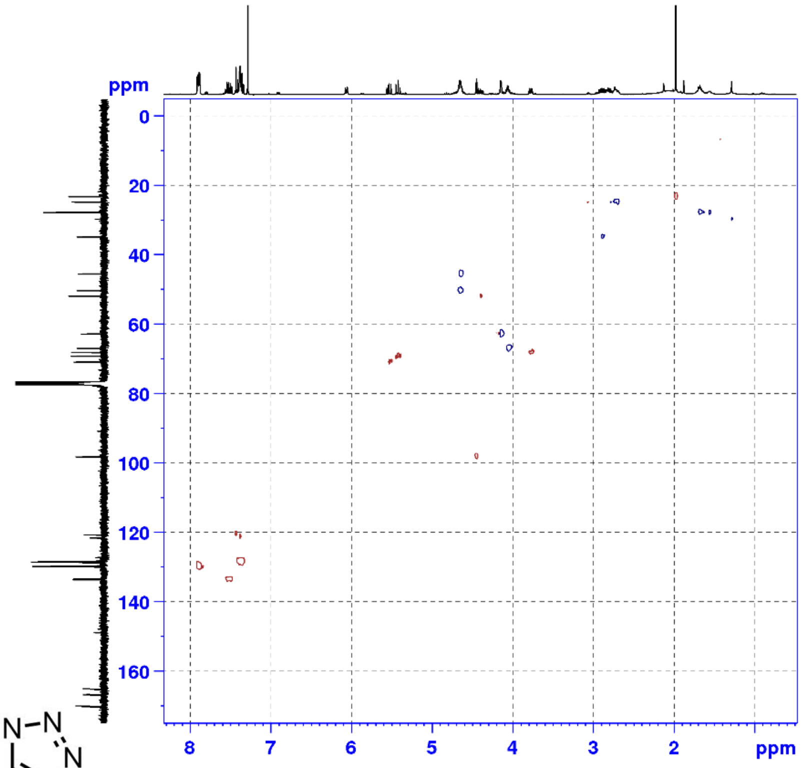

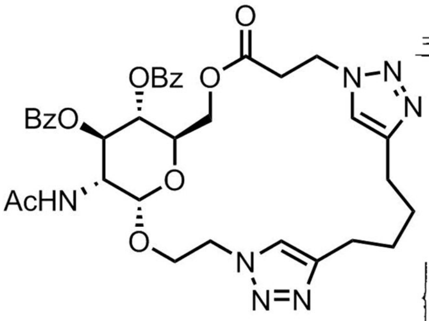

DM24, 16a

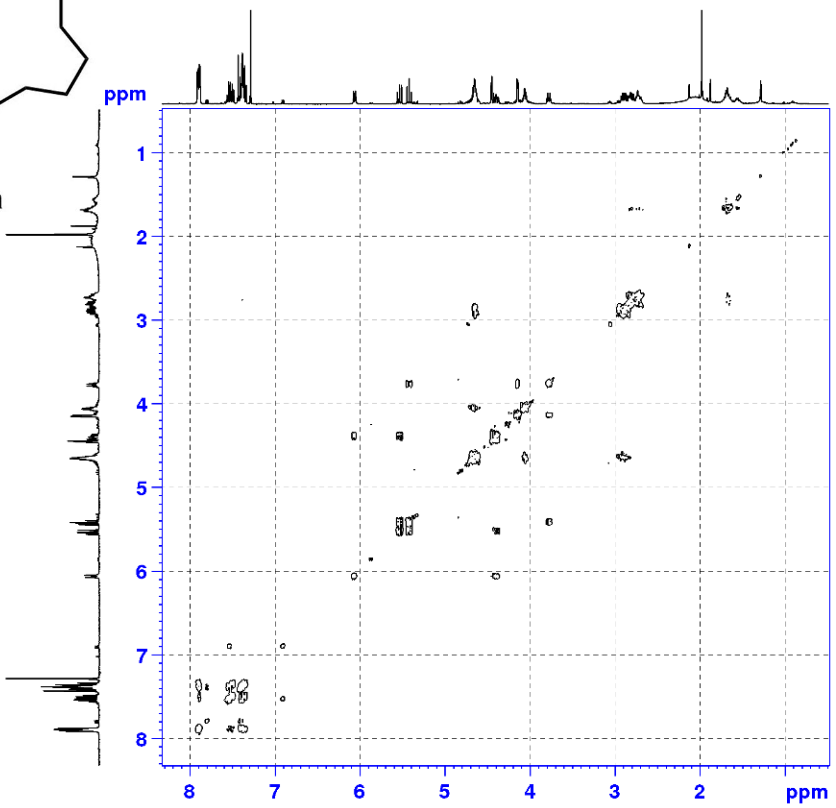

HSQC and COSY NMR spectra of compound **16a** in $CDCl_3$

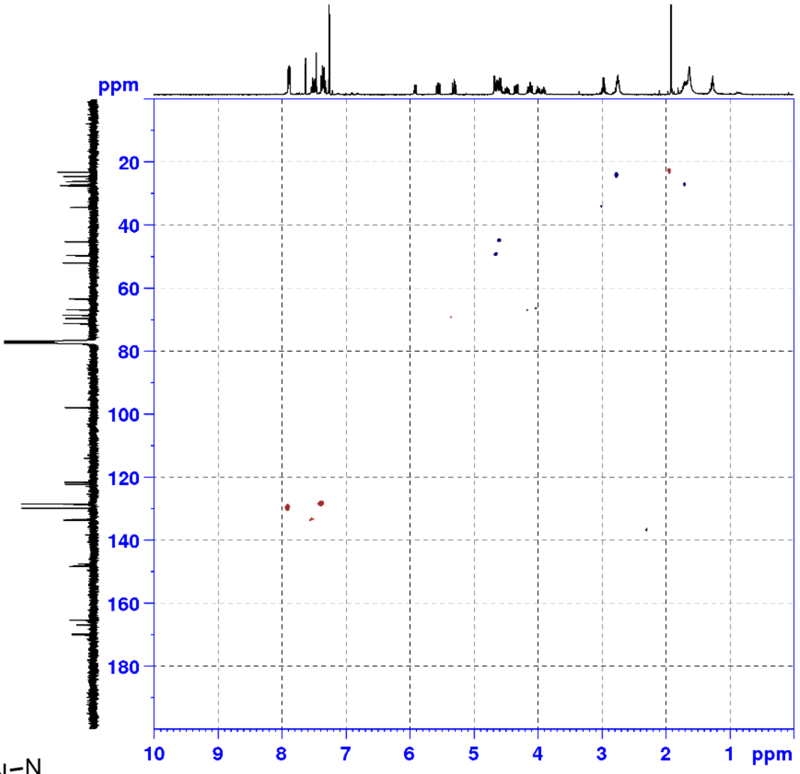

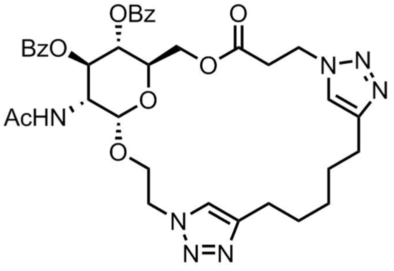

DM25, 16b

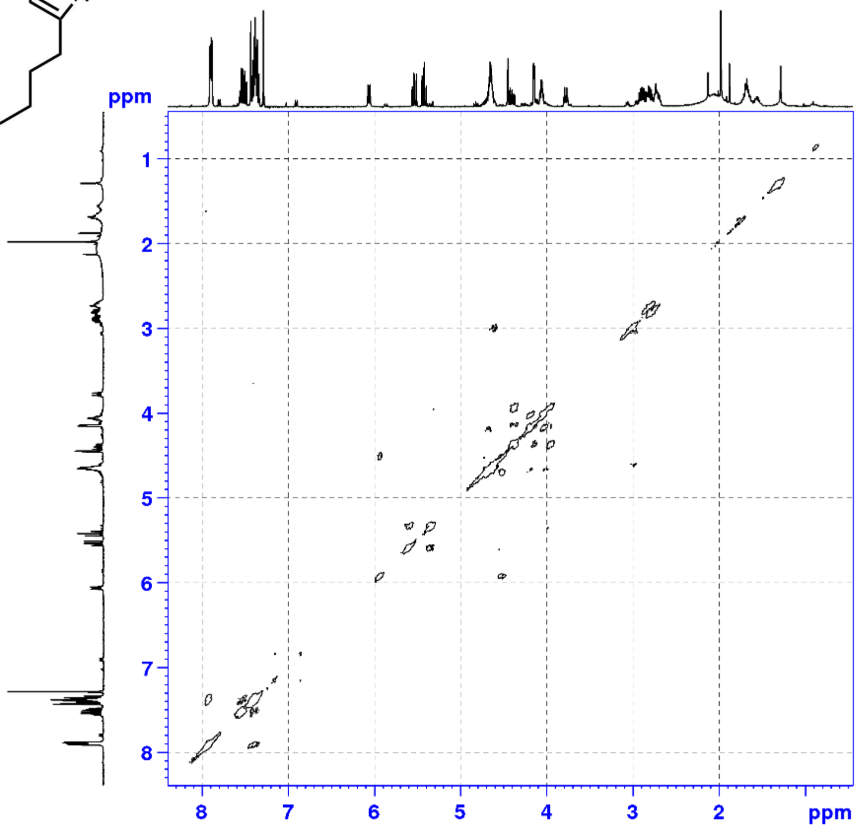

HSQC and COSY NMR spectra of compound **16b** in $CDCl_3$

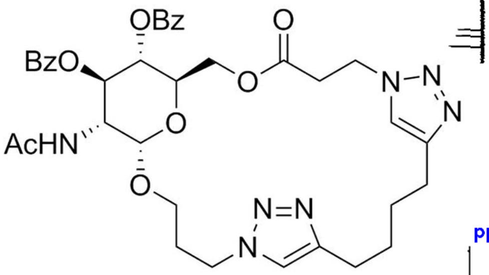

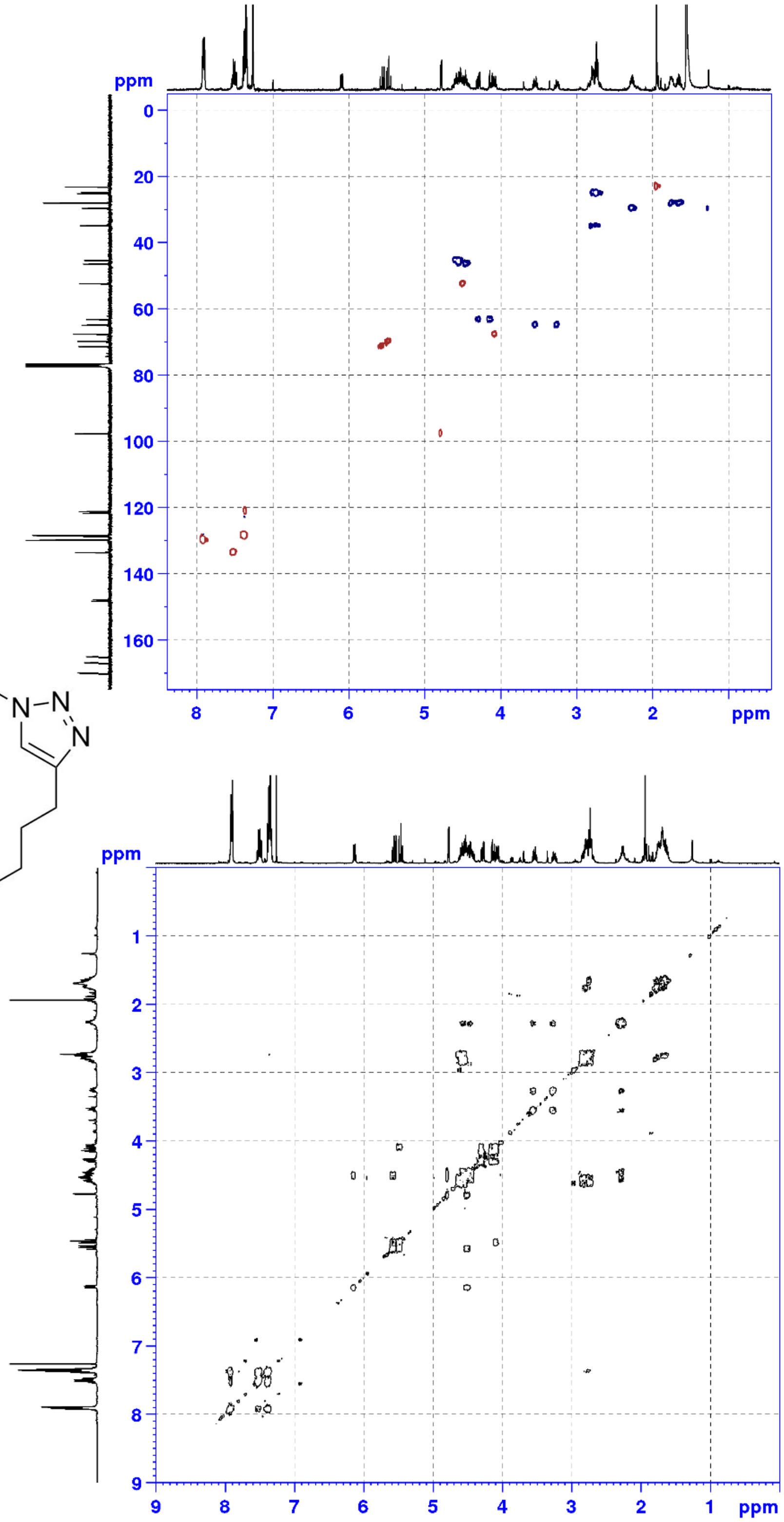

DM34, 16c

HSQC and COSY NMR spectra of compound **16c** in $CDCl_3$

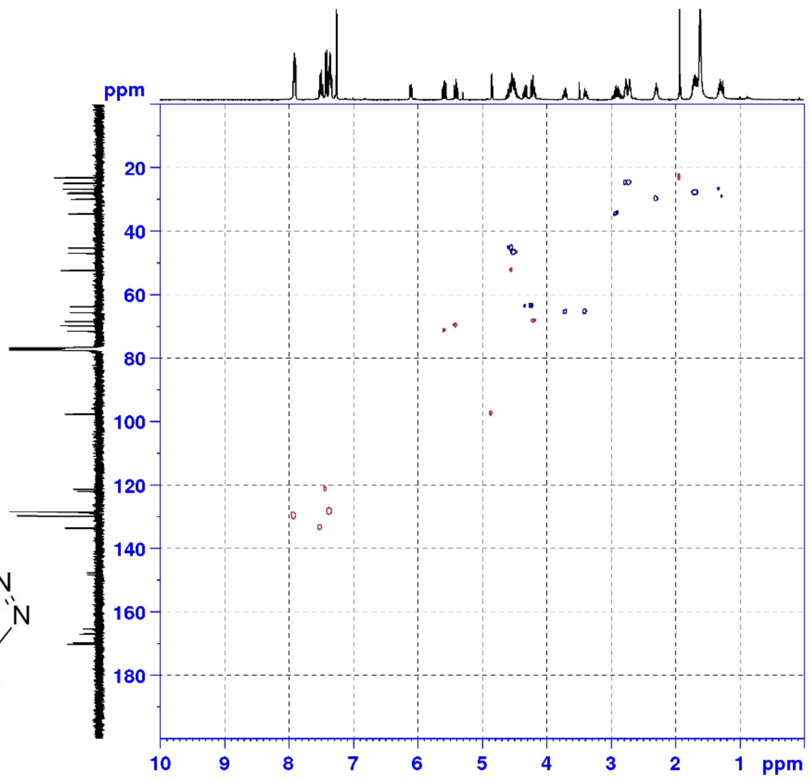

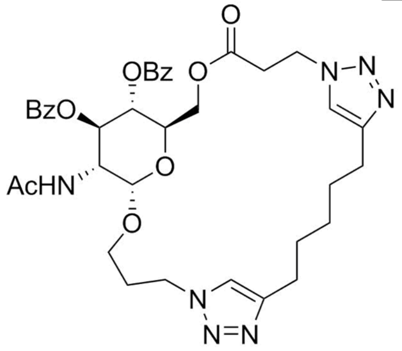

DM35, 16d

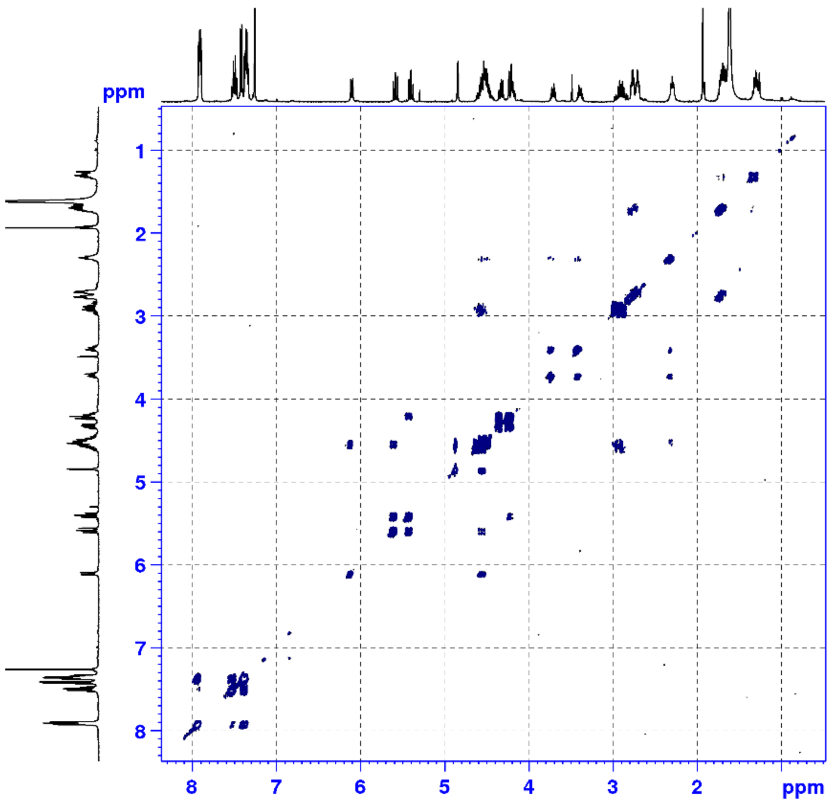

HSQC and COSY NMR spectra of compound **16d** in $CDCl_3$

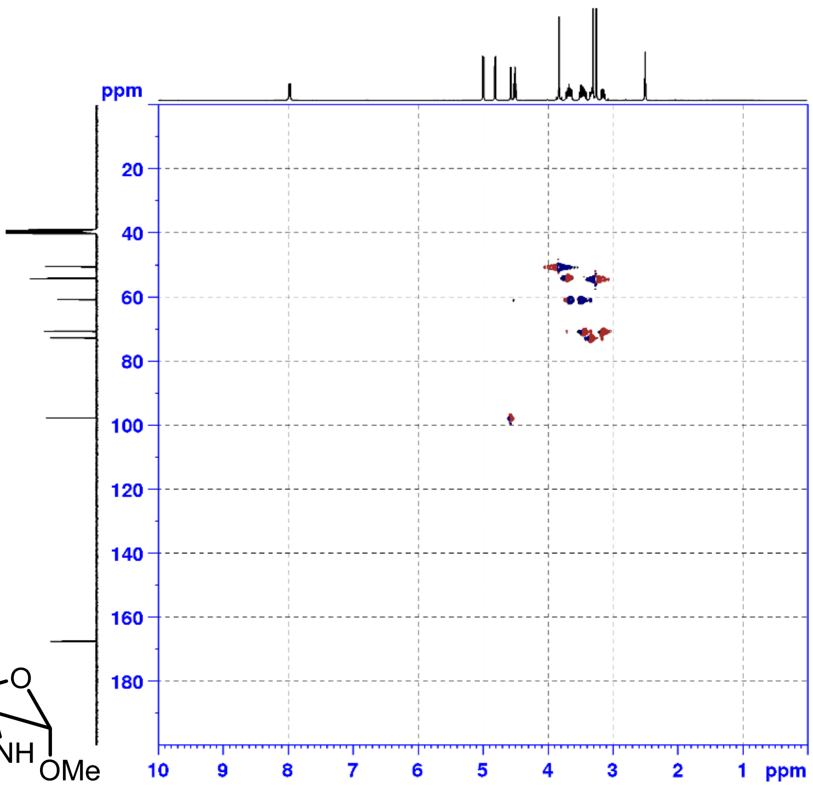

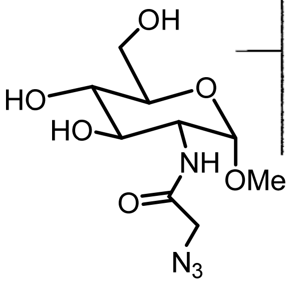

18

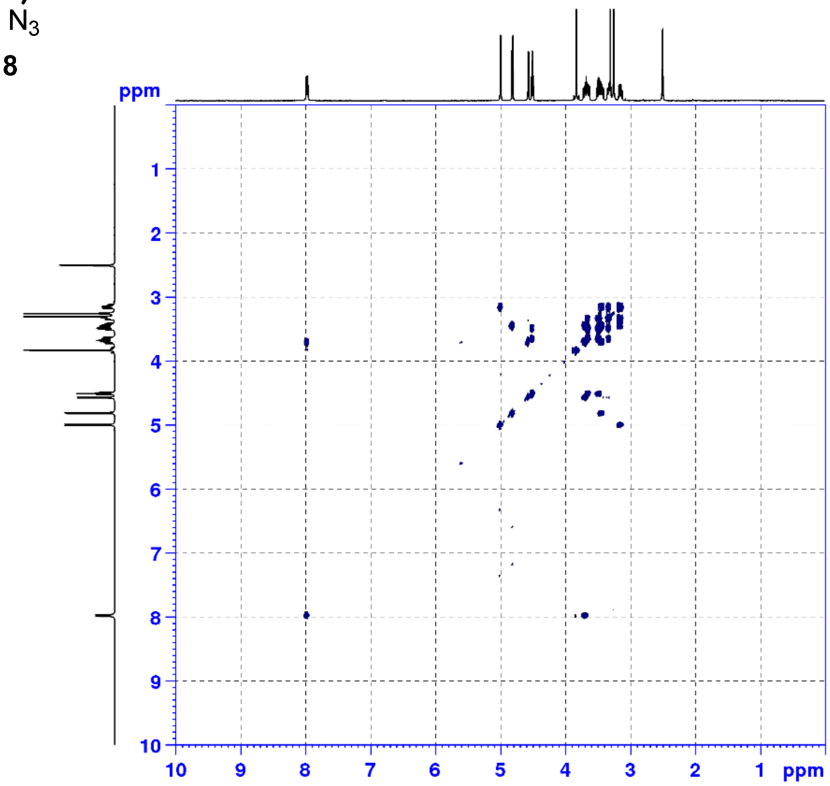

HSQC and COSY NMR spectra of compound **18** in DMSO-d_6

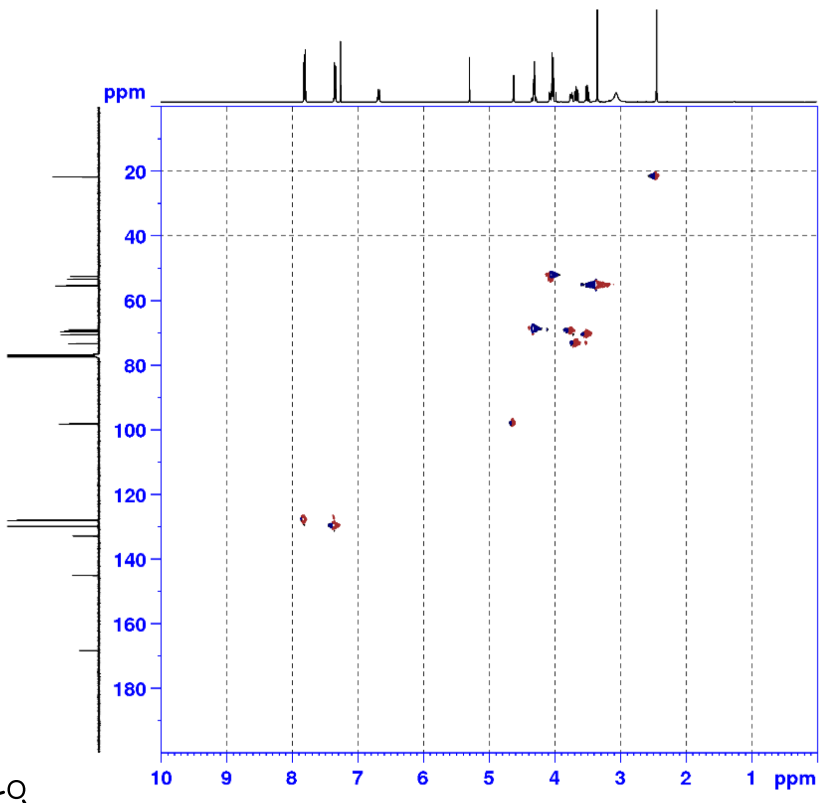

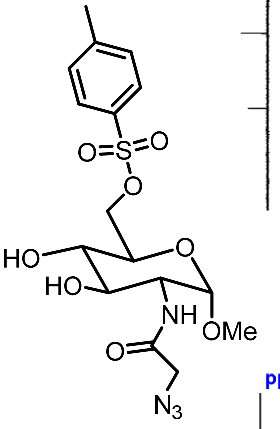

19

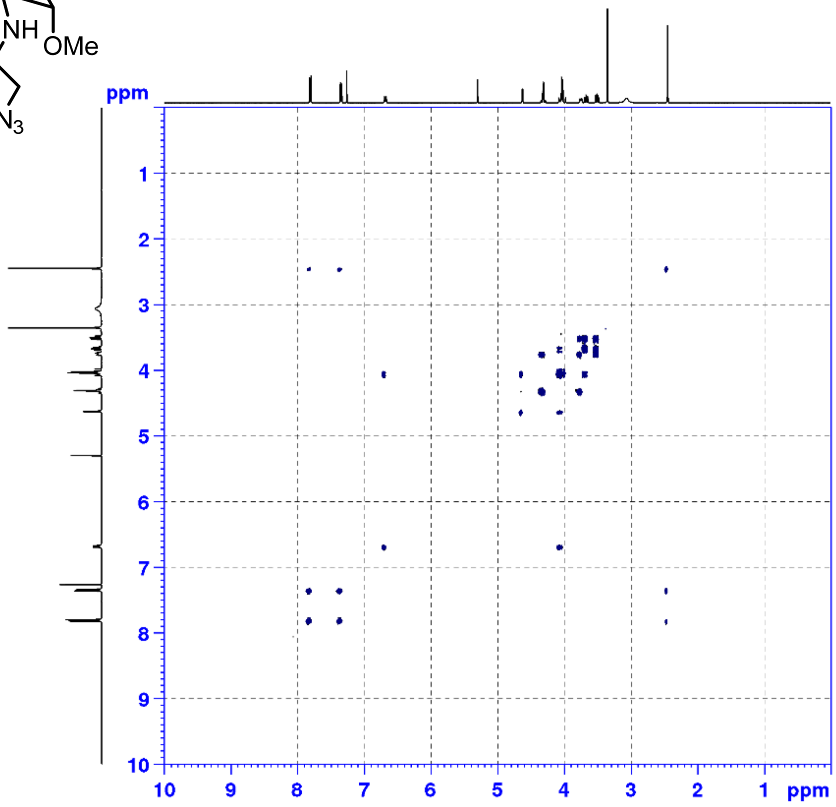

HSQC and COSY NMR spectra of compound **19** in $CDCl_3$

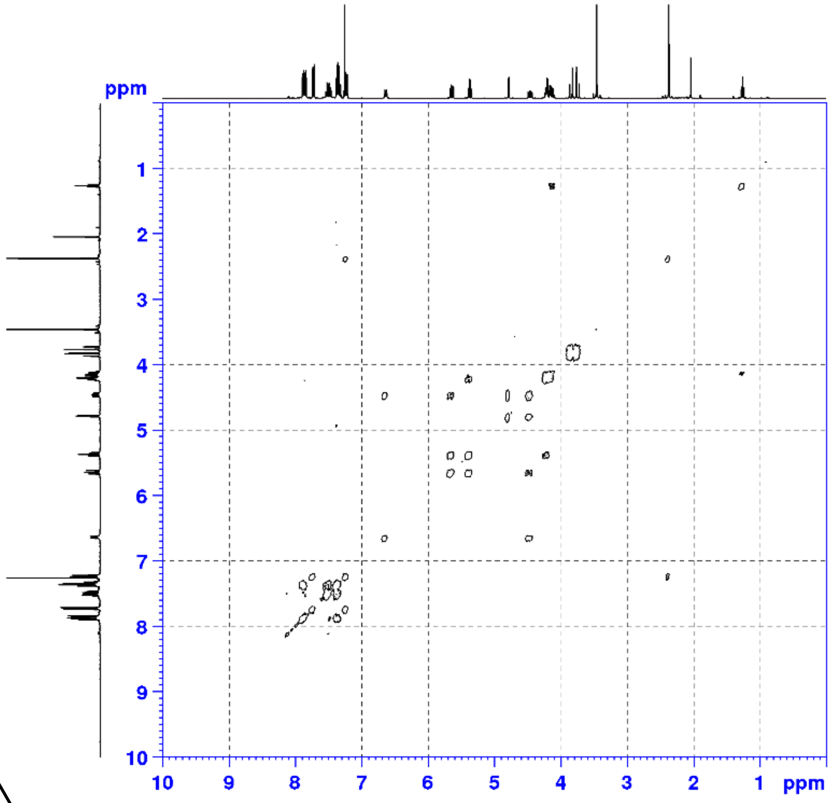

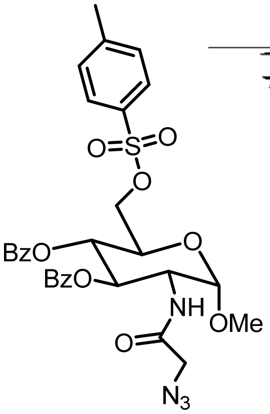

20

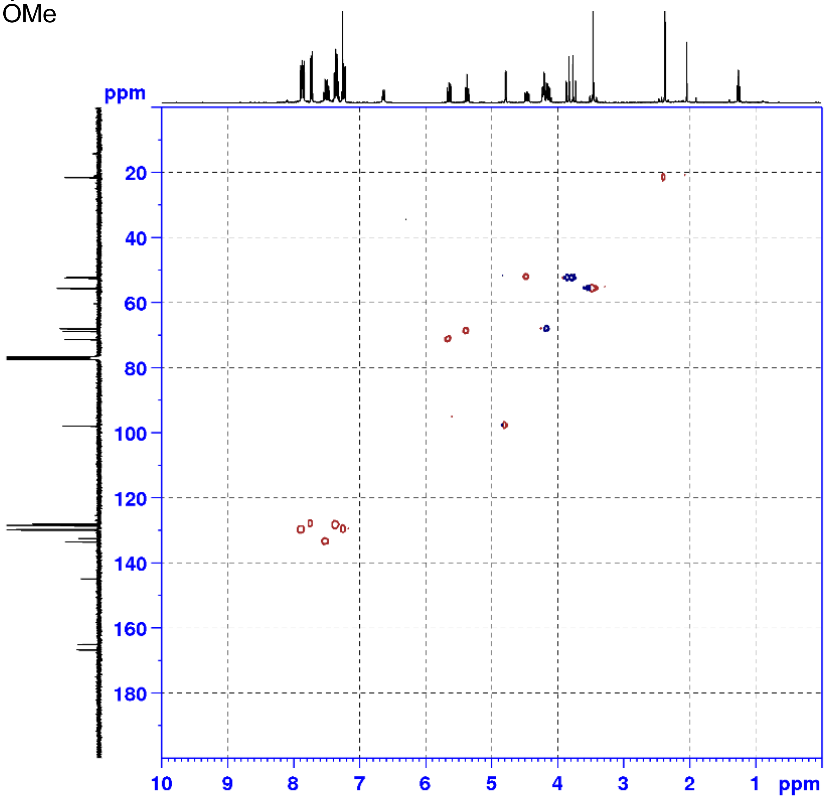

HSQC and COSY NMR spectra of compound **20** in $CDCl_3$

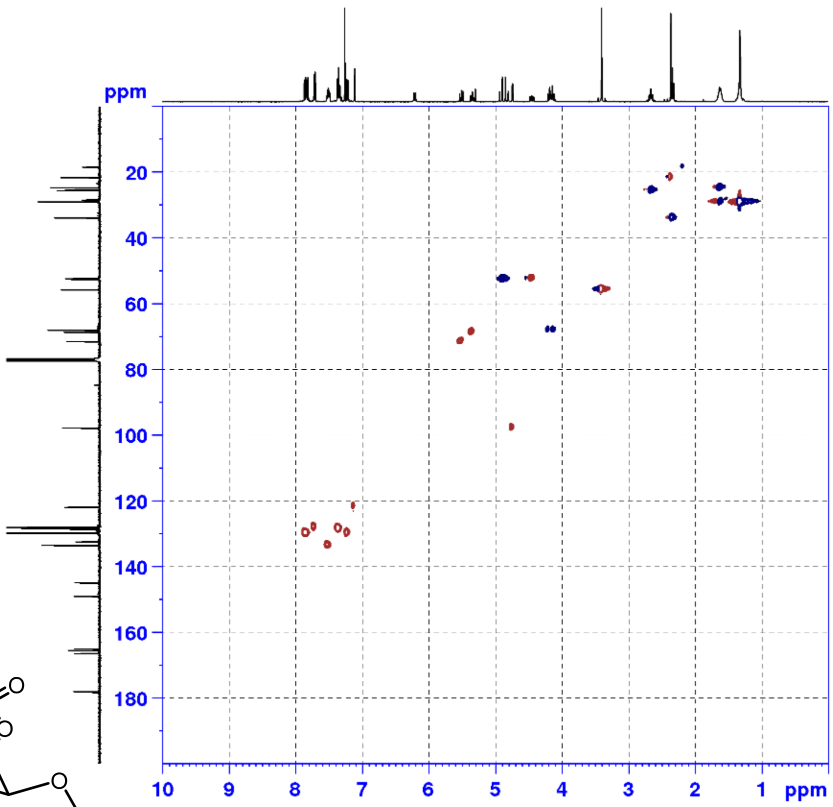

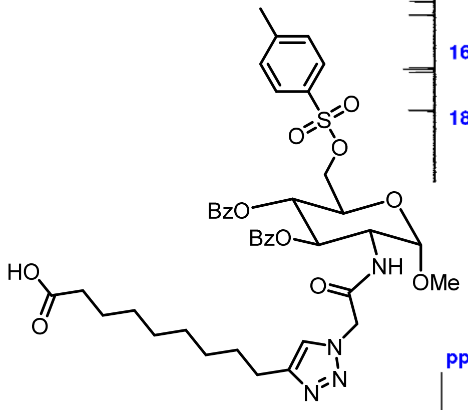

21

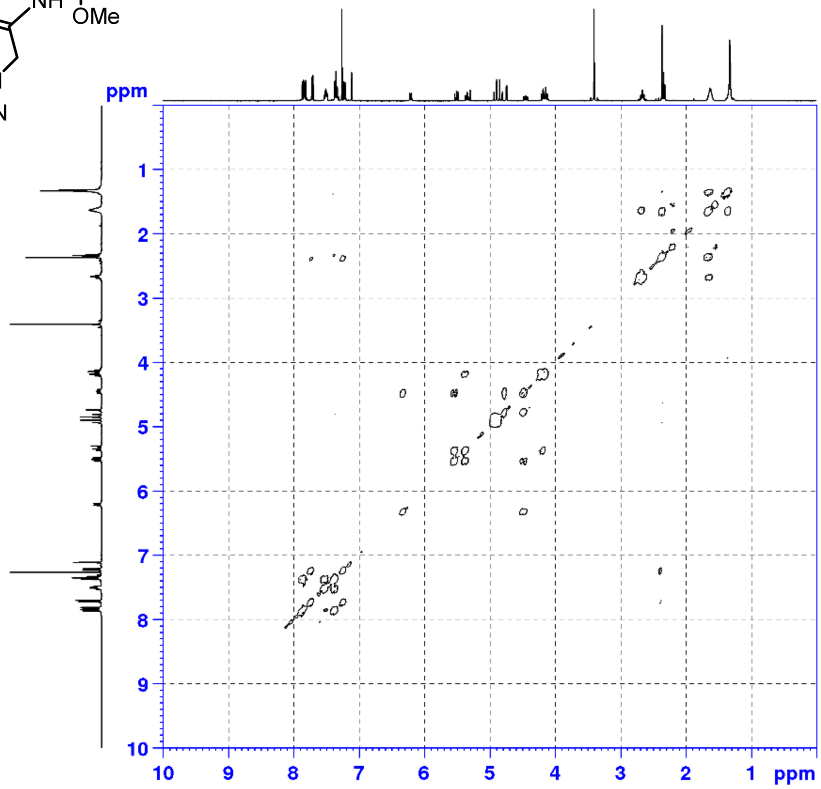

HSQC and COSY NMR spectra of compound **21** in $CDCl_3$

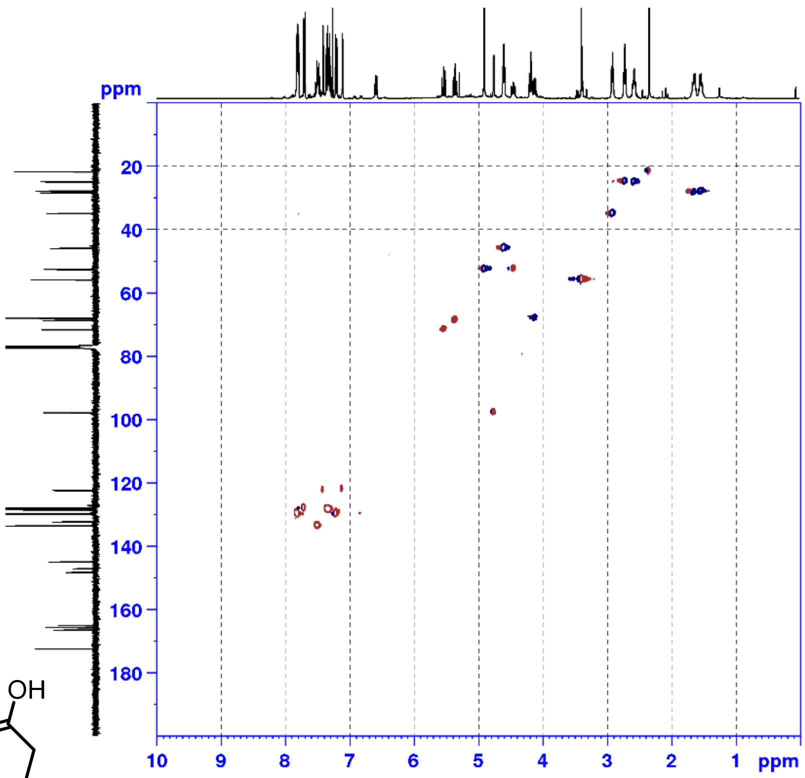

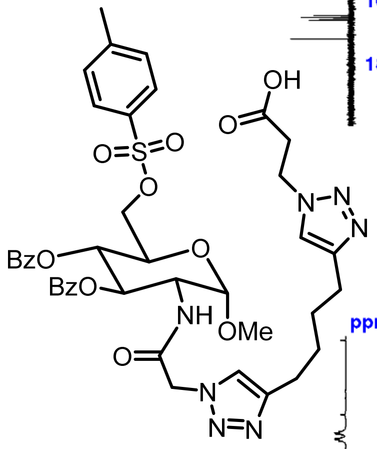

23

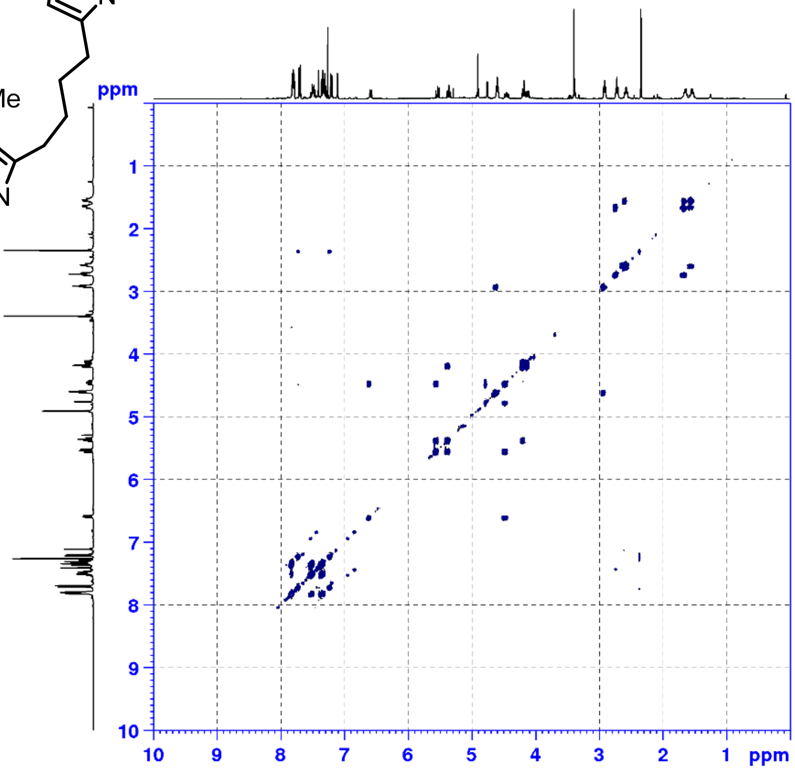

HSQC and COSY NMR spectra of compound **23** in $CDCl_3$

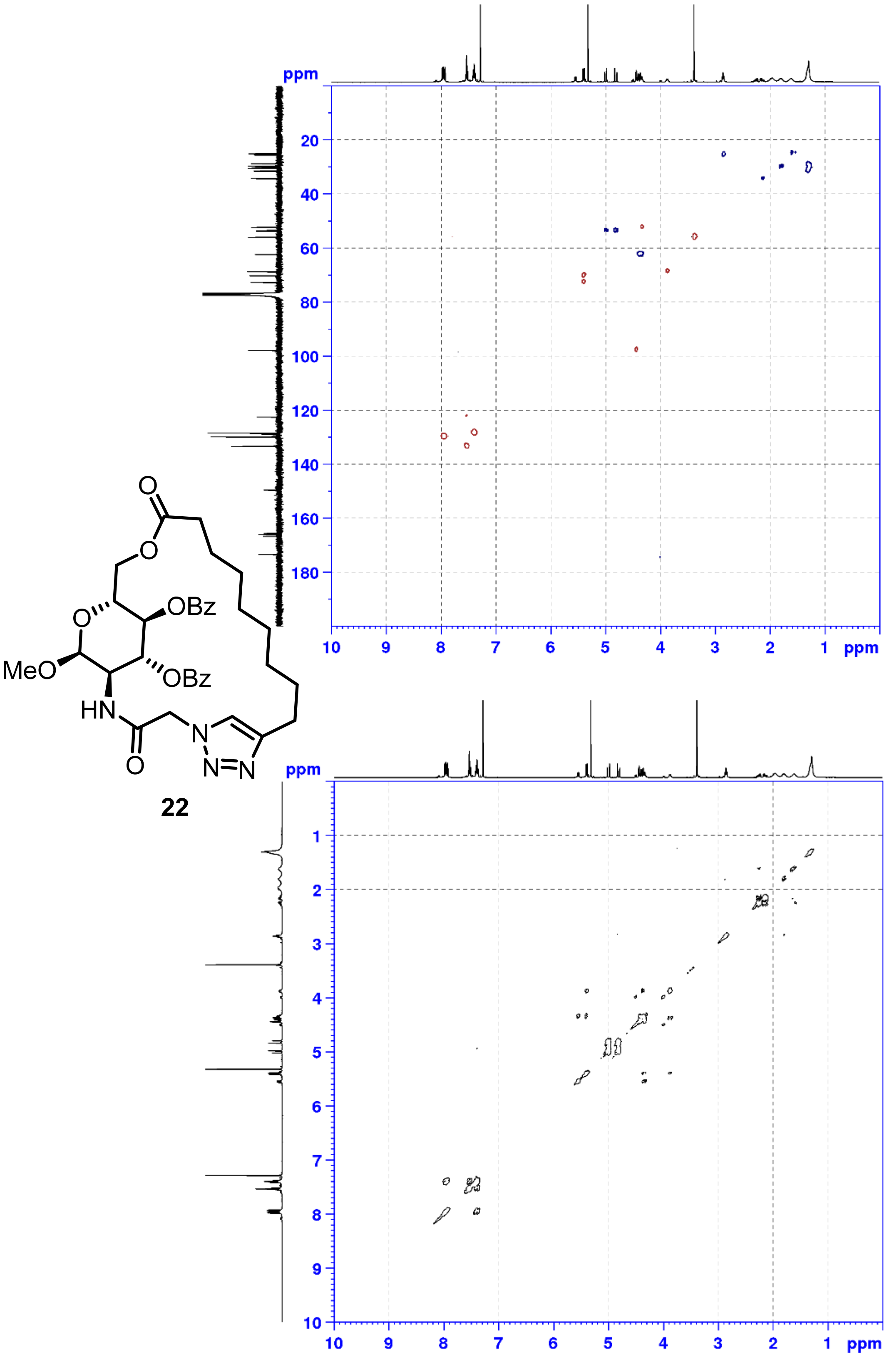

HSQC and COSY NMR spectra of compound **22** in $CDCl_3$

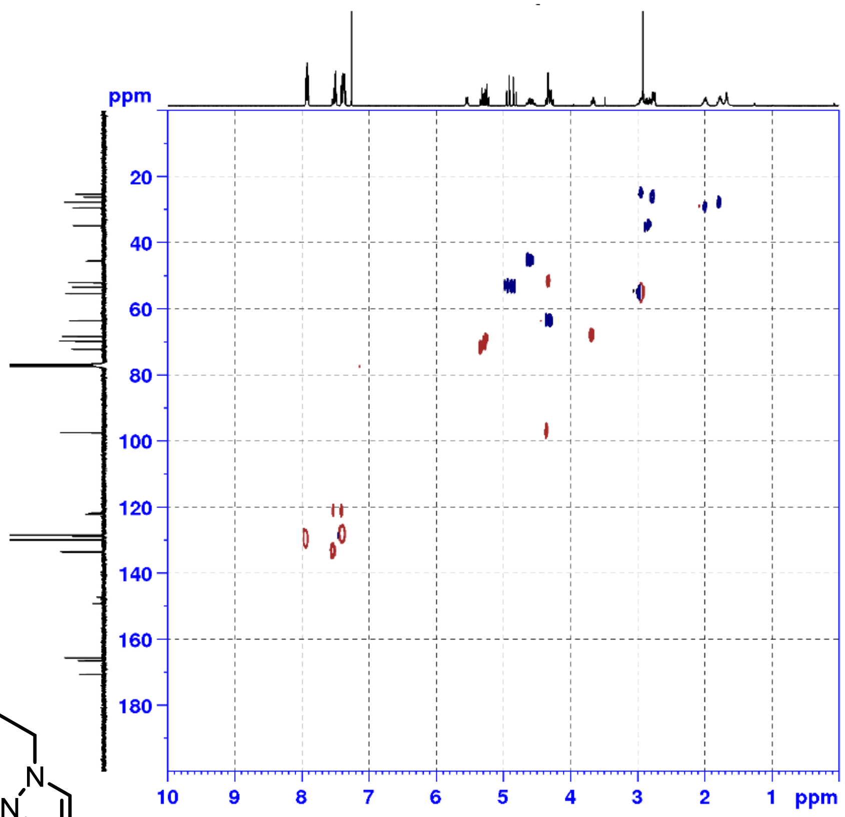

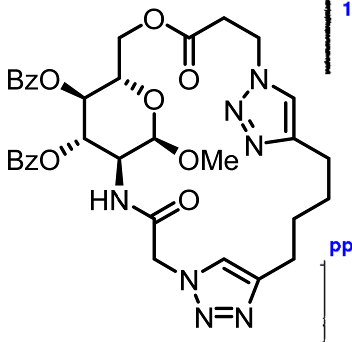

24

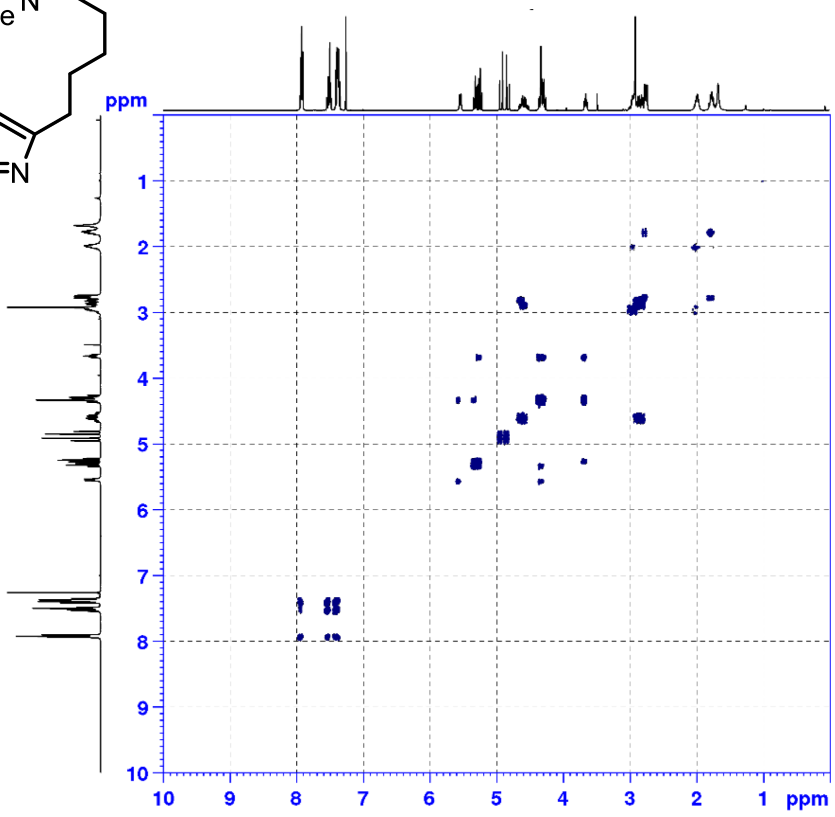

HSQC and COSY NMR spectra of compound **24** in $CDCl_3$

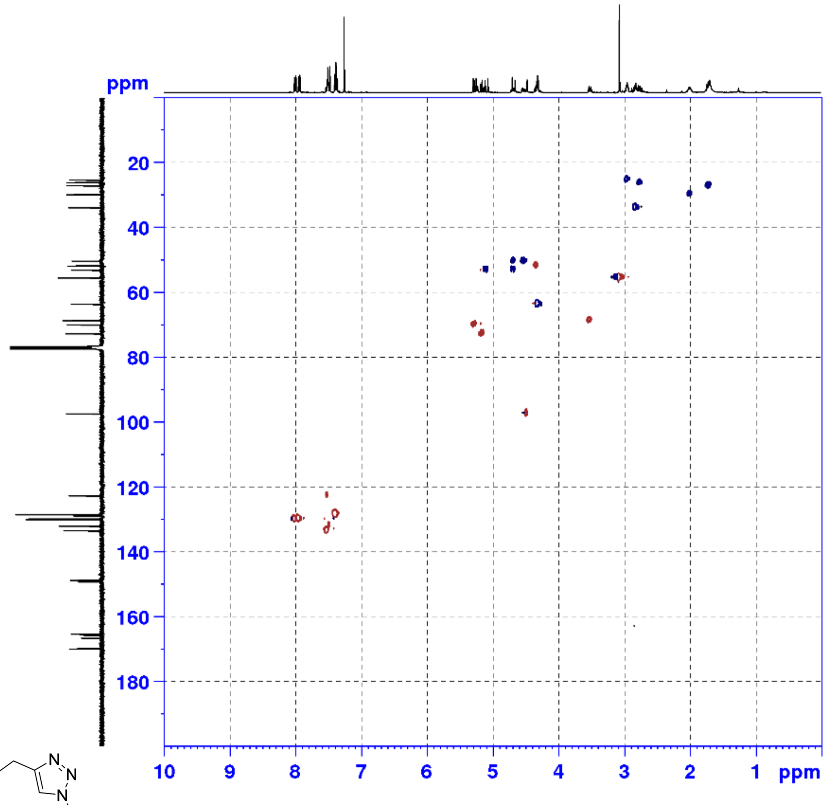

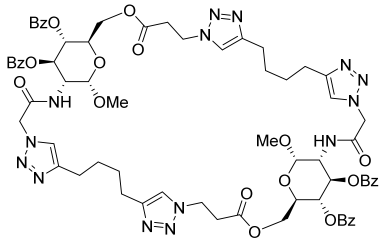

25

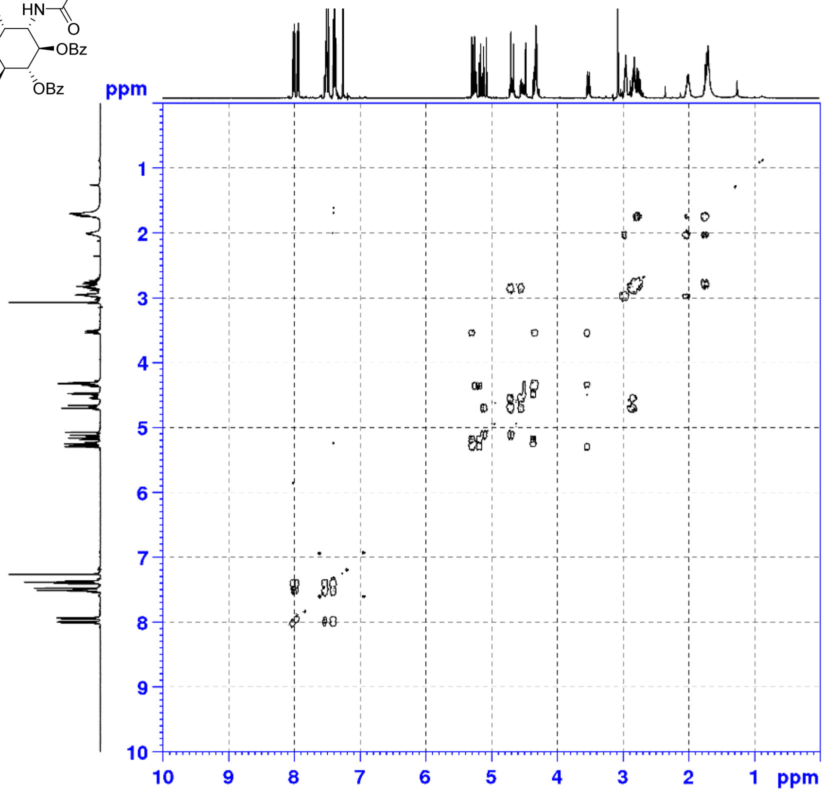

HSQC and COSY NMR spectra of compound **25** in $CDCl_3$

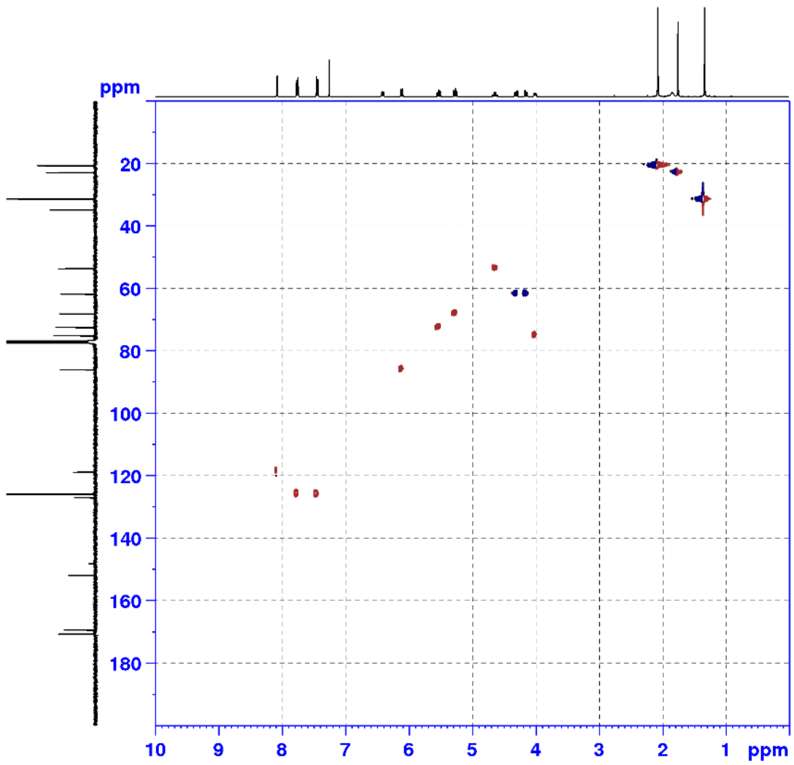

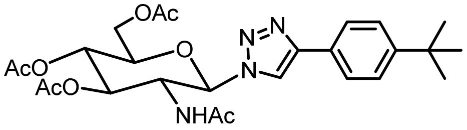

31

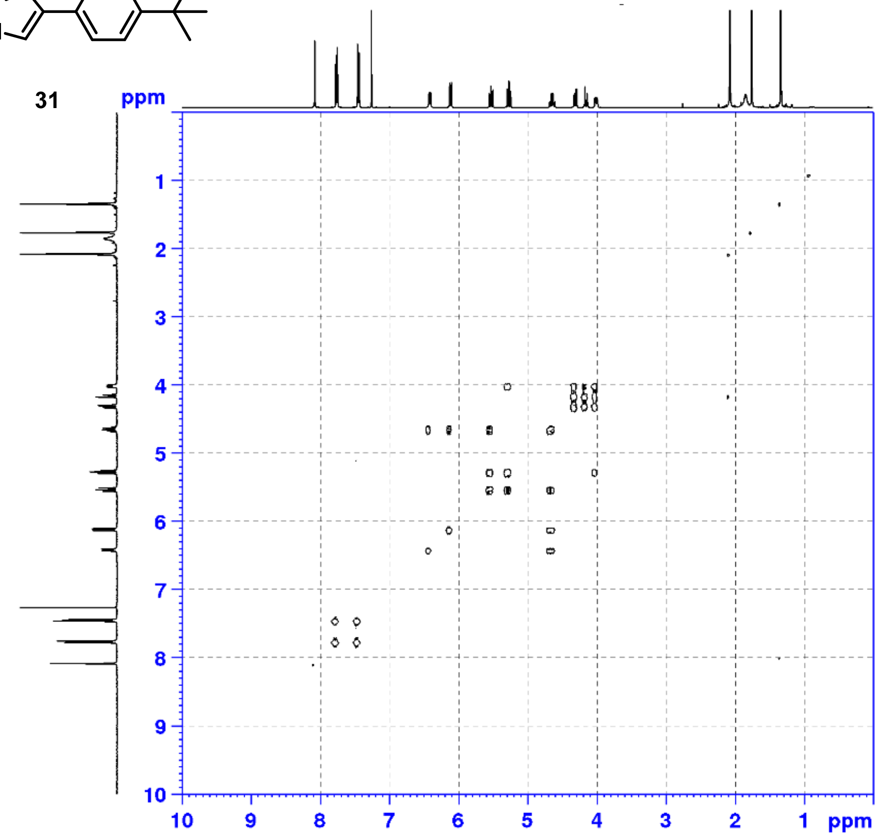

HSQC and COSY NMR spectra of compound **31** in $CDCl_3$

IV. Anion binding studies by ^{1}H NMR spectroscopy

1. **LM28** with tetrabutylammonium chloride (TBACl)

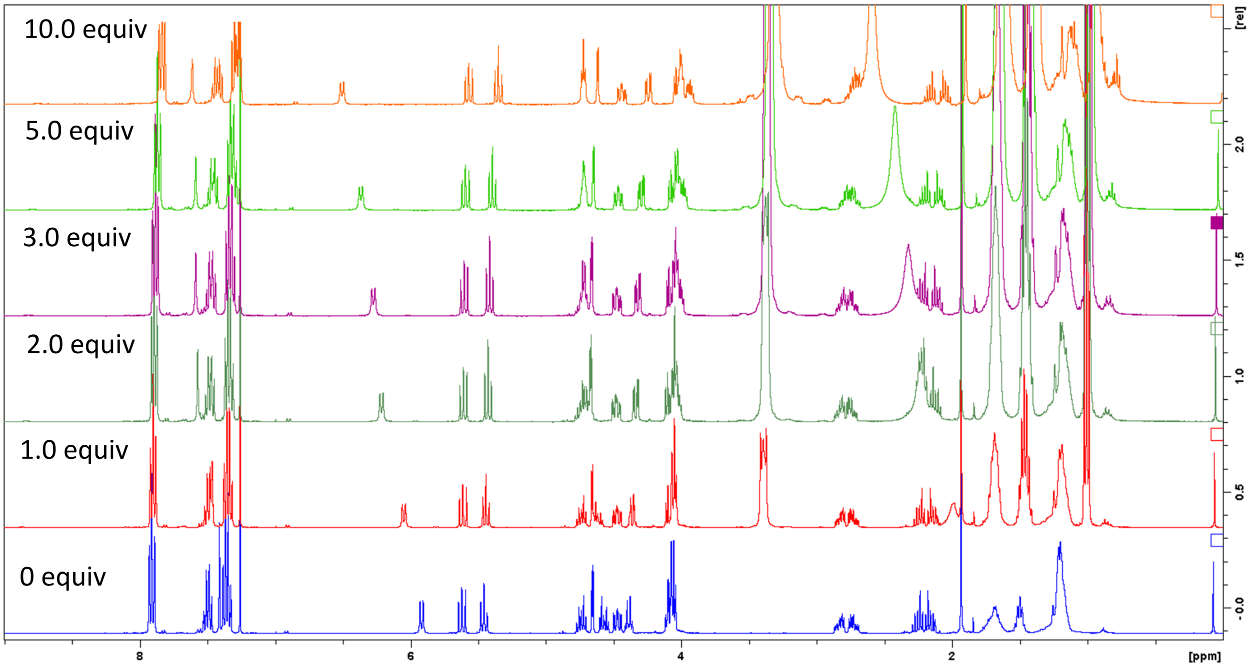

Figure S1. Stacked ^{1}H NMR spectra of **LM28** with different concentrations of TBACl (0-9 ppm)

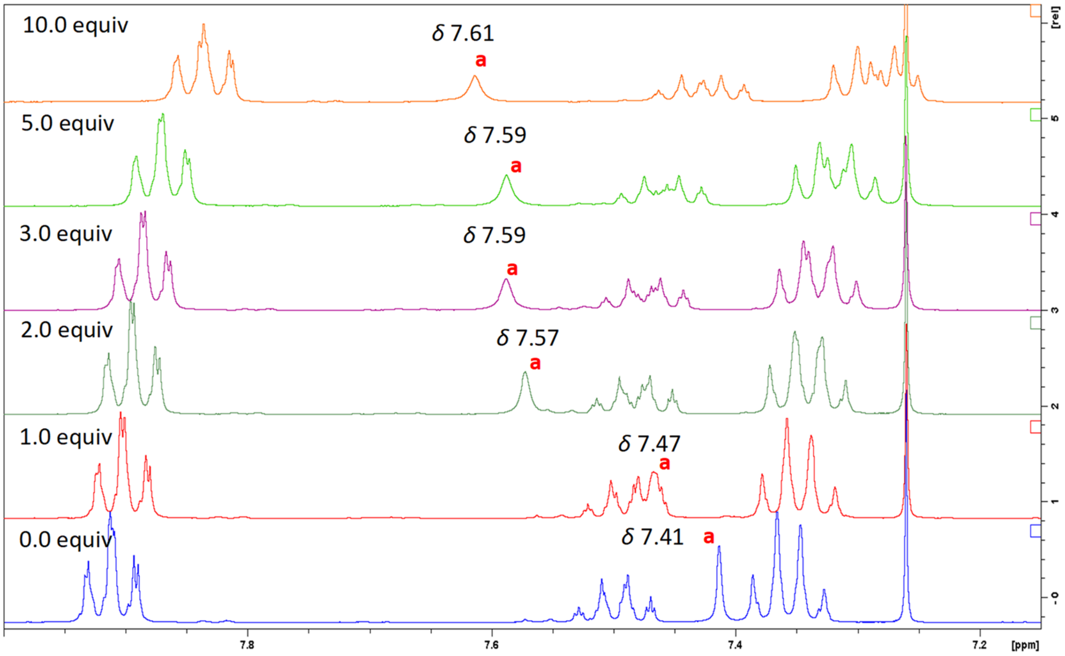

Figure S2. Stacked ^{1}H NMR spectra of **LM28** with different concentrations of TBACl.

2. **LM28** with tetrabutylammonium bromide (TBAB)

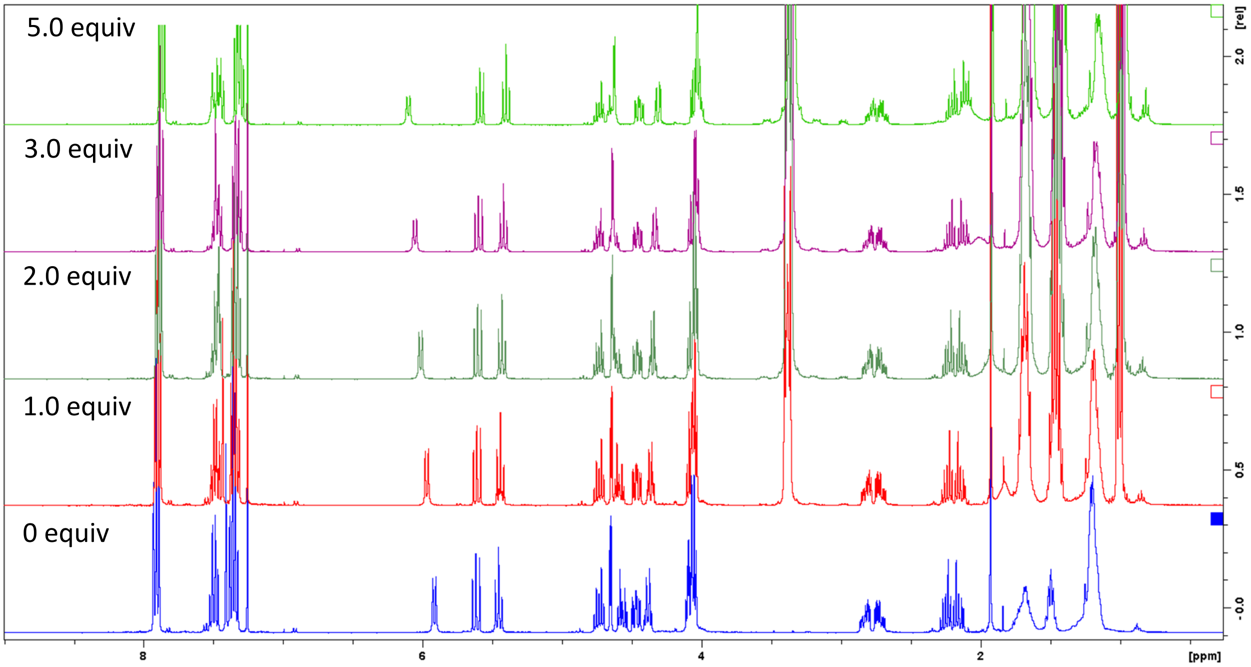

Figure S3. Stacked ^{1}H NMR spectra of **LM28** with different concentrations of TBAB (0-9 ppm).

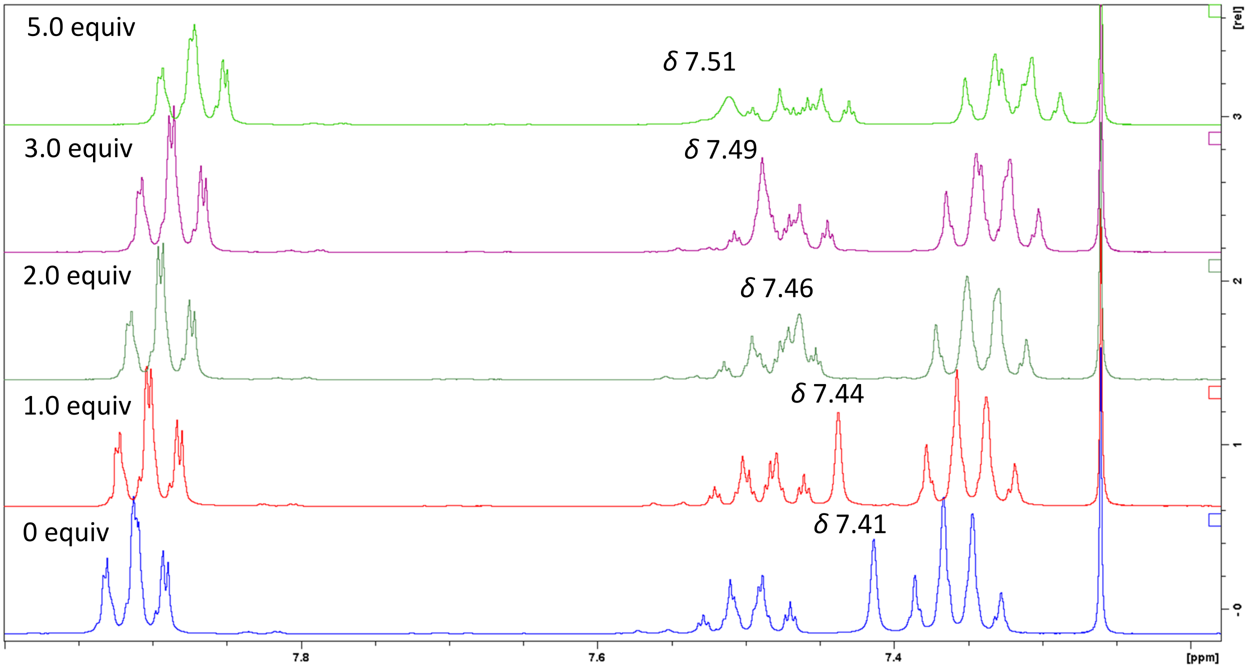

Figure S4. Stacked ^{1}H NMR spectra of **LM28** with different concentrations of TBAB, triazole proton labeled.

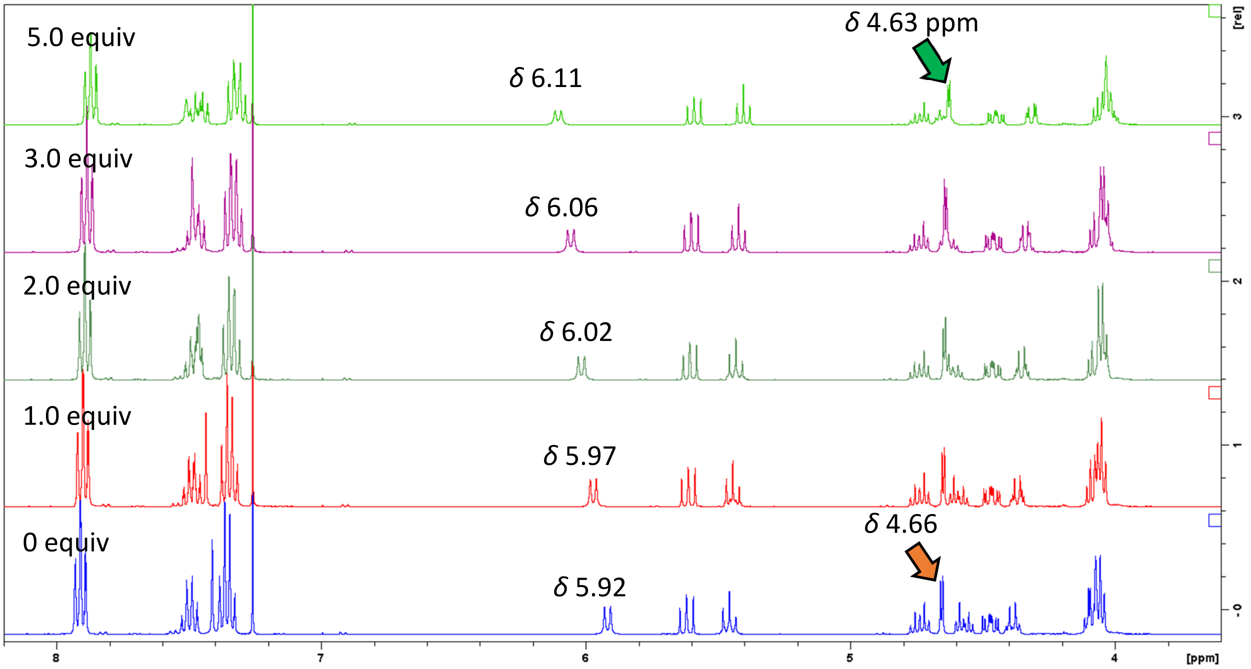

Figure S5. Stacked ^{1}H NMR spectra of **LM28** with different concentrations of TBAB.

3. **LM28** with tetrabutylammonium iodide (TBAI)

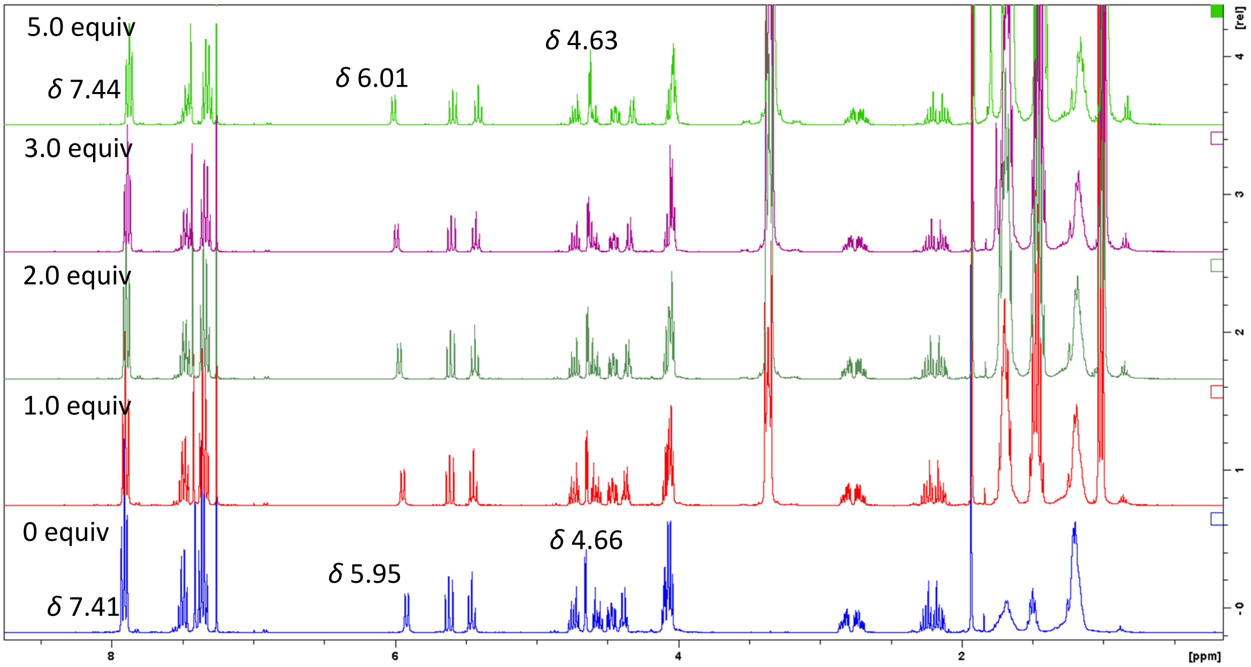

Figure S6. Stacked ^{1}H NMR spectra of **LM28** with different concentrations of TBAI (0-8.7 ppm).

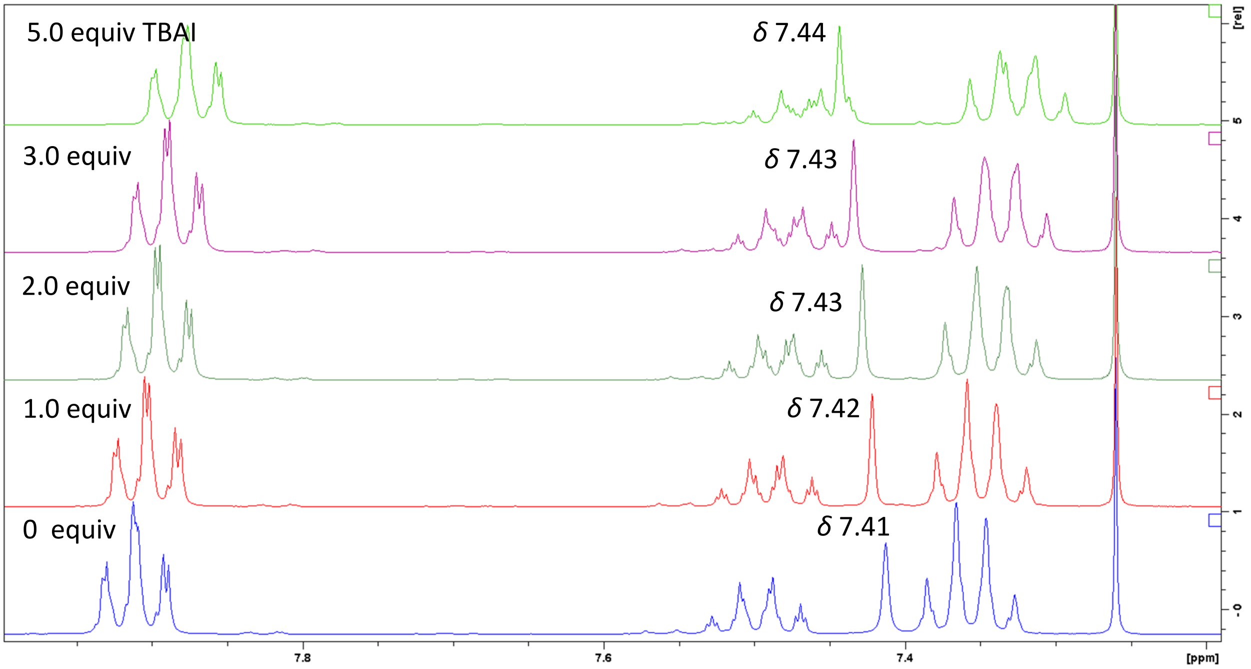

Figure S7. Stacked ^{1}H NMR spectra of **LM28** with different concentrations of TBAI.

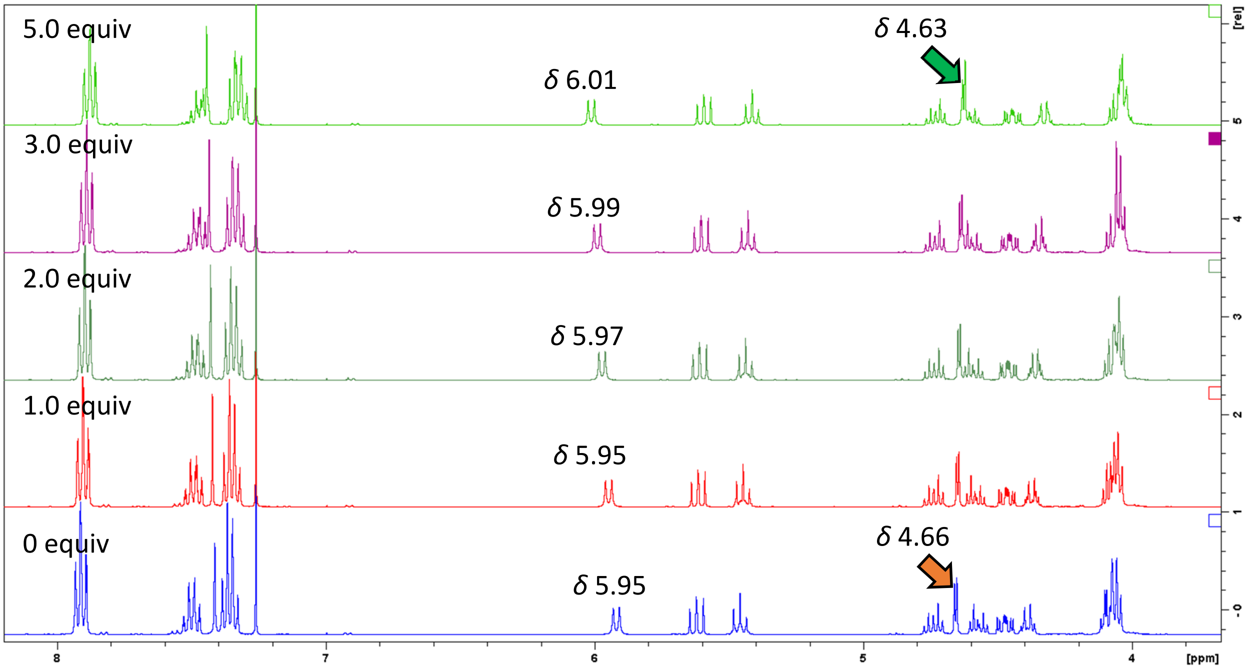

Figure S8. Stacked ^{1}H NMR spectra of **LM28** with different concentrations of TBAI.

4. **DM35** with TBACl

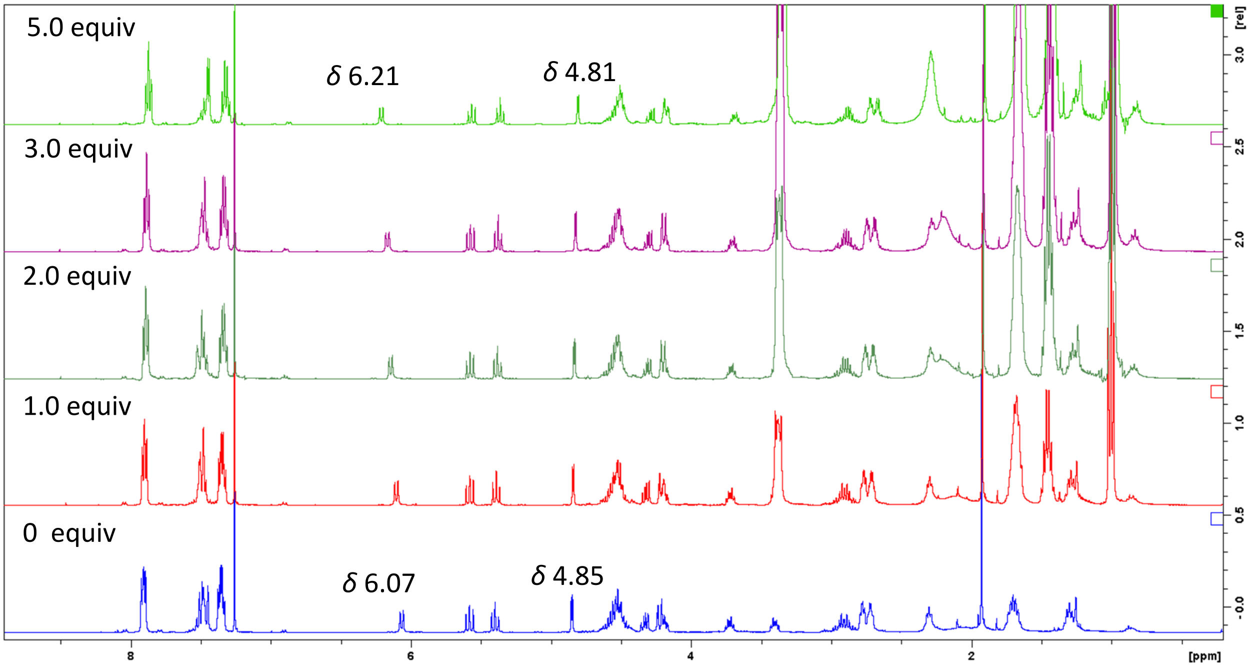

Figure S9. Stacked ^{1}H NMR spectra of **DM35** with different concentrations of TBACl.

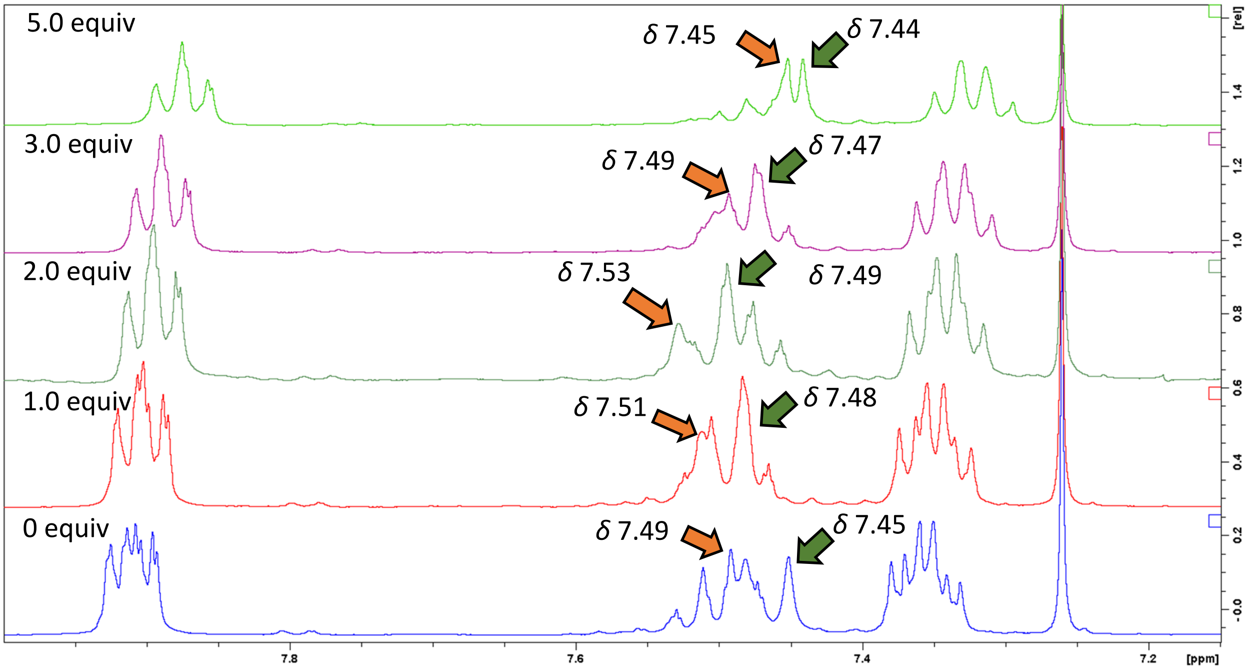

Figure S10. Stacked ^{1}H NMR spectra of **DM35** with different concentrations of TBACl, triazole protons labeled.

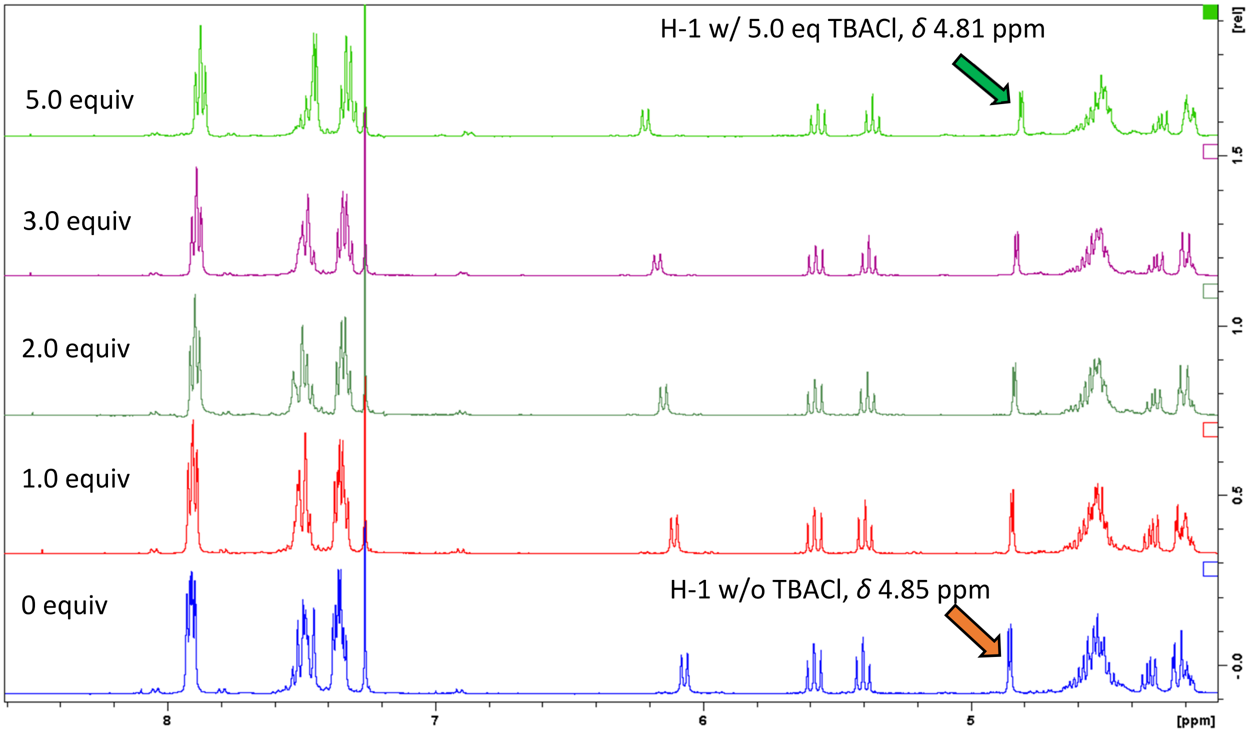

Figure S11. Stacked ^{1}H NMR spectra of **DM35** with different concentrations of TBACl.

5. **DM35** with TBAB

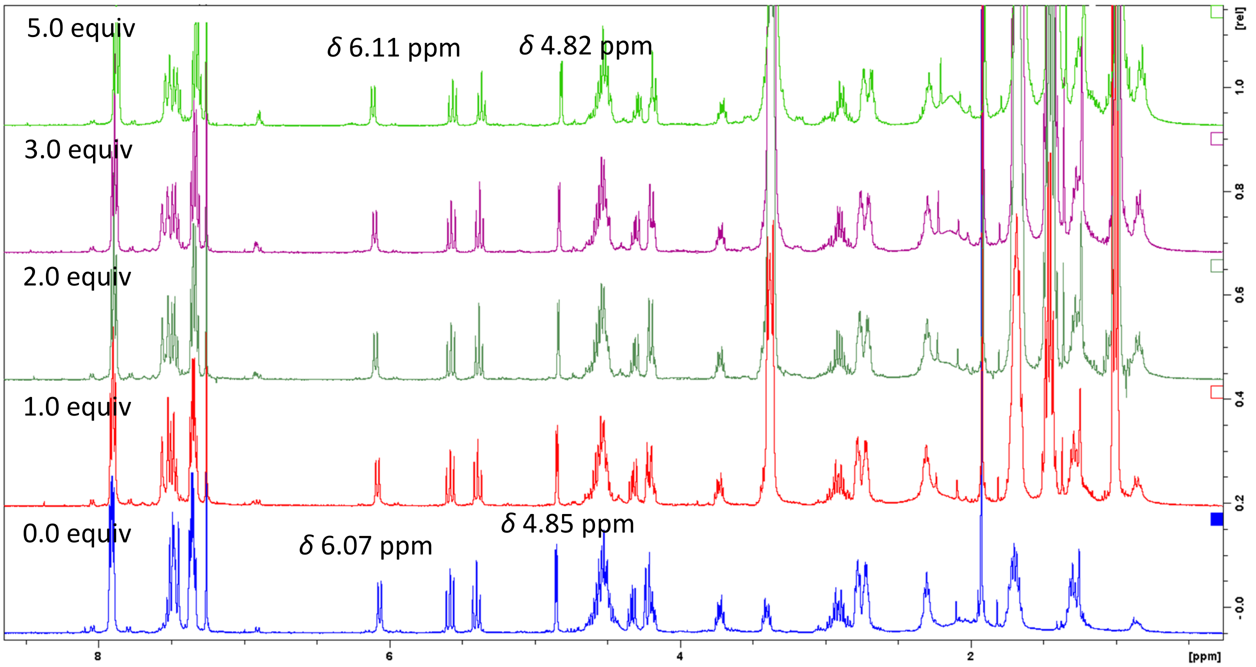

Figure S12. Stacked ^{1}H NMR spectra of **DM35** with different concentrations of TBAB.

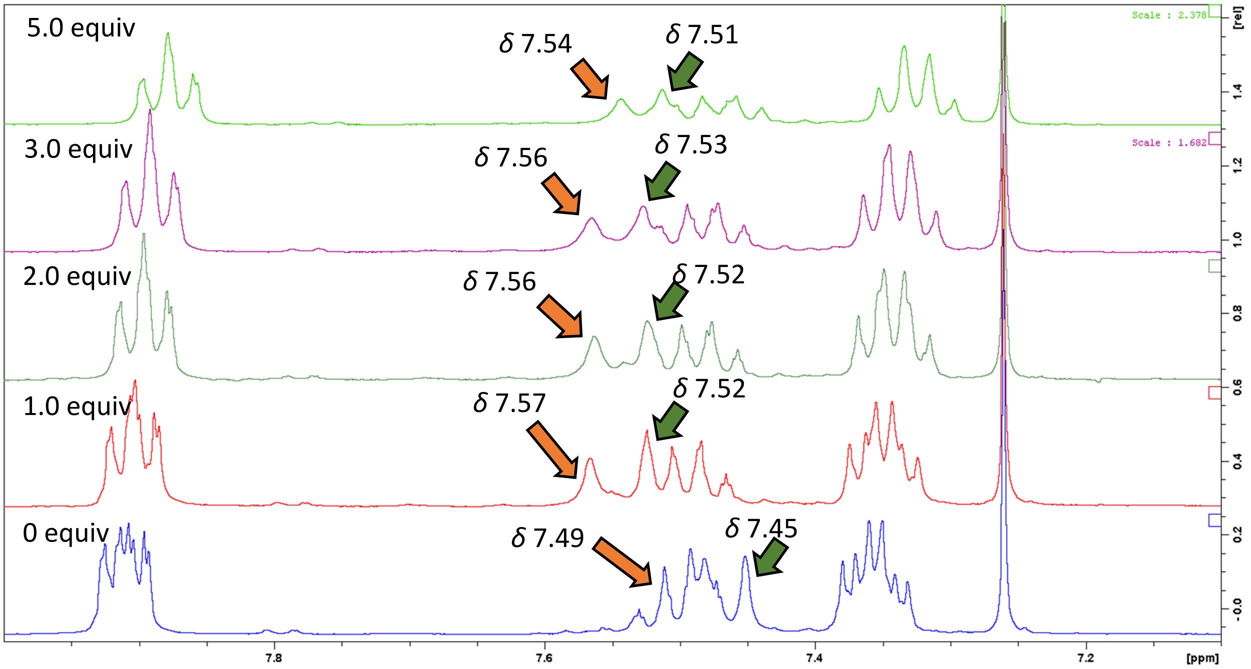

Figure S13. Stacked ^{1}H NMR spectra of **DM35** with different concentrations of TBAB, triazole protons marked.

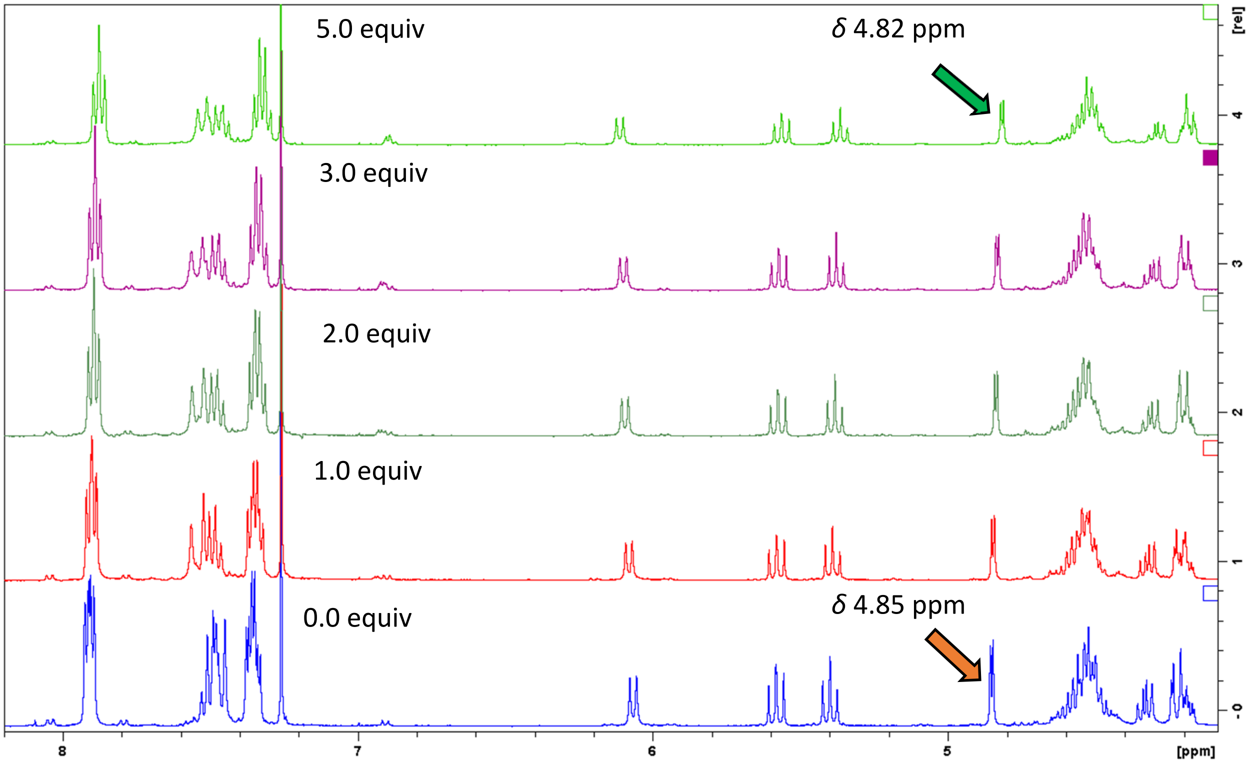

Figure S14. Stacked ^{1}H NMR spectra of **DM35** with different concentrations of TBAB.

6. DM35 with TBAI

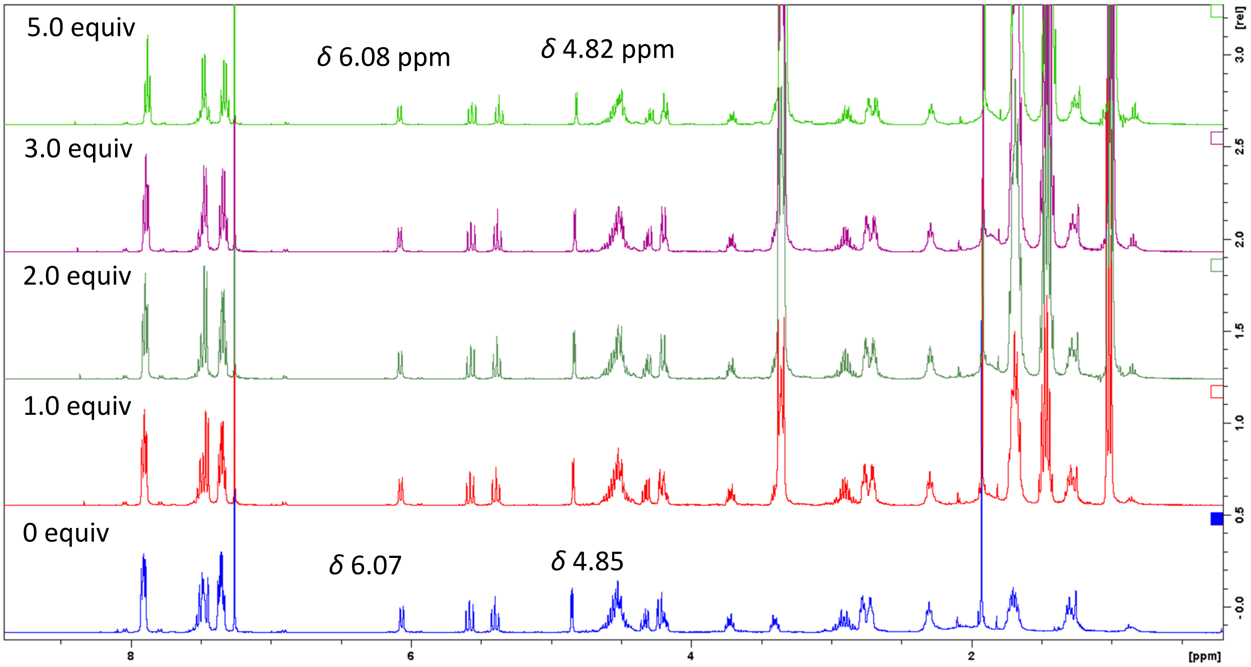

Figure S15. Stacked ^{1}H NMR spectra of **DM35** with different concentrations of TBAI.

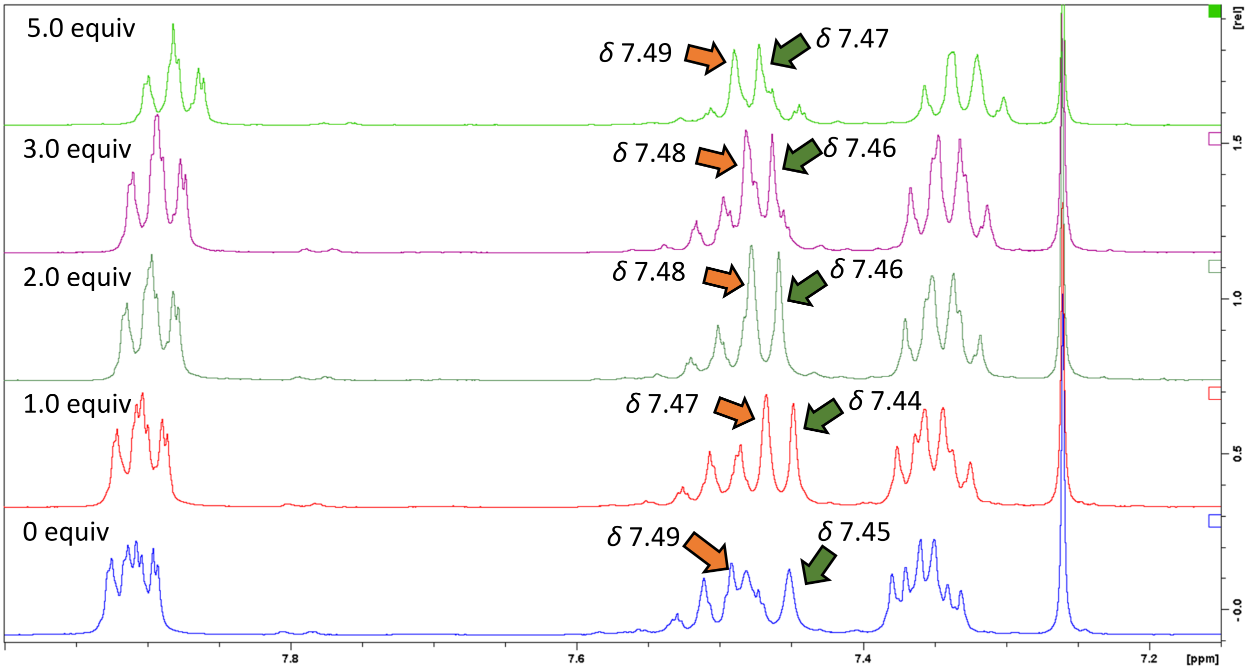

Figure S16. Stacked ^{1}H NMR spectra of **DM35** with different concentrations of TBAI, triazole protons labeled.

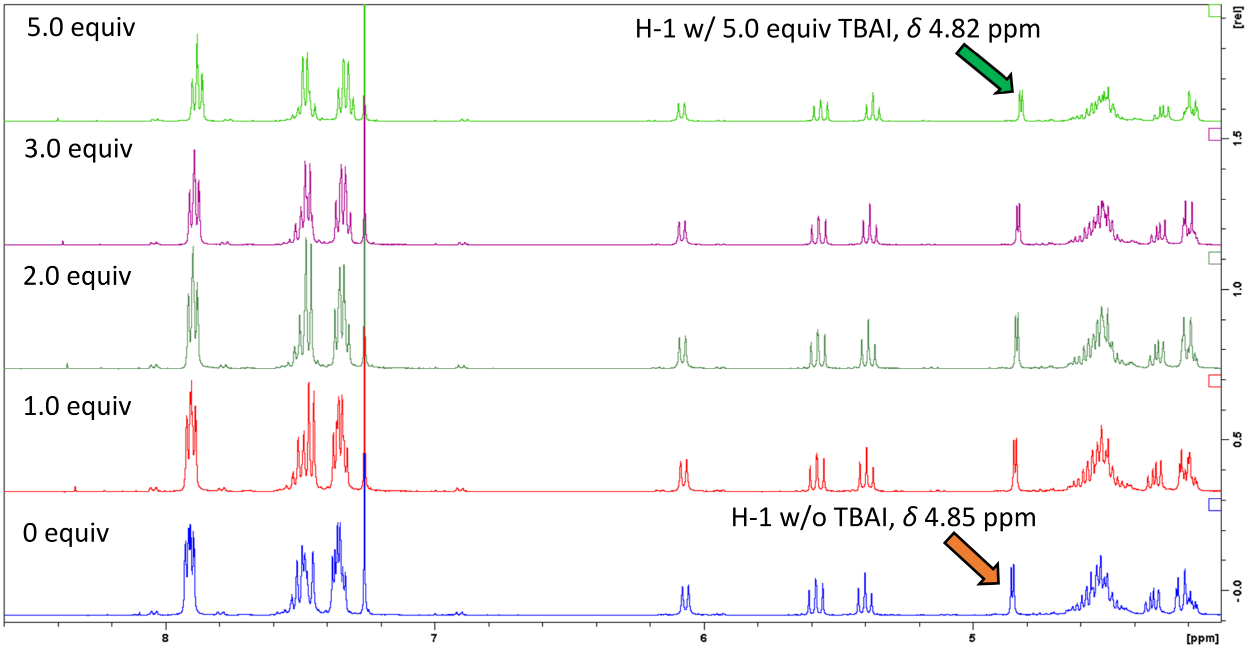

Figure S17. Overlay of ^{1}H NMR spectra of **DM35** with different concentrations of TBAI.

7. **C2ML** (compound **22**) with TBACl

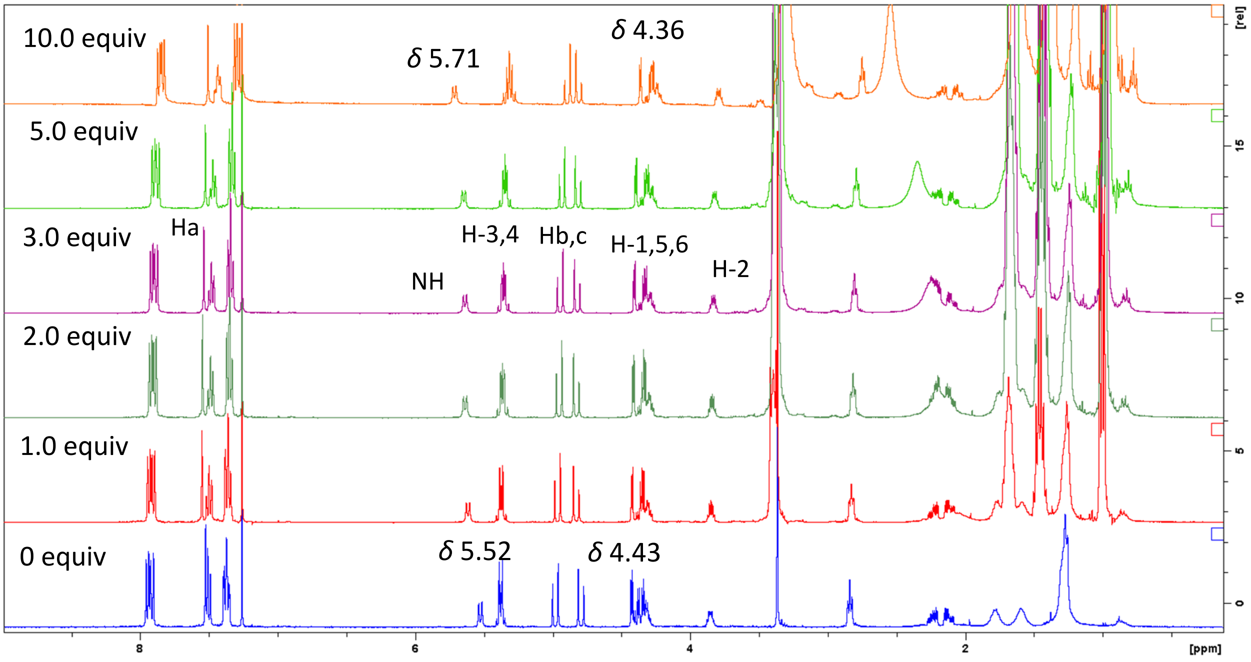

Figure S18. Stacked ^{1}H NMR spectra of compound **22** with TBACl (0-8.5 ppm).

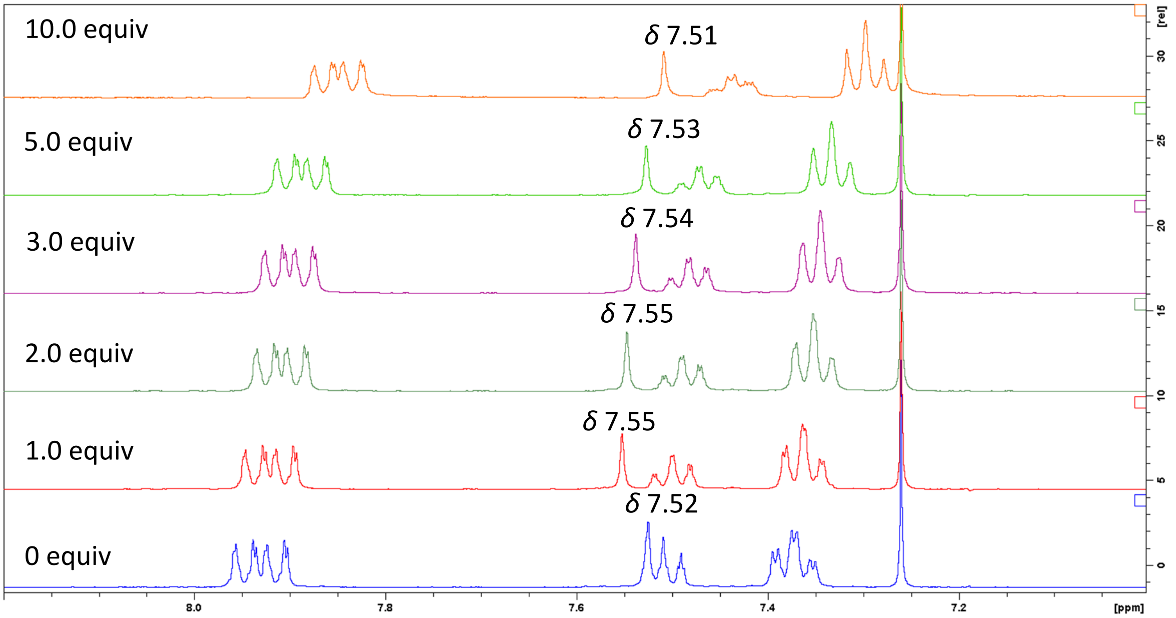

Figure S19. ^{1}H NMR spectra of compound **22** with TBACl, triazole signal marked.

8. C2DML (compound **24**) with TBACl

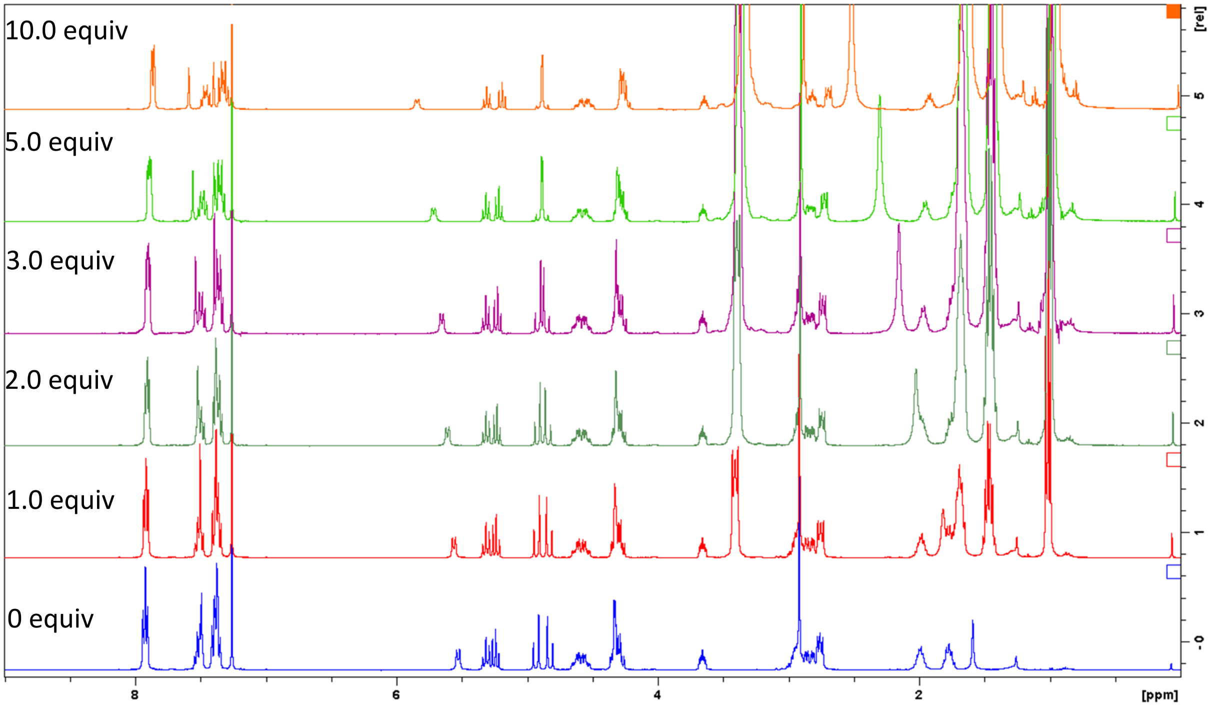

Figure S20. Stacked ^{1}H NMR spectra of compound **24** at different concentrations of TBACl.

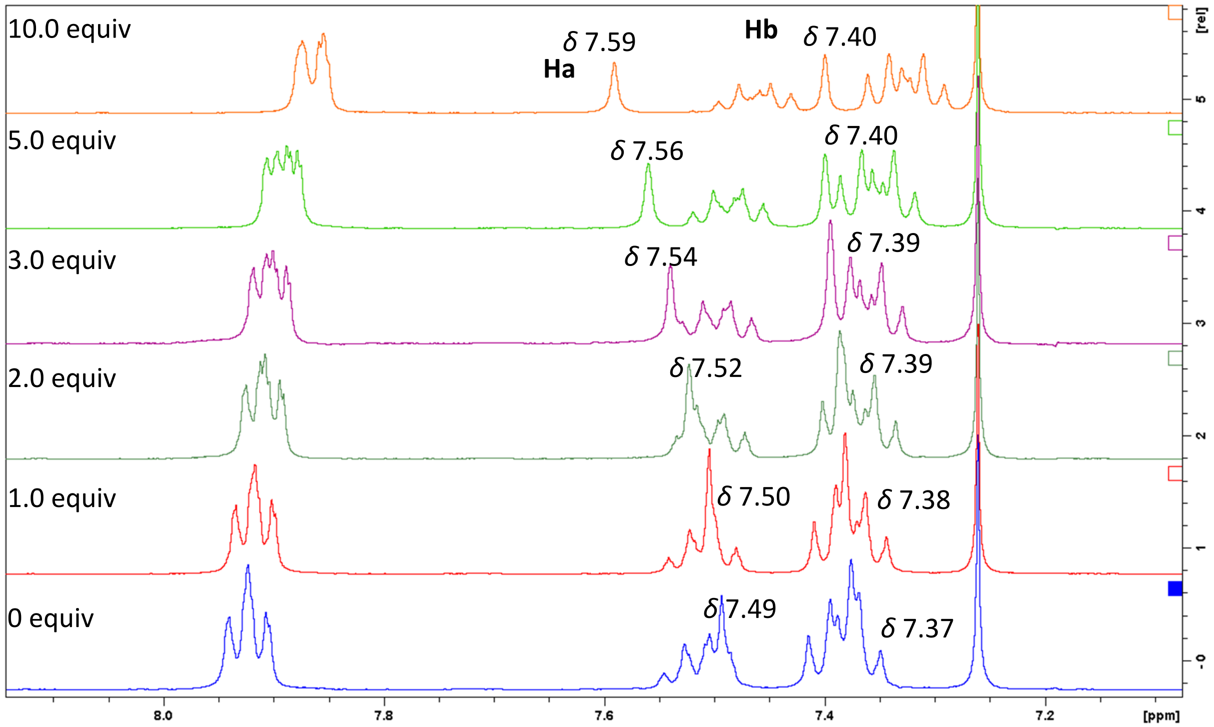

Figure S21. Stacked ^{1}H NMR spectra of compound **24** at different concentrations of TBACl,

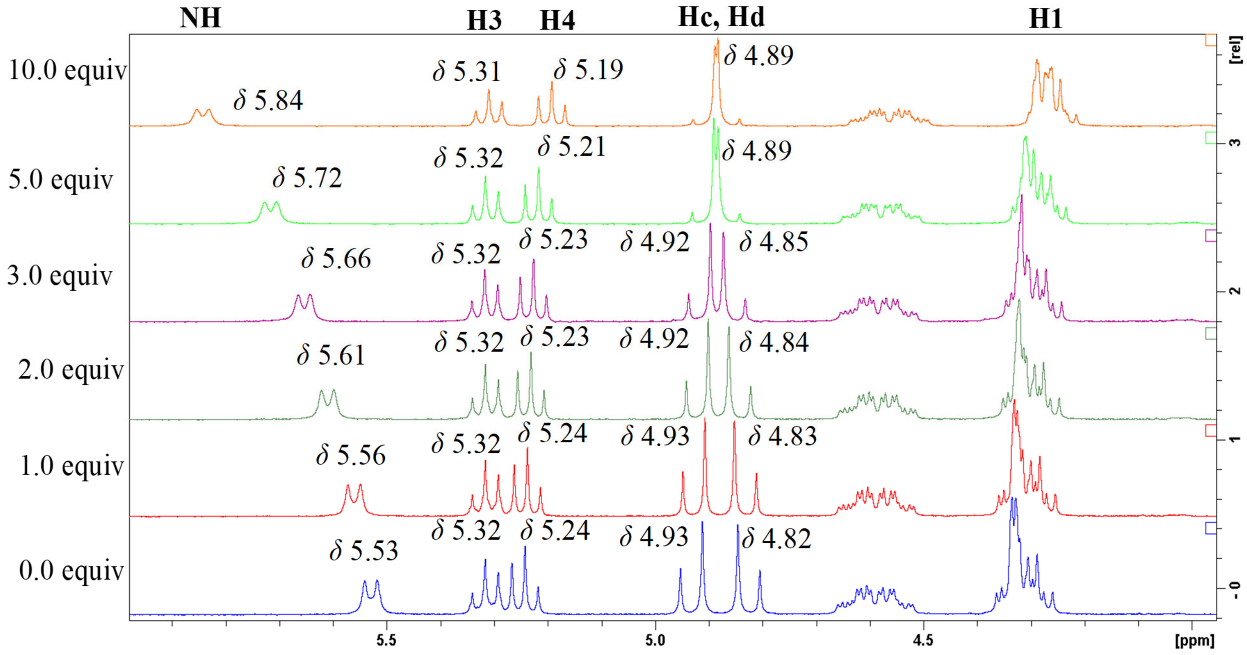

Figure S22. Stacked ^{1}H NMR spectra of compound **24** at different concentrations of TBACl.

V. Copper Binding Studies

DM35 with $CuSO_4{\cdot}5H_2O$

To a 25 mL RBF, **DM35** (27.0 mg, 0.036 mmol, 1.0 equiv) was dissolved in 2.0 mL of EtOH:H_2O (v/v 1:1) and $CuSO_4{\cdot}5H_2O$ (9.23 mg, 0.036, 1.0 equiv) was added. The reaction mixture was refluxed for 3 hours to observe greenish solution. The ^{1}H NMR at 3.0 hour showed disappearance of triazole peaks. The reaction was stopped, and the solvent was removed under pressure to obtain light green solid.

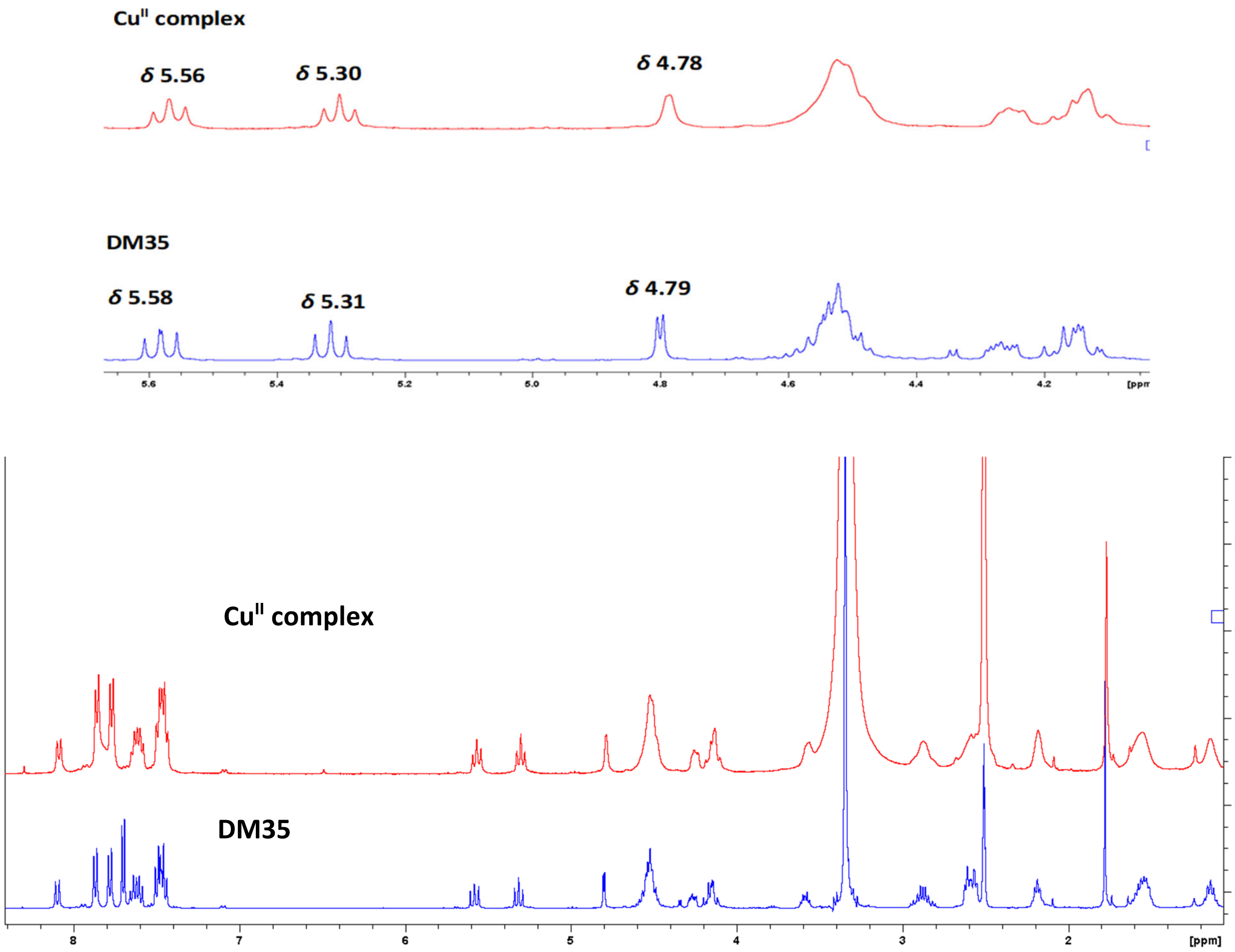

Figure S23. ^{1}H NMR spectra of **DM35** and its complex with $CuSO_4{\cdot}5H_2O$ in d_6-DMSO, the expanded regions are on top of the figure.

Temperature Study of DM35 Cu^{II} complex

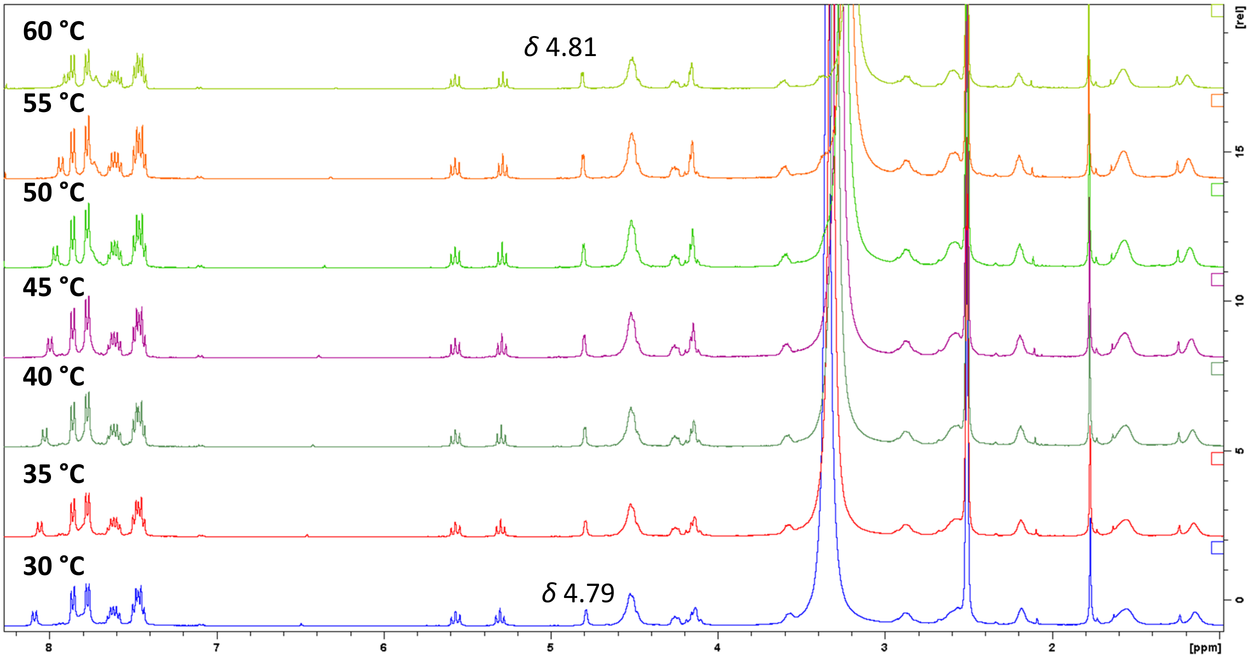

Figure S24. Stacked ^{1}H NMR spectra of **DM35** Cu^{II} complex with $CuSO_4 \cdot 5H_2O$, full range.

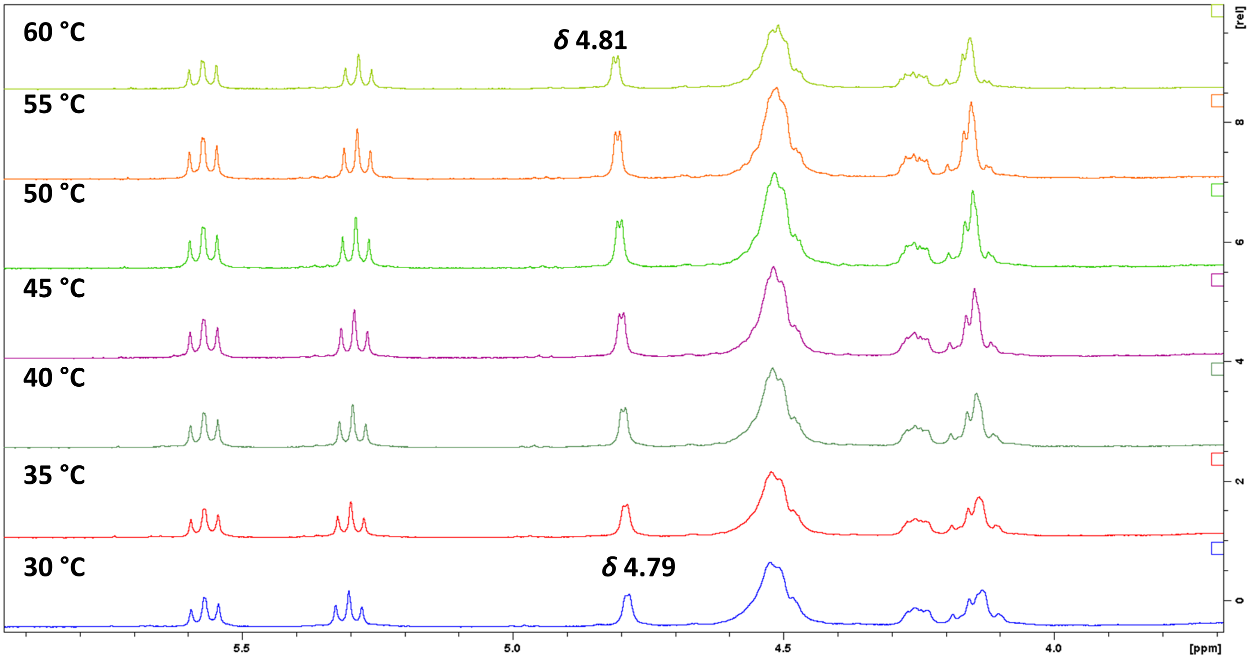

Fig S24a. ^{1}H NMR spectra of **DM35** Cu^{II} complex with $CuSO_4 \cdot 5H_2O$, anomeric proton labeled.

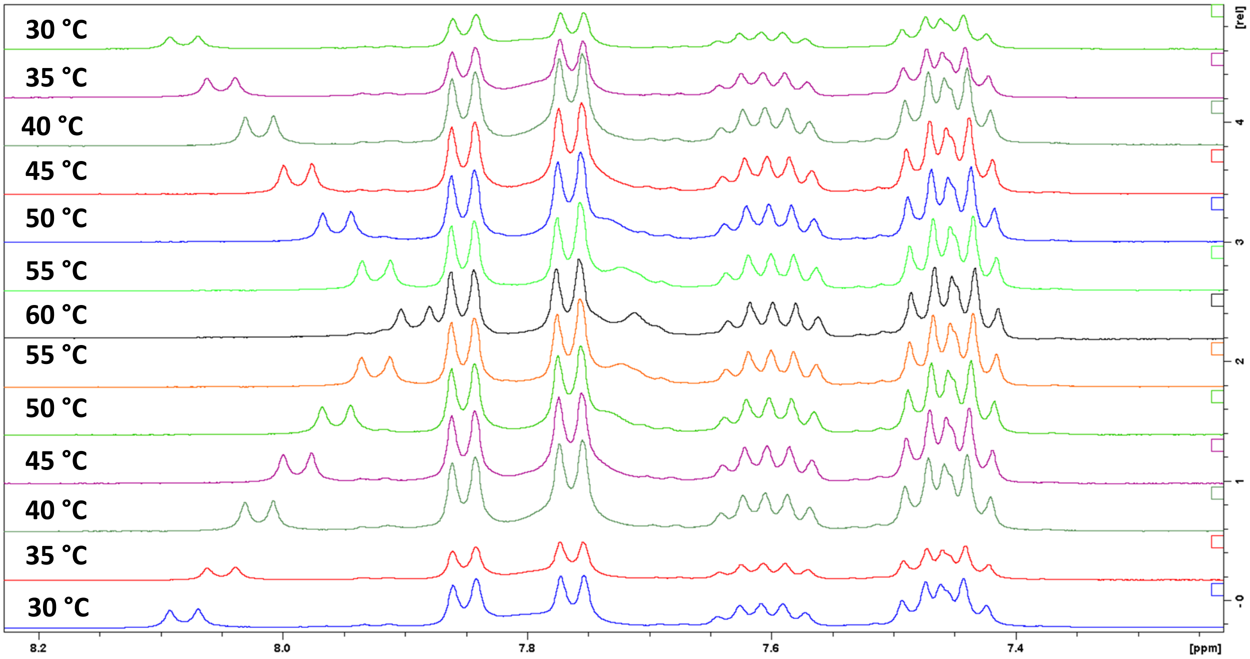

Figure S25. Stacked ^{1}H NMR spectra of **DM35** Cu^{II} complex with $CuSO_4{\cdot}5H_2O$, increasing and decreasing the temperature.

DM35 with $Cu(OAc)_2{\cdot}H_2O$

To a 25 mL RBF, **DM35** (12.0 mg, 0.016 mmol, 1.0 equiv) was dissolved in 1.0 mL of EtOH and $Cu(OAc)_2{\cdot}H_2O$ (3.28 mg, 0.016, 1.0 equiv) was added. The reaction mixture was refluxed for 2 hours to observe greenish blue solution. The ^{1}H NMR at 2.0 hour showed the only the broad peaks except for both triazole peaks moved downfield with broadening. The reaction was stopped, and the solvent was removed under pressure to obtain greenish solid.

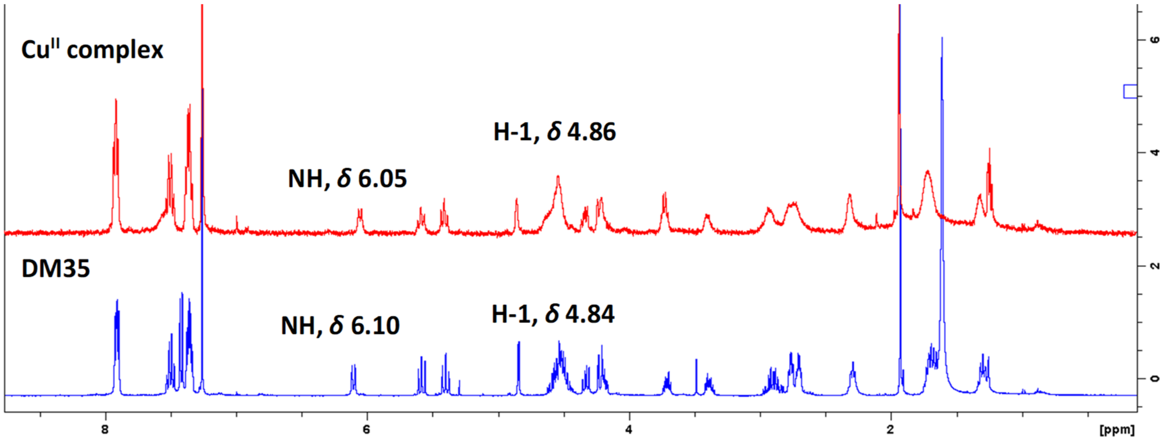

Figure S26. Stacked ^{1}H NMR spectra of **DM35** and its Cu^{II} complex with $Cu(OAc)_2{\cdot}H_2O$.

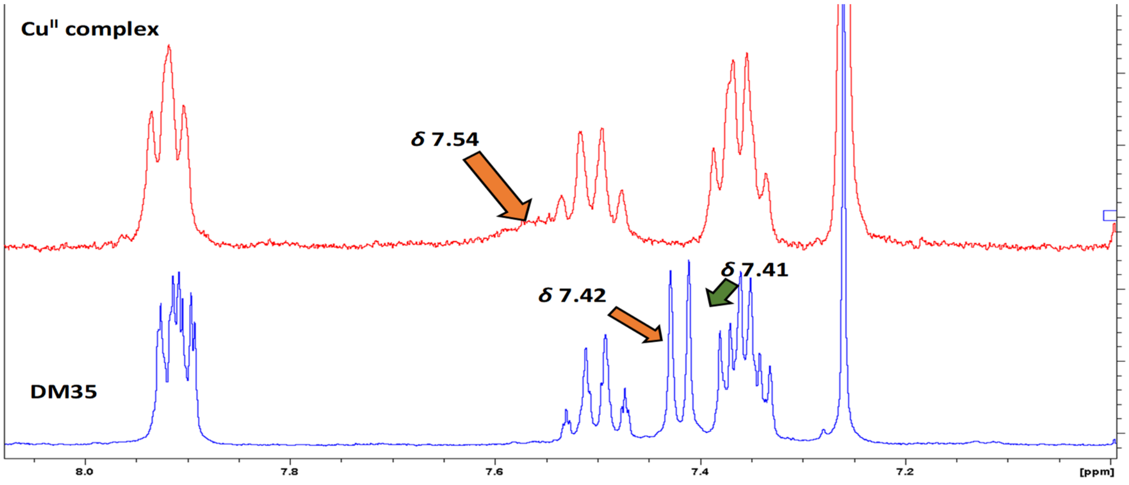

Figure S27. ^{1}H NMR spectra of **DM35** and its complex with $Cu(OAc)_2{\cdot}H_2O$, triazole proton.

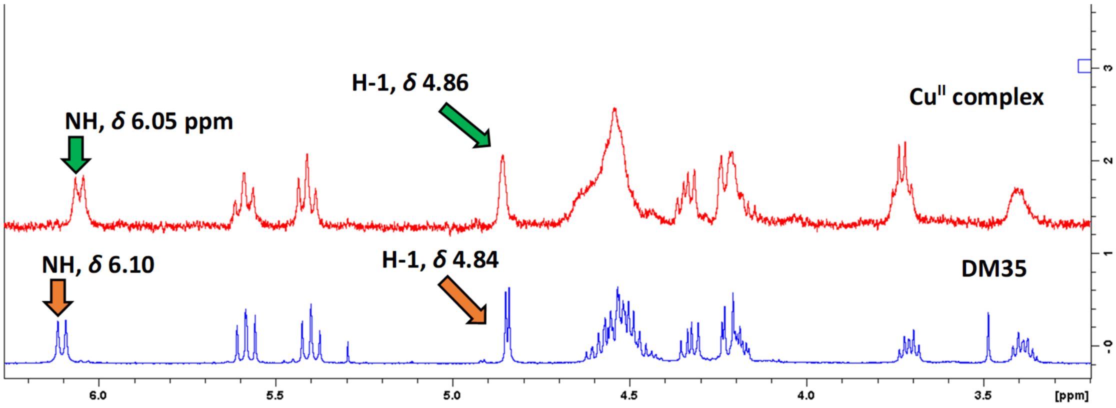

Figure S28. Stacked ^{1}H NMR spectra of **DM35** and its Cu^{II} complex with $Cu(OAc)_2{\cdot}H_2O$, anomeric proton.

LM28 with $CuSO_4 \cdot 5H_2O$

To a 25 mL RBF, **LM28** (10.0 mg, 0.015 mmol, 1.0 equiv) was dissolved in 2.0 mL of $EtOH:H_2O$ (v/v 1:1) and $CuSO_4 \cdot 5H_2O$ (3.8 mg, 0.015, 1.0 equiv) was added. The reaction mixture was refluxed for 2.0 hours to observe clear solution. The ^1H NMR at 2.0 hour showed down field shift of triazole peak. The reaction was stopped, and the solvent was removed under pressure to obtain white solid.

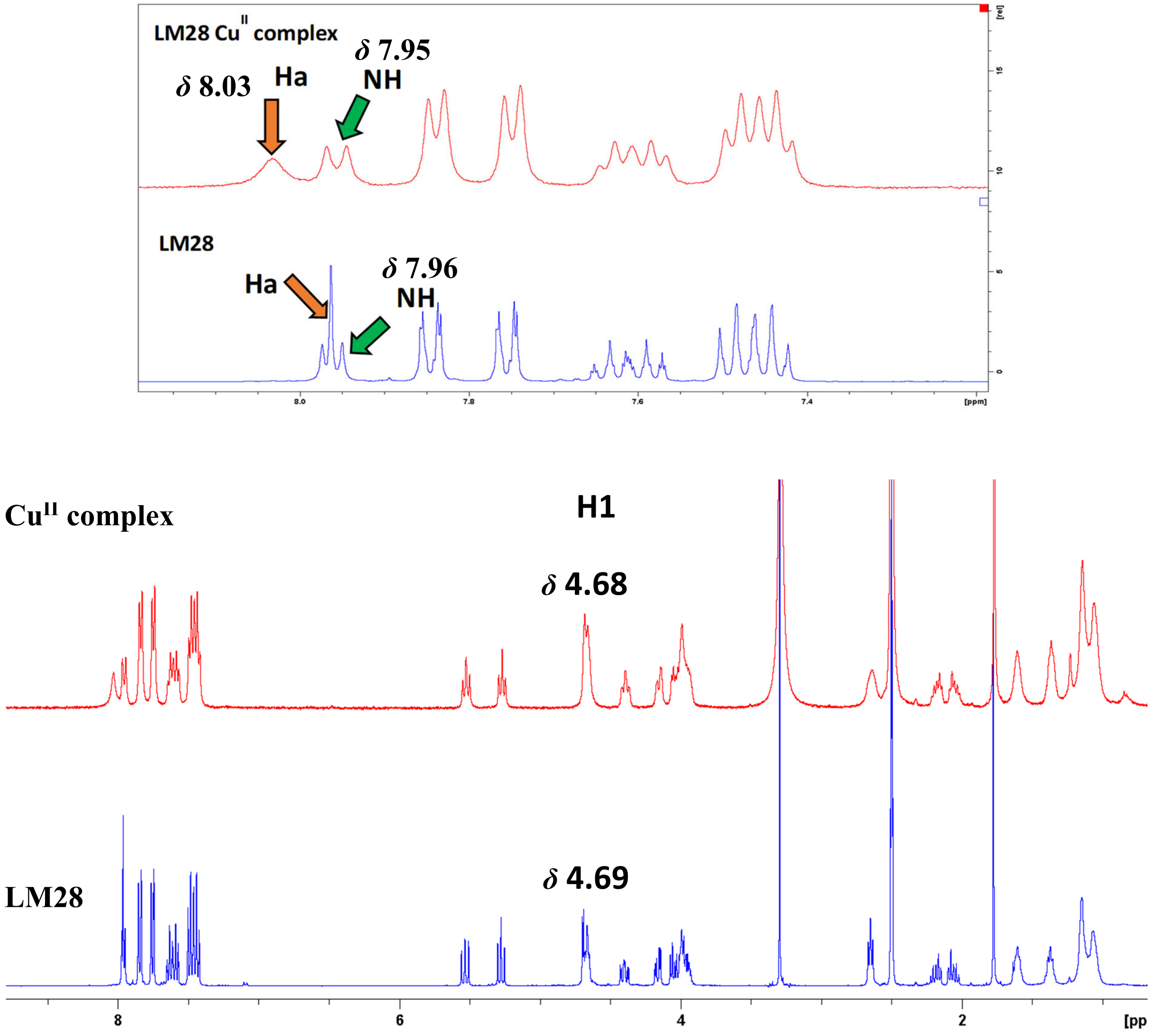

Figure S29. Stacked ^1H NMR spectra of **LM28** complex with $CuSO_4 \cdot 5H_2O$ full range, top insert shows NH and triazole proton.

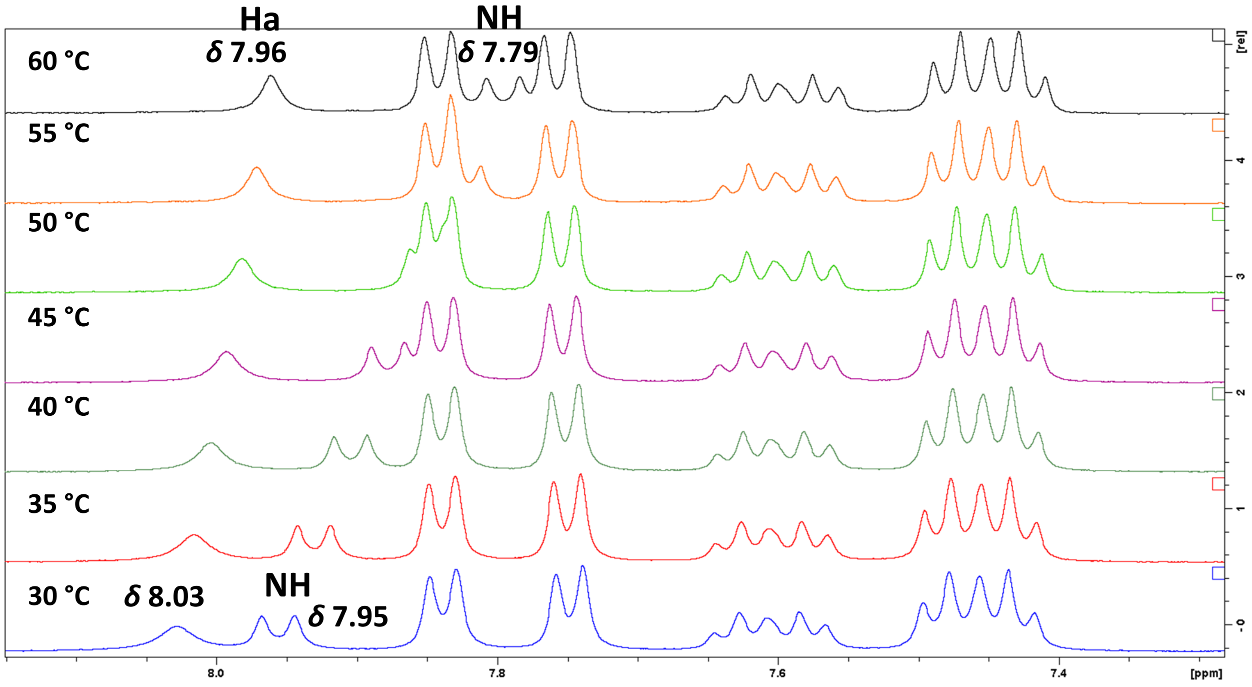

Figure S30. Stacked ^{1}H NMR spectra of **LM28** Cu^{II} complex with $CuSO_4{\cdot}5H_2O$.

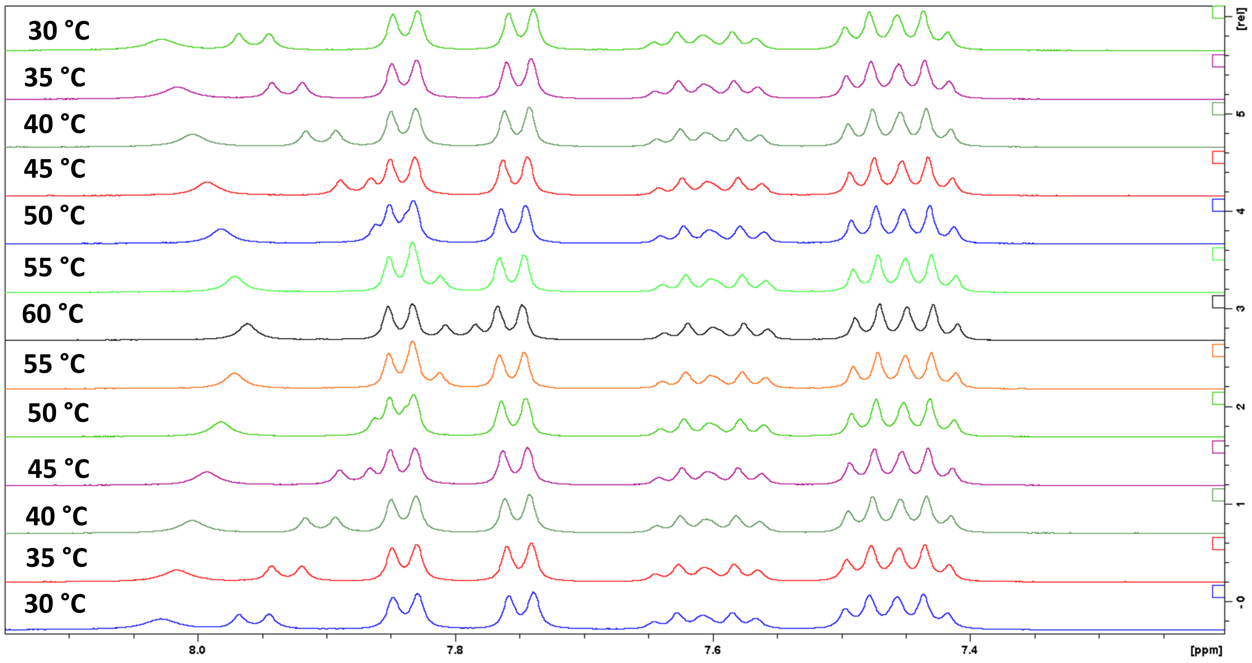

Figure S31. Stacked ^{1}H NMR spectra of **LM28** Cu^{II} complex with $CuSO_4{\cdot}5H_2O$, anomeric proton, increasing and decreasing temperatures.

Compound **24** with $CuSO_4·5H_2O$

Compound **24** (16.0 mg, 0.03 mmol, 1.0 equiv) was dissolved in 2.0 mL of EtOH:H_2O (v/v 1:1) and $CuSO_4·5H_2O$ (5.8 mg, 0.016, 1.0 equiv) was added. The reaction mixture was refluxed for 3 hours to observe clear solution. The ^{1}H NMR at 3.0 hour showed disappearance of triazole peaks. Only broad peak (hump) appeared downfield due to the paramagnetism. The reaction was stopped, and the solvent was removed under pressure to obtain light green solid.

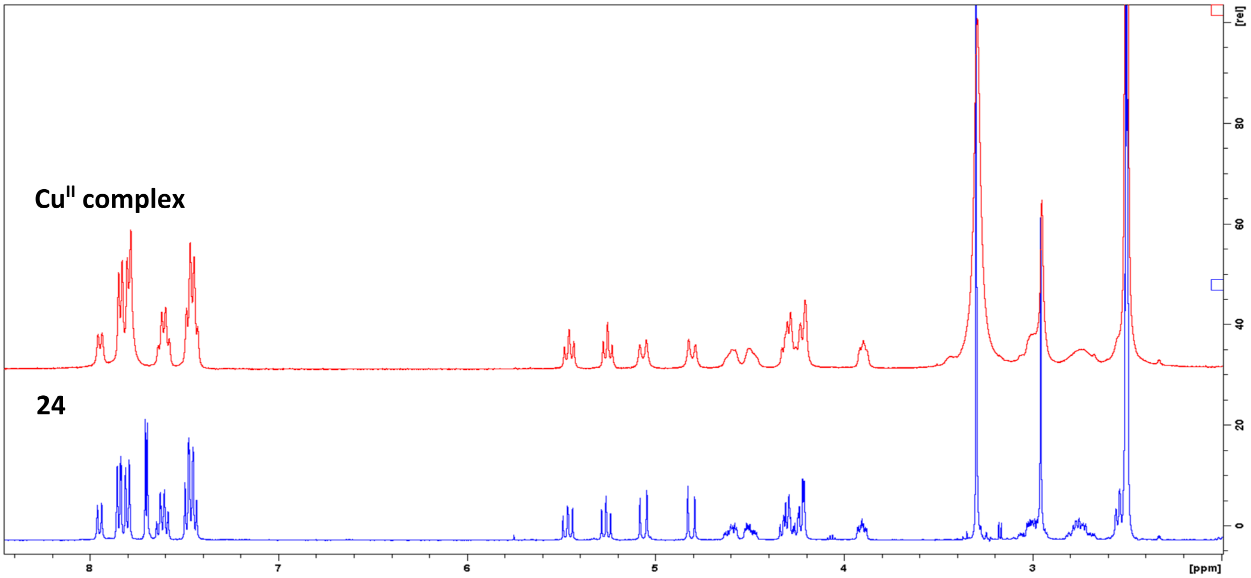

Figure S32. Overlay of ^{1}H NMR spectra of compound **24** and its Cu^{II} complex with $CuSO_4·5H_2O$.

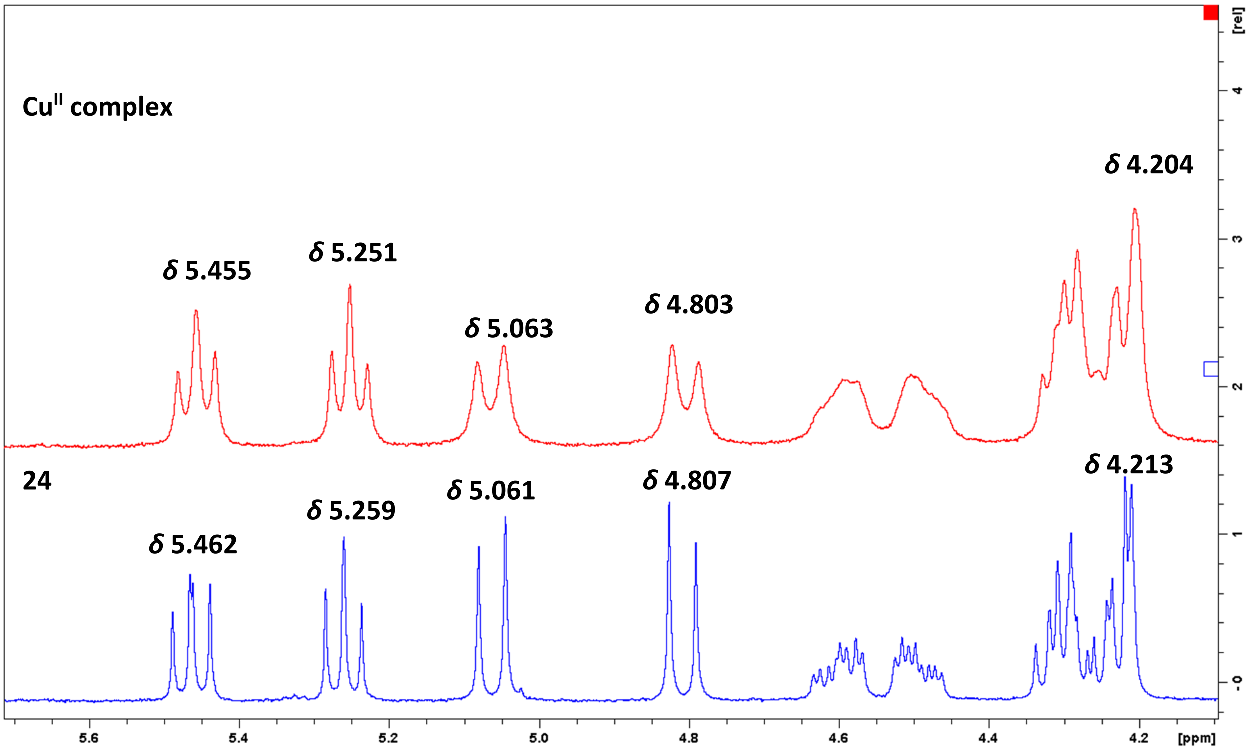

Figure S33. Stacked ^{1}H NMR spectra of compound **24** and its Cu^{II} complex with $CuSO_4.5H_2O$.

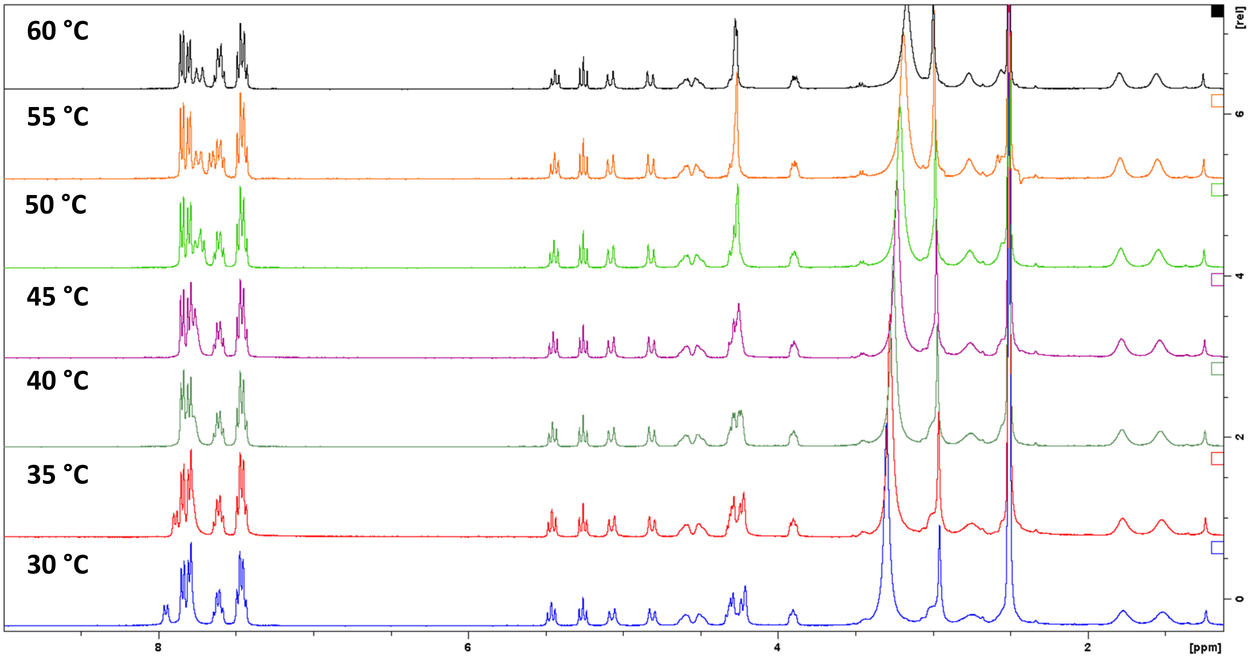

Figure S34. Stacked ^{1}H NMR spectra of compound **24** Cu^{II} complex with $CuSO_4·5H_2O$ at different temperature.

VI. Screening Macrocycles for Azide Alkyne Cycloadditions

1. General method for the cycloaddition reactions of sugar azide with alkynes

The screening reactions were carried out in a Heidolph carousel 12 plus reaction station system with 20 mL glass vessels. The click test reactions were performed using the apparatus under nitrogen atmosphere at controlled temperatures using similar method as described. Control experiments without macrocycles were carried out in the same batch under the same conditions with other reactions. The reaction vessel was flushed with nitrogen, about 40.0 mg of the sugar azide **26** was added to then macrocycles were then added, followed by 1.0 mL of ethanol and the corresponding alkynes (1.2 to 1.5 equiv), followed by addition of 1.0 mL $CuSO_4$ aqueous solution and lastly NaAsc. The reaction vessel was capped and nitrogen balloon was used to maintain the reaction under nitrogen atmosphere. The reaction was set to stir at 1000 rpm and temperature was set at 30.0 ºC. $CuSO_4{\cdot}5H_2O$ (54.0 mg) was dissolved in 10.0 mL of DI water (degassed) to prepare the stock solution for reactions of 40.0 mg of the azide for phenyl acetylene; and 27.0 mg of $CuSO_4{\cdot}5H_2O$ was dissolved in 10 mL of DI water (degassed) to make the stock solution for 1-octyne.

2. Tables and stacked ^{1}H NMR spectra of click reaction catalyst screening

2.1 Selected screening for phenylacetylene

Synthesis of compound **28**,[5,6] general procedure for Tables 2 and S1

Sugar azide (40.0 mg, 0.107 mmol, 1.0 equiv) and Phenylacetylene (17 µL, 0.16 mmol, 1.5 equiv) were dissolved in EtOH: H_2O (v 1:1, 2.0 mL), then $CuSO_4{\cdot}5H_2O$ (5.36 mg, 0.02 mmol, 0.2 equiv), sodium ascorbate (106 mg, 0.05 mmol, 0.5 equiv) were added to the reaction mixture. To this mixture macrocycle **DM35** (1.97, 2.5 mol%) was added. The reaction was stirred rt for 1.0 h to at which time the starting material was full converted to product, as indicated by ^{1}H NMR. The reaction was stopped, and solvent was removed using a rotavap, to obtain the crude, which was further purified with flash chromatography using eluent of DCM to 2% MeOH/DCM to obtain the desired product as white solid (49 mg, 96%), R_f = 0.29 in 5% MeOH/DCM. Additional screening reaction was carried out using 1.5 equivalent of phenylacetylene as shown in Table S2.

Table S1. Effect of the macrocycle on catalyzing the click chemistry using phenyl acetylene

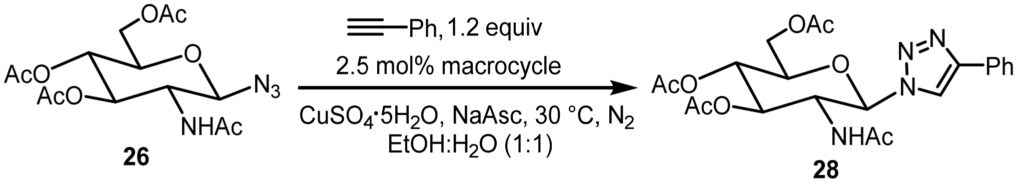

Entry	MC code	Conversion (%) at 1 h	Conversion (%) at 2 h	Conversion (%) at 5 h	Conversion (%) at 9 h
**1**		-	15	21	N/A
**2**	**LM24**	38	50	57	68
**3**	**LM26**	57	61	78	85
**4**	**LM28**	57	61	67	79
**5**	**LM34**	55	66	79	93
**6**	**LM36**	56	60	77	89
**7**	**LM38**	49	49	51	51
**8**	**DM24**	51	51	54	58
**9**	**DM25**	68	84	100	
**10**	**DM34**	53	60	61	65
**11**	**DM35**	68	81	100	

Condition: Sugar azide (1.0 equiv, 40.0 mg), $CuSO_4 \cdot 5H_2O$ (0.2 equiv, 5.4 mg), phenylacetylene (1.2 equiv, 14.0 µL), EtOH/H_2O (v/v 1:1, 2.0 mL), macrocycle (2.5 mol%), NaAsc (0.4 equiv, 8.5 mg).

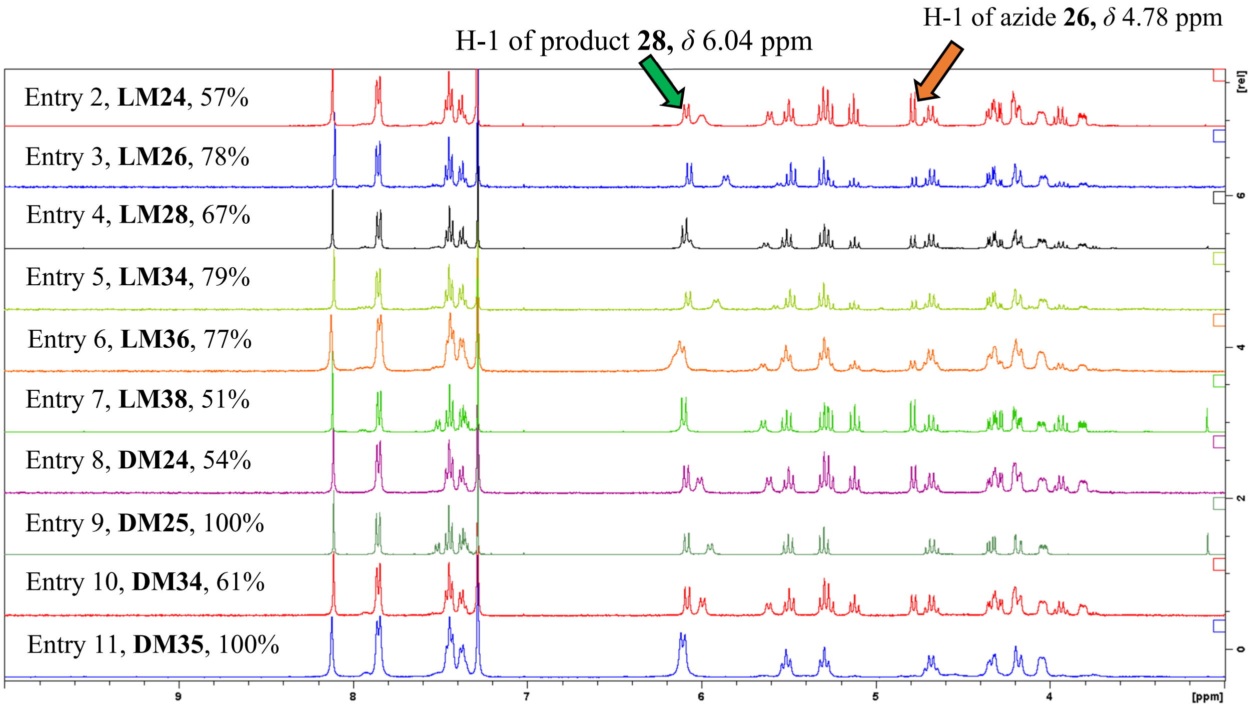

Figure S35. Overlay of ^{1}H NMR spectra of entries 2-11 at 5 h.

Table S2. Effect of the macrocycle on catalyzing the click chemistry using phenyl acetylene

Entry	MC code	mol% of MC	Conversion (%) at 0.5 h	Conversion (%) at 2 h	Conversion (%) at 5 h
1			18	18	21
2	**LM26**	5.0	51	74	100
3	**LM34**	5.0	42	51	94
4	**LM36**	5.0	27	34	87
5	**DM24**	2.5	43	56	70
6	**DM25**	2.5	46	59	100
7	**DM34**	2.5	46	54	68
8	**DM35**	2.5	35	42	100
9	**22**	2.5	-	10	20
10	**24**	2.5	-	27	56

Conditions: Sugar azide (1.0 equiv, 40.0 mg), $CuSO_4 \cdot 5H_2O$ (0.2 equiv, 5.4 mg), phenylacetylene (1.5 equiv, 17.0 μL), EtOH/H_2O (v/v 1:1, 2.0 mL), NaAsc (0.5 equiv, 10.6 mg), 0.025 or 0.05 equivalent of the macrocycles.

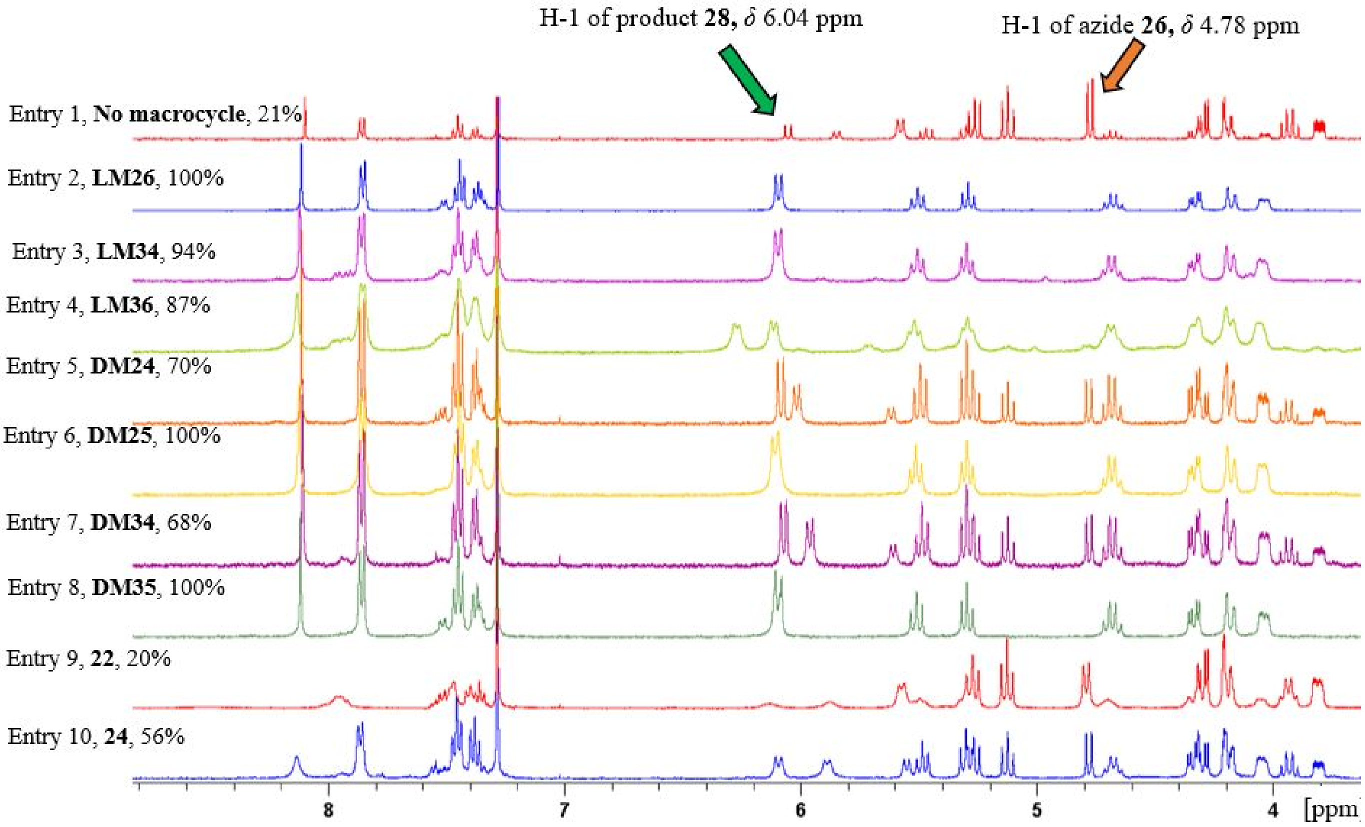

Figure S36. Overlay of ^{1}H NMR spectra of entries 1-10 at 5 h.

2.2 Selected screening for 1-octyne: The initial screening was done using 0.1 equiv. of $CuSO_4 \cdot 5H_2O$ and 2.5 mol% macrocycle, the results are shown in Table S3, stacked NMR spectra are shown in Figure S37. Further screening used 5.0 mol% for mono-triazole macrocycles and 1.5 equiv of 1-octyne and 0.3 equiv. of sodium ascorbate and the results are shown in Table S4 and Figure S38.

Synthesis of compound **29**,[5] general procedure and one example for Table 3

Sugar azide **26 (**40.0 mg, 0.107 mmol, 1.0 equiv) and 1-octyne (23.7 µL, 0.16 mmol, 1.5 equiv) were dissolved in EtOH: H_2O (v 1:1, 2.0 mL), then $CuSO_4 \cdot 5H_2O$ (2.7 mg, 0.01 mmol, 0.1 equiv), sodium ascorbate (6.4 mg, 0.03 mmol, 0.3 equiv) were added to the reaction mixture. To this mixture macrocycle **DM34** (1.93 mg, 2.5 mol%) was added. The reaction was stirred rt for 1.0 h to at which time the starting material was full converted to product, as indicated by ^{1}H NMR. The reaction was stopped, and solvent was removed using a rotavap, to obtain the crude, which was

further purified with flash chromatography using eluent of DCM to 2% MeOH/DCM to obtain the desired product as white solid (45 mg, 87%), R_f = 0.32 in 5% MeOH/DCM.

Table S3. Effect of the macrocycles on copper mediated click reaction of 1-octyne.

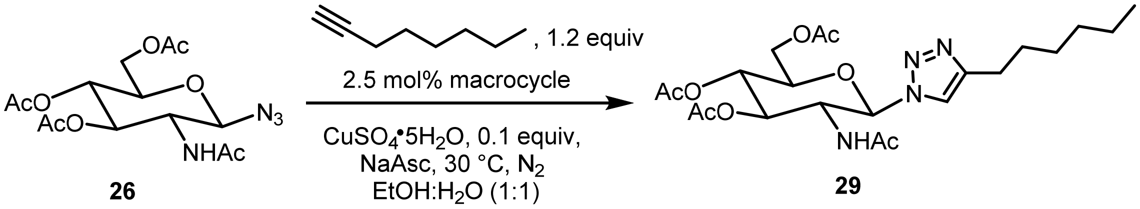

Entry	MC code	mol% of MC	Conversions (%)		
			1 h	2 h	5 h
1			-	25	28 (6 h)
2	**LM24**	2.5	8	9	13
3	**LM26**	2.5	7	9	19
4	**LM28**	2.5	14	16	25
5	**LM34**	2.5	25	25	26
6	**LM36**	2.5	15	16	25
7	**LM38**	2.5	10	16	27
8	**DM24**	2.5	22	27	42
9	**DM25**	2.5	17	24	53
10	**DM34**	2.5	83	93	97
11	**DM35**	2.5	17	26	32

Condition: Sugar azide (1.0 equiv, 40.0 mg), $CuSO_4 \cdot 5H_2O$ (0.1 equiv, 2.7 mg), 1-octyne (1.2 equiv, 19.0 µL), EtOH/H_2O (v/v 1:1, 2.0 mL), macrocycle (2.5 mol%), NaAsc (0.2 equiv, 4.3 mg)

H-1 of azide **26,** δ 4.78 ppm

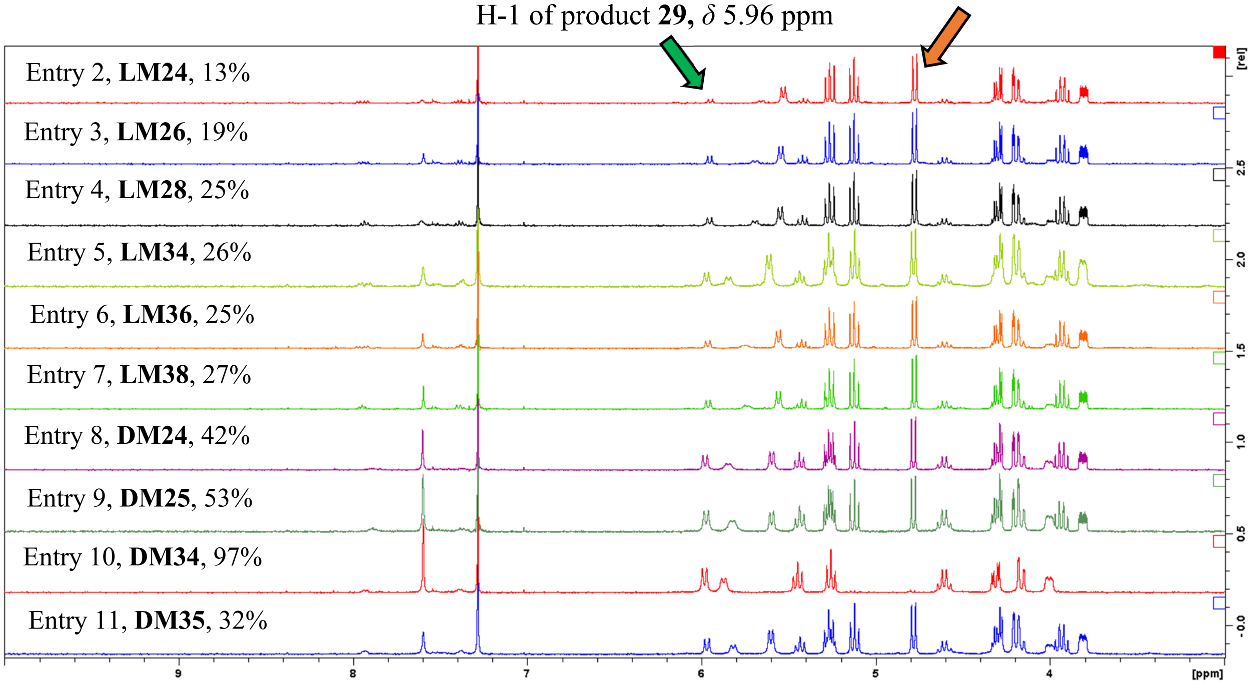

Figure S37. Overlay of ^{1}H NMR spectra of entries 2-11 at 5 hours.

Table S4. Effect of the macrocycle on catalyzing the click reaction of 1-octyne

Entry	MC code	mol% of MC	Conversion (%) at 1 h	Conversion (%) at 2 h	Conversion (%) at 5 h
1			11	14	20
2	**LM26**	5.0	8	12	25
3	**LM34**	5.0	16	18	28
4	**LM36**	5.0	12	14	23
5	**DM24**	2.5	27	35	46
6	**DM25**	2.5	12	25	57
7	**DM34**	2.5	100		
8	**DM35**	2.5	20	22	37

Condition: Sugar azide (1.0 equiv, 40.0 mg), $CuSO_4{\cdot}5H_2O$ (0.1 equiv, 2.7 mg), 1-octyne (1.5 equiv, 23.7 µL), EtOH/H_2O (v/v 1:1, 2.0 mL), NaAsc (0.3 equiv, 6.4mg)

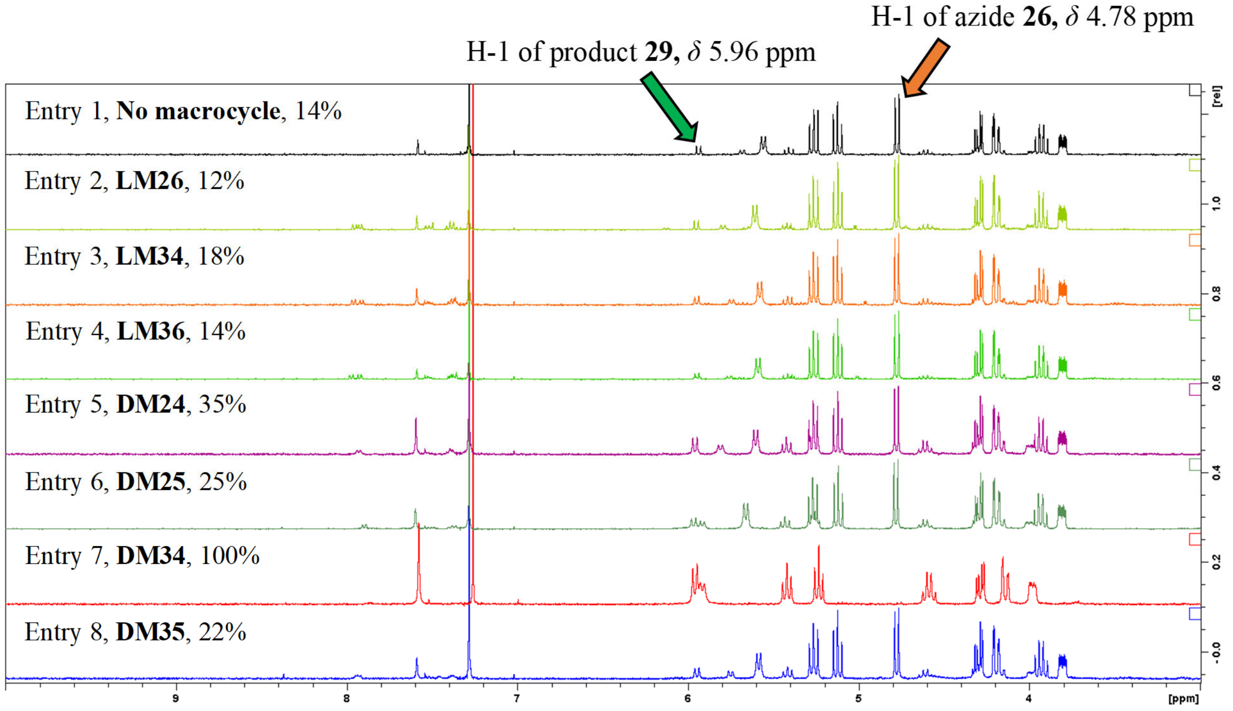

Figure S38. Overlay of ^{1}H NMR spectra of entries 1-5 and 8 at 2 hours and entry 7 at 1 hour.

2.3 Screening reactions for 5-phenyl-1-pentyne

Synthesis of compound **30**,[5] general procedure and one example for Table 4

Sugar azide (40.0 mg, 0.107 mmol, 1.0 equiv) and 5-Phenyl-1-Pentyne (24.5 µL, 0.16 mmol, 1.5 equiv) were dissolved in EtOH: H_2O (v 1:1, 2.0 mL), then $CuSO_4 \cdot 5H_2O$ (1.34 mg, 0.005 mmol, 0.05 equiv), sodium ascorbate (2.13 mg, 0.01 mmol, 0.1 equiv) were added to the reaction mixture. To this mixture macrocycle **DM35** (1.97, 2.5 mol%) was added. The reaction was stirred rt for 1.0 h to at which time the starting material was full converted to product, as indicated by ^{1}H NMR. The reaction was stopped, and solvent was removed using a rotavap to obtain the crude, which was further purified with flash chromatography using eluent of DCM to 2% MeOH/DCM to afford the desired product as a white solid (48 mg, 86%), R_f = 0.32 in 5% MeOH/DCM.

Table S5. Screening results of 5-phenyl-1-pentyne with copper acetate

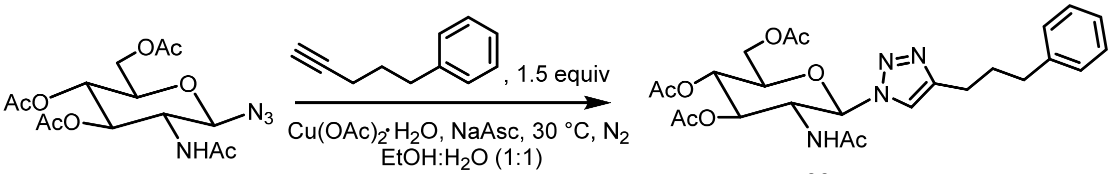

26 30

Entry	Macrocycle	$Cu(OAc)_2$	Conversion (%) at 1 h	Conversion (%) at 2 h	Conversion (%) at 5h	Conversion (%) at 10 h
1	No MC	0.01	/	/	17	37
**2**	**DM35,** 2.5%	0.01	25	/	77	99
**3**	No MC	0.02	/	13		
**4**	**DM35,** 2.5%	0.02	63	100		

Condition for Table S5: 26 (1.0 equiv, 40.0 mg), $Cu(OAc)_2 \cdot H_2O$ (0.02 equiv, 0.4 mg), 5-phenyl-1-pentyne (1.5 equiv, 24.5 μL, 23.2 mg), EtOH/H_2O (v/v 1:1, 2.0 mL), **DM35** (2.5 mol%), NaAsc (0.04 equiv, 0.9 mg). Condition for Table S6 are the same to above except $CuSO_4 \cdot 5H_2O$ (0.05 or other equiv, 1.3 mg), macrocycle (1.0-2.5 mol%), NaAsc (3 equiv. of $CuSO_4$).

Table S6. Screening results of 5-phenyl-1-pentyne using reduced copper sulfate

entry	Macrocycle	$CuSO_4$	Conversion (%) at 1 h	Conversion (%) at 2h	Conversion (%) at 5h
1	No MC	0.01	2	5	27
**2**	No MC	0.05	33	51	
3	No MC	0.1	95	100	
4	2.5% of **DM35**	0.01	29	52	74
5	1.0 % of **DM35**	0.02	58	88	100 (4h)
6	2.5% of **DM24**	0.02	62	88	100 (4h)
**7**	1.0 % of **DM25**	0.05	77	98	
**8**	2.5% of **DM34**	0.05	100		
**9**	2.5% of **DM35**	0.05	100		

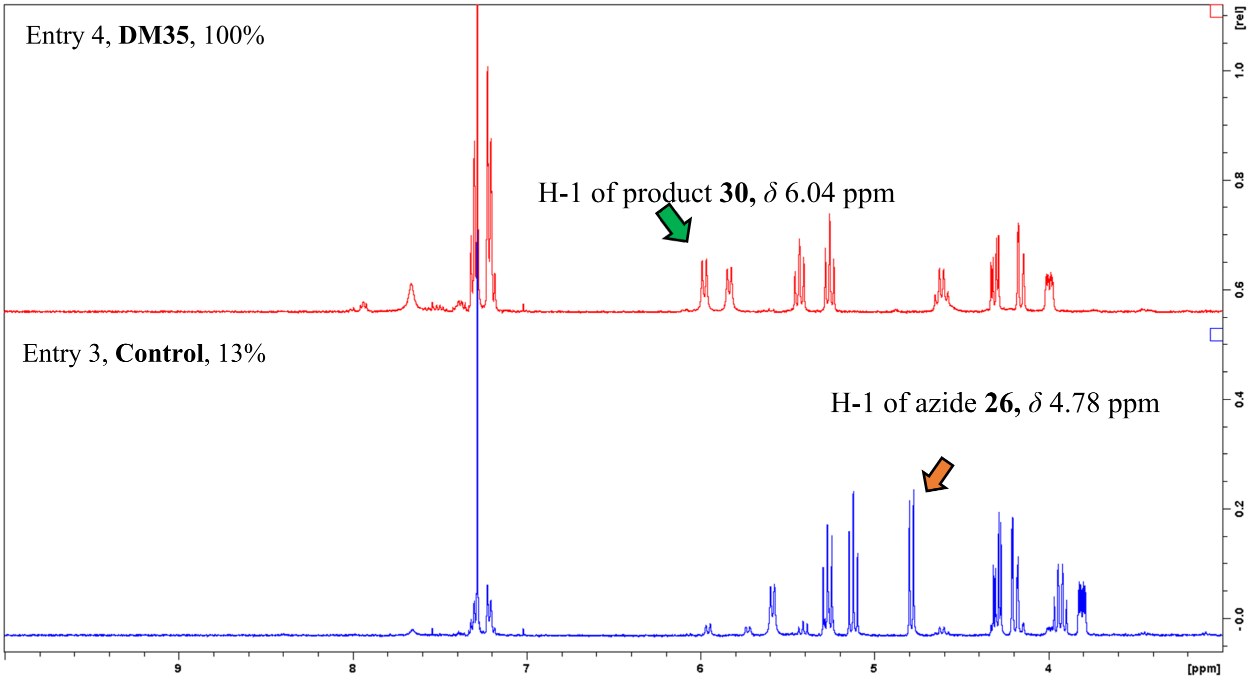

Figure S39. Overlay of ^{1}H NMR spectra of entries 1-2 in table S5 at 2 hours for $Cu(OAc)_2{\cdot}H_2O$.

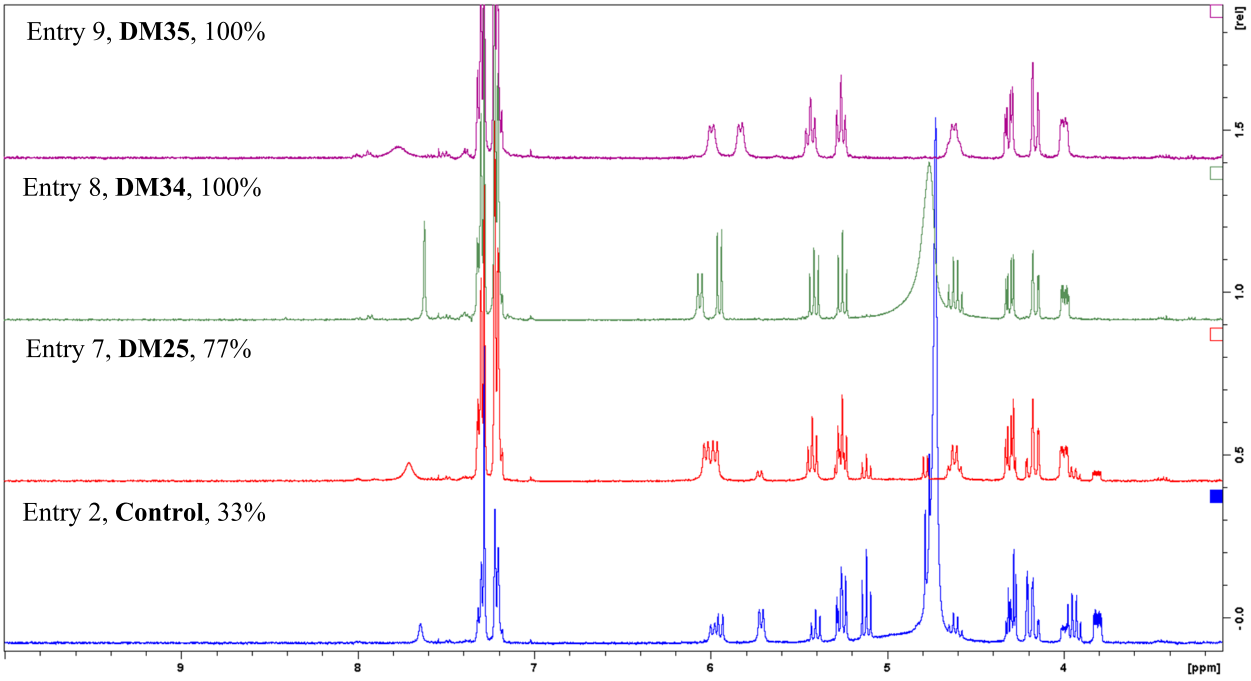

Figure S40. Overlay of ^{1}H NMR spectra of entries 2, 7-9 in Table S6 at 1 hour for 5% $CuSO_4{\cdot}5H_2O$

2.4 Screening reactions for 4-tert-butylphenylacetylene

Table S7. Effect of the macrocycles on copper mediated click reaction of sugar azide with 4-tert-butylphenylacetylene.

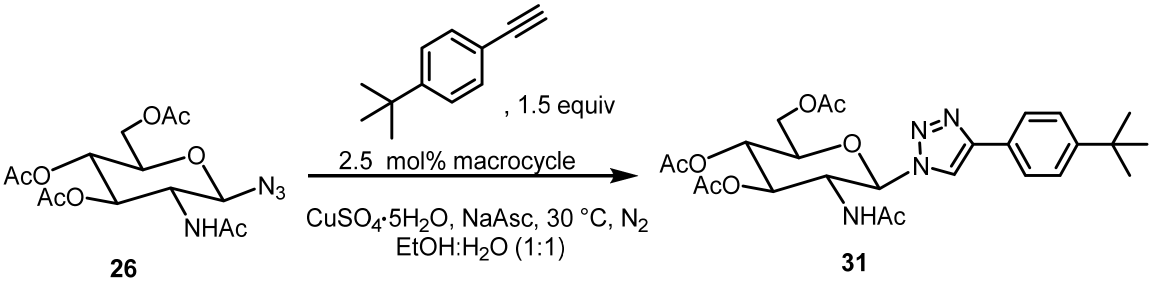

Entry		mol% of MC	Conversion (%) at 1 h	Conversion (%) at 2 h	Conversion (%) at 5 h	Conversion (%) at 20 h
1			-	4	10	27
2	**DM25**	2.5	26	47	80	100
3	**DM34**	2.5	7	15	34	70
4	**DM35**	2.5	19	28	45	100

Condition A: Sugar azide (1.0 equiv, 40.0 mg), $CuSO_4 \cdot 5H_2O$ (0.1 equiv, 2.7 mg), 4-tert-butylphenylacetylene (1.5 equiv, 29.0 µL), EtOH/H_2O (v/v 1:1, 2.0 mL), macrocycle (2.5 mol%), NaAsc (0.3 equiv, 6.4 mg).

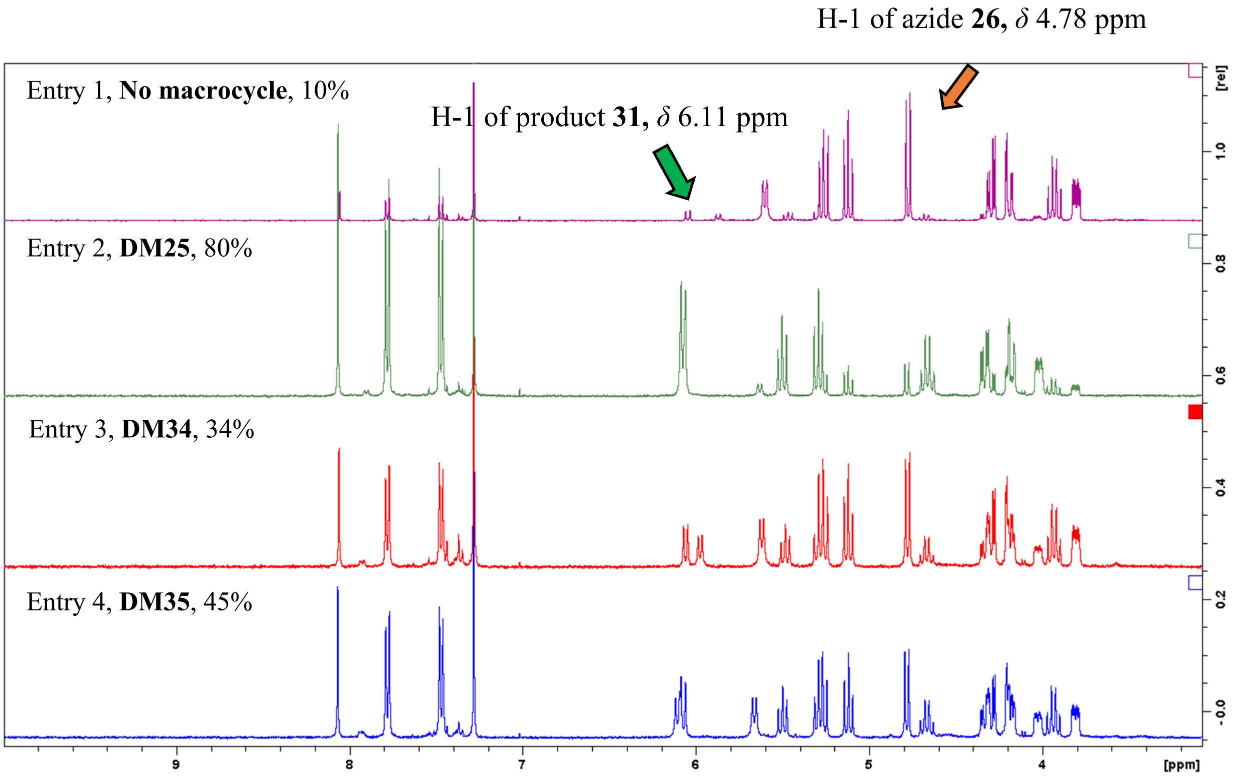

Figure S41a. Overlay of ^{1}H NMR spectra at 5 hours.

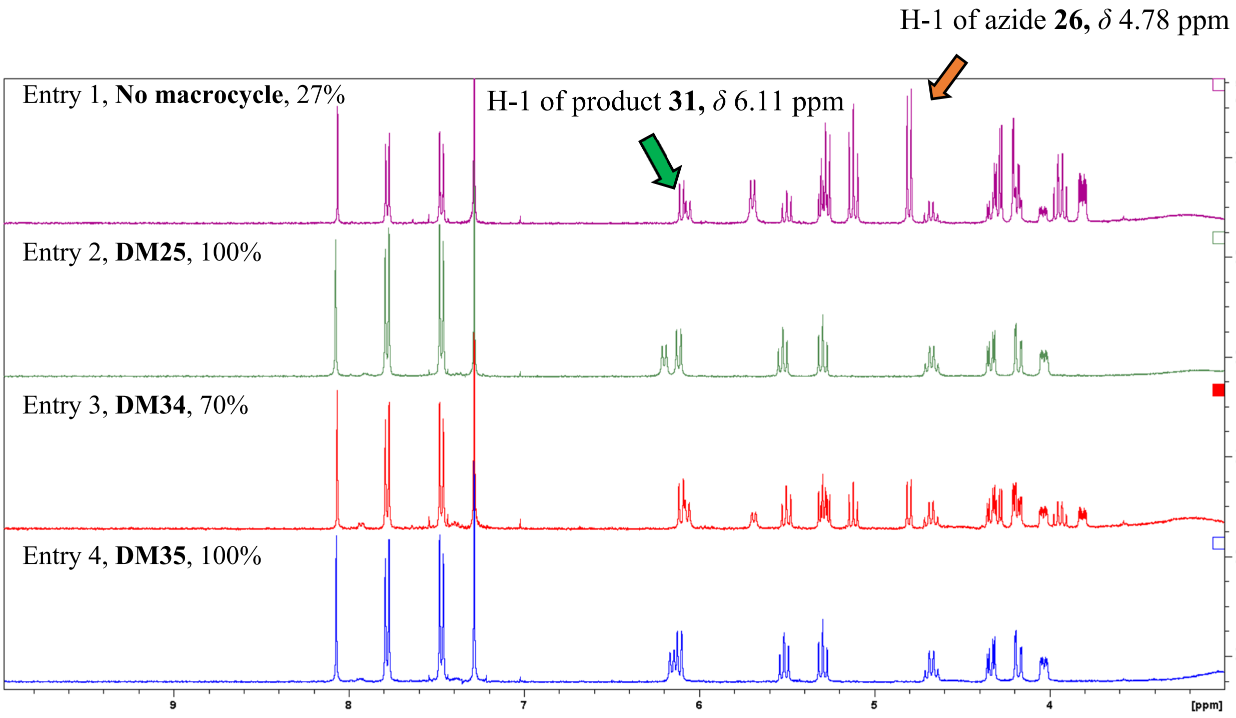

Figure S41b. Overlay of ^{1}H NMR spectra at 20 hours.

Table S8. Further screening reaction with p-t-butyl-phenylacetylene with increased copper amount

Entry		mol% of MC	Conversion (%) at 2 h	Conversion (%) at 5 h	Conversion (%) at 10 h
1		0	42	62	83
2	**DM24**	5.0	61	78	87
3	**DM25**	2.5	30	100	-
4	**DM25**	5.0	43	100	-
5	**DM34**	5.0	48	75	84
6	**DM35**	5.0	68	98	100

Condition B: Sugar azide (1.0 equiv, 40.0 mg), $CuSO_4 \cdot 5H_2O$ (0.2 equiv, 5.4 mg), 4-tert-butylphenylacetylene (1.5 equiv, 29.0 µL), EtOH/H_2O (v/v 1:1, 2.0 mL), macrocycle (2.5 mol%), NaAsc (0.4 equiv, 8.5 mg).

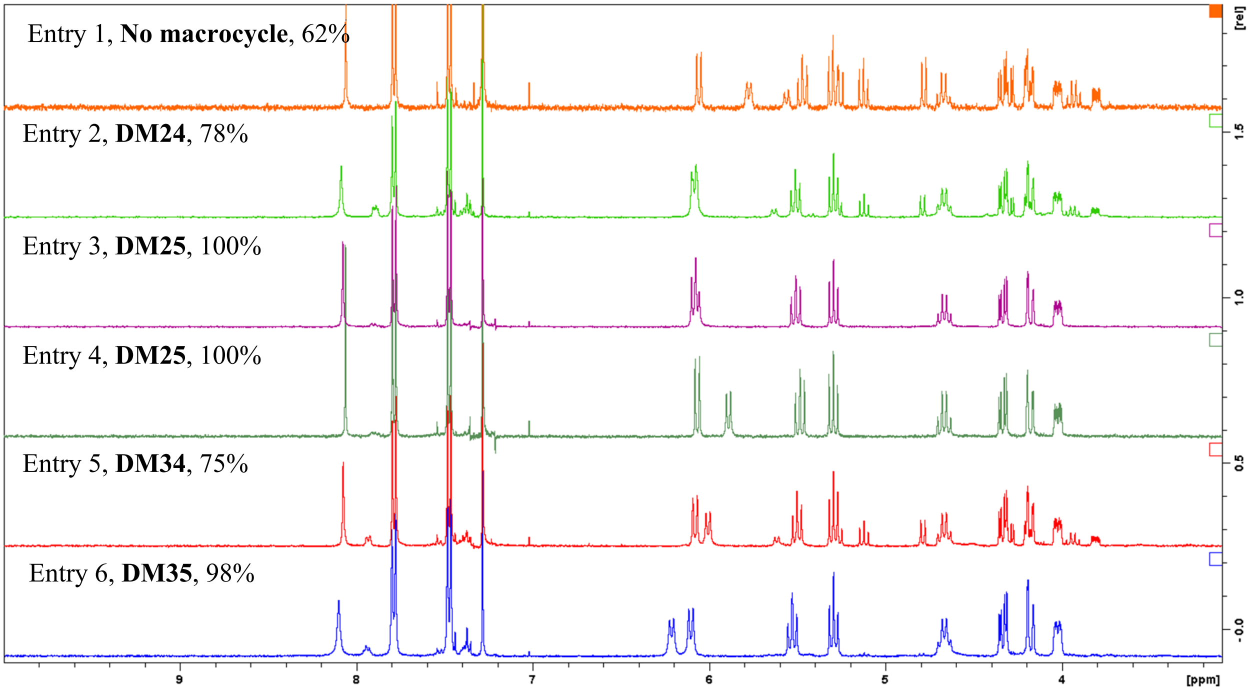

Figure S42. Overlay of ^{1}H NMR spectra of entries 1-6 at 5 hours.

VII. IR spectra for several macrolactones:

The IR spectra were obtained using a Bruker Platinum ATR (Alpha) FT-IR Spectrometer, the parameters are Resolution: 4 cm^{-1}, 64 Scans.

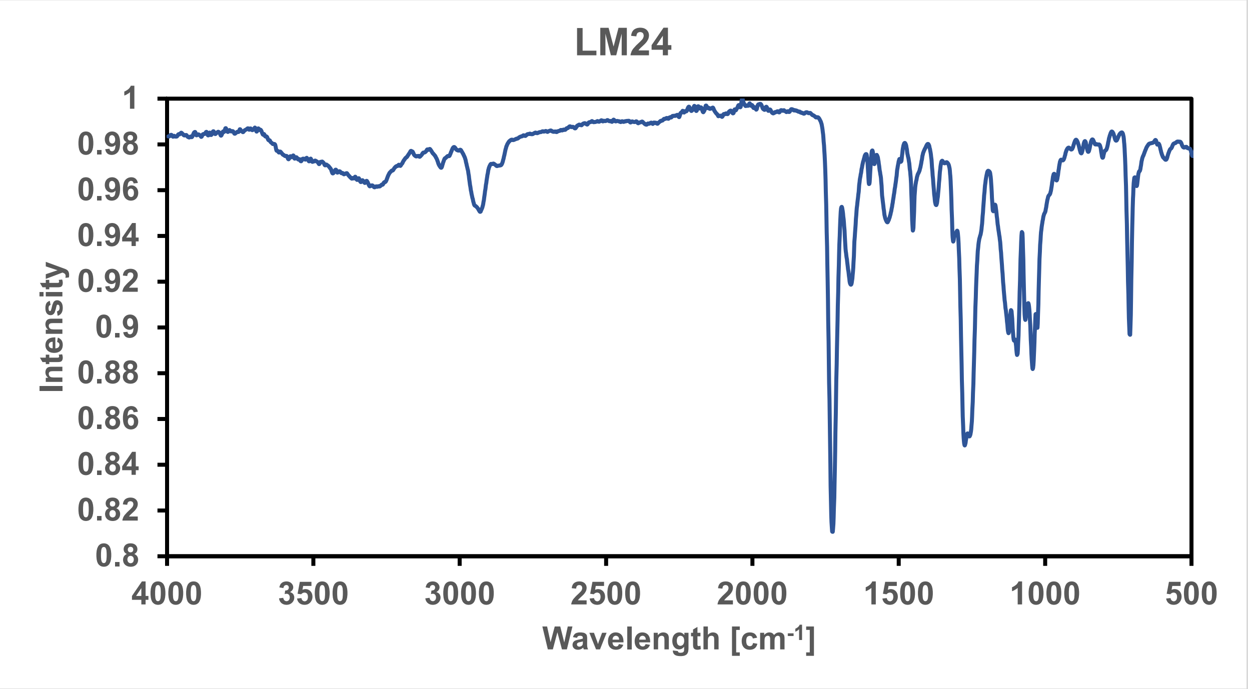

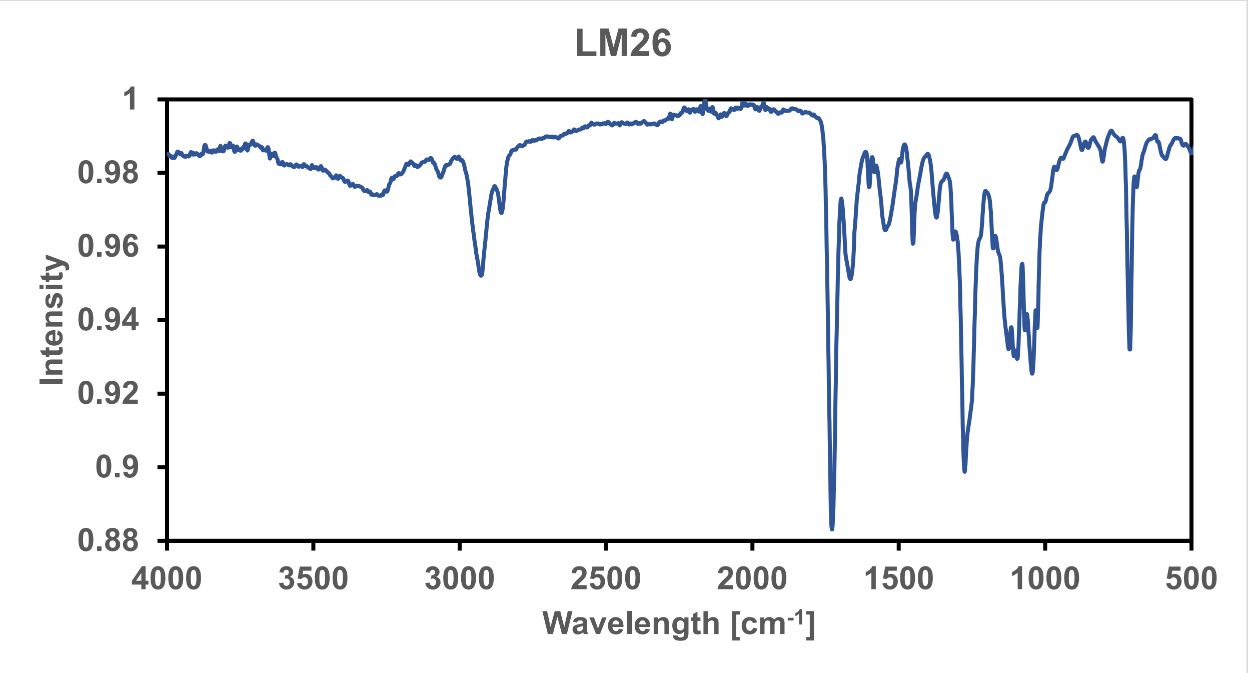

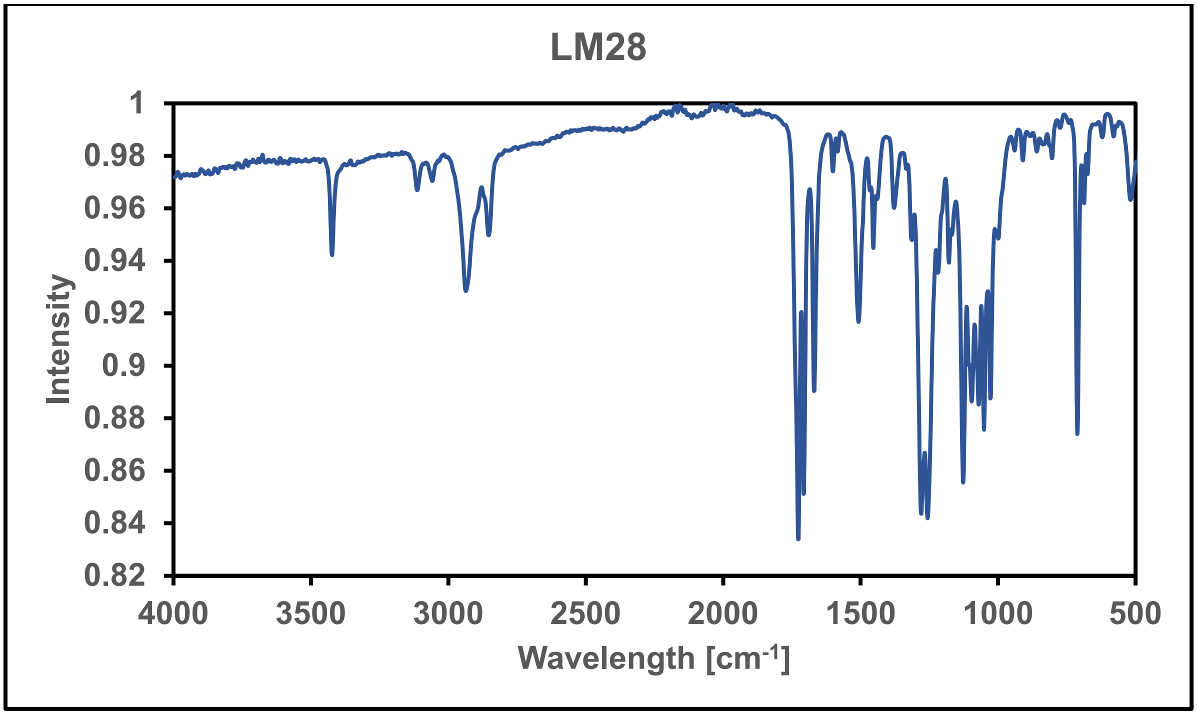
LM28
Intensity
1
0.98
0.96
0.94
0.92
0.9
0.88
0.86
0.84
0.82
4000
3500
3000
2500
2000
1500
1000
500
Wavelength [cm-1]

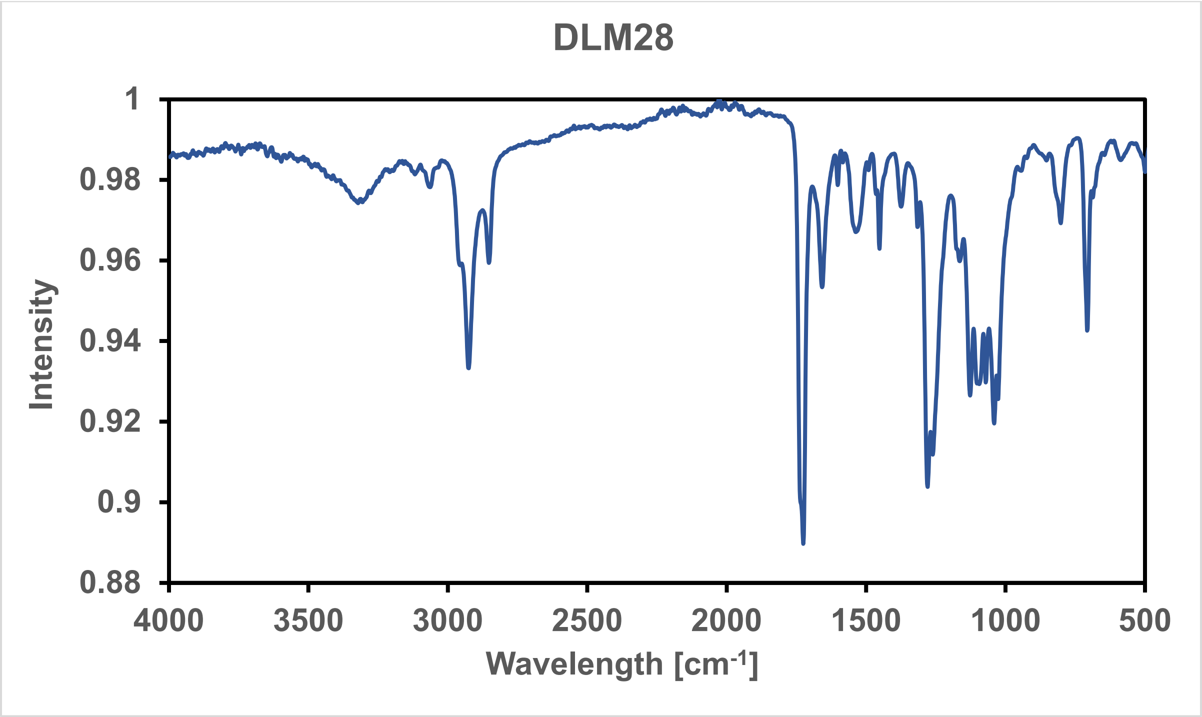
DLM28
Intensity
1
0.98
0.96
0.94
0.92
0.9
0.88
4000
3500
3000
2500
2000
1500
1000
500
Wavelength [cm-1]

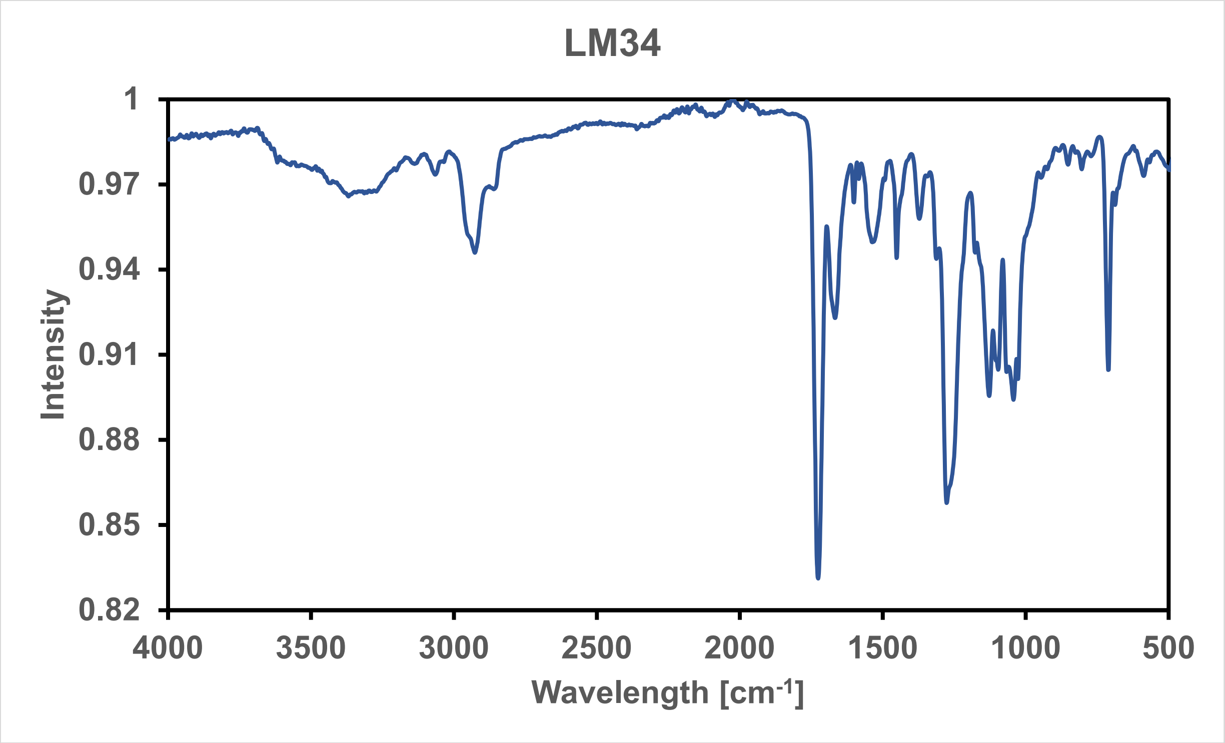
LM34
1
0.97
0.94
0.91
0.88
0.85
0.82
Intensity
4000
3500
3000
2500
2000
1500
1000
500
Wavelength [cm-1]

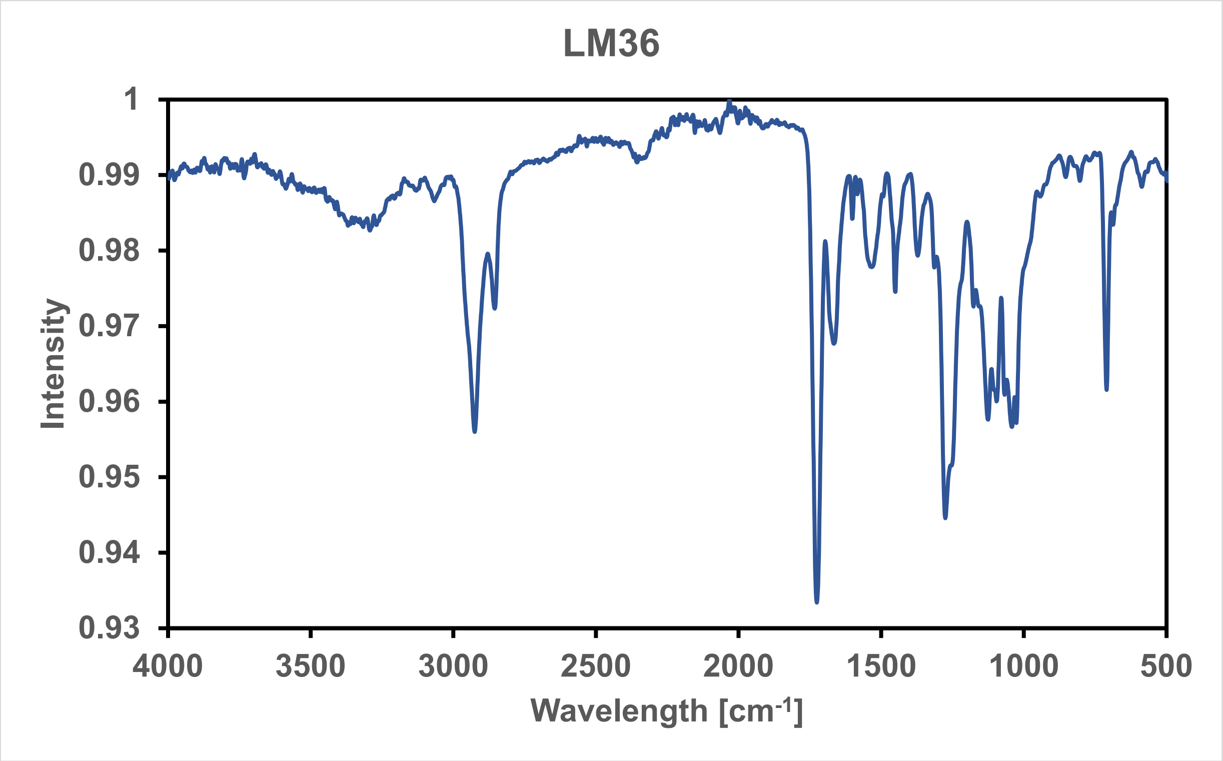
LM36
1
0.99
0.98
0.97
0.96
0.95
0.94
0.93
Intensity
4000
3500
3000
2500
2000
1500
1000
500
Wavelength [cm-1]

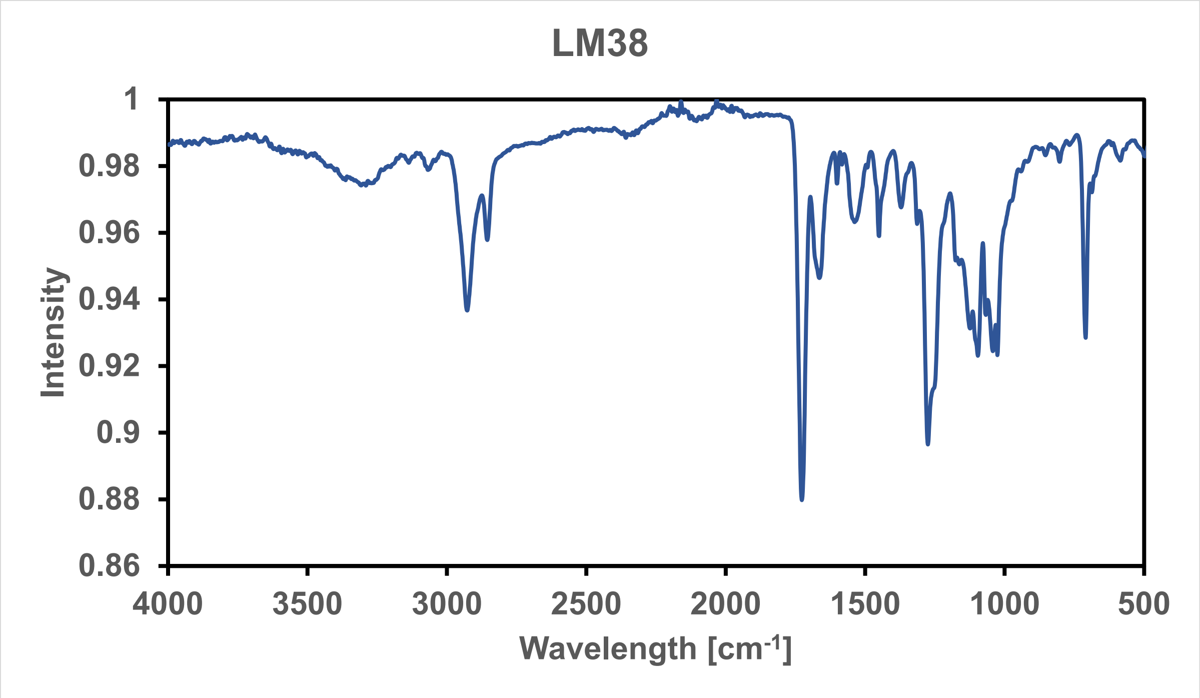
LM38
Intensity
Wavelength [cm-1]
1
0.98
0.96
0.94
0.92
0.9
0.88
0.86
4000
3500
3000
2500
2000
1500
1000
500

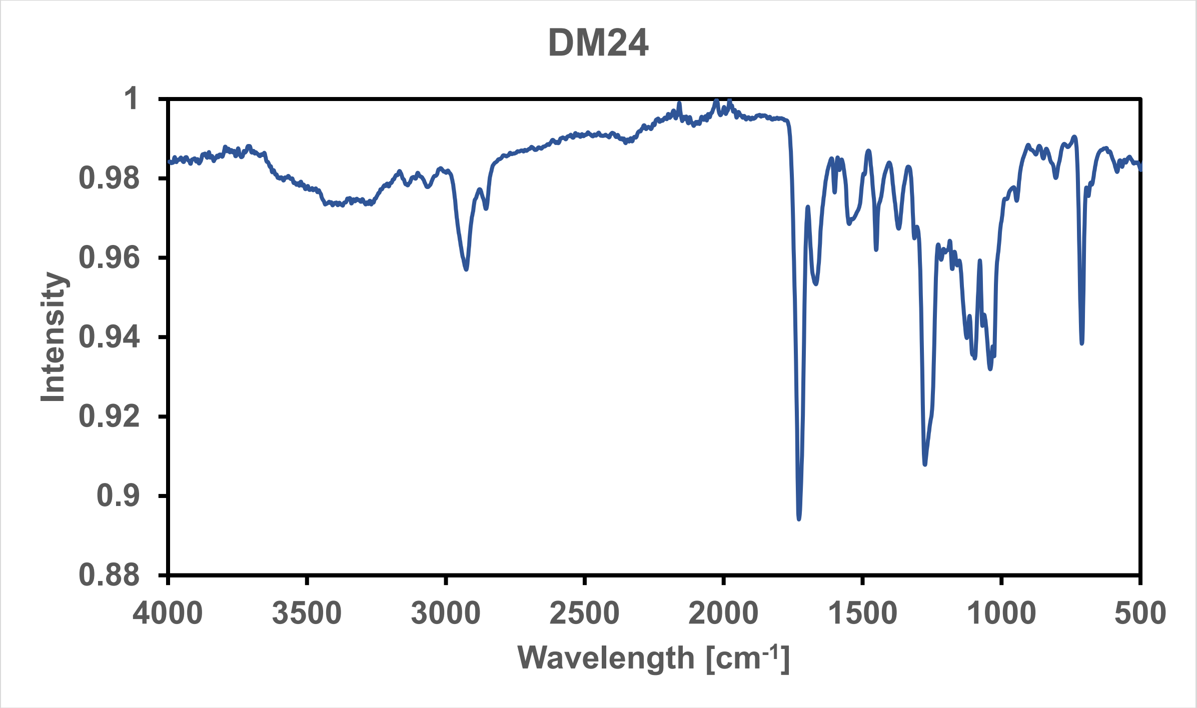
DM24
Intensity
Wavelength [cm-1]
1
0.98
0.96
0.94
0.92
0.9
0.88
4000
3500
3000
2500
2000
1500
1000
500

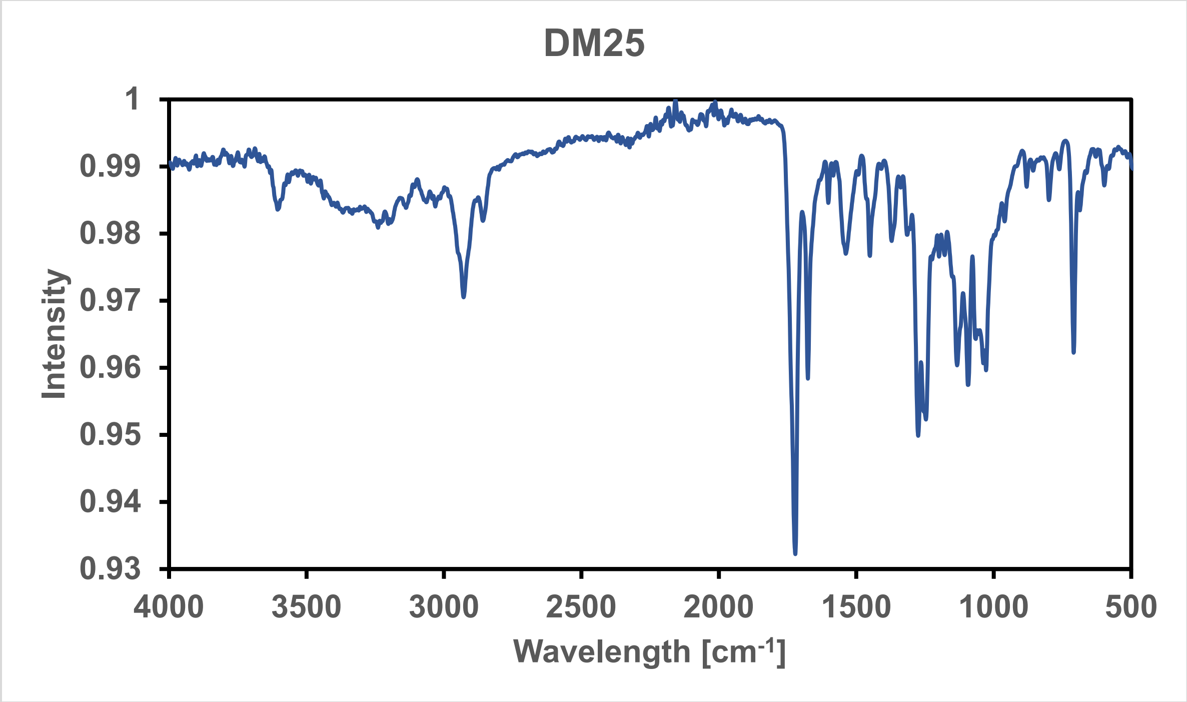
DM25
Intensity
Wavelength [cm-1]
1
0.99
0.98
0.97
0.96
0.95
0.94
0.93
4000
3500
3000
2500
2000
1500
1000
500

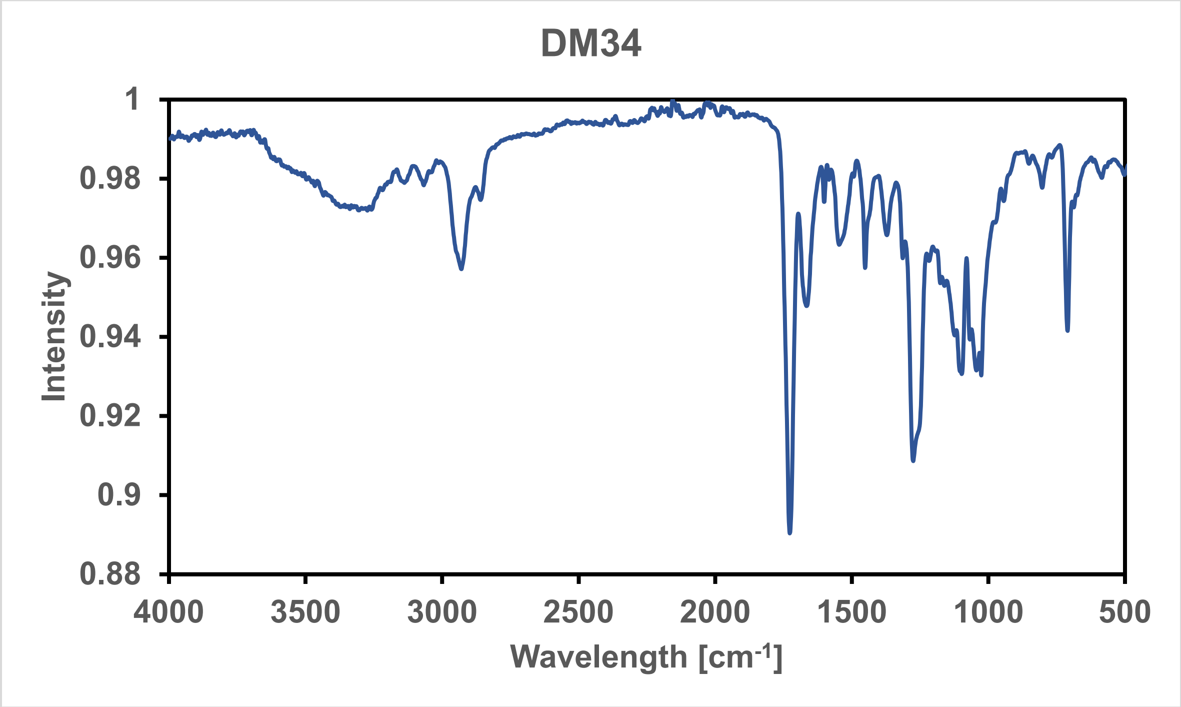
DM34
Intensity
Wavelength [cm-1]
1
0.98
0.96
0.94
0.92
0.9
0.88
4000
3500
3000
2500
2000
1500
1000
500

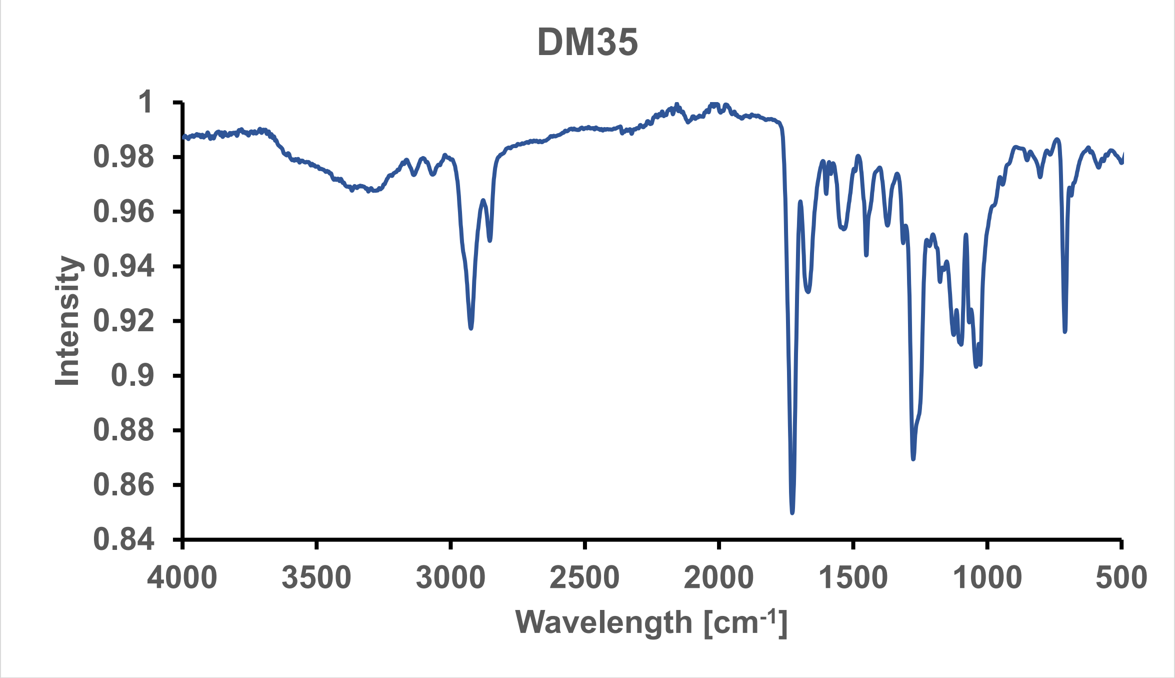
DM35
1
0.98
0.96
0.94
0.92
0.9
0.88
0.86
0.84
Intensity
4000
3500
3000
2500
2000
1500
1000
500
Wavelength [cm-1]

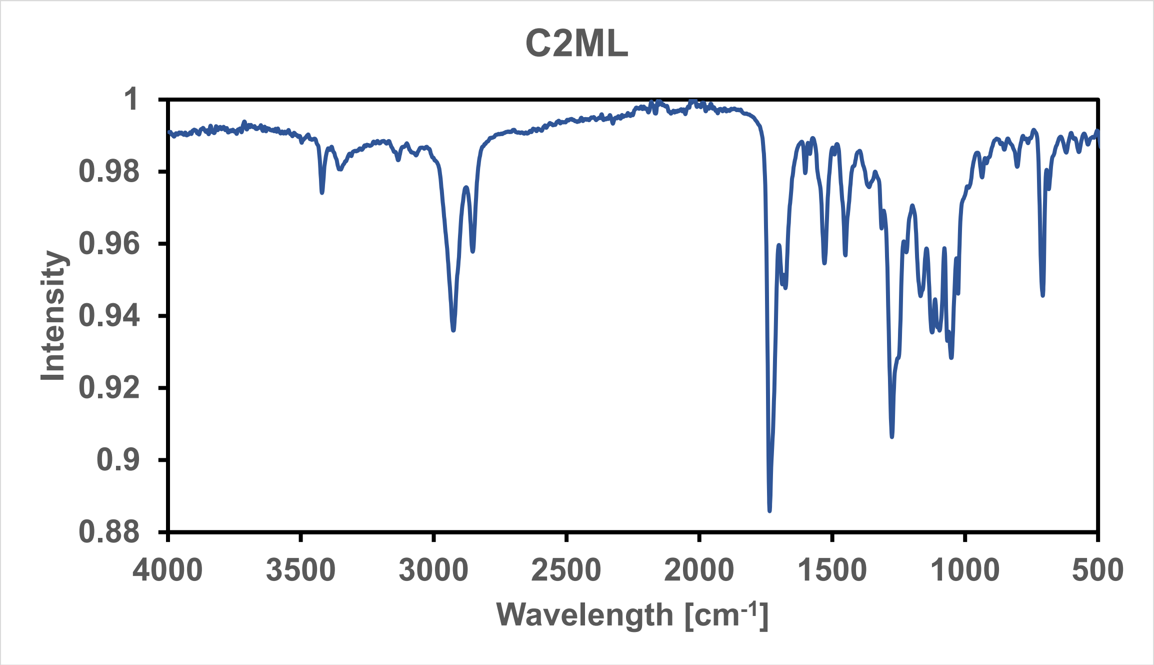
C2ML
1
0.98
0.96
0.94
0.92
0.9
0.88
Intensity
4000
3500
3000
2500
2000
1500
1000
500
Wavelength [cm-1]

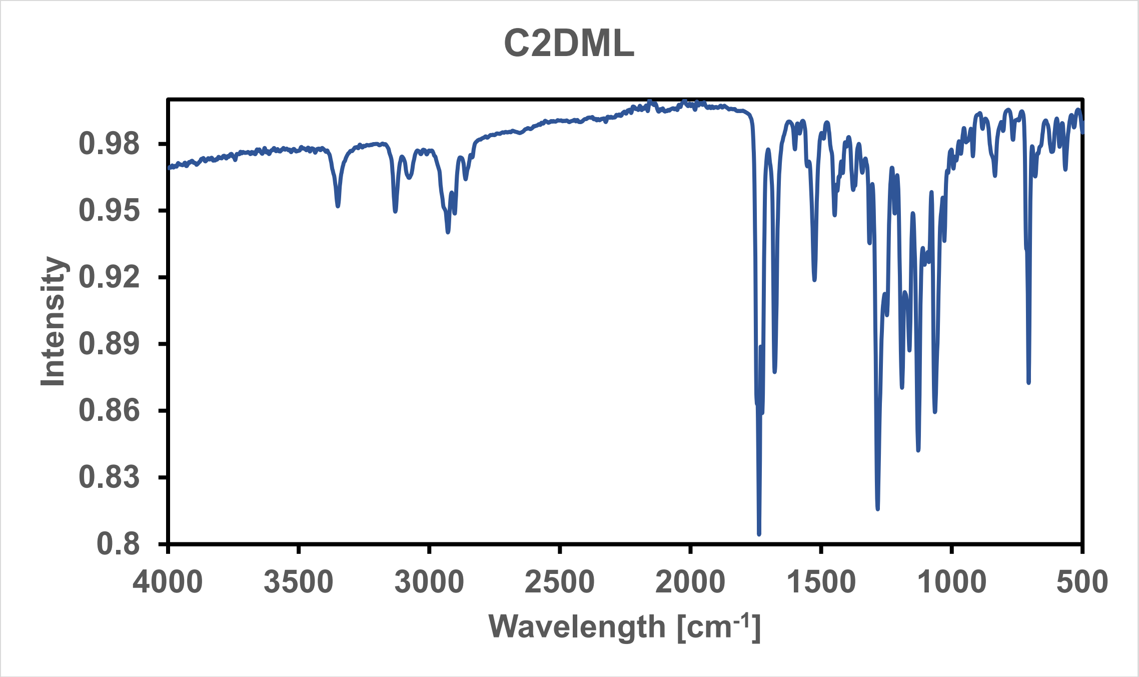

References:

(1) Disney, M. D.; Seeberger, P. H. The Use of Carbohydrate Microarrays to Study Carbohydrate-Cell Interactions and to Detect Pathogens. *Chem. Biol.* **2004**, *11*, 1701-1707.

(2) Roy, R.; Kim, J. M. Cu(II)-Self-assembling bipyridyl-glycoclusters and dendrimers bearing the Tn-antigen cancer marker: syntheses and lectin binding properties. *Tetrahedron* **2003**, *59*, 3881-3893.

(3) Chen, A.; Samankumara, L. P.; Dodlapati, S.; Wang, D.; Adhikari, S.; Wang, G. Syntheses of Bis-Triazole Linked Carbohydrate Based Macrocycles and Their Applications for Accelerating Copper Sulfate Mediated Click Reaction. *Eur. J. Org. Chem.* **2019**, *2019*, 1189-1194.

(4) Lau, K.; Thon, V.; Yu, H.; Ding, L.; Chen, Y.; Muthana, M. M.; Wong, D.; Huang, R.; Chen, X. Highly efficient chemoenzymatic synthesis of β1-4-linked galactosides with promiscuous bacterial β1-4-galactosyltransferases. *Chem. Commun.* **2010**, *46*, 6066-6068.

(5) Mangunuru, H. P. R.; Yerabolu, J. R.; Wang, G. Synthesis and study of N-acetyl D-glucosamine triazole derivatives as effective low molecular weight gelators. *Tetrahedron Lett.* **2015**, *56*, 3361-3364.

(6) Li, T.; Guo, L.; Zhang, Y.; Wang, J.; Li, Z.; Lin, L.; Zhang, Z.; Li, L.; Lin, J.; Zhao, W.; Li, J.; Wang, P. G. Design and synthesis of O-GlcNAcase inhibitors via 'click chemistry' and biological evaluations. *Carbohydr. Res.* **2011**, *346*, 1083-1092.

(1) Disney, M. D.; Seeberger, P. H. The Use of Carbohydrate Microarrays to Study Carbohydrate-Cell Interactions and to Detect Pathogens. *Chem. Biol.* **2004**, *11*, 1701-1707.

(2) Roy, R.; Kim, J. M. Cu(II)-Self-assembling bipyridyl-glycoclusters and dendrimers bearing the Tn-antigen cancer marker: syntheses and lectin binding properties. *Tetrahedron* **2003**, *59*, 3881-3893.

(3) Chen, A.; Samankumara, L. P.; Dodlapati, S.; Wang, D.; Adhikari, S.; Wang, G. Syntheses of Bis-Triazole Linked Carbohydrate Based Macrocycles and Their Applications for Accelerating Copper Sulfate Mediated Click Reaction. *Eur. J. Org. Chem.* **2019**, *2019*, 1189-1194.

(4) Lau, K.; Thon, V.; Yu, H.; Ding, L.; Chen, Y.; Muthana, M. M.; Wong, D.; Huang, R.; Chen, X. Highly efficient chemoenzymatic synthesis of β1-4-linked galactosides with promiscuous bacterial β1-4-galactosyltransferases. *Chem. Commun.* **2010**, *46*, 6066-6068.

(1) Disney, M. D.; Seeberger, P. H. The Use of Carbohydrate Microarrays to Study Carbohydrate-Cell Interactions and to Detect Pathogens. *Chem. Biol.* **2004**, *11*, 1701-1707.

(2) Roy, R.; Kim, J. M. Cu(II)-Self-assembling bipyridyl-glycoclusters and dendrimers bearing the Tn-antigen cancer marker: syntheses and lectin binding properties. *Tetrahedron* **2003**, *59*, 3881-3893.

(3) Chen, A.; Samankumara, L. P.; Dodlapati, S.; Wang, D.; Adhikari, S.; Wang, G. Syntheses of Bis-Triazole Linked Carbohydrate Based Macrocycles and Their Applications for Accelerating Copper Sulfate Mediated Click Reaction. *Eur. J. Org. Chem.* **2019**, *2019*, 1189-1194.

(4) Lau, K.; Thon, V.; Yu, H.; Ding, L.; Chen, Y.; Muthana, M. M.; Wong, D.; Huang, R.; Chen, X. Highly efficient chemoenzymatic synthesis of β1-4-linked galactosides with promiscuous bacterial β1-4-galactosyltransferases. *Chem. Commun.* **2010**, *46*, 6066-6068.

(5) Mangunuru, H. P. R.; Yerabolu, J. R.; Wang, G. Synthesis and study of N-acetyl D-glucosamine triazole derivatives as effective low molecular weight gelators. *Tetrahedron Lett.* **2015**, *56*, 3361-3364.

(6) Li, T.; Guo, L.; Zhang, Y.; Wang, J.; Li, Z.; Lin, L.; Zhang, Z.; Li, L.; Lin, J.; Zhao, W.; Li, J.; Wang, P. G. Design and synthesis of O-GlcNAcase inhibitors via 'click chemistry' and biological evaluations. *Carbohydr. Res.* **2011**, *346*, 1083-1092.
